# Supplementary material for: Time intervals and routes to diagnosis for lung cancer in 10 jurisdictions: cross-sectional study findings from the International Cancer Benchmarking Partnership (ICBP)
Source: BMJ Open. 2019 Nov 27;9(11):e025895. doi: 10.1136/bmjopen-2018-025895 (PMC6886977; doi:10.1136/bmjopen-2018-025895)
Supplement: Supplementary data [file bmjopen-2018-025895supp001.pdf]

Supplementary Web Appendix

Contents

Appendix A. Lung cancer questionnaires

A1: Patient questionnaire……………………………………………………....................……….2

A2: Primary care physician (PCP) questionnaire………………………………………………...16

A3: Cancer treatment specialists (CTS) questionnaire ……………………………………………24

Appendix B . Rules for missing, incomplete, multiple response and out of range data……………….…32

Appendix C . Supplementary Tables

C1: Classification of lung cancer -specific symptoms reported by patients into cancer specific and non- cancer specific……………………………………………..............................39

C2: Time intervals (days) depicted as median, 75th and 90th centiles for each of the ten jurisdictions. In Sweden, no data was available on primary care interval……………………………40

C3. Data sources used to define dates and percentage of imputed dates…….41

C4: Percentages of negative intervals set to 0, large intervals (>365 days) set to 365 days, and intervals based not on dates…………………………………………………………42

Appendix A: Lung cancer questionnaires

A1: Patient questionnaire

ICBP
International Cancer Benchmarking Partnership

International Cancer Benchmarking Partnership Module 4

Patient questionnaire
Lung Cancer

Thank you very much for taking the time to fill in this questionnaire – it should take about 20 minutes to complete. We are sending the questionnaire to a large sample of people who we understand have had a diagnosis of lung cancer. If this has been sent to you in error and you do not have cancer, please do not continue and return the documents in the prepaid envelope.

Sample

Our aim is to gain a better understanding of the process by which people have their cancer diagnosed. We would also like to find out more about the symptoms they experience (if any), and the pathway they follow from start of symptoms to treatment of their cancer. This will help in identifying ways in which cancers can be diagnosed quickly and effectively. Thank you once again for your time.

This information is confidential and will not be passed to anyone involved in your treatment.

Name: ____________________

Date of Birth: ____________________

Address: ____________________

Copyright property of the International Benchmarking Partnership (ICBP)
and Module 4 collaborators. Usage must be authorised

ICBP
International Cancer Benchmarking Partnership

Consent form

Please read the consent form and sign your name and date **BELOW**.

If you require any clarification, please do not hesitate to ring the study team members. Their contact details are found on the information sheet.

Please be reassured that your responses are completely confidential and will not be passed to anyone involved in your treatment. For the purposes of the study it is important that you agree to consent to all the statements listed below.

- I confirm that I have read the attached information sheet and I understand why the research is being done.
- I am willing for the team to request information from my GP and hospital doctors which is relevant to the audit as described in the information sheet.
- I give permission for my details (name, address) to be given to the cancer registry (NHS Information Centre for Health and Social Care) for follow up.
- I agree for the information I have provided and any other relevant information from my medical records to be stored as described in the information sheet under the custodianship of University College London.
- I consent to sharing of coded data which contains no personal identifiers between researchers, some of whom are located outside the European Union.
- I consent for use of my data if I become mentally incapacitated during the course of the project.

I agree to all the statements listed and consent to participate in the study.

Name (Please print)

Signature: Date:

If we have any questions, may we phone you for clarification? (Please tick) ☐ **Yes** ☐ **No**

If **Yes**, please provide your telephone number:

Copyright property of the International Benchmarking Partnership (ICBP) and Module 4 collaborators. Usage must be authorised

Sample

ICBP
International Cancer Benchmarking Partnership

1. **Please can you confirm the details of your GP/GP practice (name, practice address – as best as you can remember): We appreciate that you may have more than one GP involved in your care – in which case, we are interested in the GP you would say provides the majority of your care, particularly relating to the cancer you've had diagnosed.**

Name of doctor ______

Name of practice ______

Address ______

______

______

Postcode ______

Town ______

Sample

Copyright property of the International Benchmarking Partnership (ICBP) and Module 4 collaborators. Usage must be authorised

ICBP
International Cancer Benchmarking Partnership

2. Which of the following best describes the events which led to your diagnosis of cancer? (please tick only **ONE** answer) ✓

I had symptoms/I noticed a bodily change and went to see a doctor (e.g. GP)	
I had symptoms/I noticed a bodily change and went/was taken to Accident and Emergency (A&E)	
I had seen a doctor/GP with symptoms, but went/was taken to Accident and Emergency (A&E) when things worsened	
I was being investigated by my doctor(s) for another problem during which time the cancer was discovered	
Other (please describe):	

Sample

Copyright property of the International Benchmarking Partnership (ICBP) and Module 4 collaborators. Usage must be authorised

ICBP
International Cancer Benchmarking Partnership

3. The following health concerns or symptoms are commonly experienced with lung cancer.

Cough
Breathlessness
Being short of breath
Chest pain / Pain on breathing
Coughing up blood
Bloody sputum/blood in spit
Unexplained weight loss
Loss of appetite
Fatigue

Sample

Please write down ALL health concern(s) or symptom(s) you may have had before contacting a doctor or taking part in screening. It does not matter if they are not included in the list above:

Please write your health concern(s) or symptom(s) in the boxes below:
1)
2)
3)
4)
5)
6)

	✓
This is not applicable to me (e.g. I did not have any symptoms), please tick	

Copyright property of the International Benchmarking Partnership (ICBP)
and Module 4 collaborators. Usage must be authorised

ICBP
International Cancer Benchmarking Partnership

4. Please write down your best estimate of the date you noticed the first of these health concern(s) or symptom(s). If you cannot remember the exact date, you can fill in the month and the year.

Day (optional), month, year

D	D	M	M	Y	Y	Y	Y

	✓
This is not applicable to me (e.g. I had no symptoms), please tick	

5. Approximately how long did you have health concern(s) or symptom(s) before contacting a doctor? (Please think of the first visit to the doctor, not re-visits after that). Please tick only **ONE** answer.

	✓
Less than 1 week	
1-2 weeks	
3-4 weeks	
5-7 weeks	
2-5 months	
6-12 months	
More than 12 months	

Sample

	✓
This is not applicable to me (e.g. I had no symptoms), please tick	

Copyright property of the International Benchmarking Partnership (ICBP) and Module 4 collaborators. Usage must be authorised

ICBP
International Cancer Benchmarking Partnership

6a. Once you contacted a practice about your health concern(s) or symptom(s), how long did it take to get an appointment with a doctor? (Please think of the first visit to the doctor, to discuss your health concern(s) or symptom(s)). Please tick only **ONE** answer.

	✓
Same day/next day	
Within 1 week	
1-2 weeks	
3-4 weeks	
Longer	
If there was no waiting time (e.g. you went/were taken to A&E), please tick this box	
This is not applicable to me (e.g. I had no symptoms), please tick	

Sample

6b. What was the date you first saw your doctor about your health concern(s) or symptom(s)? If you cannot remember the exact date, you can fill in the month and the year.

Day (optional), month, year

D	D	M	M	Y	Y	Y	Y

	✓
This is not applicable to me (e.g. I had no symptoms), please tick	

Copyright property of the International Benchmarking Partnership (ICBP) and Module 4 collaborators. Usage must be authorised

ICBP
International Cancer Benchmarking Partnership

7. How many times did you visit the following for the investigation of your symptoms before your cancer was diagnosed?

	Please write down the number of visits
GP	
Hospital	
Consultant/specialist outside of a hospital	

	✓
This is not applicable to me (e.g. I had no symptoms), please tick	

8a. After your doctor referred you to a specialist, how long did it take you to get an appointment? Please tick only **ONE** answer.

Sample

	✓
Less than 1 week	
1-2 weeks	
3-4 weeks	
5-7 weeks	
2-5 months	
6-12 months	
More than 12 months	

	✓
This is not applicable to me (e.g. my doctor did not refer me), please tick	

Copyright property of the International Benchmarking Partnership (ICBP)
and Module 4 collaborators. Usage must be authorised

ICBP
International Cancer Benchmarking Partnership

8b. What was the date of your first appointment with a doctor, involved in investigating and/or treating your cancer, to whom you were referred?
If you cannot remember the exact date, you can fill in the month and the year.

Day (optional), month, year

D	D	M	M	Y	Y	Y	Y

	✓
This is not applicable to me (e.g. my doctor did not refer me), please tick	

9. What was the date you were told you had cancer? If you cannot remember the exact date, you can fill in the month and the year.

Day (optional), month, year

D	D	M	M	Y	Y	Y	Y

Sample

Copyright property of the International Benchmarking Partnership (ICBP) and Module 4 collaborators. Usage must be authorised

ICBP
International Cancer Benchmarking Partnership

10. Have you had any of the following treatments for your cancer yet? If so, please can you estimate the date this treatment started? Please tick **ALL** that apply. If you cannot remember the exact date, you can fill in the month and the year.

	Type of treatment		Date of treatment (give first date if you had more than one)
a.	Surgery	☐ Yes ☐ No	Day (optional), month, year D D M M Y Y Y Y
b.	Chemotherapy	☐ Yes ☐ No	Day (optional), month, year D D M M Y Y Y Y
c.	Radiotherapy	☐ Yes ☐ No	Day (optional), month, year D D M M Y Y Y Y
d.	Other Please specify:	☐ Yes ☐ No	Day (optional), month, year D D M M Y Y Y Y
e.	Treatment not started yet	☐ Yes	

Sample

Copyright property of the International Benchmarking Partnership (ICBP) and Module 4 collaborators. Usage must be authorised

ICBP
International Cancer Benchmarking Partnership

11. Who is the consultant doctor who has taken responsibility for diagnosing and or/treating your cancer?

Name of consultant:

Hospital name:

Hospital department:

Please can you answer some more general questions about your health?
It will help us in interpreting your responses to this questionnaire to know about your general health and other health problems you may have had in the past.

12. Looking back to the 2 years before you were diagnosed with cancer, would you say your general health was (Please tick only **ONE** answer.): ✓

Very good	
Good	
Fair	
Poor	
Very poor	

Sample

13. Have you been treated before for any of the conditions below?
Please tick 'yes' or 'no' for each condition:

Heart disease	☐ **Yes**	☐ **No**
Stroke	☐ **Yes**	☐ **No**
Lung disease (excluding lung cancer)	☐ **Yes**	☐ **No**
Diabetes	☐ **Yes**	☐ **No**

Copyright property of the International Benchmarking Partnership (ICBP) and Module 4 collaborators. Usage must be authorised

ICBP
International Cancer Benchmarking Partnership

Finally, a little more information about you. The information you provide below will help us to analyse the results of the survey in more detail.

14. Which of these best describes your ethnic group? (please tick one box, as appropriate). If you are descended from more than one ethnic or racial group, please tick the group you consider you belong to, or tick 'any other ethnic group'.

	✓		✓		✓		✓
White		Chinese		Black - Caribbean		Black - African	
Black - other		Indian		Pakistani		Bangladeshi	
Any other ethnic group, please specify:							

Sample

15. What is the main language spoken in your home? Please tick

	✓
English	
Other, please specify:	

16. What is the highest level of education you have achieved?
Please tick only **ONE** answer.

	✓
Finished school at or before the age of fifteen	
Completed GCSEs, O-levels or equivalent	
Completed A Levels or equivalent	
Completed further education but not a degree	
Completed a Bachelor's degree / Masters degree / PhD	
Other, please specify:	

Copyright property of the International Benchmarking Partnership (ICBP) and Module 4 collaborators. Usage must be authorised

ICBP
International Cancer Benchmarking Partnership

17. Have you ever smoked cigarettes, including hand-rolled ones, pipes or cigars?

☐ Yes ☐ No

18. Are you a current smoker, smoking either cigarettes, including hand-rolled ones, pipes or cigars?

☐ Yes ☐ No

19. If you are a current smoker or have smoked in the past, how many cigarettes, including hand-rolled ones, pipes or cigars on average do you smoke/have you smoked per day?

Number per day:	

Sample

Copyright property of the International Benchmarking Partnership (ICBP) and Module 4 collaborators. Usage must be authorised

Menon U, *et al. BMJ Open* 2019; 9:e025895. doi: 10.1136/bmjopen-2018-025895

ICBP
International Cancer
Benchmarking Partnership

20. Further comments

Please add anything else that you would like to tell us about your cancer diagnosis or treatment.

Sample

Thank you very much for taking the time to complete this questionnaire.

Copyright property of the International Benchmarking Partnership (ICBP)
and Module 4 collaborators. Usage must be authorised

A2: Primary care physician (PCP) questionnaire

ICBP
International Cancer Benchmarking Partnership

International Cancer Benchmarking Partnership Module 4

Primary Care Audit
Lung Cancer

Thank you very much for agreeing to fill in this questionnaire. As part of an international study examining differences in cancer survival, we are sending the questionnaire to health care providers of a sample of consented patients with cancer.

Our aim is to gain a better understanding of the process by which people have their cancer diagnosed – the symptoms they experience, and the pathway they follow from onset of symptoms to treatment of their cancer. This will help in identifying ways in which cancers can be diagnosed and treated quickly and effectively. Thank you once again for your time.

Sample

Please can you refer to your patient's notes in completing the questionnaire as this will help in obtaining accurate data on time points.

If you would prefer to return this questionnaire without the patient details, please tear off along the dotted line

ID-number: Jurisdiction-ID + Patient-ID: ______________________

Copyright property of the International Benchmarking Partnership (ICBP)
and Module 4 collaborators. Usage must be authorised

ICBP
International Cancer Benchmarking Partnership

Sample

Patient information

ID-number: Jurisdiction-ID + Patient-ID:

Full name:

Address:

Postcode:

Date of birth: D D M M Y Y Y Y

Copyright property of the International Benchmarking Partnership (ICBP) and Module 4 collaborators. Usage must be authorised

Menon U, *et al. BMJ Open* 2019; 9:e025895. doi: 10.1136/bmjopen-2018-025895

ICBP
International Cancer Benchmarking Partnership

1. Duration of symptoms

Please estimate how long your patient had symptom(s), attributable to lung cancer, before attending your practice (or other health service).

We appreciate that identifying a 'date of first symptom' is not always straightforward – particularly when there are multiple and/or chronic symptoms. Nevertheless, we hope you can provide a 'best estimate'.

**Estimate of symptom duration** (please tick **one**):	✓	**What were the symptoms?** Please describe:
Less than 1 week		
1 to 4 weeks		
5 to 7 weeks		
2-5 months		
6-12 months		
More than 12 months		
Not possible to estimate		
No symptoms (e.g. screen detected cancers)		

Sample

Copyright property of the International Benchmarking Partnership (ICBP) and Module 4 collaborators. Usage must be authorised

ICBP
International Cancer Benchmarking Partnership

2. Pathway of presentation

2.1 Through what route did the patient first present? Please tick **ONE**:

	✓	
Your patient first presented to primary care (either in-hours or out-of-hours)		Please can you provide your best approximation of the date of this **primary care** visit<br>D D M M Y Y Y Y
Your patient presented straight to A&E (with or without your involvement)		
Your patient first presented to primary care, but then at a later date presented to A&E as an emergency (with or without your involvement)		Please can you provide your best approximation of the date of this **primary care** visit<br>D D M M Y Y Y Y
Other – please describe:		

Sample

Copyright property of the International Benchmarking Partnership (ICBP)
and Module 4 collaborators. Usage must be authorised

ICBP
International Cancer Benchmarking Partnership

3. Date you ordered any tests/investigations in response to symptom(s).

We are interested in any kind of tests/investigations (e.g. imaging etc) that you may have ordered. Please only consider the tests/investigations that you ordered yourself. Please tick **all** that apply and put in the date that the test/investigation was ordered:

	✓	
Chest x-ray		D D M M Y Y Y Y
MRI scan		D D M M Y Y Y Y
CT scan		D D M M Y Y Y Y
PET scan		D D M M Y Y Y Y
Sputum test		D D M M Y Y Y Y
Lung biopsy		D D M M Y Y Y Y
Bronchoscopy		D D M M Y Y Y Y
Other (please specify):		D D M M Y Y Y Y

Sample

4. Date of referral to specialist medical services

At what date did you **first** refer the patient to hospital or another specialist transferring the responsibility for on-going investigation/treatment to other medical services?

D D M M Y Y Y Y

Copyright property of the International Benchmarking Partnership (ICBP) and Module 4 collaborators. Usage must be authorised

ICBP
International Cancer Benchmarking Partnership

5. Nature of this referral

5.1 Do you know the date that the patient was seen for this referral?

☐ **Yes**, please provide the date: D D M M Y Y Y Y

☐ **No**

5.2 If you did make a referral to specialist services, which of the following best describes the nature/characteristics of this referral? Please tick **one**.

	✓
Emergency admission: a referral to A&E (or equivalent) for immediate admission	
An urgent referral for assessment of cancer symptoms/signs/test results (Note this will be within 2 weeks for England/Wales)	
A less urgent referral in which cancer is raised as a possibility (Note this will be greater than 2 weeks for England/Wales)	
A more general referral for investigation and assessment without cancer mentioned	
No referral was made	
Other – please describe	

Sample

5.3 Would you say this patient's diagnostic pathway was conducted predominantly in the public or private system? Please tick **one**.

	✓
Public healthcare system	
Private healthcare system	

Copyright property of the International Benchmarking Partnership (ICBP) and Module 4 collaborators. Usage must be authorised

ICBP
International Cancer Benchmarking Partnership

6. Date of lung cancer diagnosis

This can be decided in different ways. Please tick **all** that apply.
Please provide whichever of the following dates you have to hand:

	✓	
Date of histological confirmation [ideal]		D D M M Y Y Y Y
Date results of investigation (histological or other) confirming cancer received		D D M M Y Y Y Y
Date patient was told		D D M M Y Y Y Y
Date biopsy undertaken		D D M M Y Y Y Y
Date patient was first admitted to hospital because of the malignancy		D D M M Y Y Y Y
Other (please specify)		D D M M Y Y Y Y

Sample

Copyright property of the International Benchmarking Partnership (ICBP) and Module 4 collaborators. Usage must be authorised

ICBP
International Cancer Benchmarking Partnership

7. Additional information

Finally, we are interested to know what other conditions your patient has, and the severity/impact of these conditions

Have you and/or any of your partners treated this patient (or has the patient been to hospital) for any of the following conditions? Please tick **all** that apply:

Cardiovascular disease	☐ Yes ☐ No
Stroke	☐ Yes ☐ No
Lung disease (except lung cancer)	☐ Yes ☐ No
Diabetes	☐ Yes ☐ No

Sample

Are there any other comments you would like to make about this patient?

Name (and title):

Signature:

Date:

Thank you very much for taking the time to complete this questionnaire.

Copyright property of the International Benchmarking Partnership (ICBP)
and Module 4 collaborators. Usage must be authorised

A3: Cancer treatment specialists (CTS) questionnaire

ICBP
International Cancer Benchmarking Partnership

International Cancer Benchmarking Partnership Module 4

Specialist Care Audit
Lung Cancer

Thank you very much for agreeing to fill in this questionnaire – it should take about 10 minutes to complete. As part of an international study examining differences in cancer survival, we are sending the questionnaire to health care providers of a sample of patients with cancer.

Our aim is to gain a better understanding of the process by which people have their cancer diagnosed – the symptoms they experience, and the pathway they follow from onset of symptoms to treatment of their cancer. We hope you can help us with information on this patient's cancer journey once they were referred to specialist cancer services. This will help in identifying ways in which cancers can be diagnosed and treated quickly and effectively.

Sample

Thank you once again for your time

Please can you refer to your patient's notes in completing the questionnaire, as this will help in obtaining accurate data on time points.

If you would prefer to return this questionnaire without the patient details, please tear off along the dotted line.

Your patient ____

is participating in the study.

Copyright property of the International Benchmarking Partnership (ICBP) and Module 4 collaborators. Usage must be authorised

ICBP
International Cancer
Benchmarking Partnership

Sample

Patient information

ID-number: Jurisdiction-ID + Patient-ID:

Full name:

Address:

Postcode:

Date of birth: D D M M Y Y Y Y

Copyright property of the International Benchmarking Partnership (ICBP)
and Module 4 collaborators. Usage must be authorised

Menon U, *et al. BMJ Open* 2019; 9:e025895. doi: 10.1136/bmjopen-2018-025895

ICBP
International Cancer Benchmarking Partnership

1. **Date patient first attended hospital/specialist services related to their cancer diagnosis.** We appreciate this date can at times be difficult to identify, particularly when there have been multiple visits in the lead up to a definitive diagnosis. Put another way, it's the date that the hospital/specialist service assumed responsibility for on-going investigation/treatment for your patient.

Day (optional), month, year

D	D	M	M	Y	Y	Y	Y

2. **How was the patient referred to the hospital/specialist services related to their cancer diagnosis?** Please tick.

Was it through a:

	✓		✓
GP referral		Referral from respiratory clinic	
Medical specialist/Consultant referral		Other referral – please specify:	

Sample

3. **Where did this first contact/appointment happen?** Please tick.
Which of the following best describes where this first contact/appointment took place?

	✓		✓
Emergency department ('A&E')		Medical outpatient department, please specify which department	
Oncology general outpatient department		Surgical outpatient department, please specify which department	
Other – please specify:			

Copyright property of the International Benchmarking Partnership (ICBP) and Module 4 collaborators. Usage must be authorised

Menon U, *et al. BMJ Open* 2019; 9:e025895. doi: 10.1136/bmjopen-2018-025895

ICBP
International Cancer Benchmarking Partnership

4. Date of diagnosis

This can be decided in different ways.
Please tick and complete as many of the following dates as possible.

	✓	
Date of histological confirmation (ideal)		Day (optional), month, year<br>D D M M Y Y Y Y
Date results of investigation confirming cancer received		Day (optional), month, year<br>D D M M Y Y Y Y
Date patient was told		Day (optional), month, year<br>D D M M Y Y Y Y
Date of biopsy		Day (optional), month, year<br>D D M M Y Y Y Y
Date patient was first admitted to hospital because of the malignancy		Day (optional), month, year<br>D D M M Y Y Y Y
Date of MDT confirmation of diagnosis		Day (optional), month, year<br>D D M M Y Y Y Y
Other (please specify):		Day (optional), month, year<br>D D M M Y Y Y Y

Sample

Copyright property of the International Benchmarking Partnership (ICBP)
and Module 4 collaborators. Usage must be authorised

ICBP
International Cancer Benchmarking Partnership

5. Date treatment for the cancer commenced

Based on your records, when would you say that any treatment specifically targeting the patient's cancer started?

Day (optional), month, year

D	D	M	M	Y	Y	Y	Y

6. Additional information

Please can you provide any further information on the patient's cancer:

**TNM**, please tick as appropriate:	
0	
I	
IIA	
IIB	
IIC	
IIIA	
IIIB	
IIIC	
IV	
Not able to stage	

Sample

Copyright property of the International Benchmarking Partnership (ICBP)
and Module 4 collaborators. Usage must be authorised

ICBP
International Cancer Benchmarking Partnership

6.1 Histological subtype:

	✓
Squamous cell carcinoma	
Adenocarcinoma	
Large cell carcinoma	
Non-small carcinoma	
Small cell carcinoma	
Carcinoid	
Unclassified	
Other (please specify):	

Sample

Copyright property of the International Benchmarking Partnership (ICBP)
and Module 4 collaborators. Usage must be authorised

Menon U, *et al. BMJ Open* 2019; 9:e025895. doi: 10.1136/bmjopen-2018-025895

ICBP
International Cancer Benchmarking Partnership

Further comments

Sample

Copyright property of the International Benchmarking Partnership (ICBP) and Module 4 collaborators. Usage must be authorised

ICBP
International Cancer Benchmarking Partnership

Name (and title):

Signature:

Date:

Sample

Are you a … (please tick below): ✓

Surgeon	
Medical Oncologist	
Clinical Oncologist	
Clinical Nurse Specialist	
Other (please specify):	

Thank you very much for taking the time to complete this questionnaire.

Copyright property of the International Benchmarking Partnership (ICBP)
and Module 4 collaborators. Usage must be authorised

Appendix B:

Rules for missing, incomplete, multiple response and out of range data

1. Oversampling<br>To handle oversampling in Ontario, include only the first 315 consecutive lung cancer patients;
2. Language/Participation in study/Presence of cancer<br>Exclude patients who checked "No, I don't understand the language" or "I don't want to participate in this study" or "I don't have cancer".
3. Survey responders<br>a) Exclude Patient/PCP/Specialist survey from the analysis, if it was not written by Patient/PCP/Specialist (example: a medical oncologist completed a PCP survey);<br>b) In the case of duplicates, include only the first survey (example: 2 specialists completed surveys for the same patient).
4. Gender<br>Exclude patients with unknown Gender.
5. Age<br>a) Exclude patients with unknown age;<br>b) Exclude patients younger 40 years;<br>c) Use registry data, if Age is reported by both patient and registry.
6. No cancer or Previous cancer in the same organ<br>a) Exclude patients with no cancer based on registry data;<br>b) Exclude patients with previous cancer in the same organ based on data from registry or free-text for Presentation in the patient survey.
7. Date of consent<br>Exclude patients with date of consent which is unknown, before 01.01.2013 or in the future.
8. Multiple responses to Dates<br>If multiple responses were given to the dates (of first symptom; first presentation to primary care; referral; diagnosis; treatment start), then use the earliest date.
9. Order of Dates<br>The dates must be in the following order –<br>First symptom; first presentation to Primary Care; referral; diagnosis; treatment start.<br>If not, check for mistakes.

10. Date of first symptom
Date of first symptom is defined as date of first symptom from patient data.

11. Date of first presentation
Date of first presentation to Primary Care is defined as (in the order of declining priority):
a) date of first presentation to Primary Care from PCP data;
b) date of first presentation to Primary Care and A&E from PCP data;
c) date of first presentation to Primary Care from patient data.

12. Date of referral
Date of referral is defined as date of referral from PCP data.

13. Date of diagnosis
Definition
a) If Registry reports both date of histological confirmation and date of confirming investigation, then use date of histological confirmation.
b) Date of diagnosis (based on patient data, PCP data, specialist data, registry data) is defined as (in the order of declining priority):
- date of diagnosis from registry;
- date of histological confirmation (from specialist data, PCP data);
- date of biopsy (from specialist data, PCP data);
- date of confirming investigation (from specialist data, PCP data);
- date of first hospital admission (from specialist data, PCP data);
- date of MDT confirmation (from specialist data, PCP data);
- date patient was told (from specialist data, PCP data);
- other date of diagnosis (from specialist data, PCP data, patient data);

Choose a Date from a lower level of hierarchy, if the Date from a higher level is after the Date of consent or more than 9 months (=271 days) before the Date of consent.

Exclusion criteria

a) Unknown date of diagnosis;
b) Date of diagnosis is after the date of consent;
c) Date of diagnosis is more than 9 months before the Date of consent.

14. Date of treatment start
a) Date of treatment start from patient data is defined as the earliest of the treatment dates for Surgery, Chemo, Radio and Other (e.g. palliative care, participation in a clinical trial, targeted agents like erlotinib and procedures like plueral tap)
b) Date of treatment start (based on registry data, specialist data, patient data) is defined as (in the

order of declining priority):
- date of treatment start from registry data,
- date of treatment start from specialist data,
- date of treatment start from patient data,
- anticipated date of treatment from patient data.

15. Imputation of missing day in the date

Imputation rules for missing day (given month and year are known):

a) Set missing day to '16';
b) Consider adjacent dates in a backwards order (from "Treatment" to "First symptom"). For each pair of such adjacent dates: If dates are not in a logical order (e.g. "Treatment" is before "Diagnosis"), but month and year are the same in both dates, and the day was imputed to '16' in one of the dates:
   - Recode the day imputed earlier to '16' to the day from the adjacent date.

16. Considering time

If patient gave multiple answers to the "How long did you have symptoms before contacting a doctor?" question, then use the option with the shortest time interval.

17. Delay arranging appointment

If patient gave multiple answers to the "How long did it take to get an appointment with PCP?" question, then use the option with the shortest time interval.

18. Duration of symptoms

If PCP gave multiple answers to the "Duration of symptoms" question, then use the option with the shortest time interval.

19. Definition of Presentation

A. *Define Presentation within a Data Source*
1. Review the free-text for Presentation (Patient, PCP sources) and re-code, if possible.
2. If PCP reports 'Other' as Presentation and at least one symptom (or "Duration of Symptoms") or if Patient reports 'Other' as Presentation and at least one symptom (or date of first symptom or "Consider waiting time" or "Delay arranging appointment"), then re-code the Presentation in the corresponding data source to 'Unknown'- option.
3. In the case of multiple Presentation responses (Patient, PCP sources) - use a single option (in the order of declining priority):
   a) 'VisitPCP and AE',
   b) 'VisitPCP´, 'AE´ (if both 'VisitPCP´ and 'AE´ are given, then re-code as 'VisitPCP and AE'),
   c) 'Unknown',
   d) 'Investigation for another problem' ,
   e) 'Other"

B. Define Presentation from Alternative Data

If Presentation hasn't been reported in either of data sources, then define it 'Unknown', <br>if PCP reports at least one symptom (or "Duration of symptoms"); or if Patient reports <br>at least one symptom (or date of first symptom or "Consider waiting time" or "Delay arranging <br>appointment"); <br>C. *Define Presentation from Data Source Hierarchy*   1. In all jurisdictions, except Sweden – use Presentation data from (in the order of declining priority):   a) PCP data;   b) Patient data;   2. In Sweden – use Presentation data from Patient data.
20. <u>Patient interval</u>   The Patient interval is defined as (in the order of declining priority):   a) "Date of first presentation to Primary Care" (rule 11) minus "Date of first symptom" (rule 10);   b) If the interval in (a) is unknown or negative: Calculate the interval as the low boundary of "Considering time" (rule 16) plus the low boundary of "Delay arranging appointment" (rule 17);   c) If the interval in (a) is unknown or negative and the interval in (b) is unknown: Calculate the interval as the low boundary of "Duration of symptoms interval" (rule 18).
21. <u>Primary Care interval</u>   The Primary Care interval is defined as "Date of referral" (rule 12) minus "Date of first presentation to Primary Care" (rule 11).
22. <u>Diagnostic interval</u>   a) The Diagnostic interval is defined as "Date of diagnosis" (rule 13) minus "Date of first presentation to Primary Care" (rule 11).
23. <u>Treatment interval</u>   The Treatment interval is defined as "Date of treatment start" (rule 14) minus "Date of diagnosis" (rule 13).
24. <u>Total interval</u>   a) The Total interval is defined as "Date of treatment start" (rule 14) minus "Date of first symptom" (rule 10).
25. <u>Range of Time intervals</u>   The time intervals (Patient, Primary Care, Diagnosis, Treatment, Total) must be in range 0-1 year. <br>If > 1 year: set the interval to 365 days   If negative: set the interval to 0.

26. Number of visits<br>If patient gave multiple answers to the "Number of visits" questions, then use the option with a fewer number of visits.
27. Specialist waiting time interval<br>If patient gave multiple answers to the ""How long did it take to get an appointment with specialist?" question, then use the option with the shortest time interval.
28. Type of treatment<br>If patient ticked both "Yes" and "No" as answers to the "Type of treatment (Surgery, Chemotherapy, Radiotherapy)" questions, then choose "Yes" answer.
29. Health state<br>If patient gave multiple answers to the "Health state" question, then use the option with a better health condition.
30. Comorbidity<br>a) If patient ticked both "Yes" and "No" as answers to the "Comorbidity (Heart disease, Stroke, Lung disease, Diabetes)" questions, then choose "Yes" answer;<br>b) If both patient and PCP report "Comorbidity", then use the PCP Data.
31. Ethnicity<br>a) If patient didn't report "Ethnicity", then use the information from (in the order of declining priority):<br>- "Ethnicity_Other_Details";<br>- "Other main language spoken at home";<br>- "The main language spoken at home" (only for Victoria);<br>- "The main language spoken at home is the chief one for this jurisdiction"="Yes" given "Main language spoken at home is other than the main one for this jurisdiction"="No";<br>b) Consider Ethnicity as unknown, if answers to the "Ethnicity" question are multiple and belong to different categories ('white', 'Asian', 'black', 'other').
32. Education<br>If patient gave multiple answers to the "Education" question, then use the option with a higher level of education.
33. Smoking Current<br>a) If patient ticked both "Yes" and "No" as answers to the "Smoking Current" question, then use "Yes" answer;<br>b) If patient hasn't ticked neither "Yes" nor "No, then consider this case as Unknown.

34. Smoking Number

If patient reports "SmokingNumber" as text, then re-code using following rules:

a) Where there is a number smoked /day – accept number;
b) Where a range has been given – take the upper value;
c) Where patient has put 10+ or 20+ - capture this as 11 or 21;
d) Where number of cigarettes smoked in the past and currently being smoked are provided - average the numbers;
e) Non entries code as "." ;
f) Non-smokers (eg, "nil", "N/A") are coded as "0".

35. Smoked ever

a) If patient ticked both "Yes" and "No" as answers to the "Smoking ever" question, then use "Yes" answer;
b) If patient hasn't ticked neither "Yes" nor "No": consider it as "Yes", if patient is a current smoker ("Smoking_Current="Yes"") or has specified a number of cigarettes ("SmokingNumber">0). Otherwise consider this case as Unknown.
c) If patient has ticked "No": recode it to "Yes", if patient is a current smoker ("Smoking_Current="Yes").

36. Nature of referral

a) Review free-text for " Nature of referral" (PCP Data) and re-code, if possible;
b) In the case of multiple responses, use a single option as (in the order of declining priority):

- "Referral for immediate admission";
- "Urgent referral";
- "Less urgent referral";
- "General referral" ;
- "No referral";
- "Other".

37. Refer Public or Private

a) If PCP ticked both "Public" and "Private" as answers to the "Refer Public or Private" question, then use "Private" answer;
b) If PCP hasn't ticked neither "Public" nor "Private", then consider this case as Unknown.

38. Type of referral

If specialist gave multiple responses to the "How was the patient referred…" question, then use a single option (in the order of declining priority):

- "Respiratory clinic";
- "Specialist/consultant";
- "PCP";
- "Other".

37

39. <u>First Attendance Place</u><br>If specialist gave multiple responses to the “First Attendance Place” question, then consider this case as Unknown.
40. <u>Stage-TNM</u><br>a) If specialist gave multiple responses to the “Stage_TNM” question, then use the highest category;<br>b) If registry gave multiple responses to the “Stage_TNM”, then use a single option (in the order of declining priority):<br>- stage at time of diagnosis<br>- stage at surgery<br>- stage at oncology<br>c) If “Stage_TNM” is reported by both the specialist and registry, then use the registry data.

Appendix C: Supplementary Tables

C 1: Classification of lung cancer -specific symptoms reported by patients into cancer specific and non- cancer specific

Cancer specific symptoms

1 persistent cough
2 chest infection that wouldn't get better
3 coughing up blood-stained phlegm (sputum)
4 breathlessness
5 chest or shoulder pain
6 a hoarse voice
7 difficulty swallowing
8 swelling of lymph nodes (glands) in my neck
9 ends of fingers becoming larger
10 breast and axilla swelling (in men)

Non-cancer specific symptoms

1 a dull ache or sharp pain when I coughed or took a deep breath
2 pain/discomfort under my ribs
3 face swelling
4 blood clots (thrombosis)
5 pins and needles or numbness in fingers
6 weight loss
7 felt sick/vomiting/nausea/loss of appetite
8 fatigue
9 muscle weakness
10 drowsiness, weakness, dizziness or confusion
11 high temperature (fever) of 38C (100.4F)
12 other

C2: Time intervals (days) depicted as median, 75th and 90th centiles for each of the ten jurisdictions. In Sweden, no data was available on primary care interval

Menon U, *et al. BMJ Open* 2019; 9:e025895. doi: 10.1136/bmjopen-2018-025895

Intervals	Centiles	Wales	Denmark	Sweden	England	N Ireland	Scotland	Manitoba	Ontario
Patient Interval	Number	181	233	172	213	179	169	133	205
	Median	21	14	21	17	18	21	25	22
	75th centile	61	53	61	65	60	60	67	61
	90th centile	216	180	214	205	240	267	180	187
Primary Care interval	Number	110	159	n/a	147	124	119	80	75
	Median	20	7		11	13	16	30	29
	75th centile	43	20		31	51	35	75	73
	90th centile	91	64		73	112	90	138	183
Diagnostic interval	Number	176	229	165	212	170	173	138	212
	Median	45	35	28	54	65	42	87	57
	75th centile	108	67	83	100	122	106	147	122
	90th centile	162	162	143	161	281	198	265	331
Treatment interval	Number	192	279	190	238	200	187	182	263
	Median	43	16	34	22	32	42	19	47
	75th centile	64	25	59	41	48	62	56	70
	90th centile	89	37	77	56	72	90	97	96
Total interval	Number	147	192	147	176	157	143	117	178
	Median	116	67	107	114	105	117	127	130
	75th centile	204	116	190	183	227	253	216	216
	90th centile	365	210	329	323	365	365	365	339

C3: Data sources used to define dates and percentage of imputed dates

Type of date	Data sources used to define a date* (%)				Cases with imputed day in a date** (%)
	Patient	PCP	CST	Registry	
First noticing symptoms	100	0	0	0	66
First presentation to health care	49	51	0	0	30
First referral to secondary care	0	100	0	0	1
Diagnosis	5	6	8	81	1
Start of curative or palliative treatment	55	0	32	13	11

* based on rules 10-14, supplementary file Appendix B
** based on rule 15, supplementary file Appendix B
*** Registry/CST medical records on date of treatment were not available for 55% patients, therefore an alternative data source (patient survey) was used instead

Menon U, *et al. BMJ Open* 2019; 9:e025895. doi: 10.1136/bmjopen-2018-025895

C4. Percentages of negative intervals set to 0, large intervals (>365 days) set to 365 days, and intervals based not on dates

**Type of interval**	**Negative intervals set to 0 days* (%)**	**Intervals >365 days set to 365 days* (%)**	**Intervals where variables other than dates were used (%)****
Patient	<1	5	29
Primary care	4	2	0
Diagnosis	6	5	0
Treatment	6	<1	0
Total	2	9	0

* based on rule 25, supplementary file Appendix B
* based on rule 20b,c, supplementary file Appendix B

Menon U, *et al. BMJ Open* 2019; 9:e025895. doi: 10.1136/bmjopen-2018-025895
