# Supplementary material for: Time intervals and routes to diagnosis for lung cancer in 10 jurisdictions: cross-sectional study findings from the International Cancer Benchmarking Partnership (ICBP)
Source: BMJ Open. 2019 Nov 27;9(11):e025895. doi: 10.1136/bmjopen-2018-025895 (PMC6886977; doi:10.1136/bmjopen-2018-025895)
Supplement: Author's manuscript [file bmjopen-2018-025895.draft_revisions.pdf]

BMJ Open

BMJ Open is committed to open peer review. As part of this commitment we make the peer review history of every article we publish publicly available.

When an article is published we post the peer reviewers' comments and the authors' responses online. We also post the versions of the paper that were used during peer review. These are the versions that the peer review comments apply to.

The versions of the paper that follow are the versions that were submitted during the peer review process. They are not the versions of record or the final published versions. They should not be cited or distributed as the published version of this manuscript.

BMJ Open is an open access journal and the full, final, typeset and author-corrected version of record of the manuscript is available on our site with no access controls, subscription charges or pay-per-view fees (http://bmjopen.bmj.com).

If you have any questions on BMJ Open's open peer review process please email info.bmjopen@bmj.com

BMJ Open

Time intervals and routes to diagnosis for lung cancer in ten jurisdictions: cross-sectional study findings from the International Cancer Benchmarking Partnership (ICBP)

Journal:	*BMJ Open*
Manuscript ID	bmjopen-2018-025895
Article Type:	Research
Date Submitted by the Author:	14-Aug-2018

Complete List of Authors:	Menon, Usha; University College London, Institute for Women's Health<br>Vedsted, Peter; Aarhus University, Research Unit for General Practice<br>Zalounina Falborg, Alina; Aarhus University, Research Unit for General Practice<br>Jensen, Henry; Aarhus University, Research Unit for General Practice<br>Harrison, Samantha; Cancer Research UK, Policy & information<br>Reguilon, Irene; Cancer Research UK, Policy & information<br>Barisic, Andriana; Cancer Care Ontario<br>Bergin, Rebecca; Cancer Council Victoria, Centre for Behavioural Research in Cancer; University of Melbourne, Department of General Practice<br>Brewster, David; Edinburgh University, Centre for Population Health Sciences; NHS Scotland National Services Division, Scottish Cancer Registry, Information Services Division<br>Butler, John; The Royal Marsden<br>Brustugun, Odd Terje; Olso University Hospital, Institute for Cancer Research<br>Bucher, Oliver; CancerCare Manitoba, Department of Epidemiology and Cancer Registry<br>Cairnduff, Victoria; Queen's University Belfast, Northern Ireland Cancer Registry<br>Gavin, Anna; Queen's University Belfast, Northern Ireland Cancer Registry<br>Grunfeld, Eva; Ontario Institute for Cancer Research, Health Services Research Program<br>Harland, Elizabeth; CancerCare Manitoba, Department of Epidemiology and Cancer Registry<br>Kalsi, Jatinderpal; University College London, Institute for Women's Health<br>Knudsen, Anne Kari; Olso University Hospital, European Palliative Care Research Centre (PRC); University of Oslo, Institute of Clinical Medicine<br>Lambe, Mats; Karolinska Institutet, Department of Medical Epidemiology; University Hospital, Regional Oncologic Center<br>Law, Rebecca-Jane; Bangor University, North Wales Centre for Primary Care Research<br>Lin, Yulan; University of Oslo, Institute of Clinical Medicine; Olso University Hospital, European Palliative Care Research Centre (PRC)<br>Malmberg, Martin; Skanes universitetssjukhus Lund<br>Turner, Donna; CancerCare Manitoba<br>Neal, RD; Bangor University, North Wales Centre for Primary Care Research; University of Leeds, Academic Unit of Primary Care<br>White, Victoria; Cancer Council Victoria, Centre for Behavioural Research in Cancer; Deakin University, School of Psychology

Weller, David; Edinburgh University, Centre for Population Health Sciences

Keywords: EPIDEMIOLOGY, International health services < HEALTH SERVICES ADMINISTRATION & MANAGEMENT, Organisation of health services < HEALTH SERVICES ADMINISTRATION & MANAGEMENT, Respiratory tract tumours < ONCOLOGY

SCHOLARONE™ Manuscripts

For peer review only

Time intervals and routes to diagnosis for lung cancer in ten jurisdictions: cross-sectional study findings from the International Cancer Benchmarking Partnership (ICBP)

1. Usha Menon*, Institute for Women's Health, University College London, London, United Kingdom
2. Peter Vedsted*, Research Unit for General Practice, Aarhus University, Aarhus, Denmark
3. Alina Zalounina Falborg, Research Unit for General Practice, Aarhus University, Aarhus, Denmark
4. Henry Jensen, Research Unit for General Practice, Aarhus University, Aarhus, Denmark
5. Samantha Harrison, Policy and Information, Cancer Research UK, London, United Kingdom
6. Irene Reguilon, Policy and Information, Cancer Research UK, London, United Kingdom
7. Andriana Barisic, Ontario Renal Network, Cancer Care Ontario, Toronto, Canada
8. Rebecca J Bergin, Centre for Behavioural Research in Cancer, Cancer Council Victoria, Victoria, Australia; Department of General Practice, University of Melbourne, Victoria, Australia
9. David H Brewster, Scottish Cancer Registry, Information Services Division, NHS National Services Scotland, Edinburgh, United Kingdom; Centre for Population Health Sciences, University of Edinburgh, Edinburgh, United Kingdom
10. John Butler, The Royal Marsden, Fulham Road, London, United Kingdom
11. Odd Terje Brustugun, Institute for Cancer Research, Department of Cancer Genetics, Oslo University Hospital, Oslo, Norway
12. Oliver Bucher, Department of Epidemiology and Cancer Registry, CancerCare Manitoba, Manitoba
13. Victoria Cairnduff, Northern Ireland Cancer Registry, Centre for Public Health, Queen's University Belfast, Mulhouse Building, Mulhouse Road, Belfast, United Kingdom
14. Anna T Gavin, Northern Ireland Cancer Registry, Queen's University Belfast, Belfast, United Kingdom
15. Eva Grunfeld, Health Services Research Program, Ontario Institute for Cancer Research, Toronto, Canada
16. Elizabeth Harland, Department of Epidemiology and Cancer Registry, CancerCare Manitoba, Winnipeg, Canada
17. Jatinderpal Kalsi, Gynaecological Cancer Research Centre, Women's Cancer, Institute for Women's Health, University College London, London, United Kingdom

For peer review only - http://bmjopen.bmj.com/site/about/guidelines.xhtml

18. Anne Kari Knudsen, European Palliative Care Research Centre (PRC), Department of Oncology, Oslo University Hospital and Institute of Clinical Medicine, University of Oslo, Oslo, Norway
19. Mats Lambe, Regional Cancer Center Uppsala and Department of Medical Epidemiology and Biostatics, Karolinska Institutet, Stockholm, Sweden
20. Rebecca-Jane Law, North Wales Centre for Primary Care Research, Bangor University, Wrexham, United Kingdom
21. Yulan Lin, European Palliative Care Research Centre (PRC), Department of Oncology, Oslo University Hospital and Institute of Clinical Medicine, University of Oslo, Oslo, Norway
22. Martin Malmberg, Department of Oncology, Lund University Hospital, Lund, Sweden
23. Donna Turner, Population Oncology, Cancer Care Manitoba, Winnipeg, Canada
24. Richard D Neal, North Wales Centre for Primary Care Research, Bangor University, Wrexham, United Kingdom; Academic Unit of Primary Care, University of Leeds, Leeds, United Kingdom
25. Victoria White, Centre for Behavioral Research in Cancer, Cancer Council Victoria, Victoria, Australia; School of Psychology Deakin University, Geelong, Victoria, 3217, Australia
26. David Weller*, Centre for Population Health Sciences, University of Edinburgh, Edinburgh, United Kingdom

On behalf of the ICBP Module 4 Working Group

* equal contribution

Corresponding author:
Professor Usha Menon
MRC CTU at UCL
Institute of Clinical Trials and Methodology
90 High Holborn, 2nd Floor, London WC1V 6LJ
Email: u.menon@ucl.ac.uk

Word count: 3074; Tables: 6 ; Figures: 2 ; Supplementary Files: 6

For peer review only

Abstract

Objective

Differences in time intervals to diagnosis and treatment between jurisdictions may contribute to previously reported differences in stage at diagnosis and survival. The International Cancer Benchmarking Partnership Module 4 (ICBP M4) reports the first international comparison of routes to diagnosis and time intervals from symptom onset until treatment start for lung cancer patients.

Design

Patients, their primary care physicians (PCP) and cancer treatment specialists (CTS) were surveyed in Victoria (Australia), Manitoba and Ontario (Canada), Northern Ireland, England, Scotland and Wales (UK), Denmark, Norway and Sweden. Using Wales as the reference jurisdiction, the 50th, 75th and 90th percentiles for intervals were compared using quantile regression adjusted for age, gender and comorbidity.

Participants

Consecutive newly diagnosed lung cancer patients, aged >40 years, identified through cancer registries. Of 10,203 eligible patients contacted, 2,631 (25.8%) responded and 2,143 were included in the analysis.

Primary and secondary outcome measures:

Interval lengths (days, primary), routes to diagnosis, symptoms (secondary).

Results

With the exception of Denmark, in all other jurisdictions the median total interval from symptom onset to treatment, for respondents diagnosed in 2012-15, was similar to that of Wales. Overall 55% (range 35-75%) were diagnosed following presentation to the PCP. Jurisdiction specific significant differences in patient, diagnostic and treatment intervals, especially for the 10% of patients who waited the longest, were observed. Based on overall trends, jurisdictions could be grouped into those with trends of reduced, longer and similar intervals to Wales. All symptoms other than persistent cough were less frequently reported by the PCP when compared to patients.

Conclusion

For peer review only - http://bmjopen.bmj.com/site/about/guidelines.xhtml

There are differences between jurisdictions in interval lengths, especially for patients who wait the longest. The data will allow jurisdictions to develop more focused lung cancer policy and clinical initiatives. Future analysis will explore if these differences in intervals impact on stage or survival.

Key words:
lung cancer, routes to diagnosis, time intervals, international health systems, symptomatic presentation

For peer review only

For peer review only - http://bmjopen.bmj.com/site/about/guidelines.xhtml

Strengths and limitations of this study

- This is the first international study to use standarised survey methods and definitions to systematically examine key intervals from patients first noticing symptoms or bodily changes until the start of treatment for lung cancer
- Recall bias was minimised by the triangulation of different data sources and by patients completing the questionnaire within a limited time window (median 5 months) after the cancer diagnosis
- A key limitation, as with all questionnaire-based studies was non-response bias which varied across jurisdictions, and analyses were adjusted for age, gender and comorbidity, but not for ethnicity and education as there are different classification systems in participating countries
- Recruitment of patients up to 9 rather than 6 months after diagnosis might have magnified selection bias due to high mortality in lung cancer but a sensitivity analysis suggests that this did not impact on the results.
- In Norway and Victoria, a small sample size and restriction of eligibility to only surgical patients, respectively, means some comparisons are made with caution – this mainly applies to the treatment interval and some patient characteristics.

For peer review only

For peer review only - http://bmjopen.bmj.com/site/about/guidelines.xhtml

Introduction

Lung cancer is the most common cancer worldwide, with nearly 1.83 million cases diagnosed in 2012, and is the leading cause of cancer death globally, accounting for 19% of cancer deaths.[1] Survival is typically low, with 5-year survival in Europe and North America <20%.[2] A key factor is diagnosis at advanced stage. Reasons for this are multi-faceted and include delays due to the atypical nature of some presenting symptoms, poor sensitivity of chest X-rays and primary care physicians not acting quickly enough.[3] Within European countries, differences of 12 and 5 percentage points in 1- and 5-year relative survival, respectively, have been reported for lung cancers diagnosed between 1999-2007.[4] This and other international comparisons raises the possibility of additional contributory factors such as variations in referral patterns, access to diagnostic tests and delays in treatment.[5]

One way of addressing this is to chart the patient journey from first noticing symptoms to treatment start. While many national studies using different methodologies have reported on time intervals to treatment in lung cancer, as far as we are aware no international comparisons exist.[6-18]

The International Cancer Benchmarking Partnership (ICBP) was established to explore differences in cancer outcomes and their causes in countries with comparable wealth and universal access to healthcare.[19] We report results from Module 4 (ICBP M4) on differences in time intervals and routes to diagnosis in symptomatic lung cancer patients from ten jurisdictions in Australia, Canada, Denmark, Norway, Sweden and the United Kingdom.

Methods

Methods have been previously detailed.[20] In brief, in each of the ten participating jurisdictions (Victoria (Australia), Manitoba, Ontario (Canada), Denmark, Norway, Sweden Northern Ireland, England, Scotland and Wales), consecutive patients aged $\geq$40 years, newly diagnosed with malignant lung or bronchus cancer (ICD-10:C34.0-C34.9; behaviour code ICD-O-3) were identified by the cancer registry using validated methods (hospital episode, cancer registration, pathology). Exclusion criteria included previous lung or synchronous cancers. Patients with a previous non-lung primary cancer were eligible. Target recruitment was 200 symptomatic patients per jurisdiction.

Following a vital status check, cancer registries posted the patient questionnaire (Supplementary File 1) either 1) to the relevant primary care physician (PCP) who then forwarded the pre-addressed envelope (Wales, England, Scotland) or 2) to the patient

For peer review only - http://bmjopen.bmj.com/site/about/guidelines.xhtml

directly or via the research team (remaining seven jurisdictions). In an attempt to decrease attrition and recall bias, the protocol initially specified that all patient questionnaires should be completed within 6 months of diagnosis. As there were administrative delays in cancer notification, this was extended to 9 months.

On receipt of a completed patient questionnaire, in all jurisdictions except Sweden, the relevant PCP and cancer treatment specialist (CTS) were sent questionnaires (Supplementary Files 2 and 3). Specialists provided information on diagnosis and start date of treatment. The latter was collected directly from registry records in Northern Ireland and clinical databases in Denmark. Manitoba did not provide specialist data. Date of diagnosis and stage was also collected where possible through cancer registries. Information on the types of treatment (surgery, chemotherapy, radiotherapy and other) were obtained from the patient survey.

Data handling

Data were recoded centrally to ensure that the same explicit rules were applied throughout. Patients in whom age, date of diagnosis or consent were missing were excluded from analyses. Rules were used to combine data from the different sources in a standardised way that ensured reproducibility and transparency (Supplementary File 4). The rules employed a ‘hierarchy’ principle in terms of the order in which different data sources were used, and included imputation rules based on the available data. The exact rule was guided by the measure in question – for example, patient interval was collected primarily from the patient questionnaire whereas primary care time-points from the PCP questionnaire. All the measures were further validated using algorithms for outliers and implausible measures (e.g. negative time intervals).

Routes to diagnosis and symptoms prompting physician visit

These were derived from patient and PCP responses. Symptoms were coded by two PCP authors (DW and PV) into ‘lung cancer specific’ or ‘other’ (Supplementary File 5, Table 1).

Time intervals

Time intervals were derived using the checklist for the Aarhus Statement.[21] The following time-points were used to calculate the corresponding time intervals (Figure 1):

- first noticing symptoms
- first presentation to health care
- first referral to secondary care

For peer review only - http://bmjopen.bmj.com/site/about/guidelines.xhtml

- diagnosis date
- start of curative or palliative treatment

FIGURE 1

All time-points were validated if there were obvious inconsistency and negative intervals were set to 0 days. All intervals were truncated at 365 days. Missing data were imputed based on specific rules to ensure that the direction of a possible misclassification bias was known.

Demographics

Health status was measured using the self-reported general health item from short form 36 health survey (SF36).[22] Comorbidity was defined as one of four patient or PCP-reported diseases (heart or lung disease, stroke or diabetes) and categorised into: 'none', 'medium' (one or two) or 'high' (three or four). Level of educational was categorised as 'low' (vocational school or lower) and 'high' (university). Stage data (tumour, node, metastasis - TNM - classification) was grouped as I, II, III, IV or missing.[23]

Statistical analysis

Patient characteristics across jurisdictions were compared using Kruskal-Wallis test and Pearson's chi-squared test. Quantile regression was used to estimate differences in intervals.[24] The 50th, 75th and 90th percentiles were compared, using Wales as the reference jurisdiction as it had the lowest lung cancer survival in ICBP Module 1 analysis.[5] Counting days, we used the 'qcount' procedure.[25, 26] Parameters were calculated with 1000 jittered samples. For all interval analyses, multivariable models controlled for differences in age, sex and comorbidity between jurisdictions. Significance level was set to $\leq$0.05 with 95% confidence intervals (95%CI) calculated where appropriate. Statistical analyses were carried out using STATA v14.

Sensitivity and validity analyses

All analyses were repeated using only (1) those who fulfilled the 6-month cut-off criteria for interval from diagnosis to questionnaire completion and (2) patient data. The effect of excluding patients for whom at least one interval was missing was investigated. Agreement between the different data sources (registry, patient, PCP (except in Sweden) and CTS (except in Sweden and Manitoba) was measured by Lin's concordance correlation coefficient (CCC).[27]

For peer review only - http://bmjopen.bmj.com/site/about/guidelines.xhtml

Patient involvement

The research questions for this survey drew on an extensive literature relating to diagnosis and treatment delays leading to negative patient experiences. While patient experience was not a primary outcome measure for this study, patients were given the opportunity to comment on their experience through questionnaire free-text response options (under separate analysis). Patients were involved in the piloting of study instruments to ascertain if recruitment and questionnaire content and dissemination strategies were appropriate, described elsewhere.[20] Each jurisdiction has committed to communicating the findings and local implications of this study to organisations representing their study participants.

For peer review only

Results

Of 14,583 lung cancer patients, diagnosed between October 2012 and March 2015 who were alive when identified, 70% (10,203/14,583) were contacted (Table 1). 2,631 (25.8% of contacted, 18% of eligible) completed the patient questionnaire at a median of 5 months (range: 0.1,9) after diagnosis (Table 2). The response rate of contacted patients varied from 11.1% (146/1318) in Norway to 61.8% (333/539) in Denmark. Responding patients were more likely to be aged 60-79 years with less advanced stage (Table 1) and alive one year post diagnosis (data not shown). Of the 2,631 responses, 2,143 (81.5%) were included in the analyses which equates to 14.7% (2,143/14,583) of eligible patients. The key reason respondents were excluded were local oversampling (43.9%; 214/488) for additional analyses (Table 1). In Victoria, the registry was only able to contact patients who had undergone surgery while the sample size in Norway was limited (n=88) due to delays in securing appropriate approvals.

For peer review only - http://bmjopen.bmj.com/site/about/guidelines.xhtml

Table 1: Cohort for all ten jurisdictions and overall

Jurisdiction	Patients approached via PCP						Patient approached directly by registries/research teams														Total	
	Wales		England		Scotland		N Ireland		Denmark		Manitoba		Ontario		Sweden		Norway		Victoria			
	n	(%)	n	(%)	n	(%)	n	(%)	n	(%)	n	(%)	n	(%)	n	(%)	n	(%)	n	(%)	n	(%)
**Eligible patients [a, b]**	**1,811**	**(100)**	**2,517**	**(100)**	**1,366**	**(100)**	**620**	**(100)**	**539**	**(100)**	**980**	**(100)**	**4,080**	**(100)**	**493**	**(100)**	**1,318**	**(100)**	**859**	**(100)**	**14,583**	**(100)**
**Packs sent to PCP [c, d]**	**1,811**	**(99.7)**	**1,759**	**(69.9)**	**1,137**	**(83.2)**															**4,707**	**(82.7)**
pack not forwarded by PCP	547	(30.1)	255	(14.5)	201	(17.7)															1,003	(21.3)
unsure if pack forwarded by PCP	531	(29.2)	559	(31.8)	234	(20.6)															1,324	(28.1)
**Patients contacted by PCP [c, d]**	**733**	**(40.4)**	**945**	**(53.7)**	**702**	**(61.7)**															**2,380**	**(50.6)**
**Patients approached directly [c]**							**614**	**(99.0)**	**539**	**(100)**	**745**	**(76.0)**	**3,687**	**(90.4)**	**493**	**(100)**	**1,200**	**(91)**	**545**	**(63.4)**	**7,823**	**(88)**
patient died							6	(1.0)	0	(0.0)	103	(13.8)	249	(6.8)	0	(0.0)	0	(0.0)	0	(0.0)	358	(4.6)
no address							0	(0.0)	0	(0.0)	9	(1.2)	255	(6.9)	0	(0.0)	0	(0.0)	0	(0.0)	264	(3.4)
Other							0	(0.0)	0	(0.0)	6	(0.8)	215	(5.8)	0	(0.0)	0	(0.0)	0	(0.0)	221	(2.8)
**Patient responses (% of eligible patients)[c]**	**223**	**(12.3)**	**261**	**(10.4)**	**235**	**(17.2)**	**226**	**(36.5)**	**333**	**(61.8)**	**205**	**(20.9)**	**572**	**(14.0)**	**217**	**(44)**	**146**	**(11.1)**	**213**	**(24.8)**	**2,631**	**(18)**
**Patient responses (% of contacted)[e]**	**223**	**(30.4)**	**261**	**(27.6)**	**235**	**(33.5)**	**226**	**(37.2)**	**333**	**(61.8)**	**205**	**(32.7)**	**572**	**(19.3)**	**217**	**(44)**	**146**	**(12.2)**	**213**	**(39.1)**	**2,631**	**(27.5)**
extra sample for local purpose	0	(0.0)	0	(0.0)	0	(0.0)	0	(0.0)	0	(0.0)	0	(0.0)	214	(37.4)	0	(0.0)	0	(0.0)	0	(0.0)	214	(8.1)
other	0	(0.0)	0	(0.0)	35	(14.9)	25	(11.1)	38	(11.4)	0	(0.0)	43	(7.5)	0	(0.0)	0	(0.0)	3	(1.4)	144	(5.5)
**Patient surveys submitted for analyses [f]**	**223**	**(100)**	**261**	**(100)**	**200**	**(85.1)**	**201**	**(88.9)**	**295**	**(88.6)**	**205**	**(100)**	**315**	**(55.1)**	**217**	**(100)**	**146**	**(100)**	**210**	**(98.6)**	**2,273**	**(86.4)**
excluded for analyses – total	12	(5.4)	9	(3.4)	2	(1.0)	1	(0.5)	10	(3.4)	3	(1.5)	27	(8.6)	6	(2.8)	58	(39.7)	2	(1.0)	130	(5.7)
- previous cancer	0	(0.0)	5	(1.9)	0	(0.0)	0	(0.0)	0	(0.0)	0	(0.0)	3	(1.0)	0	(0.0)	0	(0.0)	0	(0.0)	8	(0.4)
- unknown date of consent or diagnosis	0	(0.0)	0	(0.0)	0	(0.0)	0	(0.0)	5	(1.7)	0	(0.0)	1	(0.3)	6	(2.8)	4	(2.7)	0	(0.0)	16	(0.7)
- consent too late/too early	12	(5.4)	4	(1.5)	2	(1.0)	1	(0.5)	5	(1.7)	3	(1.5)	22	(7.0)	0	(0.0)	33	(22.6)	2	(1.0)	84	(3.7)
- other	0	(0.0)	0	(0.0)	0	(0.0)	0	(0.0)	0	(0.0)	0	(0.0)	1	(0.3)	0	(0.0)	21	(14.4)	0	(0.0	22	(1.0)
**Patients included in analyses [i] (% of forwarded surveys)**	**211**	**(94.6)**	**252**	**(96.6)**	**198**	**(99.0)**	**200**	**(99.5)**	**285**	**(96.6)**	**202**	**(98.5)**	**288**	**(91.4)**	**211**	**(97.2)**	**88**	**(60.3)**	**208**	**(99.0)**	**2,143**	**(94.3)[h]**
**PCP surveys [j] (% of analysed patients)**	**133**	**(63.0)**	**196**	**(77.8)**	**149**	**(75.3)**	**181**	**(90.5)**	**218**	**(76.5)**	**109**	**(54.0)**	**93**	**(32.5)**	**n/a [h]**		**27**	**(30.7)**	**105**	**(50.5)**	**1,211**	**(56.6)[l]**
**Specialist surveys [k] (% of analysed patients)**	**98**	**(46.4)**	**153**	**(60.7)**	**106**	**(53.5)**	**n/a [g]**		**149 [g]**	**(52.3)**	**n/a [h]**		**62**	**(21.7)**	**n/a [h]**		**20**	**(22.7)**	**55**	**(26.4)**	**643**	**(37.0)[m]**

[a]Eligible as per protocol: individual aged 40 years or more, with cancer of the lung or bronchus (ICD-10 code: C34.0-C34.9; behaviour code ICD-O 3) but no synchronous primary cancer or prior history of lung cancer, alive at identification who completed consent to participate within nine months of diagnosis. [b]In some jurisdictions some 'eligible' patients had pre-opted out from being contacted and a small number where PCP information was not available. [c]Percentages of eligible patients. [d]Maximum of potentially contacted patients. i.e. sum of packs forwarded by PCP and packs unsure if forwarded by PCP. [e]Percentages of patients contacted by PCP (see note d) for Wales, England and Scotland or percentages of patients contacted directly by a registry excl. non-accessible patients (all other jurisdictions). [f] Percentages of patient responses. [g] Data obtained from registries instead in N Ireland and Denmark. [h] Data not collected in this jurisdiction. [i] Denominator = total number of forwarded cases excl. patients not included in analytic sample in Ontario. [j] Denominator r = total number of analysed cases excl. patients from Sweden. [k] Denominator = total number of analysed cases excl. patients from Sweden. Manitoba & N Ireland.

For peer review only - http://bmjopen.bmj.com/site/about/guidelines.xhtml

Baseline characteristics

The characteristics are detailed in Table 2. The cohort was predominantly White (95%), median age 70 years (IQR 64,75) with 82% reporting 'low' levels of education. Norway provided stage data classified as local, regional and distant which could not be converted to TNM stage data.

Ontario was the only jurisdiction with more female (65%) than male respondents. While self-reported health differed significantly, with Welsh patients (9%) reporting twice as high 'poor health' rates than English or Swedish (4%) and eight-fold that of Manitoba (2%), there was no difference in self-reported comorbidity rates. Even after the exclusion of Victoria, there was significant variation in early Stage (I/II) disease which ranged from 25% (Wales) to 59% (Ontario) and surgical resection rates which varied from 27% (Wales) to 58% (Ontario) (Table 2).

For peer review only

Table 2. The characteristics of eligible patients by jurisdictions and overall

	Wales (N=211)	England (N=252)	Scotland (N=198)	N Ireland (N=200)	Denmark (N=285)	Manitoba (N=202)	Ontario (N=288)	Sweden (N=211)	Norway (N=88)	Victoria (N=208)	Overall (N=2143)	p-value[6]
Date of diagnosis of first patient	03/04/2013	28/01/2013	26/04/2013	22/01/2013	16/05/2013	10/10/2012	08/10/2013	01/10/2013	16/01/2014	08/01/2013	10/10/2012	
Date of diagnosis of last patient	25/09/2014	14/08/2013	04/12/2013	02/12/2014	13/11/2013	27/03/2015	01/10/2014	30/05/2014	21/01/2015	28/12/2014	27/03/2015	
Interval from diagnosis date of first patient to last patient in months (recruitment period)	18	7	7	23	6	30	12	8	12	24	30	
Median (range) interval diagnosis to questionnaire completion in months	5 (0.6,9)	5 (3,9)	5 (1,9)	4 (0.1,9)	5 (2,8)	6 (4,9)	6 (4,9)	4 (3,8)	7 (5,9)	5 (0.2,8)	5 (0.1,9)	
**Age** years Median (IQR)	71 (65, 77)	71 (65, 77)	70 (64, 76)	69 (62, 75)	70 (63, 74)	70 (63, 77)	70 (64, 75)	70 (63, 75)	69 (64, 73)	68 (63, 73)	70 (64, 75)	0.010[1]
**Sex** n(%) Male	127(60)	134(53)	100(51)	105(53)	151(53)	100(50)	131(45)	111(53)	46(52)	112(54)	1117(52)	0.159[2]
**Health State** n(%) Good	135(64)	176(70)	131(66)	126(63)	190(67)	156(77)	220(76)	152(72)	50(57)	163(78)	1499(70)	<0.001*[1] <0.001**[2]
Fair	55(26)	59(23)	52(26)	51(26)	62(22)	41(20)	47(16)	49(23)	32(36)	33(16)	481(22)	
Poor	20(9)	11(4)	14(7)	16(8)	18(6)	4(2)	16(6)	9(4)	6(7)	10(5)	124(6)	
Missing	1(0.5)	6(2)	1(0,5)	7(4)	15(5)	1(0.5)	5(2)	1(0.5)	0(0)	2(1)	39(2)	
**Comorbidity**[3] n(%) No	92(44)	113(45)	98(49)	82(41)	111(39)	101(50)	136(47)	114(54)	35(40)	98(47)	980(46)	0.029*[1] 0.032**[2]
Medium	111(53)	129(51)	92(46)	103(52)	157(55)	93(46)	132(46)	87(41)	48(55)	100(48)	1052(49)	
High	7(3)	9(4)	8(4)	15(8)	11(4)	6(3)	18(6)	6(3)	5(6)	8(4)	93(4)	
Missing	1(0.5)	1(0.4)	0(0)	0(0)	6(2)	2(1)	2(0.7)	4(2)	0(0)	2(1)	18(0.8)	
**Education** n(%) Low	172(82)	217(86)	174(88)	166(83)	232(81)	170(84)	224(78)	159(75)	65(74)	181(87)	1760(82)	<0.001*[1] <0.001**[2]
High	11(5)	15(6)	11(6)	17(9)	15(5)	21(10)	55(19)	48(23)	14(16)	24(12)	231(11)	
Missing	28(13)	20(8)	13(7)	17(9)	38(13)	11(5)	9(3)	4(2)	9(10)	3(1)	152(7)	
**Ethnicity** n(%)	205(97)	249(99)	197(99)	196(98)	267(94)	173(86)	261(91)	n/a	87(99)	199(96)	1834(95)	

For peer review only

For peer review only - http://bmjopen.bmj.com/site/about/guidelines.xhtml

	Wales (N=211)	England (N=252)	Scotland (N=198)	N Ireland (N=200)	Denmark (N=285)	Manitoba (N=202)	Ontario (N=288)	Sweden (N=211)	Norway (N=88)	Victoria (N=208)	Overall (N=2143)	p-value[6]
White												<0.001*[2] <0.001**[2]
Asian	1(0.5)	0(0)	1(0.5)	0(0)	0(0)	12(6)	21(7)	n/a	1(1)	6(3)	41(2)	
Black	0(0)	1(0.4)	0(0)	0(0)	1(0.4)	0(0)	1(0.3)	n/a	0(0)	0(0)	3(0.2)	
Other	0(0)	0(0)	0(0)	0(0)	0(0)	15(7)	2(0.7)	n/a	0(0)	0(0)	17(0.9)	
Missing	5(2)	2(0.8)	0(0)	4(2)	17(6)	2(1)	3(1)	n/a	0(0)	3(1)	36(2)	
**Smoking** n(%) Never	13(6)	18(7)	12(6)	19(10)	15(5)	22(11)	31(11)	41(19)	11(13)	33(16)	215(10)	<0.001*[2] <0.001**[2]
Currently	19(9)	28811)	26(13)	41(21)	58(20)	24(12)	29(10)	29(14)	11(13)	9(4)	274(13)	
In the past	174(82)	204(81)	160(81)	137(69)	205(72)	156(77)	225(78)	139(66)	66(75)	165(79)	1631(76)	
Missing	5(2)	2(0.8)	0(0)	3(2)	7(2)	0(0)	3(1)	2(0.9)	0(0)	1(0.5)	23(1)	
**Tumour stage – TNM** n(%) I	26(12)	68(27)	49(25)	42(21)	74(26)	83(41)	133(46)	59(28)	3(3)	124(60)	661(31)	<0.001*[2] <0.001**[2]
II	27(13)	36(14)	32(16)	33(17)	26(9)	19(9)	38(13)	11(5)	5(6)	47(23)	274(13)	
III	64(30)	57(23)	56(28)	59(30)	84(29)	48(24)	54(19)	40(19)	5(6)	19(9)	486(23)	
IV	62(29)	80(32)	50(25)	62(31)	94(33)	48(24)	54(19)	94(45)	3(3)	13(6)	560(26)	
Missing	32(15)	11(4)	11(6)	4(2)	7(2)	4(2)	9(3)	7(3)	72(82)[4]	5(2)	162(8)	
**Tumour stage – TNM**[5] n(%) I/II	53(25)	104(41)	81(41)	75(38)	100(35)	102(51)	171(59)	70(33)	8(9)	*171(82)*	764(39)	<0.001*[2,5] <0.001**[2,5]
III/IV	126(60)	137(54)	106(54)	121(61)	178(62)	96(48)	108(38)	134(64)	8(9)	32(15)	1014(52)	
Missing	32(15)	11(4)	11(6)	4(2)	7(2)	4(2)	9(3)	7(3)	72(82)	5(2)	157(8)	
**Treatment Surgery** n(%) Yes	57(27)	107(42)	84(42)	65(33)	81(28)	113(56)	168(58)	65(31)	36(41)	199(96)	975(45)	<0.001*[2,5] <0.001**[2,5]
No	61(29)	56(22)	55(28)	94(47)	90(32)	44(22)	111(39)	79(37)	28(32)	5(2)	623(29)	
Missing	93(44)	89(35)	59(30)	41(21)	114(40)	45(22)	9(3)	67(32)	24(27)	4(2)	545(25)	
**Treatment Chemo** n(%) Yes	105(50)	125(50)	98(49)	93(47)	159(56)	93(46)	107(37)	133(63)	50(57)	63(30)	1026(48)	<0.001*[2,5] <0.001**[2,5]
No	41(19)	51(20)	46(23)	65(33)	45(16)	62(31)	172(60)	37(18)	18(20)	137(66)	674(32)	
Missing	65(31)	76(30)	54(27)	42(21)	81(28)	47(23)	9(3)	41(19)	20(23)	8(4)	443(21)	
**Treatment Radio** n(%) Yes	82(39)	68(27)	76(38)	72(36)	114(40)	81(40)	98(34)	70(33)	41(47)	29(14)	731(34)	<0.001*[2,5] <0.001**[2,5]
No	50(24)	72(29)	50(25)	77(39)	69(24)	71(35)	180(63)	69(33)	22(25)	155(75)	815(38)	
Missing	79(37)	112(44)	72(36)	51(26)	102(36)	50(25)	10(3)	72(34)	25(28)	24(12)	597(28)	
**Treatment Other** n(%) Yes	10(5)	14(6)	14(7)	11(6)	30(11)	18(9)	9(3)	0(0)	7(8)	16(8)	129(6)	<0.001*[2,5] <0.001**[2,5]
No	48(23)	63(25)	45(23)	69(35)	255(89)	3(1)	261(91)	0(0)	2(2)	138(66)	884(41)	
Missing	153(72)	175(69)	139(70)	120(60)	0(0)	181(90)	18(6)	211(100)	79(90)	54(26)	1130(53)	

For peer review only - http://bmjopen.bmj.com/site/about/guidelines.xhtml

[1]Differences between jurisdictions were tested by the Kruskal-Wallis test, [2] Differences between jurisdictions were tested by the Pearson's Chi^2 test, [3]Comorbidity coded as none=no reported, medium=1-2 reported and high=3+ reported, [4] This included cases which could not be mapped as they were classified as per Cancer Registry of Norway into local (stage I), regional (stage II-III) and distant (stage IV) [5]Excluding Victoria, [6]Excluding Norway, *Missing category is excluded, **Missing category is included,
Abbreviations: IQR=inter-quartile range.

For peer review only

For peer review only - http://bmjopen.bmj.com/site/about/guidelines.xhtml

Routes to diagnosis

Results are detailed in Table 3. Over half (55%) were diagnosed following presentation to the PCP of whom 63% (range: 29% Norway - 82% Wales; data not shown) were urgently referred with a suspicion of cancer, based on the PCP questionnaire.

For peer review only

For peer review only - http://bmjopen.bmj.com/site/about/guidelines.xhtml

Table 3. Routes to diagnosis of lung cancer patients for each jurisdiction. All figures are n(%) unless otherwise stated.

	Wales (N=211)	England (N=252)	Scotland (N=198)	N Ireland (N=200)	Denmark (N=285)	Manitoba (N=202)	Ontario (N=288)	Sweden (N=211)	Norway (N=88)	Victoria (N=208)	Total (N=2143)
Symptoms prompting visit to PCP	109(52)	150(60)	128(65)	131(66)	170(60)	101(50)	91(32)	65(31)	35(40)	106(51)	1086(51)
Symptoms prompting emergency (A&E) department visit[1]	11(5)	18(7)	12(6)	22(11)	21(7)	25(12)	39(14)	18(9)	3(3)	6(3)	175(8)
Symptoms prompting visit to PCP and emergency (A&E) department[1]	10(5)	4(2)	7(4)	18(9)	8(3)	15(7)	12(4)	8(4)	5(6)	3(1)	90(4)
Incidental diagnosis in course of investigation/treatment for another problem[2]	68(32)	37(15)	36(18)	19(10)	55(19)	57(28)	116(40)	107(51)	32(36)	90(43)	617(29)
Unknown routes to diagnosis[3]	10(5)	17(7)	11(6)	5(3)	16(6)	4(2)	14(5)	11(5)	8(9)	0(0)	96(5)
Other[4]	3(1)	25(10)	3(2)	4(2)	14(5)	0(0)	16(6)	2(1)	5(6)	3(1)	75(3)
Missing	0(0)	1(0.4)	1(0.5)	1(0.5)	1(0.4)	0(0)	0(0)	0(0)	0(0)	0(0)	4(0.2)

[1] Emergency (A&E) route was ascribed when the patient reported pathway to cancer diagnosis involving going or being taken to the emergency department or the PCP reported that the patient presented to emergency department with or without their involvement [2] this could be by PCP, another doctor or via hospital; [3]includes cases where PCP or patient reported routes to diagnosis as 'Other' or 'Missing' but also reported symptoms or duration of symptoms, or date of first symptom, or waiting time for PCP appointment; [4]includes cases where PCP or patient reported routes to diagnosis as 'Other' and hasn't reported any symptoms or duration of symptoms, or date of first symptom, or waiting time for PCP appointment.

For peer review only - http://bmjopen.bmj.com/site/about/guidelines.xhtml

Symptoms prompting visit to physician

The median number of patient-reported symptoms were 2 (IQR 1,3). Across jurisdictions, the most common patient-reported symptoms were persistent cough (39%), breathlessness (37%) and fatigue (27%) although there was significant variation in proportion of patients presenting with individual symptoms (Table 4).

The PCPs reported a median of 1 (IQR 1,2) symptom at first presentation, with the most common being persistent cough (39%). Across jurisdictions, the reporting of other symptoms by the PCP was significantly lower compared to patients, especially fatigue (4%) and weight loss (8%). Unlike patients, there was minimal variation in PCP reporting of symptoms, with significant differences limited to 'no symptoms', 'other symptoms not previously listed' and weight loss. Overall 64% of symptoms were labelled as 'cancer specific' by the study PCPs (Table 4).

For peer review only

For peer review only - http://bmjopen.bmj.com/site/about/guidelines.xhtml

Table 4. Symptoms experienced by patients and presenting symptoms noted by PCP for eligible patients. All figures are n(%)

	Wales	England	Scotland	N Ireland	Denmark	Manitoba	Ontario	Sweden	Norway	Victoria	Overall	p[1]
**Symptoms (reported by patient)**	(N=211)	(N=252)	(N=198)	(N=200)	(N=285)	(N=202)	(N=288)	(N=211)	(N=88)	(N=208)	(N=2143)	
persistent cough	113(54)	123(49)	97(49)	83(42)	97(34)	71(35)	125(43)	84(40)	10(11)	39(19)	842(39)	<0.001
breathlessness	109(52)	126(50)	82(41)	73(37)	99(35)	63(31)	119(41)	77(36)	12(14)	24(12)	784(37)	<0.001
fatigue	75(36)	79(31)	64(32)	61(31)	38(13)	55(27)	92(32)	60(28)	17(19)	42(20)	583(27)	<0.001
weight loss	38(18)	39(15)	44(22)	37(19)	41(14)	29(14)	36(13)	34(16)	9(10)	20(10)	327(15)	0.147
felt sick /vomiting /nausea/loss of appetite	42(20)	33(13)	31(16)	24(12)	33(12)	22(11)	45(16)	28(13)	3(3)	20(10)	281(13)	0.132
coughing up blood-stained phlegm (sputum)	35(17)	32(13)	24(12)	31(16)	21(7)	16(8)	27(9)	25(12)	5(6)	23(11)	239(11)	0.014
chest or shoulder pain	23(11)	9(4)	18(9)	24(12)	10(4)	28(14)	43(15)	25(12)	4(5)	22(11)	206(10)	<0.001
other symptoms not listed above	51(24)	80(32)	59(30)	54(27)	63(22)	40(20)	30(10)	75(36)	26(30)	42(20)	520(24)	<0.001
no symptoms	29(14)	24(10)	32(16)	21(11)	63(22)	50(25)	67(23)	36(17)	33(38)	75(36)	430(20)	<0.001
missing	1(0.5)	16(6)	6(3)	17(9)	31(11)	9(4)	4(1)	4(2)	12(14)	1(0.5)	101(5)	<0.001
**Presenting symptom (reported by PCP)**	(N=85)	(N=133)	(N=115)	(N=0)	(N=151)	(N=86)	(N=61)	(N=0)	(N=17)	(N=81)	(N=729)	
persistent cough	33(39)	57(43)	45(39)	n/a	67(44)	24(28)	18(30)	n/a	7(41)	33(41)	284(39)	0.093
breathlessness	17(20)	27(20)	20(17)	n/a	27(18)	11(13)	11(18)	n/a	4(24)	13(16)	130(18)	0.803
fatigue	1(1)	5(4)	3(3)	n/a	9(6)	2(2)	2(3)	n/a	1(6)	3(4)	26(4)	0.449
weight loss	5(6)	9(7)	15(13)	n/a	19(13)	3(3)	2(3)	n/a	0(0)	5(6)	58(8)	0.027
felt sick /vomiting /nausea/loss of appetite	1(1)	1(0.8)	2(2)	n/a	6(4)	2(2)	0(0)	n/a	0(0)	0(0)	12(2)	0.312
coughing up blood-stained phlegm (sputum)	8(9)	10(8)	10(9)	n/a	7(5)	2(2)	3(5)	n/a	0(0)	7(9)	47(6)	0.305
chest or shoulder pain	8(9)	10(8)	15(13)	n/a	15(10)	1(1)	7(12)	n/a	1(6)	4(5)	61(8)	0.08
other symptoms not listed above	24(28)	44(33)	33(29)	n/a	64(42)	15(17)	7(12)	n/a	7(41)	20(25)	214(29)	<0.001
no symptoms	5(6)	6(5)	6(5)	n/a	2(1)	31(36)	12(20)	n/a	0(0)	10(12)	72(10)	<0.001
missing	7(8)	7(5)	11(10)	n/a	16(11)	12(14)	6(10)	n/a	2(12)	10(12)	71(10)	0.392

For peer review only

For peer review only - http://bmjopen.bmj.com/site/about/guidelines.xhtml

**Cancer-specificity of symptom presented**												
cancer-specific symptom	62(73)	97(73)	79(69)	n/a	94(62)	36(42)	38(62)	n/a	10(59)	48(59)	464(64)	<0.001
non-specific symptom	11(13)	23(17)	19(17)	n/a	39(26)	7(8)	5(8)	n/a	5(29)	13(16)	122(17)	
no symptoms /missing	12(14)	13(10)	17(15)	n/a	18(12)	43(50)	18(30)	n/a	2(12)	20(25)	143(19)	

[1] Differences between jurisdictions (excluding Victoria and Norway) were tested by the Pearson's Chi^2 test

For peer review only

Time intervals

The observed time intervals are shown in Supplementary File 5, Table 2 and are summarised in Figure 2. Based on overall trends, jurisdictions could be grouped into those with reduced, longer and similar intervals to Wales (Table 5). It was not possible to interpret variations observed in Norway (small sample size) and Victoria (surgical cohort). In the remaining jurisdictions, there was no difference in the median adjusted patient interval compared to Wales. Denmark had shorter median adjusted primary care interval (-11 days); Sweden had shorter (-20) and Manitoba longer (+40) diagnostic intervals compared to Wales. Denmark (-13), Manitoba (-11), England (-9) and Northern Ireland (-4) had shorter treatment intervals. The median adjusted total interval was shorter only in Denmark (Table 5, Figure 2). The differences were greater for the 90th percentile. The total interval in patients who waited longest (90th percentile) was significantly shorter in two jurisdictions (Denmark -142, England -28 days) compared to Wales.

For peer review only - http://bmjopen.bmj.com/site/about/guidelines.xhtml

Table 5. Differences in adjusted intervals (days) between Wales and the other nine jurisdictions for lung cancer patients

Intervals	percentiles	Wales	Denmark	Sweden	England	N Ireland	Scotland	Manitoba	Ontario	Norway	Victoria
		Reference in days	Overall trend - shorter intervals			Similar with some intervals longer, some shorter		Overal trend - longer intervals		Difficult to interpret (see text for reasons)	
Ranking by 5-year survival rates for lung cancers diagnosed in 1999-2007 [5]		10	6	1	9	7	8	3	2	5	4
Patient Interval	Number of patients	181	233	172	213	179	169	133	205	55	141
	50th percentile (95% CI)	21	-6 (-13,0)	-1 (-9,8)	-3 (-10,5)	-3 (-13,6)	-3 (-10,4)	1 (-8,10)	1 (-11,14)	0 (-8,8)	**-9** (-16,-2)
	75th percentile (95% CI)	61	-13 (-38,13)	-2 (-24,21)	-2 (-42,37)	-8 (-55,39)	1 (-25,27)	3 (-23,30)	-7 (-54,39)	-9 (-60,42)	-4 (-46,38)
	90th percentile (95% CI)	216	**-34** (-55,-13)	-1 (-25,23)	-15 (-42,12)	24 (-21,70)	**43** (7,79)	**-35** (-59,-10)	**-34** (-66,-2)	**59** (21,96)	**-35** (-49,-21)
Primary Care interval	Number of patients	110	159	N/A	147	124	119	80	75	19	89
	50th percentile (95% CI)	20	**-11** (-18,-3)		-7 (-17,3)	-5 (-15,4)	-3 (-14,8)	7 (-8,21)	5 (-9,19)	**-11** (-18,-4)	-8 (-17,1)
	75th percentile (95% CI)	43	**-29** (-47,-12)	N/A	-17 (-42,8)	1 (-45,48)	-11 (-36,14)	19 (-47,85)	20 (-72,112)	-10 (-57,37)	-12 (-70,46)
	90th percentile (95% CI)	91	-30 (-66,7)		-39 (-85,6)	17 (-55,90)	-20 (-67,25)	13 (-38,65)	102 (-56,258)	-22 (-109,66)	-19 (-89,51)
Diagnostic interval	Number of patients	176	229	165	212	170	173	138	212	52	160
	50th percentile (95% CI)	45	-12 (-25,1)	**-20** (-35,-5)	9 (-3,21)	17 (-5,39)	-4 (-16,8)	**40** (14,66)	10 (-6,26)	4 (-16,24)	7 (-13,27)
	75th percentile (95% CI)	108	**-45** (-52,-39)	**-22** (-30,-15)	-7 (-20,7)	**12** (2,22)	-15 (-32,1)	**35** (22,48)	5 (-9,19)	-4 (-15,8)	-2 (-8,4)
	90th percentile (95% CI)	162	-27 (-153,99)	-34 (-206,138)	-14 (-100,72)	112 (-165,389)	31 (-81,143)	**112** (32,192)	106 (-122,335)	0 (-93,93)	**62** (10,114)
Treatment interval	Number of patients	192	279	190	238	200	187	182	263	87	199
	50th percentile (95% CI)	43	**-13** (-15,-11)	-2 (-8,3)	**-9** (-12,-5)	**-4** (-7,-2)	0 (-4,4)	**-11** (-17,-5)	3 (-4,10)	**-8** (-11,-6)	**-29** (-32,-27)
	75th percentile (95% CI)	64	**-32** (-36,-28)	-2 (-8,4)	**-18** (-23,-13)	**-13** (-18,-7)	1 (-7,9)	-5 (-16,6)	6 (-2,14)	**-13** (-19,-8)	**-33** (-41,-25)
	90th percentile (95% CI)	89	**-45** (-50,-40)	**-10** (-17,-4)	**-28** (-36,-20)	**-16** (-23,-9)	-6 (-14,1)	4 (-5,13)	4 (-4,13)	**-22** (-30,-14)	**-39** (-45,-32)
Total interval	Number of patients	147	192	147	176	153	143	117	178	52	113
	50th percentile (95% CI)	116	**-49** (-95,-3)	-8 (-64,47)	-7 (-52,38)	-16 (-41,10)	-2 (-70,66)	11 (-41,63)	9 (-78,97)	**-34** (-56,-12)	-32 (-64,2)
	75th percentile (95% CI)	204	-91 (-270,87)	-17 (-40,7)	-29 (-175,118)	5 (-191,201)	33 (-144,211)	13 (-77,103)	-7 (-331,317)	-39 (-107,29)	-23 (-61,14)
	90th percentile (95% CI)	365	**-142** (-150,-134)	-18 (-59,23)	**-28** (-37,-18)	0 (-4,5)	15 (-26,55)	15 (-26,55)	0 (-78,79)	**-84** (-119,-49)	0 (-3,3)

Interval relative to Wales	Trends	Significant
Reduction		
Increase		

The majority of patients were diagnosed between 2013-14. The differences were calculated for the 50th, 75th and 90th percentiles by setting age to its mean value and gender and comorbidity to their modes (i.e. male gender and medium comorbidity). It is not possible to interpret differences observed for Norway (due to the small sample size) and Victoria (the cohort was limited to those who had undergone surgery).

For peer review only - http://bmjopen.bmj.com/site/about/guidelines.xhtml

FIGURE 2

Sensitivity and validity analyses

The estimates of routes to diagnosis, time intervals, and regression analysis trends were not significantly altered by changing the cut-off to 6 months or using only patient data (results not shown).Comparing the dates between the different data sources showed adequate agreement between all data sources for all categories of dates (CCC = 0.94 for date of treatment, CCC $\geq$ 0.93 for date of diagnosis, CCC = 0.91 for date of first presentation to primary care).

For peer review only

For peer review only - http://bmjopen.bmj.com/site/about/guidelines.xhtml

Discussion

Main findings

This is the first international study we are aware of comparing lung cancer routes and time intervals. With the exception of Denmark, in all other jurisdictions, the median total interval from symptom onset to treatment, for respondents diagnosed in 2012-15 was similar to that of Wales, the reference. However, there were jurisdiction specific differences in patient, diagnostic and treatment intervals, especially for the 10% of patients who waited the longest. Based on overall trends, jurisdictions could be grouped into those with trends of reduced, longer and similar intervals to Wales. Across jurisdictions, all symptoms other than persistent cough were less frequently reported by the PCP when compared to patients. This was especially true for fatigue and weight loss. One in four patients reported incidental diagnosis and one in ten were diagnosed following a visit to the emergency (A&E) department.

Strengths and weaknesses

Strengths of this study include 1) its international setting; 2) use of cancer registries to identify consecutive newly diagnosed patients; 3) use of standardised questionnaires; 4) inclusion of PCP and CTS questionnaires enriched by registry data; 5) minimal data interpretation by the local teams with all data cleaning performed in a standardised manner centrally; and 6) triangulation with comprehensive data rules to ensure validity, consistency and preserve statistical precision.[20] Recall bias was minimised by the triangulation of different data sources and by patients completing the questionnaire within a limited time window (median 5 months) after the cancer diagnosis.

A key limitation, as with all questionnaire-based studies was non-response bias which varied across jurisdictions. In comparing intervals, we adjusted for age, sex and comorbidity but were unable to adjust for ethnicity and education due to different classification systems. Recruitment of patients up to 9 rather than 6 months after diagnosis might have magnified the selection bias due to high mortality.[28] However, sensitivity analysis suggests that this did not impact on the results. Categorising presenting symptoms into indicative or not was done pragmatically as existing guidelines for lung cancer investigation vary across ICBP jurisdictions.[29] In Norway and Victoria, a small sample size and restriction of eligibility to only surgical patients, respectively, made comparison difficult. Nonetheless, significant differences in these two jurisdictions compared to Wales were largely limited to the treatment interval alone, suggesting that despite sampling issues, the pathway to diagnosis was comparable.

For peer review only - http://bmjopen.bmj.com/site/about/guidelines.xhtml

There was variation in stage distribution across jurisdictions. While this may be partly related to the varying response rate, true differences in lung cancer stage have been noted on analysis of registry data of patients diagnosed between 2004-2007.[5] The high lung cancer mortality and self-selection are likely to have contributed to an over-representation of early stage disease and tumours treated with surgical resection. This suggests that true variation may well be higher than that reported in this cohort of 'healthier early stage' patients.

Comparison to other studies

The most common patient-reported symptoms, in keeping with the literature, were persistent cough, breathlessness, fatigue and weight loss, with one in five reporting 'no symptoms'.[13] Only a minority (11%) of our respondents reported coughing blood or bloody sputum/spit, which is the only consistent predictor of lung cancer.[30] While haemoptysis was reported in a prospective survey (England 2011-12) by 22% of lung cancer patients identified through respiratory clinics, it was a presenting symptom in only 5% of cases.[6]

As lung cancer mortality is higher in patients attending emergency (A&E) departments, the rates are often compared in an attempt to understand international survival differences.[31] The rate of respondents who attended A&E varied two-fold across jurisdictions from 9-10% in England, Scotland and Denmark to 18-20% in Northern Ireland, Ontario and Manitoba. While rates for Scotland (10%) were similar to that reported in a prospective Scottish audit (11.5%), as were rates for Denmark (7% vs 6.3% when PCP not involved), rates for England (9%) were lower than those reported in population based audits (25%) reflecting non-response bias.[9,10] In Victoria (4%) restriction of the cohort to surgical patients is likely to have accounted for the very low rates.

Our reported median patient, primary care and diagnostic intervals are in keeping with those previously reported from the participating jurisdictions (Table 6). Minor variations in interval estimates are likely due to differences in data source, sample size and cohort characteristics.[32] Longer intervals were reported from earlier cancer cohorts - median primary care interval for England of 52 days in 1998-2000 (our median 11),[8] median total interval for Denmark of 108 days in 2004-5 (our median 67) and Norway of 118 in 2002-5 (our median 79).[12-14]

For peer review only - http://bmjopen.bmj.com/site/about/guidelines.xhtml

Table 6. Summary of literature on intervals in lung cancer patients diagnosed since 2000 in the ICBP Module 4 countries

Study No	Ref	Study Period	Jurisdiction	Design	Patients	No. of lung cancer patients	Interval[1] (days)				Total interval
							Patient	Primary care	Diagnostic	Treatment	
1	Walter FM et al (2015)	2011-2	England, UK	Prospective patient questionnaire survey - multi hospital cohort. Dates of diagnosis based on medical note review.	All attending urgent and routine respiratory clinics across the five hospitals in England aged over 40 years with symptoms suspicious of lung cancer.	153	Interval from first symptom to diagnosis Median 91 (IQR 49-184)				
2	Lyratzopoulos G et al (2015)	2009-10	England, UK	Prospective national audit of cancer diagnosis using primary practice patient records and continuous sampling during audit period	All patients aged ≥15 years who had first presented to a primary care practitioner and were subsequently diagnosed with one of 28 cancers	1128	Median 11 (IQR 0-32)	Median 3 (IQR 14-39)			
3	Neal R et al (2014)	2007–8	UK	Retrospective analysis of electonic health record data from General Practice Research Database - population cohort	All newly diagnosed with one of 15 cancers	2851			Median 112 (IQR 45-251)		
4	Barrett J et al (2008)	1998-2002	Exeter, England, UK	Retrospective case-control review of PCP records - population cohort	All with lung cancer aged ≥ 40 years, identified from the hospital cancer registry and computerised searches of all primary care practices	247		Median 52 (IQR 7–243)			
							Interval from first symptom to diagnosis - Median 121 (IQR 53,261)				
5	Baughan P et al (2009)	2005-6, 2007-8	Scotland, UK	Retrospective audit involving PCP review of medical records of all newly diagnosed cancer patients they had seen - population cohort	All newly diagnosed with lung cancer	981	Median 9.5 (IQR 31)		Median 11 (IQR 28)		
6	Guldbrandt LM et al (2015)	2010	Denmark	Retrospective PCP questionnaires survery of national registry-based population cohort	All consecutive newly diagnosed lung cancer patients	429-442 depending on interval		Median 7 (IQR, 0-30)	Median 29 (IQR, 12-69)		
7	Tørring ML et al (2013)	2004 - 5	Aarhus, Denmark	Prospective, population-based study using electronic health records and PCP survey of identified patients	All newly diagnosed with lung cancer after attending primary care	262			Median 112 (IQR 45-251)		
8	Hansen RP et al (2011)	2004-5	Aarhus, Denmark	Retrospective PCP survey	Cancer patients newly diagnosed during a 1-year period identified using administrative registry data	128-251 (depending on interval)	Median 28 (IQR 7-56)	Median 0 (IQR 0-9)		Median 51 (IQR 27-76)	Median 108 (IQR 82-167)
9	Bjerager M et al (2006)	2003	Aarhus, Denmark	Retrospective PCP survey using structured telephone interviews enriched with administrative registry data - population-based cohort	All lung cancer patients identified through histological and cytological tests from county-based registers	84		Median 32.5 (IQR 12–68)			
10	Rolke HB et al (2006)	2002-5	Norway (South)	Retrospective questionnaire-based patient survey - hospital cohort	All newly diagnosed with lung cancer	273 -376 (depending on interval)	Median 19 (2–77)				Median 118 (IQR 68–220)
11	Stokstad T et al (2017)	2011-13	Norway	Retrospective medical record audit - single hospital cohort	All cases that started diagnostic work-up and were diagnosed with lung cancer at St. Olavs Hospital, Trondheim	449				42 days (range: 2–296)	
12	Largey G et al (2016)	2013	Victoria, Australia	Retrospective medical record audit - three hospital cohort	Admitted with a new diagnosis of lung cancer over a three month period in three hospitals	78				Mean 30.4 (SD 45.3)	
13	Evans SM et al (2016)	2011-4	Victoria, Australia	Retrospective medical record audit - multi hospital cohort	All lung cancer patients newly diagnosed in six public and two private hospitals	1417			Median 15 (IQR, 5–36)	Median 30 (IQR, 6–84)	
14	Emery JD et	2012 -	Western	Prospective cluster randomised trial of	Lung cancer patients newly diagnosed in	167	Interval from first symptom to				

For peer review only - http://bmjopen.bmj.com/site/about/guidelines.xhtml

	al (2017)	4	rural Australia	symptom awareness	the control arm of the trial		diagnosis Median 34.5 (IQR 7 103.5)				
15	Burneister BH et al (2010)	2000-4	Queensland Australia	Retrospective analysis of radiation therapy waiting times	All lung cancer patients who received radiation therapy as initial treatment at a public hospital.	1535					Median 33[2]
16	Ellis PM and Vandermeer R (2011)	2010	Ontario, Canada	Retrospective patient survey using structured telephone interviews - single centre cohort. Appointment dates and diagnostic tests verified through family doctor or patient chart review.	All lung cancer patients referred to a regional cancer centre	52		Median 21	Median 27 (IQR 0-38)		Median 138 (IQR 79-175)
17	Lo DS et al (2007)	2005-7	Ontario, Canada	Retrospective medical record audit - multi hospital cohort	All with lung cancer seen on a newly implemented Time to Treat Program	144			Median interval from suspicion of lung cancer to diagnosis 37		

[1] Intervals as defined in Figure 1; [2] Limited to patients receiving radiation treatment

For peer review only - http://bmjopen.bmj.com/site/about/guidelines.xhtml

Across all jurisdictions, there was no significant difference in primary care intervals for the 10% of patients with longest interval. It is likely that these patients had vague or non-specific symptoms and signs. Referral guidelines for suspected lung cancer do not always favour patients with early symptoms and often prioritise those with more advanced disease.[33] Access to better diagnostic tools such as low-dose CT chest in the primary care setting may favour this group of patients.[34] It would be useful in future projects to explore whether such access may have contributed to the improved 1-year lung cancer survival rates reported from Australia and Canada.[5]

Diagnostic intervals were significantly longer for Manitoba. Of note, in 51% of the PCP responses presenting symptoms were missing or recorded as not present and date of first presentation was derived from patient as opposed to PCP. While this may explain why the diagnostic interval was twice that reported from ongoing local audit (personal communication), for date of first presentation, the concordance co-efficient between PCP and patients at Manitoba was 0.94, which suggest adequate agreement.

Observed median treatment intervals were below 6 weeks for nearly all jurisdictions. This was the only interval where there were significant differences between jurisdictions with Denmark, England, Norway and Northern Ireland all having shorter adjusted treatment intervals across all percentiles, with larger differences for the 75th and 90th percentile. These improvements may reflect implementation of waiting time targets in Denmark (35-38 days from first consultation depending on treatment modality) and the UK (31 days from decision to treat).[35,36] The shorter treatment intervals in Norway are in keeping with long-standing provision of standardised cancer care pathways and effective coordination between primary care and treatment centers. While a systematic review did not find evidence to support an association between intervals and lung cancer outcomes, increasing mortality with longer diagnostic intervals was noted in a more recent, high-quality study.[11] In 2000, O'Rourke reported median delays of 94 days (35-187) between the first hospital visit and starting treatment resulting in 21% of potentially curable patients becoming incurable.[37] Others have found metabolic evidence on PET/CT of pre-treatment disease progression in 21% and TNM upstaging in 18% of small-cell lung cancer patients after a relatively short median inter-scan interval of 43 days.[38] Delays can also result in deterioration in performance status. More recently, there is concern that the use of genotyping prior to starting treatment may introduce additional delays.

For peer review only - http://bmjopen.bmj.com/site/about/guidelines.xhtml

The shorter total interval in Denmark likely reflects the significant reductions in cancer waiting times following implementation of national Danish Cancer Patient Pathways (CPPs) including PCP access to fast-track diagnostic work-up.[39] The findings are in keeping with higher relative survival and lower mortality in Denmark among symptomatic cancer patients diagnosed through primary care after the implementation of CPPs and with the accelerated increase in 5-year survival among Danish lung cancer patients diagnosed in 2010-2014 when compared to patients from earlier time periods.[40] While there is some inherent lead-time bias, the findings highlight the importance and feasibility of a timely diagnosis of lung cancer.

Conclusions

The study provides for the first time, comparable data allowing for detailed evaluation between jurisdictions of routes and intervals from symptom onset to treatment in lung cancer. Across countries there were discrepancies in symptoms, especially fatigue and weight loss reported by patients and their primary care physicians. The findings highlight differences especially for patients who waited the longest and quantifies achievements, thus allowing for more focused policy and practice initiatives. Some jurisdictions may be able to revise the organisation of pathways to shortern time intervals and ultimately improve patient experience and outcomes.

Future analysis will explore the impact of these intervals on stage and survival. Meanwhile, our results draw attention to the success of secondary care initiatives in decreasing treatment intervals and underlines the need for more concerted efforts in primary care.

For peer review only

List of abbreviations

ICBP M4 – International Cancer Benchmarking Partnership Module 4

PCP – Primary Care Physician

CTS – Cancer Treatment Specialist

CPP – Danish Cancer Patient Pathways

Acknowledgement

We are grateful to Catherine Foot, Martine Bomb, Brad Groves of Cancer Research UK for managing the programme. The ICBP Module 4 Working Group (Supplementary File 6) for their support and work keeping the study going. Christian Finley (Canada), David Baldwin (England) for reviewing the manuscript. Stefan Bergström, Jan Willem Coebergh, Jon Emery, Monique E van Leerdam, Marie-Louise Essink-Bot and Una MacLeod (the ICBP M4 Academic Reference Group) for providing independent peer review of the study protocol and analysis plan development. We thank all the patients, primary care physicians, cancer treatment specialists, and registry staff of all jurisdictions who took part in this study, and also the Danish Lung Cancer Registry for providing clinical information for the study.

ICBP Programme Board:

Aileen Keel (Scottish Government, Edinburgh, Scotland); Anna Boltong (Cancer Council Victoria, Carlton, Australia); Anna Gavin (Northern Ireland Cancer Registry, Queens University, Belfast, UK); David Currow (Cancer Institute New South Wales, Sydney, Australia); Gareth Davies (Wales Cancer Network, Cardiff, UK); Gunilla Gunnarsson (Swedish Association of Local Authorities and Regions, Stockholm, Sweden); Heather Bryant (Canadian Partnership Against Cancer, Toronto, Canada); Jane Hanson (Welsh Cancer National Specialist Advisory Group, Cardiff, UK); Kathryn Whitfield (Department of Health, Victoria Australia); Linda Rabeneck (Cancer Care Ontario, Toronto, Canada); Michael A Richards (Care Quality Commission, London, UK); Michael Sherar (Cancer Care Ontario, Toronto, Canada); Nicola Quin (Cancer Council Victoria, Carlton, Australia); Nicole Mittmann (Cancer Care Ontario, Toronto, Canada); Robert Thomas (Department of Health and Human Services, Victoria, Melbourne, Australia); Sara Hiom (Cancer Research UK); Sean Duffy (NHS England, London, UK); Chris Harrison (NHS England, London, UK); Søren Brostrøm (Danish Health and Medicines Authority, Copenhagen, Denmark); Stein Kaasa (University Hospital of Trondheim, Trondheim, Norway).

Author's contribution

For peer review only - http://bmjopen.bmj.com/site/about/guidelines.xhtml

UM, DW, PV, AZF, HJ planned the study design, data collection, carried out the analyses and wrote the draft manuscript. UM, PV, DW, HJ, AB, AKK, RJB, DHB, JK, VC, ATG, EG, EH, ML, RJW, YL, MM, DT, RDN, VW, IR and SH were responsible for local data collection (alongside the Working Group), management and interpretation, and have participated in writing and have approving the final manuscript version. JB, OB and OTB provided advice on the interpretation of results in their respective jurisdictions and comments or substantial edits on the manuscript, approving the final version.

Competing Interests

None

Funding:

This work was supported by: CancerCare Manitoba; Cancer Care Ontario; Canadian Partnership Against Cancer (CPAC); Cancer Council Victoria; Cancer Research Wales; Cancer Research UK; Danish Cancer Society; Danish Health and Medicines Authority; European Palliative Care Research Centre (PRC), Norwegian University of Science and Technology (NTNU); Northern Ireland Guidelines Audit and Implementation Network (GAIN); Macmillan Cancer Support; National Cancer Action Team; NHS England; Northern Ireland Cancer Registry, funded by the Public Health Agency NI; Norwegian Directorate of Health; Research Centre for Cancer Diagnosis in Primary Care (CaP), Aarhus University, Denmark; Scottish Government; Swedish Association of Local Authorities and Regions; University College London and NIHR Biomedical Research Centre at University College London NHS Foundation Trust; University of Edinburgh; Victorian Department of Health and Human Services; Welsh Government. The funding bodies had no influence on the design of the study and collection, analysis, and interpretation of data, in writing the manuscript or whether to publish the results.

Availability of data and material

The data that support the findings of this study are available from the named authors from each ICBP jurisdiction, but restrictions apply to the availability of these data and so are not publicly available. Data are however available from the authors upon reasonable request and with permission of the ICBP Programme Board. Please contact the ICBP Programme Management team, based at Cancer Research UK, with any queries (icbp@cancer.org.uk).

Ethics approval and consent to participate

For peer review only - http://bmjopen.bmj.com/site/about/guidelines.xhtml

For each local data collection, there were specific procedures and approvals which included anonymised data transfer to University College London and Aarhus University. Approvals were received from the following institutions: Cancer Council Victoria Human Research Ethics Committee [HREC 1125]; Health Research Ethics Board, University of Manitoba [HS15227 (H2012:105)]; Research Resource Ethics Committee, CancerCare Manitoba [RRIC#28-2012]; University of Toronto Research Ethics Board [27881]; The Danish Data Protection Agency [2013-41-2030]; Swedish Ethics Review Board, Uppsala [2013/306]; Norway Regional committees for medical and health research ethics [2013/136/REK nord]; England, Wales and Scotland, NRES Committee East Midlands – Derby 2, local R&D for each health board [11/EM/0420]; Northern Ireland ORECNI Ethical approval, local governance for each health Trust [11/EM/0420].

For peer review only

References

1. Ferlay J, Steliarova-Foucher E, Lortet-Tieulent J, et al. Cancer incidence and mortality patterns in Europe: Estimates for 40 countries in 2012. Eur J Cancer 2013;49(6):1374-403.

2. Allemani C, Weir HK, Carreira H, et al. Global surveillance of cancer survival 1995-2009: analysis of individual data for 25 676 887 patients from 279 population-based registries in 67 countries (CONCORD-2). Lancet 2015;385(9972):977-1010.

3. Neal RD, Robbé IJ, Lewis M, Williamson I, Hanson J. The complexity and difficulty of diagnosing lung cancer: findings from a national primary-care study in Wales. Prim Health Care Res Dev 2015 Sep;16(5):436-49

4. Francisci S, Minicozzi P, Pierannunzio D, et al. Survival patterns in lung and pleural cancer in Europe 1999-2007: Results from the EUROCARE-5 study. Eur J Cancer 2015;51(15):2242-53.

5. Coleman MP, Forman D, Bryant H, et al. Cancer survival in Australia, Canada, Denmark, Norway, Sweden, and the UK, 1995-2007 (the International Cancer Benchmarking Partnership): an analysis of population-based cancer registry data. Lancet 2011;377(9760):127-38.

6. Walter FM, Rubin G, Bankhead C, et al. Symptoms and other factors associated with time to diagnosis and stage of lung cancer: a prospective cohort study. Br J Cancer 2015;112 Suppl 1:S6-13.

7. Neal RD, Din NU, Hamilton W, et al. Comparison of cancer diagnostic intervals before and after implementation of NICE guidelines: analysis of data from the UK General Practice Research Database. Br J Cancer 2014;110(3):584-92.

8. Barrett J, Hamilton W. Pathways to the diagnosis of lung cancer in the UK: a cohort study. BMC Fam Pract 2008;9:31.

9. Baughan P, O'Neill B, Fletcher E. Auditing the diagnosis of cancer in primary care: the experience in Scotland. Br J Cancer 2009;101 Suppl 2:S87-91.

10. Guldbrandt LM, Fenger-Gron M, Rasmussen TR, et al. The role of general practice in routes to diagnosis of lung cancer in Denmark: a population-based study of general practice involvement, diagnostic activity and diagnostic intervals. BMC Health Serv Res 2015;15:21.

11. Torring ML, Frydenberg M, Hansen RP, et al. Evidence of increasing mortality with longer diagnostic intervals for five common cancers: a cohort study in primary care. Eur J Cancer 2013;49(9):2187-98.

12. Hansen RP, Vedsted P, Sokolowski I, et al. Time intervals from first symptom to treatment of cancer: a cohort study of 2,212 newly diagnosed cancer patients. BMC Health Serv Res 2011;11:284.

For peer review only

For peer review only - http://bmjopen.bmj.com/site/about/guidelines.xhtml

13. Bjerager M, Palshof T, Dahl R, et al. Delay in diagnosis of lung cancer in general practice. Br J Gen Pract 2006;56(532):863-8.

14. Rolke HB, Bakke PS, Gallefoss F. Delays in the diagnostic pathways for primary pulmonary carcinoma in Southern Norway. Respir Med 2007;101(6):1251-7.

15. Largey G, Ristevski E, Chambers H, et al. Lung cancer interval times from point of referral to the acute health sector to the start of first treatment. Aust Health Rev 2016.

16. Evans SM, Earnest A, Bower W, et al. Timeliness of lung cancer care in Victoria: a retrospective cohort study. Med J Aust 2016;204(2):75 e1-9.

17. Ellis PM, Vandermeer R. Delays in the diagnosis of lung cancer. J Thorac Dis 2011;3(3):183-8.

18. Lo DS, Zeldin RA, Skrastins R, et al. Time to treat: a system redesign focusing on decreasing the time from suspicion of lung cancer to diagnosis. J Thorac Oncol 2007;2(11):1001-6.

19. Butler J, Foot C, Bomb M, et al. The International Cancer Benchmarking Partnership: an international collaboration to inform cancer policy in Australia, Canada, Denmark, Norway, Sweden and the United Kingdom. Health Policy 2013;112(1-2):148-55.

20. Weller D, Vedsted P, Anandan C, et al. An investigation of routes to cancer diagnosis in 10 international jurisdictions, as part of the International Cancer Benchmarking Partnership: survey development and implementation. BMJ Open 2016;6(7):e009641.

21. Weller D, Vedsted P, Rubin G, et al. The Aarhus statement: improving design and reporting of studies on early cancer diagnosis. Br J Cancer 2012;106(7):1262-7.

22. Jenkinson C, Coulter A, Wright L. Short form 36 (SF36) health survey questionnaire: normative data for adults of working age. Bmj. 1993 May 29;306(6890):1437-40

23. TNM classification of malignant tumours / edited by L.H. Sobin, M.K. Gospodarowicz, and Ch. Wittekind. — 7th ed. UICC International Union Against Cancer. ISBN 978-1-4443-3241-4

24. Koenker R, Bassett G. Regression quantiles. Econometrica 1978; 46:33-50

25. Machado JAF, Santos Silva JMC. Quantiles for Counts. J Amer Statistical Assoc 2005, 100(472): 1226-1237.

26. Miranda A. QCOUNT: Stata program to fit quantile regression models for count data. Boston: Boston College Department of Economics; 2006

27. Lin LI. A concordance correlation coefficient to evaluate reproducibility. Biometrics 1989;45(1):255-68.

For peer review only - http://bmjopen.bmj.com/site/about/guidelines.xhtml

28. Walters S, Maringe C, Coleman MP, et al. Lung cancer survival and stage at diagnosis in Australia, Canada, Denmark, Norway, Sweden and the UK: a population-based study, 2004-2007. Thorax 2013;68(6):551-64.

29. Nicholson BD, Mant D, Neal RD, et al. International variation in adherence to referral guidelines for suspected cancer: a secondary analysis of survey data. Br J Gen Pract 2016;66(643):e106-13.

30. Shim J(1), Brindle L, Simon M, George S. A systematic review of symptomatic diagnosis of lung cancer. Fam Pract. 2014 Apr;31(2):137-48

31. Hamilton W, Peters TJ, Round A, et al. What are the clinical features of lung cancer before the diagnosis is made? A population based case-control study. Thorax 2005;60(12):1059-65.

32. Beckett P, Tata LJ, Hubbard RB. Risk factors and survival outcome for non-elective referral in non-small cell lung cancer patients-analysis based on the National Lung Cancer Audit. Lung Cancer 2014;83(3):396-400.

33. Lim A, Mesher D, Gentry-Maharaj A, et al. Time to diagnosis of Type I or II invasive epithelial ovarian cancers: a multicentre observational study using patient questionnaire and primary care records. BJOG 2016;123(6):1012-20.

34. Neal RD, Allgar VL, Ali N, et al. Stage, survival and delays in lung, colorectal, prostate and ovarian cancer: comparison between diagnostic routes. Br J Gen Pract 2007;57(536):212-9.

35. Guldbrandt LM, Moller H, Jakobsen E, et al. General practice consultations, diagnostic investigations, and prescriptions in the year preceding a lung cancer diagnosis. Cancer Med 2017;6(1):79-88.

36. Danish National Board of Health. Pakkeforløb for lungekræft [Cancer Patient Pathway – Lung Cancer], 2016) National Board of Health, Copenhagen. Available at: https://www.sst.dk/da/sygdom-og-behandling/kraeft/pakkeforloeb/~/media/89192ECB2709401CAD8E4BBB0304691E.ashx [accessed 25 September 2017]

37. O'Rourke N, Edwards R. Lung cancer treatment waiting times and tumour growth. Clin Oncol (R Coll Radiol). 2000;12(3):141-4.

38. Wang J, Mahasittiwat P, Wong KK, et al. Natural growth and disease progression of non-small cell lung cancer evaluated with 18F-fluorodeoxyglucose PET/CT. Lung Cancer 2012;78(1):51-6.

39. Jensen H, Tørring ML,Vedsted P. Prognostic consequences of implementing cancer patient pathways in Denmark: a comparative cohort study of symptomatic cancer patients in primary care. BMC Cancer 2017;17:627.

40. Allemani C, Matsuda T, Di Carlo V, et al. Global surveillance of trends in cancer survival 2000–14 (CONCORD-3): analysis of individual records for 37 513 025 patients

For peer review only - http://bmjopen.bmj.com/site/about/guidelines.xhtml

diagnosed with one of 18 cancers from 322 population-based registries in 71 countries. Lancet 2018 Mar 17;391(10125):1023-1075.

For peer review only

For peer review only - http://bmjopen.bmj.com/site/about/guidelines.xhtml

Figure 1: Time intervals from onset of symptoms to start of treatment based on the Aarhus Statement [21]

Figure 2: Differences in 50th, 75th and 90th percentiles of the intervals (days) between Wales as the reference and the other nine jurisdictions.

The data are adjusted for differences in age, gender and comorbidity. The bars in black show significant differences in intervals.

For peer review only

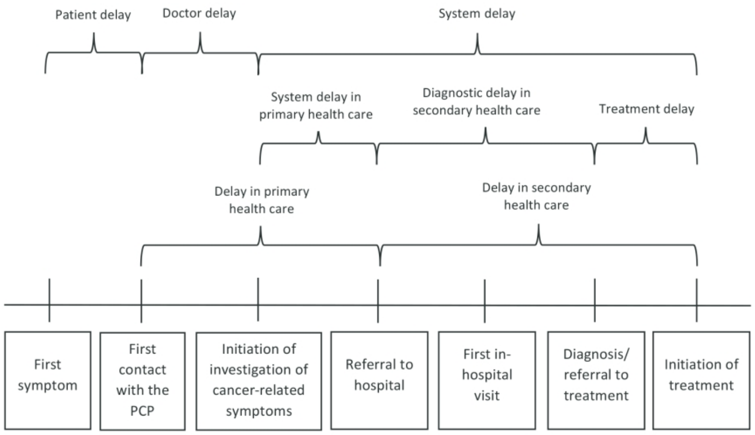

Time intervals from onset of symptoms to start of treatment based on the Aarhus Statement [21]

209x297mm (300 x 300 DPI)

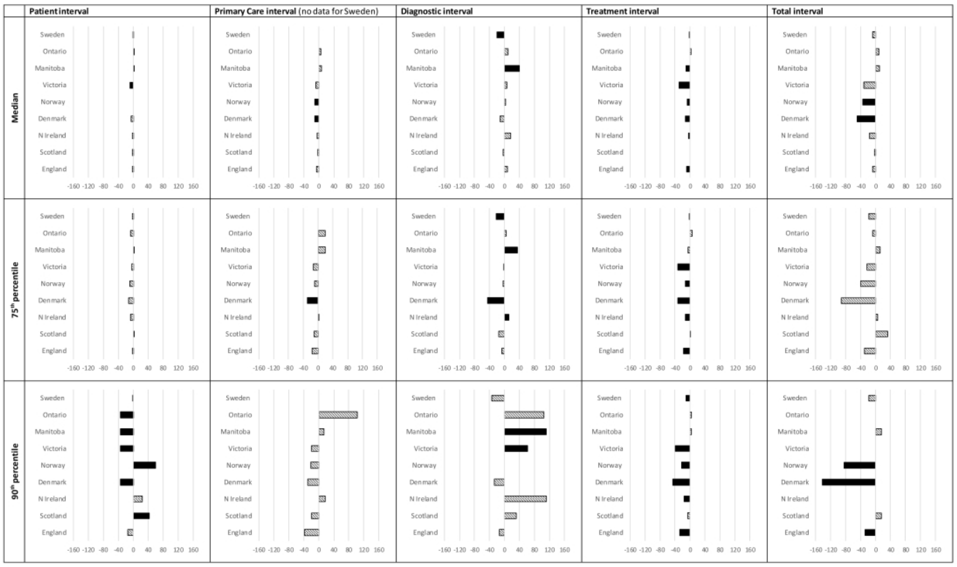

Differences in 50th, 75th and 90th percentiles of the intervals (days) between Wales as the reference and the other nine jurisdictions.

420x297mm (300 x 300 DPI)

ICBP International Cancer Benchmarking Partnership

Supplementary File 1: Patient Questionnaire

International Cancer Benchmarking Partnership Module 4

Patient questionnaire
Lung Cancer

Thank you very much for taking the time to fill in this questionnaire – it should take about 20 minutes to complete. We are sending the questionnaire to a large sample of people who we understand have had a diagnosis of lung cancer. If this has been sent to you in error and you do not have cancer, please do not continue and return the documents in the prepaid envelope.

Our aim is to gain a better understanding of the process by which people have their cancer diagnosed. We would also like to find out more about the symptoms they experience (if any), and the pathway they follow from start of symptoms to treatment of their cancer. This will help in identifying ways in which cancers can be diagnosed quickly and effectively. Thank you once again for your time.

This information is confidential and will not be passed to anyone involved in your treatment.

Name: ____________________

Date of Birth: ____________________

Address: ____________________

____

____

Copyright property of the International Benchmarking Partnership (ICBP)
and Module 4 collaborators. Usage must be authorised

Sample
For peer review only

ICBP
International Cancer
Benchmarking Partnership

Consent form

Please read the consent form and sign your name and date **BELOW**.

If you require any clarification, please do not hesitate to ring the study team members. Their contact details are found on the information sheet.

Please be reassured that your responses are completely confidential and will not be passed to anyone involved in your treatment. For the purposes of the study it is important that you agree to consent to all the statements listed below.

- I confirm that I have read the attached information sheet and I understand why the research is being done.
- I am willing for the team to request information from my GP and hospital doctors which is relevant to the audit as described in the information sheet.
- I give permission for my details (name, address) to be given to the cancer registry (NHS Information Centre for Health and Social Care) for follow up.
- I agree for the information I have provided and any other relevant information from my medical records to be stored as described in the information sheet under the custodianship of University College London.
- I consent to sharing of coded data which contains no personal identifiers between researchers, some of whom are located outside the European Union.
- I consent for use of my data if I become mentally incapacitated during the course of the project.

I agree to all the statements listed and consent to participate in the study.

Name (Please print)

Signature: Date:

If we have any questions, may we phone you for clarification?
(Please tick) ☐ **Yes** ☐ **No**

If **Yes**, please provide your telephone number:

Sample

For peer review only

Copyright property of the International Benchmarking Partnership (ICBP)
and Module 4 collaborators. Usage must be authorised

ICBP
International Cancer
Benchmarking Partnership

1. Please can you confirm the details of your GP/GP practice (name, practice address – as best as you can remember): We appreciate that you may have more than one GP involved in your care – in which case, we are interested in the GP you would say provides the majority of your care, particularly relating to the cancer you've had diagnosed.

Name of doctor ____________________

Name of practice ____________________

Address ____________________

____

____

Postcode ____________________

Town ____________________

Sample

For peer review only

Copyright property of the International Benchmarking Partnership (ICBP)
and Module 4 collaborators. Usage must be authorised

ICBP
International Cancer
Benchmarking Partnership

2. Which of the following best describes the events which led to your diagnosis of cancer? (please tick only **ONE** answer)

	✓
I had symptoms/I noticed a bodily change and went to see a doctor (e.g. GP)	
I had symptoms/I noticed a bodily change and went/was taken to Accident and Emergency (A&E)	
I had seen a doctor/GP with symptoms, but went/was taken to Accident and Emergency (A&E) when things worsened	
I was being investigated by my doctor(s) for another problem during which time the cancer was discovered	
Other (please describe):	

Sample

For peer review only

Copyright property of the International Benchmarking Partnership (ICBP) and Module 4 collaborators. Usage must be authorised

ICBP
International Cancer Benchmarking Partnership

3. The following health concerns or symptoms are commonly experienced with lung cancer.

Cough
Breathlessness
Being short of breath
Chest pain / Pain on breathing
Coughing up blood
Bloody sputum/blood in spit
Unexplained weight loss
Loss of appetite
Fatigue

Sample

Please write down ALL health concern(s) or symptom(s) you may have had before contacting a doctor or taking part in screening. It does not matter if they are not included in the list above:

Please write your health concern(s) or symptom(s) in the boxes below:
1)
2)
3)
4)
5)
6)

This is not applicable to me (e.g. I did not have any symptoms), please tick	✓

For peer review only

Copyright property of the International Benchmarking Partnership (ICBP)
and Module 4 collaborators. Usage must be authorised

ICBP
International Cancer
Benchmarking Partnership

4. Please write down your best estimate of the date you noticed the first of these health concern(s) or symptom(s). If you cannot remember the exact date, you can fill in the month and the year.

Day (optional), month, year

D	D	M	M	Y	Y	Y	Y

	✓
This is not applicable to me (e.g. I had no symptoms), please tick	

5. Approximately how long did you have health concern(s) or symptom(s) before contacting a doctor? (Please think of the first visit to the doctor, not re-visits after that). Please tick only **ONE** answer.

	✓
Less than 1 week	
1-2 weeks	
3-4 weeks	
5-7 weeks	
2-5 months	
6-12 months	
More than 12 months	

	✓
This is not applicable to me (e.g. I had no symptoms), please tick	

Sample

For peer review only

Copyright property of the International Benchmarking Partnership (ICBP) and Module 4 collaborators. Usage must be authorised

ICBP
International Cancer
Benchmarking Partnership

6a. Once you contacted a practice about your health concern(s) or symptom(s), how long did it take to get an appointment with a doctor? (Please think of the first visit to the doctor, to discuss your health concern(s) or symptom(s)).
Please tick only **ONE** answer.

	✓
Same day/next day	
Within 1 week	
1-2 weeks	
3-4 weeks	
Longer	

If there was no waiting time (e.g. you went/were taken to A&E), please tick this box	

This is not applicable to me (e.g. I had no symptoms), please tick	

Sample

6b. What was the date you first saw your doctor about your health concern(s) or symptom(s)? If you cannot remember the exact date, you can fill in the month and the year.

Day (optional), month, year

D	D	M	M	Y	Y	Y	Y

	✓
This is not applicable to me (e.g. I had no symptoms), please tick	

Copyright property of the International Benchmarking Partnership (ICBP) and Module 4 collaborators. Usage must be authorised

ICBP
International Cancer
Benchmarking Partnership

7. How many times did you visit the following for the investigation of your symptoms before your cancer was diagnosed?

	Please write down the number of visits
GP	
Hospital	
Consultant/specialist outside of a hospital	

	✓
This is not applicable to me (e.g. I had no symptoms), please tick	

8a. After your doctor referred you to a specialist, how long did it take you to get an appointment? Please tick only **ONE** answer.

	✓
Less than 1 week	
1-2 weeks	
3-4 weeks	
5-7 weeks	
2-5 months	
6-12 months	
More than 12 months	

	✓
This is not applicable to me (e.g. my doctor did not refer me), please tick	

Sample
For peer review only

Copyright property of the International Benchmarking Partnership (ICBP)
and Module 4 collaborators. Usage must be authorised

For peer review only - http://bmjopen.bmj.com/site/about/guidelines.xhtml

ICBP
International Cancer
Benchmarking Partnership

8b. What was the date of your first appointment with a doctor, involved in investigating and/or treating your cancer, to whom you were referred? If you cannot remember the exact date, you can fill in the month and the year.

Day (optional), month, year

D	D	M	M	Y	Y	Y	Y

This is not applicable to me (e.g. my doctor did not refer me), please tick	✓

9. What was the date you were told you had cancer? If you cannot remember the exact date, you can fill in the month and the year.

Day (optional), month, year

D	D	M	M	Y	Y	Y	Y

Sample

For peer review only

Copyright property of the International Benchmarking Partnership (ICBP) and Module 4 collaborators. Usage must be authorised

ICBP
International Cancer
Benchmarking Partnership

10. Have you had any of the following treatments for your cancer yet? If so, please can you estimate the date this treatment started? Please tick **ALL** that apply. If you cannot remember the exact date, you can fill in the month and the year.

	Type of treatment		Date of treatment (give first date if you had more than one)
a.	Surgery	☐ Yes ☐ No	Day (optional), month, year D D M M Y Y Y Y
b.	Chemotherapy	☐ Yes ☐ No	Day (optional), month, year D D M M Y Y Y Y
c.	Radiotherapy	☐ Yes ☐ No	Day (optional), month, year D D M M Y Y Y Y
d.	Other Please specify:	☐ Yes ☐ No	Day (optional), month, year D D M M Y Y Y Y
e.	Treatment not started yet	☐ Yes	

Sample
For peer review only

Copyright property of the International Benchmarking Partnership (ICBP) and Module 4 collaborators. Usage must be authorised

ICBP
International Cancer
Benchmarking Partnership

11. Who is the consultant doctor who has taken responsibility for diagnosing and or/treating your cancer?

Name of consultant:
Hospital name:
Hospital department:

Please can you answer some more general questions about your health?
It will help us in interpreting your responses to this questionnaire to know about your general health and other health problems you may have had in the past.

12. Looking back to the 2 years before you were diagnosed with cancer, would you say your general health was (Please tick only **ONE** answer.):

Very good	
Good	
Fair	
Poor	
Very poor	

13. Have you been treated before for any of the conditions below?
Please tick 'yes' or 'no' for each condition:

Heart disease	☐ **Yes**	☐ **No**
Stroke	☐ **Yes**	☐ **No**
Lung disease (excluding lung cancer)	☐ **Yes**	☐ **No**
Diabetes	☐ **Yes**	☐ **No**

Sample
For peer review only

Copyright property of the International Benchmarking Partnership (ICBP) and Module 4 collaborators. Usage must be authorised

ICBP
International Cancer Benchmarking Partnership

Finally, a little more information about you. The information you provide below will help us to analyse the results of the survey in more detail.

14. Which of these best describes your ethnic group? (please tick one box, as appropriate). If you are descended from more than one ethnic or racial group, please tick the group you consider you belong to, or tick 'any other ethnic group'.

	✓		✓		✓		✓
White		Chinese		Black - Caribbean		Black - African	
Black - other		Indian		Pakistani		Bangladeshi	
Any other ethnic group, please specify:							

Sample

15. What is the main language spoken in your home? Please tick

	✓
English	
Other, please specify:	

16. What is the highest level of education you have achieved?
Please tick only **ONE** answer.

	✓
Finished school at or before the age of fifteen	
Completed GCSEs, O-levels or equivalent	
Completed A Levels or equivalent	
Completed further education but not a degree	
Completed a Bachelor's degree / Masters degree / PhD	
Other, please specify:	

Copyright property of the International Benchmarking Partnership (ICBP) and Module 4 collaborators. Usage must be authorised

For peer review only - http://bmjopen.bmj.com/site/about/guidelines.xhtml

ICBP
International Cancer
Benchmarking Partnership

17. Have you ever smoked cigarettes, including hand-rolled ones, pipes or cigars?

☐ Yes ☐ No

18. Are you a current smoker, smoking either cigarettes, including hand-rolled ones, pipes or cigars?

☐ Yes ☐ No

19. If you are a current smoker or have smoked in the past, how many cigarettes, including hand-rolled ones, pipes or cigars on average do you smoke/have you smoked per day?

Number per day:	

Sample

For peer review only

Copyright property of the International Benchmarking Partnership (ICBP) and Module 4 collaborators. Usage must be authorised

ICBP
International Cancer Benchmarking Partnership

20. Further comments

Please add anything else that you would like to tell us about your cancer diagnosis or treatment.

Sample

For peer review only

Thank you very much for taking the time to complete this questionnaire.

Copyright property of the International Benchmarking Partnership (ICBP) and Module 4 collaborators. Usage must be authorised

ICBP International Cancer Benchmarking Partnership

Supplementary File 2: Primary Care Questionnaire

International Cancer Benchmarking Partnership Module 4

Primary Care Audit

Lung Cancer

Thank you very much for agreeing to fill in this questionnaire. As part of an international study examining differences in cancer survival, we are sending the questionnaire to health care providers of a sample of consented patients with cancer.

Our aim is to gain a better understanding of the process by which people have their cancer diagnosed – the symptoms they experience, and the pathway they follow from onset of symptoms to treatment of their cancer. This will help in identifying ways in which cancers can be diagnosed and treated quickly and effectively. Thank you once again for your time.

Please can you refer to your patient's notes in completing the questionnaire as this will help in obtaining accurate data on time points.

Sample

If you would prefer to return this questionnaire without the patient details, please tear off along the dotted line

ID-number: Jurisdiction-ID + Patient-ID:

Copyright property of the International Benchmarking Partnership (ICBP) and Module 4 collaborators. Usage must be authorised

ICBP
International Cancer
Benchmarking Partnership

Sample

For peer review only

Patient information

ID-number: Jurisdiction-ID + Patient-ID:

Full name:

Address:

Postcode:

Date of birth: D D M M Y Y Y Y

Copyright property of the International Benchmarking Partnership (ICBP)
and Module 4 collaborators. Usage must be authorised

ICBP
International Cancer
Benchmarking Partnership

1. Duration of symptoms

Please estimate how long your patient had symptom(s), attributable to lung cancer, before attending your practice (or other health service).

We appreciate that identifying a 'date of first symptom' is not always straightforward – particularly when there are multiple and/or chronic symptoms. Nevertheless, we hope you can provide a 'best estimate'.

**Estimate of symptom duration** (please tick **one**):	✓	**What were the symptoms?** Please describe:
Less than 1 week		
1 to 4 weeks		
5 to 7 weeks		
2-5 months		
6-12 months		
More than 12 months		
Not possible to estimate		
No symptoms (e.g. screen detected cancers)		

Sample

For peer review only

Copyright property of the International Benchmarking Partnership (ICBP) and Module 4 collaborators. Usage must be authorised

ICBP
International Cancer
Benchmarking Partnership

2. Pathway of presentation

2.1 Through what route did the patient first present? Please tick **ONE**:

	✓	
Your patient first presented to primary care (either in-hours or out-of-hours)		Please can you provide your best approximation of the date of this **primary care** visit D D M M Y Y Y Y
Your patient presented straight to A&E (with or without your involvement)		
Your patient first presented to primary care, but then at a later date presented to A&E as an emergency (with or without your involvement)		Please can you provide your best approximation of the date of this **primary care** visit D D M M Y Y Y Y
Other – please describe:		

Sample

For peer review only

Copyright property of the International Benchmarking Partnership (ICBP) and Module 4 collaborators. Usage must be authorised

ICBP
International Cancer Benchmarking Partnership

3. Date you ordered any tests/investigations in response to symptom(s).

We are interested in any kind of tests/investigations (e.g. imaging etc) that you may have ordered. Please only consider the tests/investigations that you ordered yourself. Please tick **all** that apply and put in the date that the test/investigation was ordered:

	✓	
Chest x-ray		D D M M Y Y Y Y
MRI scan		D D M M Y Y Y Y
CT scan		D D M M Y Y Y Y
PET scan		D D M M Y Y Y Y
Sputum test		D D M M Y Y Y Y
Lung biopsy		D D M M Y Y Y Y
Bronchoscopy		D D M M Y Y Y Y
Other (please specify):		D D M M Y Y Y Y

4. Date of referral to specialist medical services

At what date did you **first** refer the patient to hospital or another specialist transferring the responsibility for on-going investigation/treatment to other medical services?

D D M M Y Y Y Y

Sample

For peer review only

Copyright property of the International Benchmarking Partnership (ICBP) and Module 4 collaborators. Usage must be authorised

ICBP
International Cancer Benchmarking Partnership

5. Nature of this referral

5.1 Do you know the date that the patient was seen for this referral?

☐ **Yes**, please provide the date: D D M M Y Y Y Y

☐ **No**

5.2 If you did make a referral to specialist services, which of the following best describes the nature/characteristics of this referral? Please tick **one**.

	✓
Emergency admission: a referral to A&E (or equivalent) for immediate admission	
An urgent referral for assessment of cancer symptoms/signs/test results (Note this will be within 2 weeks for England/Wales)	
A less urgent referral in which cancer is raised as a possibility (Note this will be greater than 2 weeks for England/Wales)	
A more general referral for investigation and assessment without cancer mentioned	
No referral was made	
Other – please describe	

5.3 Would you say this patient's diagnostic pathway was conducted predominantly in the public or private system? Please tick **one**.

	✓
Public healthcare system	
Private healthcare system	

Sample

For peer review only

Copyright property of the International Benchmarking Partnership (ICBP) and Module 4 collaborators. Usage must be authorised

ICBP
International Cancer
Benchmarking Partnership

6. Date of lung cancer diagnosis

This can be decided in different ways. Please tick **all** that apply.
Please provide whichever of the following dates you have to hand:

	✓	
Date of histological confirmation [ideal]		D D M M Y Y Y Y
Date results of investigation (histological or other) confirming cancer received		D D M M Y Y Y Y
Date patient was told		D D M M Y Y Y Y
Date biopsy undertaken		D D M M Y Y Y Y
Date patient was first admitted to hospital because of the malignancy		D D M M Y Y Y Y
Other (please specify)		D D M M Y Y Y Y

Sample

For peer review only

Copyright property of the International Benchmarking Partnership (ICBP)
and Module 4 collaborators. Usage must be authorised

ICBP
International Cancer
Benchmarking Partnership

7. Additional information

Finally, we are interested to know what other conditions your patient has, and the severity/impact of these conditions

Have you and/or any of your partners treated this patient (or has the patient been to hospital) for any of the following conditions? Please tick **all** that apply:

Cardiovascular disease	☐ **Yes**	☐ **No**
Stroke	☐ **Yes**	☐ **No**
Lung disease (except lung cancer)	☐ **Yes**	☐ **No**
Diabetes	☐ **Yes**	☐ **No**

Are there any other comments you would like to make about this patient?

Sample
For peer review only

Name (and title):

Signature:

Date:

Thank you very much for taking the time to complete this questionnaire.

Copyright property of the International Benchmarking Partnership (ICBP)
and Module 4 collaborators. Usage must be authorised

International Cancer Benchmarking Partnership

International Cancer Benchmarking Partnership Module 4

Specialist Care Audit

Lung Cancer

Thank you very much for agreeing to fill in this questionnaire – it should take about 10 minutes to complete. As part of an international study examining differences in cancer survival, we are sending the questionnaire to health care providers of a sample of patients with cancer.

Our aim is to gain a better understanding of the process by which people have their cancer diagnosed – the symptoms they experience, and the pathway they follow from onset of symptoms to treatment of their cancer. We hope you can help us with information on this patient's cancer journey once they were referred to specialist cancer services. This will help in identifying ways in which cancers can be diagnosed and treated quickly and effectively.

Sample

Thank you once again for your time

Please can you refer to your patient's notes in completing the questionnaire, as this will help in obtaining accurate data on time points.

If you would prefer to return this questionnaire without the patient details, please tear off along the dotted line.

Your patient

____

is participating in the study.

Copyright property of the International Benchmarking Partnership (ICBP) and Module 4 collaborators. Usage must be authorised

ICBP
International Cancer
Benchmarking Partnership

Sample

Patient information

ID-number: Jurisdiction-ID + Patient-ID:

Full name:

Address:

Postcode:

Date of birth: D D M M Y Y Y Y

Copyright property of the International Benchmarking Partnership (ICBP)
and Module 4 collaborators. Usage must be authorised

For peer review only - http://bmjopen.bmj.com/site/about/guidelines.xhtml

ICBP
International Cancer
Benchmarking Partnership

1. **Date patient first attended hospital/specialist services related to their cancer diagnosis.** We appreciate this date can at times be difficult to identify, particularly when there have been multiple visits in the lead up to a definitive diagnosis. Put another way, it's the date that the hospital/specialist service assumed responsibility for on-going investigation/treatment for your patient.

Day (optional), month, year

D	D	M	M	Y	Y	Y	Y

2. **How was the patient referred to the hospital/specialist services related to their cancer diagnosis?** Please tick.

Was it through a:

	✓		✓
GP referral		Referral from respiratory clinic	
Medical specialist/Consultant referral		Other referral – please specify:	

Sample

3. **Where did this first contact/appointment happen?** Please tick.
Which of the following best describes where this first contact/appointment took place?

	✓		✓
Emergency department ('A&E')		Medical outpatient department, please specify which department	
Oncology general outpatient department		Surgical outpatient department, please specify which department	
Other – please specify:			

Copyright property of the International Benchmarking Partnership (ICBP)
and Module 4 collaborators. Usage must be authorised

ICBP
International Cancer
Benchmarking Partnership

4. Date of diagnosis

This can be decided in different ways.
Please tick and complete as many of the following dates as possible.

	✓	
Date of histological confirmation (ideal)		Day (optional), month, year D D M M Y Y Y Y
Date results of investigation confirming cancer received		Day (optional), month, year D D M M Y Y Y Y
Date patient was told		Day (optional), month, year D D M M Y Y Y Y
Date of biopsy		Day (optional), month, year D D M M Y Y Y Y
Date patient was first admitted to hospital because of the malignancy		Day (optional), month, year D D M M Y Y Y Y
Date of MDT confirmation of diagnosis		Day (optional), month, year D D M M Y Y Y Y
Other (please specify):		Day (optional), month, year D D M M Y Y Y Y

Sample

Copyright property of the International Benchmarking Partnership (ICBP)
and Module 4 collaborators. Usage must be authorised

ICBP
International Cancer
Benchmarking Partnership

5. Date treatment for the cancer commenced

Based on your records, when would you say that any treatment specifically targeting the patient's cancer started?

Day (optional), month, year

D	D	M	M	Y	Y	Y	Y

6. Additional information

Please can you provide any further information on the patient's cancer:

**TNM**, please tick as appropriate:	
0	
I	
IIA	
IIB	
IIC	
IIIA	
IIIB	
IIIC	
IV	
Not able to stage	

Sample

Copyright property of the International Benchmarking Partnership (ICBP) and Module 4 collaborators. Usage must be authorised

ICBP
International Cancer
Benchmarking Partnership

6.1 Histological subtype:

	✓
Squamous cell carcinoma	
Adenocarcinoma	
Large cell carcinoma	
Non-small carcinoma	
Small cell carcinoma	
Carcinoid	
Unclassified	
Other (please specify):	

Sample

Copyright property of the International Benchmarking Partnership (ICBP)
and Module 4 collaborators. Usage must be authorised

ICBP
International Cancer
Benchmarking Partnership

Further comments

Sample

Copyright property of the International Benchmarking Partnership (ICBP)
and Module 4 collaborators. Usage must be authorised

ICBP
International Cancer
Benchmarking Partnership

Name (and title):

Signature:

Date:

Sample

Are you a ... (please tick below): ✓

Surgeon	
Medical Oncologist	
Clinical Oncologist	
Clinical Nurse Specialist	
Other (please specify):	

Thank you very much for taking the time to complete this questionnaire.

Copyright property of the International Benchmarking Partnership (ICBP)
and Module 4 collaborators. Usage must be authorised

Supplementary File 4: Rules for missing, incomplete, multiple response and out of range data and for calculating intervals

1. Oversampling/Participation in local screening trials<br>a) To handle oversampling in Ontario, include only the first 315 consecutive lung cancer patients;<br>b) In jurisdictions with no national screen program: exclude patients participated in local screen trials.
2. Language/Participation in study/Presence of cancer<br>Exclude patients who checked "No, I don't understand the language" or "I don't want to participate in this study" or "I don't have cancer".
3. Survey responders<br>a) Exclude Patient/PCP/Specialist survey from the analysis, if it was not written by Patient/PCP/Specialist (example: a medical oncologist completed a PCP survey);<br>b) In the case of duplicates, include only the first survey (example: 2 specialists completed surveys for the same patient).
4. Gender<br>Exclude patients with unknown Gender.
5. Age<br>a) Exclude patients with unknown age;<br>b) Exclude patients younger 40 years;<br>c) Use registry data, if Age is reported by both patient and registry.
6. No cancer or Previous cancer in the same organ<br>a) Exclude patients with no cancer based on registry data;<br>b) Exclude patients with previous cancer in the same organ based on data from registry or free-text for Presentation in the patient survey.
7. Date of consent<br>Exclude patients with date of consent which is unknown, before 01.01.2013 or in the future.
8. Multiple responses to Dates<br>If multiple responses were given to the dates (of first symptom; screening; first presentation to primary care; referral; diagnosis; treatment start), then use the earliest date.
9. Order of Dates<br>The dates must be in the following order –<br>a) First symptom; first presentation to Primary Care; referral; diagnosis; treatment start.<br>b) Screening; diagnosis; treatment start.<br><br>If not, check for mistakes.
10. Date of first symptom<br>Date of first symptom is defined as date of first symptom from patient data.
11. Date of first presentation<br>Date of first presentation to Primary Care is defined as (in the order of declining priority):<br>a) date of first presentation to Primary Care from PCP data;<br>b) date of first presentation to Primary Care and A&E from PCP data;

For peer review only - http://bmjopen.bmj.com/site/about/guidelines.xhtml

c) date of first presentation to Primary Care from patient data.
12. Date of referral<br>Date of referral is defined as date of referral from PCP data.
13. Date of screening<br>Date of screening is defined as (in the order of declining priority):<br><br>a) date of screening from registry;<br><br>b) date of screening from patient data
14. Date of diagnosis<br>*Definition*<br>a) If Registry reports both date of histological confirmation and date of confirming investigation, then use date of histological confirmation.<br>b) Date of diagnosis (based on patient data, PCP data, specialist data, registry data) is defined as (in the order of declining priority):<br>- date of diagnosis from registry;<br>- date of histological confirmation (from specialist data, PCP data);<br>- date of biopsy (from specialist data, PCP data);<br>- date of confirming investigation (from specialist data, PCP data);<br>- date of first hospital admission (from specialist data, PCP data);<br>- date of MDT confirmation (from specialist data, PCP data);<br>- date patient was told (from specialist data, PCP data);<br>- other date of diagnosis (from specialist data, PCP data, patient data);<br><br>Choose a Date from a lower level of hierarchy, if the Date from a higher level is after the Date of consent or more than 9 months (=271 days) before the Date of consent.<br><br>*Exclusion criteria*<br><br>a) Unknown date of diagnosis;<br>b) Date of diagnosis is after the date of consent;<br>c) Date of diagnosis is more than 9 months before the Date of consent.
15. Date of treatment start<br>a) Date of treatment start from patient data is defined as the earliest of the treatment dates for Surgery, Chemo, Radio and Other;<br>b) Date of treatment start (based on registry data, specialist data, patient data) is defined as (in the order of declining priority):<br>- date of treatment start from registry data,<br>- date of treatment start from specialist data,<br>- date of treatment start from patient data,<br>- anticipated date of treatment from patient data.
16. Imputation of missing day in the date<br>Imputation rules for missing day (given month and year are known):<br><br>a) Set missing day to ‘16’;<br>b) Consider adjacent dates in a backwards order (from “Treatment” to “First symptom”). For each pair of such adjacent dates: If dates are not in a logical order (e.g.

For peer review only - http://bmjopen.bmj.com/site/about/guidelines.xhtml

"Treatment" is before "Diagnosis"), but month and year are the same in both dates, and the day was imputed to '16' in one of the dates:<br>- Recode the day imputed earlier to '16' to the day from the adjacent date
17. <u>Considering time</u><br>If patient gave multiple answers to the "How long did you have symptoms before contacting a doctor?" question, then use the option with the shortest time interval.
18. <u>Delay arranging appointment</u><br>If patient gave multiple answers to the "How long did it take to get an appointment with PCP?" question, then use the option with the shortest time interval.
19. <u>Duration of symptoms</u><br>If PCP gave multiple answers to the "Duration of symptoms" question, then use the option with the shortest time interval.
20. <u>Definition of Routes to diagnosis</u><br>A. *Define Route within a Data Source*<br>1. Review the free-text for Route (Patient, PCP sources) and re-code, if possible.<br>2. If PCP reports 'Other' as Route and at least one symptom (or "Duration of Symptoms") or if Patient reports 'Other' as Route and at least one symptom (or date of first symptom or "Consider waiting time" or "Delay arranging appointment"), then re-code the Route in the corresponding data source to 'Other non-screen-detected'-option.<br>3. In the case of multiple Routes responses (Patient, PCP sources) - use a single option (in the order of declining priority):<br>a) 'VisitPCP and AE',<br>b) 'VisitPCP´, 'AE´ (if both 'VisitPCP´ and 'AE´ are given, then re-code as 'VisitPCP and AE'),<br>c) 'Other non-screen-detected',<br>d) 'Investigation for another problem' ,<br>e) 'Other"<br><br>B. *Define Route from Alternative Data*<br><br>If Route hasn't been reported in either of data sources, then define it 'Other non-screen-detected', if PCP reports at least one symptom (or "Duration of symptoms"); or if Patient reports at least one symptom (or date of first symptom or "Consider waiting time" or "Delay arranging appointment");<br><br>C. *Define Route from Data Source Hierarchy*<br>1. In all jurisdictions, except Sweden – use Route data from (in the order of declining priority):<br>a) PCP data;<br>b) Patient data;<br>2. In Sweden – use Route data from Patient data.
21. <u>Patient interval</u><br>The Patient interval for non-screen-detected patients is defined as (in the order of declining priority):<br>a) "Date of first presentation to Primary Care" (rule 11) minus "Date of first symptom" (rule 10);

For peer review only - http://bmjopen.bmj.com/site/about/guidelines.xhtml

b) If the interval in (a) is unknown or negative: Calculate the interval as the low boundary of "Considering time" (rule 17) plus the low boundary of "Delay arranging appointment" (rule 18);
c) If the interval in (a) is unknown or negative and the interval in (b) is unknown: Calculate the interval as the low boundary of "Duration of symptoms interval" (rule 19).

22. Primary Care interval
The Primary Care interval for non-screen-detected is defined as "Date of referral" (rule 12) minus "Date of first presentation to Primary Care" (rule 11).

23. Diagnostic interval
a) The Diagnostic interval for non-screen-detected is defined as "Date of diagnosis" (rule 14) minus "Date of first presentation to Primary Care" (rule 11);
b) The Diagnostic interval for screen-detected patients is defined as "Date of diagnosis" (rule 14) minus "Date of screening" (rule 13).

24. Treatment interval
The Treatment interval is defined as "Date of treatment start" (rule 15) minus "Date of diagnosis" (rule 14).

25. Total interval
a) The Total interval for non-screen-detected patients is defined as "Date of treatment start" (rule 15) minus "Date of first symptom" (rule 10);
b) The Total interval for screen-detected patients is defined as "Date of treatment start" (rule 15) minus "Date of screening" (rule 13).

26. Range of Time intervals
The time intervals (Patient, Primary Care, Diagnosis, Treatment, Total) must be in range 0-1 year.
If > 1 year: set the interval to 365 days
If negative: set the interval to 0.

For each jurisdiction calculate the number of imputations due to:

a) unknown day in a date (given known month and year);
b) very large(>1 year) interval;
c) negative interval.

27. Number of visits
If patient gave multiple answers to the "Number of visits" questions, then use the option with a fewer number of visits.

28. Specialist waiting time interval
If patient gave multiple answers to the ""How long did it take to get an appointment with specialist?" question, then use the option with the shortest time interval.

29. Type of treatment
If patient ticked both "Yes" and "No" as answers to the "Type of treatment (Surgery, Chemotherapy, Radiotherapy)" questions, then choose "Yes" answer.

For peer review only - http://bmjopen.bmj.com/site/about/guidelines.xhtml

30. Health state
If patient gave multiple answers to the "Health state" question, then use the option with a better health condition.

31. Comorbidity
a) If patient ticked both "Yes" and "No" as answers to the "Comorbidity (Heart disease, Stroke, Lung disease, Diabetes)" questions, then choose "Yes" answer;
b) If both patient and PCP report "Comorbidity", then use the PCP Data.

32. Ethnicity
a) If patient didn't report "Ethnicity", then use the information from (in the order of declining priority):
- "Ethnicity_Other_Details";
- "Other main language spoken at home";
- "The main language spoken at home" (only for Victoria);
- "The main language spoken at home is the chief one for this jurisdiction"="Yes" given "Main language spoken at home is other than the main one for this jurisdiction"="No";
b) Consider Ethnicity as unknown, if answers to the "Ethnicity" question are multiple and belong to different categories ('white', 'Asian', 'black', 'other').

33. Education
If patient gave multiple answers to the "Education" question, then use the option with a higher level of education.

34. Smoking Current
a) If patient ticked both "Yes" and "No" as answers to the "Smoking Current" question, then use "Yes" answer;
b) If patient hasn't ticked neither "Yes" nor "No, then consider this case as Unknown.

35. Smoking Number
If patient reports "SmokingNumber" as text, then re-code using following rules:
a) Where there is a number smoked /day – accept number;
b) Where a range has been given – take the upper value;
c) Where patient has put 10+ or 20+ - capture this as 11 or 21;
d) Where number of cigarettes smoked in the past and currently being smoked are provided - average the numbers;
e) Non entries code as "." ;
f) Non-smokers (eg, "nil", "N/A") are coded as "0".

36. Smoked ever
a) If patient ticked both "Yes" and "No" as answers to the "Smoking ever" question, then use "Yes" answer;
b) If patient hasn't ticked neither "Yes" nor "No": consider it as "Yes", if patient is a current smoker ("Smoking_Current="Yes"") or has specified a number of cigarettes ("SmokingNumber">0). Otherwise consider this case as Unknown.
c) If patient has ticked "No": recode it to "Yes", if patient is a current smoker ("Smoking_Current="Yes").

For peer review only

37. Nature of referral
    a) Review free-text for "Nature of referral" (PCP Data) and re-code, if possible;
    b) In the case of multiple responses, use a single option as (in the order of declining priority):

        - "Referral for immediate admission";
        - "Urgent referral";
        - "Less urgent referral";
        - "General referral" ;
        - "No referral";
        - "Other".

38. Refer Public or Private
    a) If PCP ticked both "Public" and "Private" as answers to the "Refer Public or Private" question, then use "Private" answer;
    b) If PCP hasn't ticked neither "Public" nor "Private", then consider this case as Unknown.

39. Type of referral
    If specialist gave multiple responses to the "How was the patient referred…" question, then use a single option (in the order of declining priority):
    - "Screening";
    - "Respiratory clinic";
    - "General surgery clinic";
    - "General gynaecology";
    - "Specialist/consultant";
    - "PCP";
    - "Other".

40. First Attendance Place
    If specialist gave multiple responses to the "First Attendance Place" question, then consider this case as Unknown.

41. Stage-TNM
    a) If specialist gave multiple responses to the "Stage_TNM" question, then use the highest category;
    b) If registry gave multiple responses to the "Stage_TNM", then use a single option (in the order of declining priority):
        - stage at time of diagnosis
        - stage at surgery
        - stage at oncology
    c) If "Stage_TNM" is reported by both the specialist and registry, then use the registry data.

For peer review only

Supplementary File 5 - Tables

Table 1: Classification of symptoms reported by patients into cancer specific and non-cancer specific

Cancer specific symptoms

1 persistent cough
2 chest infection that wouldn't get better
3 coughing up blood-stained phlegm (sputum)
4 breathlessness
5 chest or shoulder pain
6 a hoarse voice
7 difficulty swallowing
8 swelling of lymph nodes (glands) in my neck
9 ends of fingers becoming larger
10 breast and axilla swelling (in men)

Non-cancer specific symptoms

1 a dull ache or sharp pain when I coughed or took a deep breath
2 pain/discomfort under my ribs
3 face swelling
4 blood clots (thrombosis)
5 pins and needles or numbness in fingers
6 weight loss
7 felt sick/vomiting/nausea/loss of appetite
8 fatigue
9 muscle weakness
10 drowsiness, weakness, dizziness or confusion
11 high temperature (fever) of 38°C (100.4°F)
12 other

For peer review only - http://bmjopen.bmj.com/site/about/guidelines.xhtml

Table 2: Time intervals (days) depicted as median (50th), 75th and 90th percentiles for each of the ten jurisdictions. In Sweden, no data was available on primary care interval

Intervals	Centiles	Wales	Denmark	Sweden	England	N Ireland	Scotland	Manitoba	Ontario	Norway	Victoria
Patient Interval	Number	181	233	172	213	179	169	133	205	55	141
	Median	21	14	21	17	18	21	25	22	19	14
	75th centile	61	53	61	65	60	60	67	61	60	60
	90th centile	216	180	214	205	240	267	180	187	277	180
Primary Care interval	Number	110	159	n/a	147	124	119	80	75	19	89
	Median	20	7		11	13	16	30	29	7	10
	75th centile	43	20		31	51	35	75	73	41	36
	90th centile	91	64		73	112	90	138	183	102	99
Diagnostic interval	Number	176	229	165	212	170	173	138	212	52	160
	Median	45	35	28	54	65	42	87	57	51	54
	75th centile	108	67	83	100	122	106	147	122	109	106
	90th centile	162	162	143	161	281	198	265	331	160	240
Treatment interval	Number	192	279	190	238	200	187	182	263	87	199
	Median	43	16	34	22	32	42	19	47	24	0
	75th centile	64	25	59	41	48	62	56	70	44	22
	90th centile	89	37	77	56	72	90	97	96	72	41
Total interval	Number	147	192	147	176	157	143	117	178	52	113
	Median	116	67	107	114	105	117	127	130	79	78
	75th centile	204	116	190	183	227	253	216	216	186	195
	90th centile	365	210	329	323	365	365	365	339	271	355

For peer review only

For peer review only - http://bmjopen.bmj.com/site/about/guidelines.xhtml

Supplementary File 6 – ICBP M4 Working and Academic Reference Groups

Section 1 – ICBP Module 4 Working Group:

Alina Zalounina Falborg, Research Unit for General Practice, Department of Public Health, Aarhus University, Bartholins Allé 2, 8000 Aarhus C, Denmark

Andriana Barisic, Department of Prevention and Cancer Control, Cancer Care Ontario, 620 University Avenue, Toronto, Ontario, M5G 2L7, Canada

Anna Gavin, Northern Ireland Cancer Registry, Centre for Public Health, Queen's University Belfast, Mulhouse Building, Mulhouse Road, Belfast, BT12 6DP, United Kingdom

Anne Kari Knudsen, European Palliative Care Research Centre (PRC), Department of Oncology, Oslo University Hospital and Institute of Clinical Medicine, University of Oslo, Oslo, N-0424 Oslo, Norway

Breann Hawryluk, Department of Patient Navigation, Cancer Care Manitoba, 675 McDermot Street, Winnipeg, Manitoba, MB R3E 0V9, Canada

Chantelle Anandan, Centre for Population Health Sciences, University of Edinburgh, Doorway 1, Medical Quad Teviot Place, Edinburgh, EH8 9DX, United Kingdom

Conan Donnelly, Centre for Public Health, Queen's University Belfast, Mulhouse Building, Mulhouse Road, Belfast, BT12 6DP, United Kingdom

David H Brewster, Scottish Cancer Registry, Information Services Division, NHS National Services Scotland, Gyle Square, 1 South Gyle Crescent, Edinburgh, EH12 9EB, United Kingdom; Centre for Population Health Sciences, University of Edinburgh, Teviot Place, Edinburgh, EH8 9DX, United Kingdom

David Weller, Centre for Population Health Sciences, University of Edinburgh, Doorway 1, Medical Quad Teviot Place, Edinburgh, EH8 9DX, United Kingdom

Donna Turner, Population Oncology, Cancer Care Manitoba, 675 McDermot Street, Winnipeg, Manitoba, MB R3E 0V9, Canada

Elizabeth Harland, Department of Epidemiology and Cancer Registry, CancerCare Manitoba, 675 McDermot Street, Winnipeg, MB R3E 0V9, Manitoba

Eva Grunfeld, Knowledge Translation Research Network Health Services Research Program, Ontario Institute for Cancer Research; Professor and Vice Chair Research Department of Family and Community Medicine, University of Toronto, 500 University Avenue, Toronto, Ontario, M5G 1V7, Canada

Evangelia Ourania Fourkala, Gynaecological Cancer Research Centre, Women's Cancer, Institute for Women's Health, University College London, London, WC1E 6BT, United Kingdom

For peer review only - http://bmjopen.bmj.com/site/about/guidelines.xhtml

Henry Jensen, Research Unit for General Practice, Department of Public Health, Aarhus University, Bartholins Allé 2, 8000 Aarhus C, Denmark

Irene Reguilon, International Cancer Benchmarking Partnership, Cancer Research UK, London, EC1V 4AD, United Kingdom

Jackie Boylan, Centre for Public Health, Queen's University Belfast, Mulhouse Building, Mulhouse Road, Belfast, BT12 6DP, United Kingdom

Jacqueline Kelly, Northern Ireland Cancer Registry, Centre for Public Health, Queen's University Belfast, Mulhouse Building, Mulhouse Road, Belfast, BT12 6DP, United Kingdom

Jatinderpal Kalsi, Gynaecological Cancer Research Centre, Women's Cancer, Institute for Women's Health, University College London, London, WC1E 6BT, United Kingdom

John Butler, The Royal Marsden, Fulham Road, London, SW3 6JJ, United Kingdom

Kerry Moore, Centre for Public Health, Queen's University Belfast, Mulhouse Building, Mulhouse Road, Belfast, BT12 6DP, United Kingdom

Maria Rejmyr Davis, Southern Sweden Regional Cancer Center, Medicon Village, Scheelevägen 8, building 404, 223 81 Lund, Sweden

Martin Malmberg, Department of Oncology, Lund University Hospital, SE-221 85 Lund, Sweden

Mats Lambe, Regional Cancer Center Uppsala and Department of Medical Epidemiology and Biostatics, Karolinska Institutet, SE-171 77 Stockholm, Sweden

Oliver Bucher, Department of Epidemiology and Cancer Registry, CancerCare Manitoba, 675 McDermot Street, Winnipeg, MB R3E 0V9, Manitoba

Peter Vedsted, Research Unit for General Practice, Department of Public Health, Aarhus University, Bartholins Allé 2, 8000 Aarhus C, Denmark

Rebecca-Jane Law, North Wales Centre for Primary Care Research, Bangor University, Cambrian House 2, Wrexham Technology Park, Wrexham, LL13 7YP, United Kingdom

Rebecca Bergin, Centre for Behavioural Research in Cancer, 615 St Kilda Rd, Melbourne, Victoria, 3004, Australia; Department of General Practice, University of Melbourne, 200 Berkeley St, Victoria, 3053, Australia

Richard D Neal, North Wales Centre for Primary Care Research, Bangor University, Cambrian House 2, Wrexham Technology Park, Wrexham, LL13 7YP, United Kingdom; Academic Unit of Primary Care, Leeds Institute of Health Sciences, University of Leeds, Leeds, LS2 9NL, United Kingdom

For peer review only

Samantha Harrison, Early Diagnosis and International Cancer Benchmarking Partnership, Policy and Information, Cancer Research UK, London, EC1V 4AD, United Kingdom

Sigrun Saur Almberg, Department of Cancer Research and Molecular Medicine, Faculty of Medicine, Norwegian University of Science and Technology (NTNU), N-7491 Trondheim, Norway

Therese Kearney, Northern Ireland Cancer Registry, Centre for Public Health, Queen's University Belfast, Mulhouse Building, Mulhouse Road, Belfast, BT12 6DP, United Kingdom

Victoria Cairnduff, Northern Ireland Cancer Registry, Centre for Public Health, Queen's University Belfast, Mulhouse Building, Mulhouse Road, Belfast, BT12 6DP, United Kingdom

Victoria Hammersley, Centre for Population Health Sciences, University of Edinburgh, Doorway 1, Medical Quad Teviot Place, Edinburgh, EH8 9DX, United Kingdom

Victoria White, Centre for Behavioural Research in Cancer, Cancer Council Victoria, 615 St Kilda Road, Melbourne, Victoria, 3004, Australia; School of Psychology Deakin University, Geelong, Victoria, 3217, Australia

Usha Menon, Gynaecological Cancer Research Centre, Women's Cancer, Institute for Women's Health, University College London, London, WC1E 6BT, United Kingdom

Yulan Lin, European Palliative Care Research Centre (PRC), Department of Oncology, Oslo University Hospital and Institute of Clinical Medicine, University of Oslo, Oslo, N-0424 Oslo, Norway

Section 2 – ICBP Module 4 Academic Reference Group:

Prof Jan Willem Coebergh, Professor of Cancer Surveillance, Department of Public Health, Erasmus Universiteit Rotterdam, Rotterdam, the Netherlands

Jon Emery, Professor of Primary Care Cancer Research, University of Melbourne and Clinical Professor of General Practice, University of Western Australia, Australia

Dr Stefan Bergström, Senior consultant oncologist, Department of Oncology, Gävle, Sweden

Dr Monique E van Leerdam, Erasmus MC University Medical Centre, the Netherlands

Prof Marie-Louise Essink-Bot, Academic Medical Centre, Amsterdam University, the Netherlands

Prof Una MacLeod, Senior Lecturer in General Practice and Primary Care, Hull-York Medical School, United Kingdom

For peer review only - http://bmjopen.bmj.com/site/about/guidelines.xhtml

The RECORD statement – checklist of items, extended from the STROBE statement, that should be reported in observational studies using routinely collected health data.

	Item No.	STROBE items	Location in manuscript where items are reported	RECORD items	Location in manuscript where items are reported
**Title and abstract**					
	1	(a) Indicate the study's design with a commonly used term in the title or the abstract (b) Provide in the abstract an informative and balanced summary of what was done and what was found	Title and abstract – p 1 and 3	RECORD 1.1: The type of data used should be specified in the title or abstract. When possible, the name of the databases used should be included.<br><br>RECORD 1.2: If applicable, the geographic region and timeframe within which the study took place should be reported in the title or abstract.<br><br>RECORD 1.3: If linkage between databases was conducted for the study, this should be clearly stated in the title or abstract.	Abstract – p 3<br><br>Abstract – p 3<br><br>N/A
**Introduction**					
Background rationale	2	Explain the scientific background and rationale for the investigation being reported	Introduction – p 6		
Objectives	3	State specific objectives, including any prespecified hypotheses	Introduction – p 6		
**Methods**					
Study Design	4	Present key elements of study design early in the paper	Methods – p 6		
Setting	5	Describe the setting, locations, and relevant dates, including periods of recruitment, exposure, follow-up, and data collection	Methods – p 6-8		
Participants	6	*(a) Cohort study* - Give the eligibility criteria, and the	Methods – p 6 – and as reference to	RECORD 6.1: The methods of study population selection (such as codes or	Provided as reference to

For peer review only

		sources and methods of selection of participants. Describe methods of follow-up<br>*Case-control study* - Give the eligibility criteria, and the sources and methods of case ascertainment and control selection. Give the rationale for the choice of cases and controls<br>*Cross-sectional study* - Give the eligibility criteria, and the sources and methods of selection of participants<br><br>*(b) Cohort study* - For matched studies, give matching criteria and number of exposed and unexposed<br>*Case-control study* - For matched studies, give matching criteria and the number of controls per case	previous paper.<br><br>N/A	algorithms used to identify subjects) should be listed in detail. If this is not possible, an explanation should be provided.<br><br>RECORD 6.2: Any validation studies of the codes or algorithms used to select the population should be referenced. If validation was conducted for this study and not published elsewhere, detailed methods and results should be provided.<br><br>RECORD 6.3: If the study involved linkage of databases, consider use of a flow diagram or other graphical display to demonstrate the data linkage process, including the number of individuals with linked data at each stage.	previous paper.<br><br>Provided as appendix and in reference to previous paper.<br><br>N/A
Variables	7	Clearly define all outcomes, exposures, predictors, potential confounders, and effect modifiers. Give diagnostic criteria, if applicable.	Methods – p 6-9 – and as reference to previous paper.	RECORD 7.1: A complete list of codes and algorithms used to classify exposures, outcomes, confounders, and effect modifiers should be provided. If these cannot be reported, an explanation should be provided.	N/A
Data sources/ measurement	8	For each variable of interest, give sources of data and details of methods of assessment (measurement).<br>Describe comparability of assessment methods if there is more than one group	Methods – p 6-9 – and as reference to previous paper.		
Bias	9	Describe any efforts to address potential sources of bias	Methods – p 7-8, discussion – p 24-25 – and as reference to		

For peer review only - http://bmjopen.bmj.com/site/about/guidelines.xhtml

			previous paper.		
Study size	10	Explain how the study size was arrived at	Methods – p 6 – and as reference to previous paper.		
Quantitative variables	11	Explain how quantitative variables were handled in the analyses. If applicable, describe which groupings were chosen, and why	Methods – p 6-8 – and as reference to previous paper.		
Statistical methods	12	(a) Describe all statistical methods, including those used to control for confounding<br>(b) Describe any methods used to examine subgroups and interactions<br>(c) Explain how missing data were addressed<br>(d) *Cohort study* - If applicable, explain how loss to follow-up was addressed<br>*Case-control study* - If applicable, explain how matching of cases and controls was addressed<br>*Cross-sectional study* - If applicable, describe analytical methods taking account of sampling strategy<br>(e) Describe any sensitivity analyses	Methods – p 8 – and as reference to previous paper.		
Data access and cleaning methods		..		RECORD 12.1: Authors should describe the extent to which the investigators had access to the database population used to create the study population.	N/A

For peer review only

				RECORD 12.2: Authors should provide information on the data cleaning methods used in the study.	Methods – p 7
Linkage		..		RECORD 12.3: State whether the study included person-level, institutional-level, or other data linkage across two or more databases. The methods of linkage and methods of linkage quality evaluation should be provided.	N/A
**Results**					
Participants	13	(a) Report the numbers of individuals at each stage of the study (*e.g.*, numbers potentially eligible, examined for eligibility, confirmed eligible, included in the study, completing follow-up, and analysed)<br>(b) Give reasons for non-participation at each stage.<br>(c) Consider use of a flow diagram	Results and as table – p 10-11. Flow diagram in previous paper, referenced.	RECORD 13.1: Describe in detail the selection of the persons included in the study (*i.e.,* study population selection) including filtering based on data quality, data availability and linkage. The selection of included persons can be described in the text and/or by means of the study flow diagram.	Results – p 10-15 - and as table. Flow diagram in previous paper, referenced.
Descriptive data	14	(a) Give characteristics of study participants (*e.g.*, demographic, clinical, social) and information on exposures and potential confounders<br>(b) Indicate the number of participants with missing data for each variable of interest<br>(c) *Cohort study* - summarise follow-up time (*e.g.*, average and total amount)	Results and as table – p 10-15.		
Outcome data	15	*Cohort study* - Report numbers of outcome events or summary measures over time<br>*Case-control study* - Report numbers in each exposure category, or summary measures	Results and as table – p 16-23.		

For peer review only

		of exposure<br>*Cross-sectional study* - Report numbers of outcome events or summary measures			
Main results	16	(a) Give unadjusted estimates and, if applicable, confounder-adjusted estimates and their precision (e.g., 95% confidence interval). Make clear which confounders were adjusted for and why they were included<br>(b) Report category boundaries when continuous variables were categorized<br>(c) If relevant, consider translating estimates of relative risk into absolute risk for a meaningful time period	Results and as table – p 21-23.		
Other analyses	17	Report other analyses done—e.g., analyses of subgroups and interactions, and sensitivity analyses	Results – p 23.		
**Discussion**					
Key results	18	Summarise key results with reference to study objectives	Discussion – p 24		
Limitations	19	Discuss limitations of the study, taking into account sources of potential bias or imprecision. Discuss both direction and magnitude of any potential bias	Discussion – p24-25	RECORD 19.1: Discuss the implications of using data that were not created or collected to answer the specific research question(s). Include discussion of misclassification bias, unmeasured confounding, missing data, and changing eligibility over time, as they pertain to the study being reported.	Discussion – p24-25
Interpretation	20	Give a cautious overall interpretation of results considering objectives, limitations, multiplicity of analyses, results from similar	Discussion – p25-28		

For peer review only

		studies, and other relevant evidence			
Generalisability	21	Discuss the generalisability (external validity) of the study results	Discussion, conclusion – p25-29.		
**Other Information**					
Funding	22	Give the source of funding and the role of the funders for the present study and, if applicable, for the original study on which the present article is based	Funding statement – p 31		
Accessibility of protocol, raw data, and programming code		..		RECORD 22.1: Authors should provide information on how to access any supplemental information such as the study protocol, raw data, or programming code.	Availability of data and material statement – p 31

*Reference: Benchimol EI, Smeeth L, Guttmann A, Harron K, Moher D, Petersen I, Sørensen HT, von Elm E, Langan SM, the RECORD Working Committee. The REporting of studies Conducted using Observational Routinely-collected health Data (RECORD) Statement. *PLoS Medicine* 2015; in press.

*Checklist is protected under Creative Commons Attribution (CC BY) license.

For peer review only

BMJ Open

Time intervals and routes to diagnosis for lung cancer in ten jurisdictions: cross-sectional study findings from the International Cancer Benchmarking Partnership (ICBP)

Journal:	*BMJ Open*
Manuscript ID	bmjopen-2018-025895.R1
Article Type:	Research
Date Submitted by the Author:	02-May-2019

Complete List of Authors:	Menon, Usha; University College London, Institute for Women's Health<br>Vedsted, Peter; Aarhus University, Research Unit for General Practice<br>Zalounina Falborg, Alina; Aarhus University, Research Unit for General Practice<br>Jensen, Henry; Aarhus University, Research Unit for General Practice<br>Harrison, Samantha; Cancer Research UK, Policy & information<br>Reguilon, Irene; Cancer Research UK, Policy & information<br>Barisic, Andriana; Cancer Care Ontario<br>Bergin, Rebecca; Cancer Council Victoria, Centre for Behavioural Research in Cancer; University of Melbourne, Department of General Practice<br>Brewster, David; Edinburgh University, Centre for Population Health Sciences; NHS Scotland National Services Division, Scottish Cancer Registry, Information Services Division<br>Butler, John; The Royal Marsden<br>Brustugun, Odd Terje; Olso University Hospital, Institute for Cancer Research<br>Bucher, Oliver; CancerCare Manitoba, Department of Epidemiology and Cancer Registry<br>Cairnduff, Victoria; Queen's University Belfast, Northern Ireland Cancer Registry<br>Gavin, Anna; Queen's University Belfast, Northern Ireland Cancer Registry<br>Grunfeld, Eva; Ontario Institute for Cancer Research, Health Services Research Program<br>Harland, Elizabeth; CancerCare Manitoba, Department of Epidemiology and Cancer Registry<br>Kalsi, Jatinderpal; University College London, Institute for Women's Health<br>Knudsen, Anne Kari; Olso University Hospital, European Palliative Care Research Centre (PRC); University of Oslo, Institute of Clinical Medicine<br>Lambe, Mats; Karolinska Institutet, Department of Medical Epidemiology; University Hospital, Regional Oncologic Center<br>Law, Rebecca-Jane; Bangor University, North Wales Centre for Primary Care Research<br>Lin, Yulan; University of Oslo, Institute of Clinical Medicine; Olso University Hospital, European Palliative Care Research Centre (PRC)<br>Malmberg, Martin; Skanes universitetssjukhus Lund<br>Turner, Donna; CancerCare Manitoba

	Neal, Richard; Bangor University, North Wales Centre for Primary Care Research; University of Leeds, Academic Unit of Primary Care<br>White, Victoria; Cancer Council Victoria, Centre for Behavioural Research in Cancer; Deakin University, School of Psychology<br>Weller, David; Edinburgh University, Centre for Population Health Sciences
**Primary Subject Heading**:	Epidemiology
Secondary Subject Heading:	Global health
Keywords:	EPIDEMIOLOGY, International health services < HEALTH SERVICES ADMINISTRATION & MANAGEMENT, Organisation of health services < HEALTH SERVICES ADMINISTRATION & MANAGEMENT, Respiratory tract tumours < ONCOLOGY, lung cancer, routes to diagnosis, time intervals, international health systems, symptomatic presentation

Time intervals and routes to diagnosis for lung cancer in ten jurisdictions: cross-sectional study findings from the International Cancer Benchmarking Partnership (ICBP)

1. Usha Menon*, MRC Clinical Trials Unit at UCL, Institute of Clinical Trials & Methodology, , University College London, London, United Kingdom
2. Peter Vedsted*, Research Unit for General Practice, Aarhus University, Aarhus, Denmark
3. Alina Zalounina Falborg, Research Unit for General Practice, Aarhus University, Aarhus, Denmark
4. Henry Jensen, Research Unit for General Practice, Aarhus University, Aarhus, Denmark
5. Samantha Harrison, Policy and Information, Cancer Research UK, London, United Kingdom
6. Irene Reguilon, Policy and Information, Cancer Research UK, London, United Kingdom
7. Andriana Barisic, Ontario Renal Network, Cancer Care Ontario, Toronto, Canada
8. Rebecca J Bergin, Centre for Behavioural Research in Cancer, Cancer Council Victoria, Victoria, Australia; Department of General Practice, University of Melbourne, Victoria, Australia
9. David H Brewster, Scottish Cancer Registry, Information Services Division, NHS National Services Scotland, Edinburgh, United Kingdom; Centre for Population Health Sciences, University of Edinburgh, Edinburgh, United Kingdom
10. John Butler, The Royal Marsden, Fulham Road, London, United Kingdom
11. Odd Terje Brustugun, Institute for Cancer Research, Department of Cancer Genetics, Oslo University Hospital, Oslo, Norway
12. Oliver Bucher, Department of Epidemiology and Cancer Registry, CancerCare Manitoba, Manitoba
13. Victoria Cairnduff, Northern Ireland Cancer Registry, Centre for Public Health, Queen's University Belfast, Mulhouse Building, Mulhouse Road, Belfast, United Kingdom
14. Anna T Gavin, Northern Ireland Cancer Registry, Queen's University Belfast, Belfast, United Kingdom

For peer review only

15. Eva Grunfeld, Health Services Research Program, Ontario Institute for Cancer Research, Toronto, Canada

16. Elizabeth Harland, Department of Epidemiology and Cancer Registry, CancerCare Manitoba, Winnipeg, Canada

17. Jatinderpal Kalsi, Women's Cancer, Institute for Women's Health, University College London, London, United Kingdom

18. Anne Kari Knudsen, European Palliative Care Research Centre (PRC), Department of Oncology, Oslo University Hospital and Institute of Clinical Medicine, University of Oslo, Oslo, Norway

19. Mats Lambe, Regional Cancer Center Uppsala and Department of Medical Epidemiology and Biostatics, Karolinska Institutet, Stockholm, Sweden

20. Rebecca-Jane Law, North Wales Centre for Primary Care Research, Bangor University, Wrexham, United Kingdom

21. Yulan Lin, European Palliative Care Research Centre (PRC), Department of Oncology, Oslo University Hospital and Institute of Clinical Medicine, University of Oslo, Oslo, Norway

22. Martin Malmberg, Department of Oncology, Lund University Hospital, Lund, Sweden

23. Donna Turner, Population Oncology, Cancer Care Manitoba, Winnipeg, Canada

24. Richard D Neal, North Wales Centre for Primary Care Research, Bangor University, Wrexham, United Kingdom; Academic Unit of Primary Care, University of Leeds, Leeds, United Kingdom

25. Victoria White, Centre for Behavioral Research in Cancer, Cancer Council Victoria, Victoria, Australia; School of Psychology Deakin University, Geelong, Victoria, 3217, Australia

26. David Weller*, Centre for Population Health Sciences, University of Edinburgh, Edinburgh, United Kingdom

On behalf of the ICBP Module 4 Working Group

* equal contribution

For peer review only

Corresponding author:

Professor Usha Menon

MRC CTU at UCL

Institute of Clinical Trials and Methodology

90 High Holborn, 2nd Floor, London WC1V 6LJ

Email: u.menon@ucl.ac.uk

Word count: 3074; Tables: 6 ; Figures: 2 ; Appendix A-D

For peer review only

Abstract

Objective

Differences in time intervals to diagnosis and treatment between jurisdictions may contribute to previously reported differences in stage at diagnosis and survival. The International Cancer Benchmarking Partnership Module 4 (ICBP M4) reports the first international comparison of routes to diagnosis and time intervals from symptom onset until treatment start for lung cancer patients.

Design

Newly diagnosed lung cancer patients, their primary care physicians (PCP) and cancer treatment specialists (CTS) were surveyed in Victoria (Australia), Manitoba and Ontario (Canada), Northern Ireland, England, Scotland and Wales (UK), Denmark, Norway and Sweden. Using Wales as the reference jurisdiction, the 50th, 75th and 90th percentiles for intervals were compared using quantile regression adjusted for age, gender and comorbidity.

Participants

Consecutive newly diagnosed lung cancer patients, aged >40 years, diagnosed between October 2012 and March 2015 were identified through cancer registries. Of 10,203 eligible symptomatic patients contacted, 2,631 (27.5%) responded and 2,143 (21.0%) were included in the analysis. Data was also available from 1,211 (56.6%) of their PCPs and 643 (37.0%) of their CTS.

Primary and secondary outcome measures:

Interval lengths (days, primary), routes to diagnosis, symptoms (secondary).

Results

With the exception of Denmark (-49 days), in all other jurisdictions the median adjusted total interval from symptom onset to treatment, for respondents diagnosed in 2012-15, was similar to that of Wales (116 days). Denmark had shorter median adjusted primary care interval (-11 days) than Wales (20 days); Sweden had shorter (-20) and Manitoba longer (+40) median adjusted diagnostic intervals compared to Wales (45 days). Denmark (-13), Manitoba (-11), England (-9) and Northern Ireland (-4) had shorter median adjusted treatment intervals than Wales (43 days). The differences were greater for the 10% of patients who waited the longest. Based on overall trends, jurisdictions could be grouped into those with trends of reduced,

For peer review only - http://bmjopen.bmj.com/site/about/guidelines.xhtml

longer and similar intervals to Wales.The proportion of patients diagnosed following presentation to the PCP ranged from 35-75%.

Conclusion

There are differences between jurisdictions in interval to treatment, which are magnified in lung cancer patients who wait the longest. The data could help jurisdictions develop more focused lung cancer policy and targeted clinical initiatives. Future analysis will explore if these differences in intervals impact on stage or survival.

Key words:

lung cancer, routes to diagnosis, time intervals, international health systems, symptomatic presentation

For peer review only

Strengths and limitations of this study

- This is the first study to use standardized survey methods and definitions to systematically examine key intervals from patients first noticing symptoms or bodily changes until the start of treatment for lung cancer across multiple jurisdictions
- Recall bias was minimised by the triangulation of different data sources and by patients completing the questionnaire within a limited time window (median 5 months) after the cancer diagnosis
- A key limitation, as with all questionnaire-based studies was selection and non-response bias which varied across jurisdictions
- Recruitment of patients up to 9 rather than 6 months after diagnosis might have magnified selection bias due to high mortality in lung cancer but a sensitivity analysis suggests that this did not impact on the results.
- The comparisons for Norway and Victoria, are limited by small sample size and inclusion of only surgical patients, respectively.

For peer review only

For peer review only - http://bmjopen.bmj.com/site/about/guidelines.xhtml

Introduction

Lung cancer is the most common cancer worldwide, with nearly 1.83 million cases diagnosed in 2012, and is the leading cause of cancer death globally, accounting for 19% of cancer deaths.[1] Survival is typically low, with 5-year survival in Europe,North America and Australia <20%.[2-3] A key factor is diagnosis at advanced stage. Reasons for this are multi-faceted and include delays due to the atypical nature of some presenting symptoms, poor sensitivity of chest X-rays and physicians not acting quickly enough.[4] Within European countries, differences of 12 and 5 percentage points in 1- and 5-year relative survival, respectively, have been reported for lung cancers diagnosed between 1999-2007.[5] This and other international comparisons raises the possibility of additional contributory factors such as variations in referral patterns, access to diagnostic tests and delays in treatment.[6]

One way of addressing this is to chart the patient journey from first noticing symptoms to treatment start. Many national studies using different methodologies have reported on time intervals to treatment of lung cancer and there are reviews that have looked at international timeframe comparisons [7-10].[11-24] However, as far as we are aware there is no study that has undertaken international comparisons of timeliness across multiple countries using the same methodology.

The International Cancer Benchmarking Partnership (ICBP) was established to explore differences in cancer outcomes and their causes in countries with comparable wealth and universal access to healthcare.[25] We report results from Module 4 (ICBP M4) on differences in time intervals and routes to diagnosis in symptomatic lung cancer patients from ten jurisdictions in Australia, Canada, Denmark, Norway, Sweden and the United Kingdom.

Methods

Methods have been previously detailed.[26] In brief, in each of the ten participating jurisdictions (Victoria (Australia), Manitoba, Ontario (Canada), Denmark, Norway, Sweden Northern Ireland, England, Scotland and Wales), consecutive patients aged ≥40 years, newly diagnosed with malignant lung or bronchus cancer (ICD-10:C34.0-C34.9; ICD-O-3 behaviour code /3) were identified by the cancer registry using validated methods (hospital episode, cancer registration, pathology). Exclusion criteria included previous lung or synchronous cancers. Patients with a previous non-lung primary cancer were eligible. Target recruitment was 200 symptomatic patients per jurisdiction.

Following a vital status check, cancer registries posted the patient questionnaire (Appendix A1) either 1) to the relevant primary care physician (PCP) who then forwarded the pre-

For peer review only - http://bmjopen.bmj.com/site/about/guidelines.xhtml

addressed envelope to the patient after confirmation that the person was aware of the diagnosis and not deemed too sick/anxious to participate in the survey. (Wales, England, Scotland) or 2) to the patient directly or via the research team (remaining seven jurisdictions). In an attempt to decrease attrition and recall bias, the protocol initially specified that all patient questionnaires should be completed within 6 months of diagnosis. As there were administrative delays in cancer notification, this was extended to 9 months.

On receipt of a completed patient questionnaire, in all jurisdictions except Sweden, the relevant PCP and cancer treatment specialist (CTS) were sent questionnaires (Appendix A.2 and A.3). Specialists provided information on diagnosis and start date of treatment. The latter was collected directly from registry records in Northern Ireland and clinical databases in Denmark. Manitoba did not provide specialist data. Date of diagnosis and stage was also collected where possible through cancer registries. Information on the types of treatment (surgery, chemotherapy, radiotherapy and other) were obtained from the patient survey.

Data handling

Data were recoded centrally to ensure that the same explicit rules were applied throughout. Patients in whom age, date of diagnosis or consent were missing were excluded from analyses. Rules were used to combine data from the different sources in a standardised way that ensured reproducibility and transparency (Appendix B). The rules employed a 'hierarchy' principle in terms of the order in which different data sources were used and included imputation rules based on the available data. The exact rule was guided by the measure in question – for example, patient interval was collected primarily from the patient questionnaire whereas primary care time-points from the PCP questionnaire. We applied rules for outliers and implausible measures (e.g. negative time intervals were recorded to zero-days and intervals longer than a year to 365 days).

Routes to diagnosis and symptoms prompting physician visit

These were derived from patient and PCP responses. Symptoms were coded by two PCP authors (DW and PV) into 'lung cancer specific' or 'other' (Appendix C1).

Time intervals

Time intervals were derived using the checklist for the Aarhus Statement.[27] The following time-points were used to calculate the corresponding time intervals (Figure 1):

- first noticing symptoms
- first presentation to health care
- first referral to secondary care

For peer review only - http://bmjopen.bmj.com/site/about/guidelines.xhtml

- diagnosis date
- start of curative or palliative treatment

FIGURE 1

All time-points were validated if there were obvious inconsistencies and negative intervals were set to 0 days. All intervals were truncated at 365 days. Missing data were imputed based on specific rules to ensure that the direction of a possible misclassification bias was known.

Demographics

Health status was measured using the self-reported general health item from short form 36 health survey (SF36).[28] Comorbidity was defined as one of four patient or PCP-reported diseases (heart or lung disease, stroke or diabetes) and categorised into: 'none', 'medium' (one or two) or 'high' (three or four). Level of educational was categorised as 'low' (vocational school or lower) and 'high' (university). Stage data (tumour, node, metastasis - TNM - classification) was grouped as I, II, III, IV or missing.[29]

Statistical analysis

Patient characteristics across jurisdictions were compared using Kruskal-Wallis test for continuous and ordinal data. For nominal data we used Pearson's chi-squared test and Fisher's exact test (if more than 20% of expected cell counts were less than 5 or at least one expected cell count was 0). The differences in intervals between the jurisdictions were estimated using quantile regression, as this method allows for a comparison across the whole distribution of length of the interval.[30] As we were interested in a measure of central tendency of length of the interval and in long and very long intervals, the focus of the study was on the 50^{th}(median), 75th and 90th interval percentiles. Wales was chosen as the reference jurisdiction as it had the lowest lung cancer survival in ICBP Module 1 analysis.[10] Since the length of the interval in days is a continuous measure which has been rounded, we applied the quantile regression analysis on the smoothed quantiles; the method based on the smoothed quantiles is recommended for analyses of discrete (count) data [31]. In STATA this method is implemented in the 'qcount' procedure.[32] Parameters were calculated with 1000 jittered samples. For all interval analyses, the differences in intervals were calculated as marginal effects after quantile regression by setting the continuous covariate (age) to their mean values and the categorical covariates (sex and comorbidity) to their modes. Significance level was set to $\leq$0.05 with 95% confidence intervals (95%CI) calculated where appropriate. Statistical analyses were carried out using STATA v14.

For peer review only - http://bmjopen.bmj.com/site/about/guidelines.xhtml

Sensitivity and validity analyses

All analyses were repeated using only (1) those who fulfilled the 6-month cut-off criteria for interval from diagnosis to questionnaire completion and (2) patient data. The effect of excluding patients for whom at least one interval was missing was investigated.We also repeated the analysis after omitting time intervals which were negative or over 365 days. Agreement between the different data sources (registry, patient, PCP (except in Sweden) and CTS (except in Sweden and Manitoba) was measured by Lin's concordance correlation coefficient (CCC).[33]

Patient involvement

The research questions for this survey drew on an extensive literature relating to diagnosis and treatment delays leading to negative patient experiences. While patient experience was not a primary outcome measure for this study, patients were given the opportunity to comment on their experience through questionnaire free-text response options (under separate analysis). Patients were involved in the piloting of study instruments to ascertain if recruitment and questionnaire content and dissemination strategies were appropriate, described elsewhere.[26] Each jurisdiction has committed to communicating the findings and local implications of this study to organisations representing their study participants.

Results

Of 14,583 lung cancer patients, diagnosed between October 2012 and March 2015 who were alive when identified, 70% (10,203/14,583) were contacted (Table 1). Of 4380 not contacted, 3367 (77%) were from England, Wales and Scotland. Major reasons reported by the PCP for not forwarding the survey included patients being terminally ill, not aware of cancer diagnosis at the time of request, having cognitive or visual impairment, language / communication difficulties, no longer at the address, not wishing to take part in research and a handful not having the index cancer. In addition patents identified were not contacted in England as the target recruitment had been exceeded. For the non-UK jurisdictions, the main reasons for not contacting patients were the patient having died or no longer at the address.

2,631 (27.5% of contacted, 18% of eligible) completed the patient questionnaire at a median of 5 months (range: 0.1,9) after diagnosis (Table 2). The response rate of contacted patients varied from 11.1% (146/1318) in Norway to 61.8% (333/539) in Denmark. Responding patients were more likely to be aged 60-79 years with less advanced stage (Table 1) and alive one year post diagnosis (data not shown). Of the 2,631 responses, 2,143 (81.5%) were included in the analyses which equates to 14.7% (2,143/14,583) of eligible patients. The key reason respondents were excluded were local oversampling (43.9%; 214/488) for additional analyses

For peer review only - http://bmjopen.bmj.com/site/about/guidelines.xhtml

(Table 1). In Victoria, the registry was only able to contact patients who had undergone surgery while the sample size in Norway was limited (n=88) due to delays in securing appropriate approvals.

For peer review only

For peer review only - http://bmjopen.bmj.com/site/about/guidelines.xhtml

Table 1: Cohort for all ten jurisdictions and overall

Jurisdiction	Patients approached via PCP						Patient approached directly by registries/research teams														Total	
	Wales		England		Scotland		N Ireland		Denmark		Manitoba		Ontario		Sweden		Norway		Victoria			
	n	(%)	n	(%)	n	(%)	n	(%)	n	(%)	n	(%)	n	(%)	n	(%)	n	(%)	n	(%)	n	(%)
**Eligible patients** [a, b]	**1,811**	**(100)**	**2,517**	**(100)**	**1,366**	**(100)**	**620**	**(100)**	**539**	**(100)**	**980**	**(100)**	**4,080**	**(100)**	**493**	**(100)**	**1,318**	**(100)**	**859**	**(100)**	**14,583**	**(100)**
**Packs sent to PCP** [c, d]	**1,811**	**(99.7)**	**1,759**	**(69.9)**	**1,137**	**(83.2)**															**4,707**	**(82.7)**
pack not forwarded by PCP	547	(30.1)	255	(14.5)	201	(17.7)															1,003	(21.3)
unsure if pack forwarded by PCP	531	(29.2)	559	(31.8)	234	(20.6)															1,324	(28.1)
**Patients contacted by PCP** [c, d]	**733**	**(40.4)**	**945**	**(53.7)**	**702**	**(61.7)**															**2,380**	**(50.6)**
**Patients approached directly** [c]							**614**	**(99.0)**	**539**	**(100)**	**745**	**(76.0)**	**3,687**	**(90.4)**	**493**	**(100)**	**1,200**	**(91)**	**545**	**(63.4)**	**7,823**	**(88)**
patient died							6	(1.0)	0	(0.0)	103	(13.8)	249	(6.8)	0	(0.0)	0	(0.0)	0	(0.0)	358	(4.6)
no address							0	(0.0)	0	(0.0)	9	(1.2)	255	(6.9)	0	(0.0)	0	(0.0)	0	(0.0)	264	(3.4)
Other							0	(0.0)	0	(0.0)	6	(0.8)	215	(5.8)	0	(0.0)	0	(0.0)	0	(0.0)	221	(2.8)
**Patient responses (% of eligible patients)**[c]	**223**	**(12.3)**	**261**	**(10.4)**	**235**	**(17.2)**	**226**	**(36.5)**	**333**	**(61.8)**	**205**	**(20.9)**	**572**	**(14.0)**	**217**	**(44)**	**146**	**(11.1)**	**213**	**(24.8)**	**2,631**	**(18)**
**Patient responses (% of contacted)**[e]	**223**	**(30.4)**	**261**	**(27.6)**	**235**	**(33.5)**	**226**	**(37.2)**	**333**	**(61.8)**	**205**	**(32.7)**	**572**	**(19.3)**	**217**	**(44)**	**146**	**(12.2)**	**213**	**(39.1)**	**2,631**	**(27.5)**
extra sample for local purpose	0	(0.0)	0	(0.0)	0	(0.0)	0	(0.0)	0	(0.0)	0	(0.0)	214	(37.4)	0	(0.0)	0	(0.0)	0	(0.0)	214	(8.1)
other	0	(0.0)	0	(0.0)	35	(14.9)	25	(11.1)	38	(11.4)	0	(0.0)	43	(7.5)	0	(0.0)	0	(0.0)	3	(1.4)	144	(5.5)
**Patient surveys submitted for analyses** [f]	**223**	**(100)**	**261**	**(100)**	**200**	**(85.1)**	**201**	**(88.9)**	**295**	**(88.6)**	**205**	**(100)**	**315**	**(55.1)**	**217**	**(100)**	**146**	**(100)**	**210**	**(98.6)**	**2,273**	**(86.4)**
excluded for analyses – total	12	(5.4)	9	(3.4)	2	(1.0)	1	(0.5)	10	(3.4)	3	(1.5)	27	(8.6)	6	(2.8)	58	(39.7)	2	(1.0)	130	(5.7)
- previous cancer	0	(0.0)	5	(1.9)	0	(0.0)	0	(0.0)	0	(0.0)	0	(0.0)	3	(1.0)	0	(0.0)	0	(0.0)	0	(0.0)	8	(0.4)
- unknown date of consent or diagnosis	0	(0.0)	0	(0.0)	0	(0.0)	0	(0.0)	5	(1.7)	0	(0.0)	1	(0.3)	6	(2.8)	4	(2.7)	0	(0.0)	16	(0.7)
- consent too late/too early	12	(5.4)	4	(1.5)	2	(1.0)	1	(0.5)	5	(1.7)	3	(1.5)	22	(7.0)	0	(0.0)	33	(22.6)	2	(1.0)	84	(3.7)
- other	0	(0.0)	0	(0.0)	0	(0.0)	0	(0.0)	0	(0.0)	0	(0.0)	1	(0.3)	0	(0.0)	21	(14.4)	0	(0.0	22	(1.0)
**Patients included in analyses** [i] **(% of forwarded surveys)**	**211**	**(94.6)**	**252**	**(96.6)**	**198**	**(99.0)**	**200**	**(99.5)**	**285**	**(96.6)**	**202**	**(98.5)**	**288**	**(91.4)**	**211**	**(97.2)**	**88**	**(60.3)**	**208**	**(99.0)**	**2,143**	**(94.3)**[h]
**PCP surveys** [j] **(% of analysed patients)**	**133**	**(63.0)**	**196**	**(77.8)**	**149**	**(75.3)**	**181**	**(90.5)**	**218**	**(76.5)**	**109**	**(54.0)**	**93**	**(32.5)**	**n/a** [h]		**27**	**(30.7)**	**105**	**(50.5)**	**1,211**	**(56.6)**[i]
**Specialist surveys** [k] **(% of analysed patients)**	**98**	**(46.4)**	**153**	**(60.7)**	**106**	**(53.5)**	**n/a** [g]		**149** [g]	**(52.3)**	**n/a** [h]		**62**	**(21.7)**	**n/a** [h]		**20**	**(22.7)**	**55**	**(26.4)**	**643**	**(37.0)**[m]

[a]Eligible as per protocol: individual aged 40 years or more, with cancer of the lung or bronchus (ICD-10 code: C34.0-C34.9; behaviour code ICD-O 3) but no synchronous primary cancer or prior history of lung cancer, alive at identification who completed consent to participate within nine months of diagnosis. [b]In some jurisdictions some 'eligible' patients had pre-opted out from being contacted and a small number where PCP information was not available. [c]Percentages of eligible patients. [d]Maximum of potentially contacted patients. i.e. sum of packs forwarded by PCP and packs unsure if forwarded by PCP. [e]Percentages of patients contacted by PCP (see note d) for Wales, England and Scotland or percentages of patients contacted directly by a registry; excl. non-accessible patients due to death or no patient addresses (all other jurisdictions). [f] Percentages of patient responses. [g] Data obtained from registries instead in N Ireland and Denmark. [h] Data not collected in this jurisdiction. [i] Denominator = total number of forwarded cases excl. patients not included in analytic sample in Ontario. [j] Denominator r = total number of analysed cases excl. patients from Sweden. [k] Denominator = total number of analysed cases excl. patients from Sweden. Manitoba & N Ireland

For peer review only

Baseline characteristics

Patient characteristics are detailed in Table 2. The cohort was predominantly White (95%), median age 70 years (IQR 64,75) with 82% reporting ‘low’ levels of education. Norway provided stage data classified as local, regional and distant which could not be converted to TNM stage data.

Ontario was the only jurisdiction with more female (65%) than male respondents. While self-reported health differed significantly, with Welsh patients (9%) reporting twice as high ‘poor health’ rates than English or Swedish (4%) and eight-fold that of Manitoba (2%), there was no difference in self-reported comorbidity rates. Even after the exclusion of Victoria, there was significant variation in early Stage (I/II) disease which ranged from 25% (Wales) to 59% (Ontario) and surgical resection rates which varied from 27% (Wales) to 58% (Ontario) (Table 2).

For peer review only

Table 2. The characteristics of eligible patients by jurisdictions and overall

	Wales (N=211)	England (N=252)	Scotland (N=198)	N Ireland (N=200)	Denmark (N=285)	Manitoba (N=202)	Ontario (N=288)	Sweden (N=211)	Norway (N=88)	Victoria (N=208)	Overall (N=2143)	p-value[6]
Date of diagnosis of first patient	03/04/2013	28/01/2013	26/04/2013	22/01/2013	16/05/2013	10/10/2012	08/10/2 013	01/10/2013	16/01/2014	08/01/2013	10/10/2012	
Date of diagnosis of last patient	25/09/2014	14/08/2013	04/12/2013	02/12/2014	13/11/2013	27/03/2015	01/10/2014	30/05/2014	21/01/2015	28/12/2014	27/03/2015	
Interval from diagnosis date of first patient to last patient in months (recruitment period)	18	7	7	23	6	30	12	8	12	24	30	
Median (range) interval diagnosis to questionnaire completion in months	5 (0.6,9)	5 (3,9)	5 (1,9)	4 (0.1,9)	5 (2,8)	6 (4,9)	6 (4,9)	4 (3,8)	7 (5,9)	5 (0.2,8)	5 (0.1,9)	
**Age** years Median (IQR)	71 (65, 77)	71 (65, 77)	70 (64, 76)	69 (62, 75)	70 (63, 74)	70 (63, 77)	70 (64, 75)	70 (63, 75)	69 (64, 73)	68 (63, 73)	70 (64, 75)	0.010[1]
**Sex** n(%) Male	127(60)	134(53)	100(51)	105(53)	151(53)	100(50)	131(45)	111(53)	46(52)	112(54)	1117(52)	0.159[2]
**Health State** n(%) Good	135(64)	176(70)	131(66)	126(63)	190(67)	156(77)	220(76)	152(72)	50(57)	163(78)	1499(70)	<0.001*[1] <0.001**[2]
Fair	55(26)	59(23)	52(26)	51(26)	62(22)	41(20)	47(16)	49(23)	32(36)	33(16)	481(22)	
Poor	20(9)	11(4)	14(7)	16(8)	18(6)	4(2)	16(6)	9(4)	6(7)	10(5)	124(6)	
Missing	1(0.5)	6(2)	1(0,5)	7(4)	15(5)	1(0.5)	5(2)	1(0.5)	0(0)	2(1)	39(2)	
**Comorbidity**[3] n(%) No	92(44)	113(45)	98(49)	82(41)	111(39)	101(50)	136(47)	114(54)	35(40)	98(47)	980(46)	0.029*[1] 0.032**[2]
Medium	111(53)	129(51)	92(46)	103(52)	157(55)	93(46)	132(46)	87(41)	48(55)	100(48)	1052(49)	
High	7(3)	9(4)	8(4)	15(8)	11(4)	6(3)	18(6)	6(3)	5(6)	8(4)	93(4)	
Missing	1(0.5)	1(0.4)	0(0)	0(0)	6(2)	2(1)	2(0.7)	4(2)	0(0)	2(1)	18(0.8)	
**Education** n(%) Low	172(82)	217(86)	174(88)	166(83)	232(81)	170(84)	224(78)	159(75)	65(74)	181(87)	1760(82)	<0.001*[1] <0.001**[2]
High	11(5)	15(6)	11(6)	17(9)	15(5)	21(10)	55(19)	48(23)	14(16)	24(12)	231(11)	

For peer review only

For peer review only - http://bmjopen.bmj.com/site/about/guidelines.xhtml

	Wales (N=211)	England (N=252)	Scotland (N=198)	N Ireland (N=200)	Denmark (N=285)	Manitoba (N=202)	Ontario (N=288)	Sweden (N=211)	Norway (N=88)	Victoria (N=208)	Overall (N=2143)	p-value[6]
Missing	28(13)	20(8)	13(7)	17(9)	38(13)	11(5)	9(3)	4(2)	9(10)	3(1)	152(7)	
**Ethnicity** n(%) White	205(97)	249(99)	197(99)	196(98)	267(94)	173(86)	261(91)	n/a	87(99)	199(96)	1834(95)	<0.001*[2] <0.001**[2]
Asian	1(0.5)	0(0)	1(0.5)	0(0)	0(0)	12(6)	21(7)	n/a	1(1)	6(3)	41(2)	
Black	0(0)	1(0.4)	0(0)	0(0)	1(0.4)	0(0)	1(0.3)	n/a	0(0)	0(0)	3(0.2)	
Other	0(0)	0(0)	0(0)	0(0)	0(0)	15(7)	2(0.7)	n/a	0(0)	0(0)	17(0.9)	
Missing	5(2)	2(0.8)	0(0)	4(2)	17(6)	2(1)	3(1)	n/a	0(0)	3(1)	36(2)	
**Smoking** n(%) Never	13(6)	18(7)	12(6)	19(10)	15(5)	22(11)	31(11)	41(19)	11(13)	33(16)	215(10)	<0.001*[2] <0.001**[2]
Currently	19(9)	28811)	26(13)	41(21)	58(20)	24(12)	29(10)	29(14)	11(13)	9(4)	274(13)	
In the past	174(82)	204(81)	160(81)	137(69)	205(72)	156(77)	225(78)	139(66)	66(75)	165(79)	1631(76)	
Missing	5(2)	2(0.8)	0(0)	3(2)	7(2)	0(0)	3(1)	2(0.9)	0(0)	1(0.5)	23(1)	
**Tumour stage – TNM** n(%) I	26(12)	68(27)	49(25)	42(21)	74(26)	83(41)	133(46)	59(28)	3(3)	124(60)	661(31)	<0.001*[1] <0.001**[2]
II	27(13)	36(14)	32(16)	33(17)	26(9)	19(9)	38(13)	11(5)	5(6)	47(23)	274(13)	
III	64(30)	57(23)	56(28)	59(30)	84(29)	48(24)	54(19)	40(19)	5(6)	19(9)	486(23)	
IV	62(29)	80(32)	50(25)	62(31)	94(33)	48(24)	54(19)	94(45)	3(3)	13(6)	560(26)	
Missing	32(15)	11(4)	11(6)	4(2)	7(2)	4(2)	9(3)	7(3)	72(82)[4]	5(2)	162(8)	
**Tumour stage – TNM**[5] n(%) I/II	53(25)	104(41)	81(41)	75(38)	100(35)	102(51)	171(59)	70(33)	8(9)	*171(82)*	764(39)	<0.001*[2,5] <0.001**[2,5]
III/IV	126(60)	137(54)	106(54)	121(61)	178(62)	96(48)	108(38)	134(64)	8(9)	32(15)	1014(52)	
Missing	32(15)	11(4)	11(6)	4(2)	7(2)	4(2)	9(3)	7(3)	72(82)	5(2)	157(8)	
**Treatment Surgery** n(%) Yes	57(27)	107(42)	84(42)	65(33)	81(28)	113(56)	168(58)	65(31)	36(41)	199(96)	975(45)	<0.001*[2,5] <0.001**[2,5]
No	61(29)	56(22)	55(28)	94(47)	90(32)	44(22)	111(39)	79(37)	28(32)	5(2)	623(29)	
Missing	93(44)	89(35)	59(30)	41(21)	114(40)	45(22)	9(3)	67(32)	24(27)	4(2)	545(25)	
**Treatment Chemo** n(%) Yes	105(50)	125(50)	98(49)	93(47)	159(56)	93(46)	107(37)	133(63)	50(57)	63(30)	1026(48)	<0.001*[2,5] <0.001**[2,5]
No	41(19)	51(20)	46(23)	65(33)	45(16)	62(31)	172(60)	37(18)	18(20)	137(66)	674(32)	
Missing	65(31)	76(30)	54(27)	42(21)	81(28)	47(23)	9(3)	41(19)	20(23)	8(4)	443(21)	
**Treatment Radio** n(%) Yes	82(39)	68(27)	76(38)	72(36)	114(40)	81(40)	98(34)	70(33)	41(47)	29(14)	731(34)	<0.001*[2,5] <0.001**[2,5]
No	50(24)	72(29)	50(25)	77(39)	69(24)	71(35)	180(63)	69(33)	22(25)	155(75)	815(38)	
Missing	79(37)	112(44)	72(36)	51(26)	102(36)	50(25)	10(3)	72(34)	25(28)	24(12)	597(28)	
**Treatment Other** n(%) Yes	10(5)	14(6)	14(7)	11(6)	30(11)	18(9)	9(3)	0(0)	7(8)	16(8)	129(6)	<0.001*[2,5] <0.001**[2,5]

	Wales (N=211)	England (N=252)	Scotland (N=198)	N Ireland (N=200)	Denmark (N=285)	Manitoba (N=202)	Ontario (N=288)	Sweden (N=211)	Norway (N=88)	Victoria (N=208)	Overall (N=2143)	p-value[6]
No	48(23)	63(25)	45(23)	69(35)	255(89)	3(1)	261(91)	0(0)	2(2)	138(66)	884(41)	
Missing	153(72)	175(69)	139(70)	120(60)	0(0)	181(90)	18(6)	211(100)	79(90)	54(26)	1130(53)	

[1]Differences between jurisdictions were tested by the Kruskal-Wallis test, [2] Differences between jurisdictions were tested by the Pearson's Chi[2] test, [3]Comorbidity coded as none=no reported, medium=1-2 reported and high=3+ reported, [4] This included cases which could not be mapped as they were classified as per Cancer Registry of Norway into local (stage I), regional (stage II-III) and distant (stage IV) [5]Excluding Victoria, [6]Excluding Norway, *Missing category is excluded, **Missing category is included,
Abbreviations: IQR=inter-quartile range.

For peer review only

Routes to diagnosis

Results are detailed in Table 3. Over half (55%) were diagnosed following presentation to the PCP of whom 63% (range: 29% Norway - 82% Wales; data not shown) were urgently referred with a suspicion of cancer, based on the PCP questionnaire.

For peer review only

Table 3. Routes to diagnosis of lung cancer patients for each jurisdiction. All figures are n(%) unless otherwise stated.

	Wales (N=211)	England (N=252)	Scotland (N=198)	N Ireland (N=200)	Denmark (N=285)	Manitoba (N=202)	Ontario (N=288)	Sweden (N=211)	Norway (N=88)	Victoria (N=208)	Total (N=2143)
Symptoms prompting visit to PCP	109(52)	150(60)	128(65)	131(66)	170(60)	101(50)	91(32)	65(31)	35(40)	106(51)	1086(51)
Symptoms prompting emergency (A&E) department visit[1]	11(5)	18(7)	12(6)	22(11)	21(7)	25(12)	39(14)	18(9)	3(3)	6(3)	175(8)
Symptoms prompting visit to PCP and emergency (A&E) department[1]	10(5)	4(2)	7(4)	18(9)	8(3)	15(7)	12(4)	8(4)	5(6)	3(1)	90(4)
Incidental diagnosis in course of investigation/treatment for another problem[2]	68(32)	37(15)	36(18)	19(10)	55(19)	57(28)	116(40)	107(51)	32(36)	90(43)	617(29)
Unknown routes to diagnosis[3]	10(5)	17(7)	11(6)	5(3)	16(6)	4(2)	14(5)	11(5)	8(9)	0(0)	96(5)
Other[4]	3(1)	25(10)	3(2)	4(2)	14(5)	0(0)	16(6)	2(1)	5(6)	3(1)	75(3)
Missing	0(0)	1(0.4)	1(0.5)	1(0.5)	1(0.4)	0(0)	0(0)	0(0)	0(0)	0(0)	4(0.2)

[1] Emergency (A&E) route was ascribed when the patient reported pathway to cancer diagnosis involving going or being taken to the emergency department or the PCP reported that the patient presented to emergency department with or without their involvement [2] this could be by PCP, another doctor or via hospital; [3]includes cases where PCP or patient reported routes to diagnosis as 'Other' or 'Missing' but also reported symptoms or duration of symptoms, or date of first symptom, or waiting time for PCP appointment; [4]includes cases where PCP or patient reported routes to diagnosis as 'Other' and hasn't reported any symptoms or duration of symptoms, or date of first symptom, or waiting time for PCP appointment.

For peer review only

Symptoms prompting visit to physician

The median number of patient-reported symptoms were 2 (IQR 1,3). Across jurisdictions, the most common patient-reported symptoms were persistent cough (39%), breathlessness (37%) and fatigue (27%) although there was significant variation in proportion of patients presenting with individual symptoms (Table 4).

The PCPs reported a median of 1 (IQR 1,2) symptom at first presentation, with the most common being persistent cough (39%). Across jurisdictions, the reporting of other symptoms by the PCP was significantly lower compared to patients, especially fatigue (4%) and weight loss (8%). When the analysis was restricted to the cohort where both patient and PCP had completed the survey, this difference persisted. Unlike patients, there was minimal variation in PCP reporting of symptoms, with significant differences limited to ‘no symptoms’, ‘other symptoms not previously listed’ and weight loss. Overall 64% of symptoms were labelled as ‘cancer specific’ by the study PCPs (Table 4).

For peer review only

Table 4. Symptoms experienced by patients and presenting symptoms noted by PCP for eligible patients. All figures are n(%)

	Wales	England	Scotland	N Ireland	Denmark	Manitoba	Ontario	Sweden	Norway	Victoria	Overall	p[1]
**Symptoms (reported by patient)**	(N=211)	(N=252)	(N=198)	(N=200)	(N=285)	(N=202)	(N=288)	(N=211)	(N=88)	(N=208)	(N=2143)	
persistent cough	113(54)	123(49)	97(49)	83(42)	97(34)	71(35)	125(43)	84(40)	10(11)	39(19)	842(39)	<0.001
breathlessness	109(52)	126(50)	82(41)	73(37)	99(35)	63(31)	119(41)	77(36)	12(14)	24(12)	784(37)	<0.001
fatigue	75(36)	79(31)	64(32)	61(31)	38(13)	55(27)	92(32)	60(28)	17(19)	42(20)	583(27)	<0.001
weight loss	38(18)	39(15)	44(22)	37(19)	41(14)	29(14)	36(13)	34(16)	9(10)	20(10)	327(15)	0.147
felt sick /vomiting /nausea/loss of appetite	42(20)	33(13)	31(16)	24(12)	33(12)	22(11)	45(16)	28(13)	3(3)	20(10)	281(13)	0.132
coughing up blood-stained phlegm (sputum)	35(17)	32(13)	24(12)	31(16)	21(7)	16(8)	27(9)	25(12)	5(6)	23(11)	239(11)	0.014
chest or shoulder pain	23(11)	9(4)	18(9)	24(12)	10(4)	28(14)	43(15)	25(12)	4(5)	22(11)	206(10)	<0.001
other symptoms not listed above	51(24)	80(32)	59(30)	54(27)	63(22)	40(20)	30(10)	75(36)	26(30)	42(20)	520(24)	<0.001
no symptoms	29(14)	24(10)	32(16)	21(11)	63(22)	50(25)	67(23)	36(17)	33(38)	75(36)	430(20)	<0.001
missing	1(0.5)	16(6)	6(3)	17(9)	31(11)	9(4)	4(1)	4(2)	12(14)	1(0.5)	101(5)	<0.001
**Presenting symptom (reported by PCP)**	(N=85)	(N=133)	(N=115)	(N=0)	(N=151)	(N=86)	(N=61)	(N=0)	(N=17)	(N=81)	(N=729)	
persistent cough	33(39)	57(43)	45(39)	n/a	67(44)	24(28)	18(30)	n/a	7(41)	33(41)	284(39)	0.093
breathlessness	17(20)	27(20)	20(17)	n/a	27(18)	11(13)	11(18)	n/a	4(24)	13(16)	130(18)	0.803
fatigue	1(1)	5(4)	3(3)	n/a	9(6)	2(2)	2(3)	n/a	1(6)	3(4)	26(4)	0.539[2]
weight loss	5(6)	9(7)	15(13)	n/a	19(13)	3(3)	2(3)	n/a	0(0)	5(6)	58(8)	0.027
felt sick /vomiting /nausea/loss of appetite	1(1)	1(0.8)	2(2)	n/a	6(4)	2(2)	0(0)	n/a	0(0)	0(0)	12(2)	0.418[2]
coughing up blood-stained phlegm (sputum)	8(9)	10(8)	10(9)	n/a	7(5)	2(2)	3(5)	n/a	0(0)	7(9)	47(6)	0.305
chest or shoulder pain	8(9)	10(8)	15(13)	n/a	15(10)	1(1)	7(12)	n/a	1(6)	4(5)	61(8)	0.08
other symptoms not listed above	24(28)	44(33)	33(29)	n/a	64(42)	15(17)	7(12)	n/a	7(41)	20(25)	214(29)	<0.001

For peer review only

no symptoms	5(6)	6(5)	6(5)	n/a	2(1)	31(36)	12(20)	n/a	0(0)	10(12)	72(10)	<0.001
missing	7(8)	7(5)	11(10)	n/a	16(11)	12(14)	6(10)	n/a	2(12)	10(12)	71(10)	0.392
**Cancer-specificity of symptom presented**												
cancer-specific symptom	62(73)	97(73)	79(69)	n/a	94(62)	36(42)	38(62)	n/a	10(59)	48(59)	464(64)	<0.001
non-specific symptom	11(13)	23(17)	19(17)	n/a	39(26)	7(8)	5(8)	n/a	5(29)	13(16)	122(17)	
no symptoms /missing	12(14)	13(10)	17(15)	n/a	18(12)	43(50)	18(30)	n/a	2(12)	20(25)	143(19)	

[1] Differences between jurisdictions (excluding Victoria and Norway) were tested by the Pearson's Chi^2 test, if nothing else stated.
[2] Differences between jurisdictions (excluding Victoria and Norway) were tested by the Fisher's exact test.

For peer review only

Time intervals

The observed time intervals are shown in Appendix C2 and are summarised in Figure 2. Based on overall trends, jurisdictions could be grouped into those with reduced, longer and similar intervals to Wales (Table 5). It was not possible to interpret variations observed in Norway (small sample size) and Victoria (surgical cohort). In the remaining jurisdictions, there was no difference in the median adjusted patient interval compared to Wales. Denmark had shorter median adjusted primary care interval (-11 days); Sweden had shorter (-20) and Manitoba longer (+40) diagnostic intervals compared to Wales. Denmark (-13), Manitoba (-11), England (-9) and Northern Ireland (-4) had shorter treatment intervals. The median adjusted total interval was shorter only in Denmark (Table 5, Figure 2). The differences were greater for the 90th percentile. The total interval in patients who waited longest (90th percentile) was significantly shorter in two jurisdictions (Denmark -142, England -28 days) compared to Wales.

Table 5. Differences in adjusted intervals (days) between Wales and the other nine jurisdictions for lung cancer patients

Intervals	percentiles	Wales	Denmark	Sweden	England	N Ireland	Scotland	Manitoba	Ontario	Norway	Victoria
		Reference in days	Overall trend - shorter intervals			Similar with some intervals longer, some shorter		Overall trend - longer intervals		Difficult to interpret (see text for reasons)	
Ranking by 5-year survival rates for lung cancers diagnosed in 1999-2007 [5]		10	6	1	9	7	8	3	2	5	4
Patient Interval	Number of patients	181	233	172	213	179	169	133	205	55	141
	50th percentile (95% CI)	21	-6 (-13,0)	-1 (-9,8)	-3 (-10,5)	-3 (-13,6)	-3 (-10,4)	1 (-8,10)	1 (-11,14)	0 (-8,8)	**-9** (-16,-2)
	75th percentile (95% CI)	61	-13 (-38,13)	-2 (-24,21)	-2 (-42,37)	-8 (-55,39)	1 (-25,27)	3 (-23,30)	-7 (-54,39)	-9 (-60,42)	-4 (-46,38)
	90th percentile (95% CI)	216	**-34** (-55,-13)	-1 (-25,23)	-15 (-42,12)	24 (-21,70)	**43** (7,79)	**-35** (-59,-10)	**-34** (-66,-2)	**59** (21,96)	**-35** (-49,-21)
Primary Care interval	Number of patients	110	159	N/A	147	124	119	80	75	19	89
	50th percentile (95% CI)	20	**-11** (-18,-3)	N/A	-7 (-17,3)	-5 (-15,4)	-3 (-14,8)	7 (-8,21)	5 (-9,19)	**-11** (-18,-4)	-8 (-17,1)
	75th percentile (95% CI)	43	**-29** (-47,-12)		-17 (-42,8)	1 (-45,48)	-11 (-36,14)	19 (-47,85)	20 (-72,112)	-10 (-57,37)	-12 (-70,46)
	90th percentile (95% CI)	91	-30 (-66,7)		-39 (-85,6)	17 (-55,90)	-20 (-67,25)	13 (-38,65)	102 (-56,258)	-22 (-109,66)	-19 (-89,51)
Diagnostic interval	Number of patients	176	229	165	212	170	173	138	212	52	160
	50th percentile (95% CI)	45	-12 (-25,1)	**-20** (-35,-5)	9 (-3,21)	17 (-5,39)	-4 (-16,8)	**40** (14,66)	10 (-6,26)	4 (-16,24)	7 (-13,27)
	75th percentile (95% CI)	108	**-45** (-52,-39)	**-22** (-30,-15)	-7 (-20,7)	**12** (2,22)	-15 (-32,1)	**35** (22,48)	5 (-9,19)	-4 (-15,8)	-2 (-8,4)
	90th percentile (95% CI)	162	-27 (-153,99)	-34 (-206,138)	-14 (-100,72)	112 (-165,389)	31 (-81,143)	**112** (32,192)	106 (-122,335)	0 (-93,93)	**62** (10,114)
Treatment interval	Number of patients	192	279	190	238	200	187	182	263	87	199
	50th percentile (95% CI)	43	**-13** (-15,-11)	-2 (-8,3)	**-9** (-12,-5)	**-4** (-7,-2)	0 (-4,4)	**-11** (-17,-5)	3 (-4,10)	**-8** (-11,-6)	**-29** (-32,-27)
	75th percentile (95% CI)	64	**-32** (-36,-28)	-2 (-8,4)	**-18** (-23,-13)	**-13** (-18,-7)	1 (-7,9)	-5 (-16,6)	6 (-2,14)	**-13** (-19,-8)	**-33** (-41,-25)
	90th percentile (95% CI)	89	**-45** (-50,-40)	**-10** (-17,-4)	**-28** (-36,-20)	**-16** (-23,-9)	-6 (-14,1)	4 (-5,13)	4 (-4,13)	**-22** (-30,-14)	**-39** (-45,-32)
Total	Number of patients	147	192	147	176	153	143	117	178	52	113

For peer review only - http://bmjopen.bmj.com/site/about/guidelines.xhtml

interval	50th percentile (95% CI)	116	**-49** (-95,-3)	-8 (-64,47)	-7 (-52,38)	-16 (-41,10)	-2 (-70,66)	11 (-41,63)	9 (-78,97)	**-34** (-56,-12)	-32 (-64,2)
	75th percentile (95% CI)	204	-91 (-270,87)	-17 (-40,7)	-29 (-175,118)	5 (-191,201)	33 (-144,211)	13 (-77,103)	-7 (-331,317)	-39 (-107,29)	-23 (-61,14)
	90th percentile (95% CI)	365	**-142** (-150,-134)	-18 (-59,23)	**-28** (-37,-18)	0 (-4,5)	15 (-26,55)	15 (-26,55)	0 (-78,79)	**-84** (-119,-49)	0 (-3,3)

Interval relative to Wales	Trends	Significant
Reduction		
Increase		

The majority of patients were diagnosed between 2013-14. The differences were calculated for the 50th, 75th and 90th percentiles by setting age to its mean value and gender and comorbidity to their modes (i.e. male gender and medium comorbidity). It is not possible to interpret differences observed for Norway (due to the small sample size) and Victoria (the cohort was limited to those who had undergone surgery).

FIGURE 2

Sensitivity and validity analyses

The estimates of routes to diagnosis, time intervals, and regression analysis trends were not significantly altered by changing the cut-off to 6 months or using only patient data (results not shown).

Appendix C3 details, which sources were used based on the standardization rules, to define dates and also how often a day in the date was imputed. With regards to the dates of first presentation to healthcare (CCC=0.91), diagnosis (CCC $\geq$ 0.93) and treatment (CCC=0.94), there was adequate agreement between all data sources where the data on these dates was collected. Agreement between patient versus PCP for dates of first presentation to healthcare (CCC=0.91) and diagnosis (CCC=0.93) was also adequate as was agreement between patient versus CTS for dates of diagnosis (CCC=0.94) and treatment (CCC=0.94).

Omitting time intervals which were negative or over 365 days (Appendix C4) led to change in direction of difference which was non-significant in long intervals (75th or 90th percentile) between Wales and jurisdictions in four cases: Norway and Victoria (patient interval), N Ireland (diagnostic interval), England (total interval). All other results were similar to the main results.

Discussion

Main findings

This is the first international study we are aware of comparing lung cancer routes and time intervals. With the exception of Denmark, in all other jurisdictions, the median total interval

For peer review only - http://bmjopen.bmj.com/site/about/guidelines.xhtml

from symptom onset to treatment, for respondents diagnosed in 2012-15 was similar to that of Wales, the reference. However, there were jurisdiction specific differences in patient, diagnostic and treatment intervals, especially for the 10% of patients who waited the longest. Based on overall trends, jurisdictions could be grouped into those with trends of reduced, longer and similar intervals to Wales.

Across jurisdictions, all symptoms other than persistent cough were less frequently reported by the PCP when compared to patients. This was especially true for fatigue and weight loss. One in four patients reported incidental diagnosis and one in ten were diagnosed following a visit to the emergency (A&E) department.

Strengths and weaknesses

Our study helps address the shortcomings of current international comparisons across multiple national studies with significant variation in methodology including differences in definition of intervals. Strengths of our study include 1) use of the same methodology across countries 2) use of cancer registries to identify consecutive newly diagnosed patients; 3) use of standardised questionnaires; 4) inclusion of PCP and CTS questionnaires enriched by registry data; 5) minimal data interpretation by the local teams with all data cleaning performed in a standardised manner centrally; and 6) triangulation with comprehensive data rules to ensure validity, consistency and preserve statistical precision.[21] Recall bias was minimised by the triangulation of different data sources and by patients completing the questionnaire within a limited time window (median 5 months) after the cancer diagnosis.

A key limitation, as with all questionnaire-based studies was both selection and non-response bias which varied across jurisdictions and has implications for interpretation and generalisation of findings. In comparing intervals, we adjusted for age, sex and comorbidity but were unable to adjust for ethnicity and education due to different classification systems. Recall bias was minimised by the triangulation of different data sources and by patients completing the questionnaire within a limited time window (median 5 months) after the cancer diagnosis. Recruitment of patients up to 9 rather than 6 months after diagnosis might have magnified the selection bias due to high mortality.[34] However, sensitivity analysis suggests that this did not impact on the results. Categorising presenting symptoms into indicative or not was done pragmatically as existing guidelines for lung cancer investigation vary across ICBP jurisdictions.[35] In Norway and Victoria, a small sample size and restriction of eligibility to only surgical patients, respectively, made comparison difficult. Nonetheless, significant differences in these two jurisdictions compared to Wales were largely limited to the treatment interval alone.

For peer review only - http://bmjopen.bmj.com/site/about/guidelines.xhtml

There was variation in stage distribution across jurisdictions. While this may be partly related to the varying response rate, true differences in lung cancer stage have been noted on analysis of registry data of patients diagnosed between 2004-2007.[6] The high lung cancer mortality and self-selection are likely to have contributed to an over-representation of early stage disease and tumours treated with surgical resection. This suggests that true variation may well be higher than that reported in this cohort of 'healthier early stage' patients.

Comparison to other studies

The most common patient-reported symptoms, in keeping with the literature, were persistent cough, breathlessness, fatigue and weight loss, with one in five reporting 'no symptoms'.[18] Only a minority (11%) of our respondents reported coughing blood or bloody sputum/spit, which is the only consistent predictor of lung cancer.[36] While haemoptysis was reported in a prospective survey (England 2011-12) by 22% of lung cancer patients identified through respiratory clinics, it was a presenting symptom in only 5% of cases.[11]

The median number of symptoms reported by patients was more than that reported by the PCP in all jurisdictions. This was especially so for fatigue and weight loss. A number of factors could have contributed to this - patients not listing all symptoms at presentation, patients having a different understanding/recall of their symptoms post diagnosis, PCPs only recording key symptoms such as cough. Further research on under reporting of systemic symptoms such as fatigue and weight loss is warranted.

As lung cancer mortality is higher in patients attending emergency (A&E) departments, the rates are often compared in an attempt to understand international survival differences.[37] The rate of respondents who attended A&E varied two-fold across jurisdictions from 9-10% in England, Scotland and Denmark to 18-20% in Northern Ireland, Ontario and Manitoba. While rates for Scotland (10%) were similar to that reported in a prospective Scottish audit (11.5%), as were rates for Denmark (7% vs 6.3% when PCP not involved), rates for England (9%) were lower than those reported in population based audits (25%) reflecting non-response bias.[14,15] In Victoria (4%) restriction of the cohort to surgical patients is likely to have accounted for the very low rates.

Our reported median patient, primary care and diagnostic intervals are in keeping with those previously reported from the participating jurisdictions (Table 6). Minor variations in interval estimates are likely due to differences in data source, sample size and cohort characteristics.[38] Longer intervals were reported from earlier cancer cohorts - median

For peer review only - http://bmjopen.bmj.com/site/about/guidelines.xhtml

primary care interval for England of 52 days in 1998-2000 (our median 11),[13] median total interval for Denmark of 108 days in 2004-5 (our median 67) and Norway of 118 in 2002-5 (our median 79).[17-19,24]

For peer review only

Table 6. Summary of literature on intervals in lung cancer patients diagnosed since 2000 in the ICBP Module 4 countries

Study No	Ref	Study Period	Jurisdiction	Design	Patients	No. of lung cancer patients	Interval[1] (days)				Total interval
							Patient	Primary care	Diagnostic	Treatment	
1	Walter FM et al (2015)	2011-2	England, UK	Prospective patient questionnaire survey - multi hospital cohort. Dates of diagnosis based on medical note review.	All attending urgent and routine respiratory clinics across the five hospitals in England aged over 40 years with symptoms suspicious of lung cancer.	153	Interval from first symptom to diagnosis Median 91 (IQR 49-184)				
2	Lyratzopoulos G et al (2015)	2009-10	England, UK	Prospective national audit of cancer diagnosis using primary practice patient records and continuous sampling during audit period	All patients aged ≥15 years who had first presented to a primary care practitioner and were subsequently diagnosed with one of 28 cancers	1128	Median 11 (IQR 0-32)	Median 3 (IQR 14-39)			
3	Neal R et al (2014)	2007–8	UK	Retrospective analysis of electonic health record data from General Practice Research Database - population cohort	All newly diagnosed with one of 15 cancers	2851			Median 112 (IQR 45-251)		
4	Barrett J et al (2008)	1998-2002	Exeter, England, UK	Retrospective case-control review of PCP records - population cohort	All with lung cancer aged ≥ 40 years, identified from the hospital cancer registry and computerised searches of all primary care practices	247		Median 52 (IQR 7–243)			
							Interval from first symptom to diagnosis - Median 121 (IQR 53,261)				
5	Baughan P et al (2009)	2005-6, 2007-8	Scotland, UK	Retrospective audit involving PCP review of medical records of all newly diagnosed cancer patients they had seen - population cohort	All newly diagnosed with lung cancer	981	Median 9.5 (IQR 31)		Median 11 (IQR 28)		
6	Guldbrandt LM et al (2015)	2010	Denmark	Retrospective PCP questionnaires survery of national registry-based population cohort	All consecutive newly diagnosed lung cancer patients	429-442 depending on interval		Median 7 (IQR, 0-30)	Median 29 (IQR, 12-69)		
7	Tørring ML et al (2013)	2004 - 5	Aarhus, Denmark	Prospective, population-based study using electronic health records and PCP survey of identified patients	All newly diagnosed with lung cancer after attending primary care	262			Median 112 (IQR 45-251)		
8	Hansen RP et al (2011)	2004-5	Aarhus, Denmark	Retrospective PCP survey	Cancer patients newly diagnosed during a 1-year period identified using administrative registry data	128-251 (depending on interval)	Median 28 (IQR 7-56)	Median 0 (IQR 0-9)		Median 51 (IQR 27-76)	Median 108 (IQR 82-167)
9	Bjerager M et al (2006)	2003	Aarhus, Denmark	Retrospective PCP survey using structured telephone interviews enriched with administrative registry data - population-based cohort	All lung cancer patients identified through histological and cytological tests from county-based registers	84		Median 32.5 (IQR 12–68)			
10	Rolke HB et al (2006)	2002-5	Norway (South)	Retrospective questionnaire-based patient survey - hospital cohort	All newly diagnosed with lung cancer	273 -376 (depending on interval)	Median 19 (2–77)				Median 118 (IQR 68–220)
11	Stokstad T et al (2017)	2011-13	Norway	Retrospective medical record audit -single hospital cohort	All cases that started diagnostic work-up and were diagnosed with lung cancer at St. Olavs Hospital, Trondheim	449				42 days (range: 2–296)	
12	Largey G et al (2016)	2013	Victoria, Australia	Retrospective medical record audit -three hospital cohort	Admitted with a new diagnosis of lung cancer over a three month period in three hospitals	78				Mean 30.4 (SD 45.3)	
13	Evans SM et al (2016)	2011-4	Victoria, Australia	Retrospective medical record audit - multi hospital cohort	All lung cancer patients newly diagnosed in six public and two private hospitals	1417			Median 15 (IQR, 5–36)	Median 30 (IQR, 6–84)	
14	Emery JD et al (2017)	2012 -4	Western rural Australia	Prospective cluster randomised trial of symptom awareness	Lung cancer patients newly diagnosed in the control arm of the trial	167	Interval from first symptom to diagnosis Median 34.5 (IQR 7 103.5)				

For peer review only

15	Burneister BH et al (2010)	2000-4	Queensland Australia	Retrospective analysis of radiation therapy waiting times	All lung cancer patients who received radiation therapy as initial treatment at a public hospital.	1535					Median 33[2]
16	Ellis PM and Vandermeer R (2011)	2010	Ontario, Canada	Retrospective patient survey using structured telephone interviews - single centre cohort. Appointment dates and diagnostic tests verified through family doctor or patient chart review.	All lung cancer patients referred to a regional cancer centre	52		Median 21	Median 27 (IQR 0-38)		Median 138 (IQR 79-175)
17	Lo DS et al (2007)	2005-7	Ontario, Canada	Retrospective medical record audit - multi hospital cohort	All with lung cancer seen on a newly implemented Time to Treat Program	144			Median interval from suspicion of lung cancer to diagnosis 37		

[1] Intervals as defined in Figure 1; [2] Limited to patients receiving radiation treatment

For peer review only

Across all jurisdictions, there was no significant difference in primary care intervals for the 10% of patients with longest interval. It is likely that these patients had vague or non-specific symptoms and signs. Referral guidelines for suspected lung cancer do not always favour patients with early symptoms and often prioritise those with more advanced disease.[39] Access to better diagnostic tools such as low-dose CT chest in the primary care setting may favour this group of patients.[40] It would be useful in future projects to explore whether such access may have contributed to the improved 1-year lung cancer survival rates reported from Australia and Canada.[6]

Diagnostic intervals were significantly longer for Manitoba compared to other jurisdictions and twice that reported in an ongoing local PCP audit (personal communication). While one might suspect overestimation due to differences in the source of date of first presentation, between our study (in almost half, it was derived from patients) and local audit, this is less likely as the concordance coefficient between PCP and patient derived data at Manitoba was 0.94.

Observed median treatment intervals were below 6 weeks for nearly all jurisdictions. This was the only interval where there were significant differences between jurisdictions with Denmark, England, Norway and Northern Ireland all having shorter adjusted treatment intervals across all percentiles, with larger differences for the 75th and 90th percentile. These improvements may reflect implementation of waiting time targets in Denmark (35-38 days from first consultation depending on treatment modality) and the UK (31 days from decision to treat).[41,42] The shorter treatment intervals in Norway are in keeping with long-standing provision of standardized cancer care pathways and effective coordination between primary care and treatment centers. While a systematic review did not find evidence to support an association between intervals and lung cancer outcomes, increasing mortality with longer diagnostic intervals was noted in a more recent, high-quality study.[16] In 2000, O'Rourke reported median intervals of 94 days (35-187) between the first hospital visit and starting treatment resulting in 21% of potentially curable patients becoming incurable.[43] Others have found metabolic evidence on PET/CT of pre-treatment disease progression in 21% and TNM upstaging in 18% of small-cell lung cancer patients after a relatively short median inter-scan interval of 43 days.[44] Long intervals can also result in deterioration in performance status. More recently, there is concern that the need for genotyping may result in further increase in time to treatment.

The shorter total interval in Denmark likely reflects the significant reductions in cancer waiting times following a collaborative effort to set-up and implement a national centralised quality

For peer review only - http://bmjopen.bmj.com/site/about/guidelines.xhtml

management system, the Danish Cancer Patient Pathways (CPPs). The latter includes PCP access to fast-track diagnostic work-up.[45]. The findings are in keeping with higher relative survival and lower mortality in Denmark among symptomatic cancer patients diagnosed through primary care after the implementation of CPPs and with the accelerated increase in 5-year survival among Danish lung cancer patients diagnosed in 2010-2014 when compared to patients from earlier time periods.[46] While there is some inherent lead-time bias, the findings highlight the importance and feasibility of a timely diagnosis of lung cancer.

Conclusions

The study provides for the first time, robust data, collected through consistent methods in all jurisdictions, allowing for detailed comparisons of key diagnostic intervals in lung cancer and routes to diagnosis. While all jurisdictions except Denmark, had similar median adjusted total intervals, there were jurisdiction-specific significant differences in patient, diagnostic and treatment intervals, especially for the 10% of patients who waited the longest. The proportion of patients diagnosed following presentation to the PCP ranged from 35-75%. These data could help individual jurisdictions to better target their efforts to reduce time to treatment and ultimately improve patient experience and outcomes in lung cancer.'

Intervals and pathways are ultimately of interest as they relate to prognosis. A further analysis which includes all four cancers (lung, ovary, colon and breast) surveyed in ICBP4 module and explores the impact of these intervals on stage and 1-year survival is underway.

List of abbreviations

ICBP M4 – International Cancer Benchmarking Partnership Module 4

PCP – Primary Care Physician

CTS – Cancer Treatment Specialist

CPP – Danish Cancer Patient Pathways

Figure headings

Figure 1: Time intervals from onset of symptoms to start of treatment based on the Aarhus Statement

Figure 2: Differences in 50th, 75th and 90th centiles of the intervals (days) between Wales as the reference and the other nine jurisdictions. The data are adjusted for differences in age, gender and comorbidity. The bars in black show significant differences in intervals.

Acknowledgement

We are grateful to Catherine Foot, Martine Bomb, Brad Groves of Cancer Research UK for managing the programme. The ICBP Module 4 Working Group (below) for their support and work keeping the study going. Christian Finley (Canada), David Baldwin (England) for reviewing the manuscript. Stefan Bergström, Jan Willem Coebergh, Jon Emery, Monique E

For peer review only - http://bmjopen.bmj.com/site/about/guidelines.xhtml

van Leerdam, Marie-Louise Essink-Bot and Una MacLeod (the ICBP M4 Academic Reference Group) for providing independent peer review of the study protocol and analysis plan development. We thank all the patients, primary care physicians, cancer treatment specialists, and registry staff of all jurisdictions who took part in this study, and also the Danish Lung Cancer Registry for providing clinical information for the study.

ICBP Module 4 Working Group

Alina Zalounina Falborg, (Research Unit for General Practice, Department of Public Health, Aarhus University), Andriana Barisic (Department of Prevention and Cancer Control, Cancer Care Ontario) ,Anna Gavin (Northern Ireland Cancer Registry, Centre for Public Health, Queen's University Belfast), Anne Kari Knudsen (European Palliative Care Research Centre (PRC), Department of Oncology, Oslo University Hospital and Institute of Clinical Medicine), Breann Hawryluk (Department of Patient Navigation, Cancer Care Manitoba), Chantelle Anandan (Centre for Population Health Sciences, University of Edinburgh), Conan Donnelly (Centre for Public Health, Queen's University Belfast), David H Brewster, Scottish Cancer Registry, Information Services Division, NHS National Services Scotland , David Weller (Centre for Population Health Sciences, University of Edinburgh), Donna Turner (Population Oncology, Cancer Care Manitoba), Elizabeth Harland (Department of Epidemiology and Cancer Registry, CancerCare Manitoba), Eva Grunfeld (Knowledge Translation Research Network Health Services Research Program, Ontario Institute for Cancer Research; Professor and Vice Chair Research Department of Family and Community Medicine, University of Toronto), Evangelia Ourania Fourkala (Gynaecological Cancer Research Centre, Women's Cancer, Institute for Women's Health, University College London) , Henry Jensen (Research Unit for General Practice, Department of Public Health, Aarhus University), Irene Reguilon (International Cancer Benchmarking Partnership, Cancer Research UK), Jackie Boylan (Centre for Public Health, Queen's University Belfast), Jacqueline Kelly (Northern Ireland Cancer Registry, Centre for Public Health, Queen's University Belfast), Jatinderpal Kalsi (Gynaecological Cancer Research Centre, Women's Cancer, Institute for Women's Health, University College London), John Butler (The Royal Marsden) Kerry Moore, Centre for Public Health, Queen's University Belfast, Martin Malmberg (Department of Oncology, Lund University Hospital), Mats Lambe (Regional Cancer Center Uppsala and Department of Medical Epidemiology and Biostatics, Karolinska Institutet), Oliver Bucher (Department of Epidemiology and Cancer Registry, CancerCare Manitoba), Peter Vedsted, Research Unit for General Practice, Department of Public Health, Aarhus University, Rebecca-Jane Law (North Wales Centre for Primary Care Research, Bangor University), Rebecca Bergin (Centre for Behavioural Research in Cancer; Department of General Practice, University of Melbourne), Richard D Neal (North Wales Centre for Primary Care Research, Bangor University; Academic Unit of Primary Care, Leeds Institute of Health Sciences, University of Leeds), Samantha Harrison, Early Diagnosis and International Cancer Benchmarking Partnership, Policy and Information, Cancer Research UK, Sigrun Saur Almberg (Department of Cancer Research and Molecular Medicine, Faculty of Medicine, Norwegian University of Science and Technology (NTNU) , Therese Kearney (Northern Ireland Cancer Registry, Centre for Public Health, Queen's University Belfast), Victoria Cairnduff (Northern Ireland Cancer Registry, Centre for Public Health, Queen's University Belfast), Victoria Hammersley (Centre for Population Health Sciences, University of Edinburgh), Victoria White (Centre for Behavioural Research in Cancer, Cancer Council Victoria Road; School of Psychology Deakin University), Usha Menon (Gynaecological Cancer Research Centre, Women's Cancer, Institute for Women's Health, University College London), Yulan Lin (European Palliative Care Research Centre (PRC), Department of Oncology, Oslo University Hospital and Institute of Clinical Medicine)

For peer review only - http://bmjopen.bmj.com/site/about/guidelines.xhtml

ICBP Programme Board:

Aileen Keel (Scottish Government, Edinburgh, Scotland); Anna Boltong (Cancer Council Victoria, Carlton, Australia); Anna Gavin (Northern Ireland Cancer Registry, Queens University, Belfast, UK); David Currow (Cancer Institute New South Wales, Sydney, Australia); Gareth Davies (Wales Cancer Network, Cardiff, UK); Gunilla Gunnarsson (Swedish Association of Local Authorities and Regions, Stockholm, Sweden); Heather Bryant (Canadian Partnership Against Cancer, Toronto, Canada); Jane Hanson (Welsh Cancer National Specialist Advisory Group, Cardiff, UK); Kathryn Whitfield (Department of Health, Victoria Australia); Linda Rabeneck (Cancer Care Ontario, Toronto, Canada); Michael A Richards (Care Quality Commission, London, UK); Michael Sherar (Cancer Care Ontario, Toronto, Canada); Nicola Quin (Cancer Council Victoria, Carlton, Australia); Nicole Mittmann (Cancer Care Ontario, Toronto, Canada); Robert Thomas (Department of Health and Human Services, Victoria, Melbourne, Australia); Sara Hiom (Cancer Research UK); Sean Duffy (NHS England, London, UK); Chris Harrison (NHS England, London, UK); Søren Brostrøm (Danish Health and Medicines Authority, Copenhagen, Denmark); Stein Kaasa (University Hospital of Trondheim, Trondheim, Norway).

ICBP Academic Reference Group

Prof Jan Willem Coebergh, Professor of Cancer Surveillance, Department of Public Health, Erasmus Universiteit Rotterdam, Rotterdam, the Netherlands Jon Emery, Professor of Primary Care Cancer Research, University of Melbourne and Clinical Professor of General Practice, University of Western Australia, Australia Dr Stefan Bergström, Senior consultant oncologist, Department of Oncology, Gävle, Sweden Dr Monique E van Leerdam, Erasmus MC University Medical Centre, the Netherlands Prof Marie-Louise Essink-Bot, Academic Medical Centre, Amsterdam University, the Netherlands Prof Una MacLeod, Senior Lecturer in General Practice and Primary Care, Hull-York Medical School, United Kingdom

Author's contribution

UM, DW, PV, AZF, HJ planned the study design, data collection, carried out the analyses and wrote the draft manuscript. UM, PV, DW, HJ, AB, AKK, RJB, DHB, JK, VC, ATG, EG, EH, ML, RJW, YL, MM, DT, RDN, VW, IR and SH were responsible for local data collection (alongside the Working Group), management and interpretation, and have participated in writing and have approving the final manuscript version. JB, OB and OTB provided advice on the interpretation of results in their respective jurisdictions and comments or substantial edits on the manuscript, approving the final version.

Competing Interests

For peer review only - http://bmjopen.bmj.com/site/about/guidelines.xhtml

None

Data Availability statement

No additional data avaliable

Funding:

This work was supported by: CancerCare Manitoba; Cancer Care Ontario; Canadian Partnership Against Cancer (CPAC); Cancer Council Victoria; Cancer Research Wales; Cancer Research UK; Danish Cancer Society; Danish Health and Medicines Authority; European Palliative Care Research Centre (PRC), Norwegian University of Science and Technology (NTNU); Northern Ireland Guidelines Audit and Implementation Network (GAIN); Macmillan Cancer Support; National Cancer Action Team; NHS England; Northern Ireland Cancer Registry, funded by the Public Health Agency NI; Norwegian Directorate of Health; Research Centre for Cancer Diagnosis in Primary Care (CaP), Aarhus University, Denmark; Scottish Government; Swedish Association of Local Authorities and Regions; University College London and NIHR Biomedical Research Centre at University College London NHS Foundation Trust; University of Edinburgh; Victorian Department of Health and Human Services; Welsh Government. The funding bodies had no influence on the design of the study and collection, analysis, and interpretation of data, in writing the manuscript or whether to publish the results.

Availability of data and material

The data that support the findings of this study are available from the named authors from each ICBP jurisdiction, but restrictions apply to the availability of these data and so are not publicly available. Data are however available from the authors upon reasonable request and with permission of the ICBP Programme Board. Please contact the ICBP Programme Management team, based at Cancer Research UK, with any queries (icbp@cancer.org.uk).

Ethics approval and consent to participate

For each local data collection, there were specific procedures and approvals which included anonymised data transfer to University College London and Aarhus University. Approvals were received from the following institutions: Cancer Council Victoria Human Research Ethics Committee [HREC 1125]; Health Research Ethics Board, University of Manitoba [HS15227 (H2012:105)]; Research Resource Ethics Committee, CancerCare Manitoba [RRIC#28-2012]; University of Toronto Research Ethics Board [27881]; The Danish Data Protection Agency [2013-41-2030]; Swedish Ethics Review Board, Uppsala [2013/306]; Norway Regional

For peer review only - http://bmjopen.bmj.com/site/about/guidelines.xhtml

committees for medical and health research ethics [2013/136/REK nord]; England, Wales and Scotland, NRES Committee East Midlands – Derby 2, local R&D for each health board [11/EM/0420]; Northern Ireland ORECNI Ethical approval, local governance for each health Trust [11/EM/0420].

For peer review only

For peer review only - http://bmjopen.bmj.com/site/about/guidelines.xhtml

References

1. Ferlay J, Steliarova-Foucher E, Lortet-Tieulent J, et al. Cancer incidence and mortality patterns in Europe: Estimates for 40 countries in 2012. Eur J Cancer 2013;49(6):1374-403.

2. Allemani C, Weir HK, Carreira H, et al. Global surveillance of cancer survival 1995-2009: analysis of individual data for 25 676 887 patients from 279 population-based registries in 67 countries (CONCORD-2). Lancet 2015;385(9972):977-1010.

3. Australian Institute of Health and Welfare 2012. Cancer survival and prevalence in Australia: period estimates from 1982 to 2010. Cancer Series no. 69. Cat. no. CAN 65. Canberra: AIHW https://www.aihw.gov.au/getmedia/da7bff73-5b78-4cf8-a719-5903a75fcc9a/13953.pdf.aspx?inline=true

4. Neal RD, Robbé IJ, Lewis M, Williamson I, Hanson J. The complexity and difficulty of diagnosing lung cancer: findings from a national primary-care study in Wales. Prim Health Care Res Dev 2015 Sep;16(5):436-49

5. Francisci S, Minicozzi P, Pierannunzio D, et al. Survival patterns in lung and pleural cancer in Europe 1999-2007: Results from the EUROCARE-5 study. Eur J Cancer 2015;51(15):2242-53.

6. Coleman MP, Forman D, Bryant H, et al. Cancer survival in Australia, Canada, Denmark, Norway, Sweden, and the UK, 1995-2007 (the International Cancer Benchmarking Partnership): an analysis of population-based cancer registry data. Lancet 2011;377(9760):127-38.

7. Olsson JK, Schultz EM, Gould MK. Timeliness of care in patients with lung cancer: a systematic review. Thorax. 2009;64(9):749-56

8. Malalasekera A, Nahm, S, Blinman PL, Kao SC, Dhillon H, VardyJ. How long is too long? A Scoping Review of Health System delays in lung cancer. European Respiratory Reviews. 2018; 29;27(149)

9. Jacobsen MM, Silverstein SC, Quinn M, Waterston LB, Thomas CA, Benneyan JC, et al. Timeliness of access to lung cancer diagnosis and treatment: A scoping literature review. Lung Cancer-J Iaslc. 2017;112:156-64.

10. Vinas F, Ben Hassen I, Jabot L, Monnet I, Chouaid C. Delays for diagnosis and treatment of lung cancers: a systematic review. Clin Respir J. 2016;10(3):267-

For peer review only - http://bmjopen.bmj.com/site/about/guidelines.xhtml

11. Walter FM, Rubin G, Bankhead C, et al. Symptoms and other factors associated with time to diagnosis and stage of lung cancer: a prospective cohort study. Br J Cancer 2015;112 Suppl 1:S6-13.

12. Neal RD, Din NU, Hamilton W, et al. Comparison of cancer diagnostic intervals before and after implementation of NICE guidelines: analysis of data from the UK General Practice Research Database. Br J Cancer 2014;110(3):584-92.

13. Barrett J, Hamilton W. Pathways to the diagnosis of lung cancer in the UK: a cohort study. BMC Fam Pract 2008;9:31.

14. Baughan P, O'Neill B, Fletcher E. Auditing the diagnosis of cancer in primary care: the experience in Scotland. Br J Cancer 2009;101 Suppl 2:S87-91.

15. Guldbrandt LM, Fenger-Gron M, Rasmussen TR, et al. The role of general practice in routes to diagnosis of lung cancer in Denmark: a population-based study of general practice involvement, diagnostic activity and diagnostic intervals. BMC Health Serv Res 2015;15:21.

16. Torring ML, Frydenberg M, Hansen RP, et al. Evidence of increasing mortality with longer diagnostic intervals for five common cancers: a cohort study in primary care. Eur J Cancer 2013;49(9):2187-98.

17. Hansen RP, Vedsted P, Sokolowski I, et al. Time intervals from first symptom to treatment of cancer: a cohort study of 2,212 newly diagnosed cancer patients. BMC Health Serv Res 2011;11:284.

18. Bjerager M, Palshof T, Dahl R, et al. Delay in diagnosis of lung cancer in general practice. Br J Gen Pract 2006;56(532):863-8.

19. Rolke HB, Bakke PS, Gallefoss F. Delays in the diagnostic pathways for primary pulmonary carcinoma in Southern Norway. Respir Med 2007;101(6):1251-7.

20. Largey G, Ristevski E, Chambers H, et al. Lung cancer interval times from point of referral to the acute health sector to the start of first treatment. Aust Health Rev 2016.

21. Evans SM, Earnest A, Bower W, et al. Timeliness of lung cancer care in Victoria: a retrospective cohort study. Med J Aust 2016;204(2):75 e1-9.

22. Ellis PM, Vandermeer R. Delays in the diagnosis of lung cancer. J Thorac Dis 2011;3(3):183-8.

For peer review only

23. Lo DS, Zeldin RA, Skrastins R, et al. Time to treat: a system redesign focusing on decreasing the time from suspicion of lung cancer to diagnosis. J Thorac Oncol 2007;2(11):1001-6.

24. Helsper CW, van Erp NF, Peeters PHM, de Wit NJ. Time to diagnosis and treatment for cancer patients in the Netherlands: Room for improvement? European Journal of Cancer 2017; (87) 113-121

25. Butler J, Foot C, Bomb M, et al. The International Cancer Benchmarking Partnership: an international collaboration to inform cancer policy in Australia, Canada, Denmark, Norway, Sweden and the United Kingdom. Health Policy 2013;112(1-2):148-55.

26. Weller D, Vedsted P, Anandan C, et al. An investigation of routes to cancer diagnosis in 10 international jurisdictions, as part of the International Cancer Benchmarking Partnership: survey development and implementation. BMJ Open 2016;6(7):e009641.

27. Weller D, Vedsted P, Rubin G, et al. The Aarhus statement: improving design and reporting of studies on early cancer diagnosis. Br J Cancer 2012;106(7):1262-7.

28. Jenkinson C, Coulter A, Wright L. Short form 36 (SF36) health survey questionnaire: normative data for adults of working age. Bmj. 1993 May 29;306(6890):1437-40

29. TNM classification of malignant tumours / edited by L.H. Sobin, M.K. Gospodarowicz, and Ch. Wittekind. — 7th ed. UICC International Union Against Cancer. ISBN 978-1-4443-3241-4

30. Koenker R, Bassett G. Regression quantiles. Econometrica 1978; 46:33-50

31. Machado JAF, Santos Silva JMC. Quantiles for Counts. J Amer Statistical Assoc 2005, 100(472): 1226-1237.

32. Miranda A. QCOUNT: Stata program to fit quantile regression models for count data. Boston: Boston College Department of Economics; 2006

33. Lin LI. A concordance correlation coefficient to evaluate reproducibility. Biometrics 1989;45(1):255-68.

34. Walters S, Maringe C, Coleman MP, et al. Lung cancer survival and stage at diagnosis in Australia, Canada, Denmark, Norway, Sweden and the UK: a population-based study, 2004-2007. Thorax 2013;68(6):551-64.

35. Nicholson BD, Mant D, Neal RD, et al. International variation in adherence to referral guidelines for suspected cancer: a secondary analysis of survey data. Br J Gen Pract 2016;66(643):e106-13.

For peer review only

36. Shim J(1), Brindle L, Simon M, George S. A systematic review of symptomatic diagnosis of lung cancer. Fam Pract. 2014 Apr;31(2):137-48

37. Hamilton W, Peters TJ, Round A, et al. What are the clinical features of lung cancer before the diagnosis is made? A population based case-control study. Thorax 2005;60(12):1059-65.

38. Beckett P, Tata LJ, Hubbard RB. Risk factors and survival outcome for non-elective referral in non-small cell lung cancer patients-analysis based on the National Lung Cancer Audit. Lung Cancer 2014;83(3):396-400.

39. Lim A, Mesher D, Gentry-Maharaj A, et al. Time to diagnosis of Type I or II invasive epithelial ovarian cancers: a multicentre observational study using patient questionnaire and primary care records. BJOG 2016;123(6):1012-20.

40. Neal RD, Allgar VL, Ali N, et al. Stage, survival and delays in lung, colorectal, prostate and ovarian cancer: comparison between diagnostic routes. Br J Gen Pract 2007;57(536):212-9.

41. Guldbrandt LM, Moller H, Jakobsen E, et al. General practice consultations, diagnostic investigations, and prescriptions in the year preceding a lung cancer diagnosis. Cancer Med 2017;6(1):79-88.

42. Danish National Board of Health. Pakkeforløb for lungekræft [Cancer Patient Pathway – Lung Cancer], 2016) National Board of Health, Copenhagen. Available at: https://www.sst.dk/da/sygdom-og-behandling/kraeft/pakkeforloeb/~/media/89192ECB2709401CAD8E4BBB0304691E.ashx [accessed 25 September 2017]

43. O'Rourke N, Edwards R. Lung cancer treatment waiting times and tumour growth. Clin Oncol (R Coll Radiol). 2000;12(3):141-4.

44. Wang J, Mahasittiwat P, Wong KK, et al. Natural growth and disease progression of non-small cell lung cancer evaluated with 18F-fluorodeoxyglucose PET/CT. Lung Cancer 2012;78(1):51-6.

45. Jensen H, Tørring ML,Vedsted P. Prognostic consequences of implementing cancer patient pathways in Denmark: a comparative cohort study of symptomatic cancer patients in primary care. BMC Cancer 2017;17:627.

46. Allemani C, Matsuda T, Di Carlo V, et al. Global surveillance of trends in cancer survival 2000–14 (CONCORD-3): analysis of individual records for 37 513 025 patients

For peer review only - http://bmjopen.bmj.com/site/about/guidelines.xhtml

diagnosed with one of 18 cancers from 322 population-based registries in 71 countries. Lancet 2018 Mar 17;391(10125):1023-1075.

For peer review only

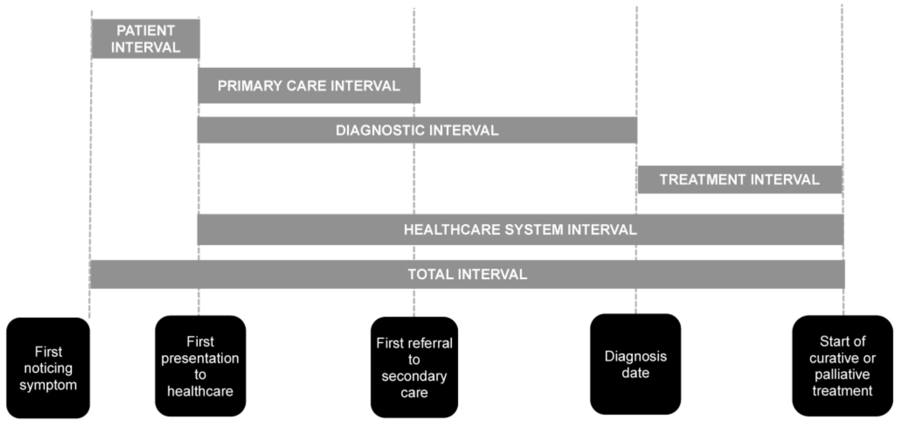

Time intervals from onset of symptoms to start of treatment based on the Aarhus Statement

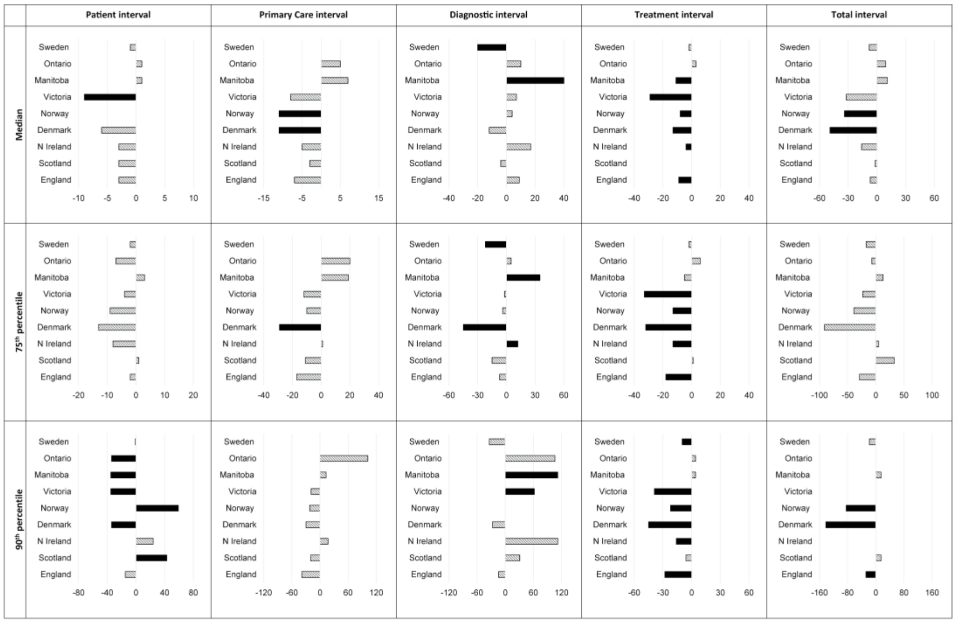

Differences in 50th, 75th and 90th centiles of the intervals (days) between Wales as the reference and the other nine jurisdictions. The data are adjusted for differences in age, gender and comorbidity. The bars in black show significant differences in intervals.

420x297mm (300 x 300 DPI)

Supplementary Web Appendix

Contents

Appendix A. Lung cancer questionnaires

A1: Patient questionnaire…………………………………………………………..................……….2

A2: Primary care physician (PCP) questionnaire……………………………………………...16

A3: Cancer treatment specialists (CTS) questionnaire ……………………………………………24

Appendix B . Rules for missing, incomplete, multiple response and out of range data……………….…32

Appendix C . Supplementary Tables

C1: Classification of lung cancer -specific symptoms reported by patients into cancer specific and non- cancer specific……………………………………………….................................39

C2: Time intervals (days) depicted as median, 75th and 90th centiles for each of the ten jurisdictions. In Sweden, no data was available on primary care interval……………………………40

C3. Data sources used to define dates and percentage of imputed dates…….41

C4: Percentages of negative intervals set to 0, large intervals (>365 days) set to 365 days, and intervals based not on dates………………………………………………………42

For peer review only - http://bmjopen.bmj.com/site/about/guidelines.xhtml

Appendix A: Lung cancer questionnaires

A1: Patient questionnaire

ICBP
International Cancer Benchmarking Partnership

International Cancer Benchmarking Partnership Module 4

Patient questionnaire
Lung Cancer

Thank you very much for taking the time to fill in this questionnaire – it should take about 20 minutes to complete. We are sending the questionnaire to a large sample of people who we understand have had a diagnosis of lung cancer. If this has been sent to you in error and you do not have cancer, please do not continue and return the documents in the prepaid envelope.

Sample

Our aim is to gain a better understanding of the process by which people have their cancer diagnosed. We would also like to find out more about the symptoms they experience (if any), and the pathway they follow from start of symptoms to treatment of their cancer. This will help in identifying ways in which cancers can be diagnosed quickly and effectively. Thank you once again for your time.

This information is confidential and will not be passed to anyone involved in your treatment.

Name: ____________________

Date of Birth: ____________________

Address: ____________________

Copyright property of the International Benchmarking Partnership (ICBP)
and Module 4 collaborators. Usage must be authorised

ICBP
International Cancer Benchmarking Partnership

Consent form

Please read the consent form and sign your name and date **BELOW**.

If you require any clarification, please do not hesitate to ring the study team members. Their contact details are found on the information sheet.

Please be reassured that your responses are completely confidential and will not be passed to anyone involved in your treatment. For the purposes of the study it is important that you agree to consent to all the statements listed below.

- I confirm that I have read the attached information sheet and I understand why the research is being done.
- I am willing for the team to request information from my GP and hospital doctors which is relevant to the audit as described in the information sheet.
- I give permission for my details (name, address) to be given to the cancer registry (NHS Information Centre for Health and Social Care) for follow up.
- I agree for the information I have provided and any other relevant information from my medical records to be stored as described in the information sheet under the custodianship of University College London.
- I consent to sharing of coded data which contains no personal identifiers between researchers, some of whom are located outside the European Union.
- I consent for use of my data if I become mentally incapacitated during the course of the project.

I agree to all the statements listed and consent to participate in the study.

Name (Please print) ______________________________

Signature: ______________________ Date: ______________

If we have any questions, may we phone you for clarification?
(Please tick) ☐ **Yes** ☐ **No**

If **Yes**, please provide your telephone number: ______________________

Sample

Copyright property of the International Benchmarking Partnership (ICBP) and Module 4 collaborators. Usage must be authorised

ICBP
International Cancer Benchmarking Partnership

1. Please can you confirm the details of your GP/GP practice (name, practice address – as best as you can remember): We appreciate that you may have more than one GP involved in your care – in which case, we are interested in the GP you would say provides the majority of your care, particularly relating to the cancer you've had diagnosed.

Name of doctor ____________________

Name of practice ____________________

Address ____________________

____

____

Postcode ____________________

Town ____________________

Sample

Copyright property of the International Benchmarking Partnership (ICBP) and Module 4 collaborators. Usage must be authorised

For peer review only - http://bmjopen.bmj.com/site/about/guidelines.xhtml

2. Which of the following best describes the events which led to your diagnosis of cancer? (please tick only **ONE** answer)

	✓
I had symptoms/I noticed a bodily change and went to see a doctor (e.g. GP)	
I had symptoms/I noticed a bodily change and went/was taken to Accident and Emergency (A&E)	
I had seen a doctor/GP with symptoms, but went/was taken to Accident and Emergency (A&E) when things worsened	
I was being investigated by my doctor(s) for another problem during which time the cancer was discovered	
Other (please describe):	

Sample

Copyright property of the International Benchmarking Partnership (ICBP) and Module 4 collaborators. Usage must be authorised

ICBP
International Cancer Benchmarking Partnership

3. The following health concerns or symptoms are commonly experienced with lung cancer.

Cough
Breathlessness
Being short of breath
Chest pain / Pain on breathing
Coughing up blood
Bloody sputum/blood in spit
Unexplained weight loss
Loss of appetite
Fatigue

Sample

Please write down ALL health concern(s) or symptom(s) you may have had before contacting a doctor or taking part in screening. It does not matter if they are not included in the list above:

Please write your health concern(s) or symptom(s) in the boxes below:
1)
2)
3)
4)
5)
6)

✓

This is not applicable to me (e.g. I did not have any symptoms), please tick	

Copyright property of the International Benchmarking Partnership (ICBP) and Module 4 collaborators. Usage must be authorised

ICBP
International Cancer Benchmarking Partnership

4. Please write down your best estimate of the date you noticed the first of these health concern(s) or symptom(s). If you cannot remember the exact date, you can fill in the month and the year.

Day (optional), month, year

D	D	M	M	Y	Y	Y	Y

	✓
This is not applicable to me (e.g. I had no symptoms), please tick	

5. Approximately how long did you have health concern(s) or symptom(s) before contacting a doctor? (Please think of the first visit to the doctor, not re-visits after that). Please tick only **ONE** answer.

	✓
Less than 1 week	
1-2 weeks	
3-4 weeks	
5-7 weeks	
2-5 months	
6-12 months	
More than 12 months	

Sample

	✓
This is not applicable to me (e.g. I had no symptoms), please tick	

Copyright property of the International Benchmarking Partnership (ICBP) and Module 4 collaborators. Usage must be authorised

ICBP
International Cancer Benchmarking Partnership

6a. Once you contacted a practice about your health concern(s) or symptom(s), how long did it take to get an appointment with a doctor? (Please think of the first visit to the doctor, to discuss your health concern(s) or symptom(s)).
Please tick only **ONE** answer.

Same day/next day	
Within 1 week	
1-2 weeks	
3-4 weeks	
Longer	

If there was no waiting time<br>(e.g. you went/were taken to A&E), please tick this box	

This is not applicable to me (e.g. I had no symptoms), please tick	

Sample

6b. What was the date you first saw your doctor about your health concern(s) or symptom(s)? If you cannot remember the exact date, you can fill in the month and the year.

Day (optional), month, year

D	D	M	M	Y	Y	Y	Y

This is not applicable to me (e.g. I had no symptoms), please tick	

Copyright property of the International Benchmarking Partnership (ICBP) and Module 4 collaborators. Usage must be authorised

ICBP
International Cancer Benchmarking Partnership

7. How many times did you visit the following for the investigation of your symptoms before your cancer was diagnosed?

	Please write down the number of visits
GP	
Hospital	
Consultant/specialist outside of a hospital	

This is not applicable to me (e.g. I had no symptoms), please tick	

8a. After your doctor referred you to a specialist, how long did it take you to get an appointment? Please tick only **ONE** answer.

Sample

Less than 1 week	
1-2 weeks	
3-4 weeks	
5-7 weeks	
2-5 months	
6-12 months	
More than 12 months	

This is not applicable to me (e.g. my doctor did not refer me), please tick	

Copyright property of the International Benchmarking Partnership (ICBP) and Module 4 collaborators. Usage must be authorised

ICBP
International Cancer Benchmarking Partnership

8b. What was the date of your first appointment with a doctor, involved in investigating and/or treating your cancer, to whom you were referred? If you cannot remember the exact date, you can fill in the month and the year.

Day (optional), month, year

D	D	M	M	Y	Y	Y	Y

This is not applicable to me (e.g. my doctor did not refer me), please tick	✓

9. What was the date you were told you had cancer? If you cannot remember the exact date, you can fill in the month and the year.

Day (optional), month, year

D	D	M	M	Y	Y	Y	Y

Sample

Copyright property of the International Benchmarking Partnership (ICBP) and Module 4 collaborators. Usage must be authorised

ICBP
International Cancer Benchmarking Partnership

10. Have you had any of the following treatments for your cancer yet? If so, please can you estimate the date this treatment started? Please tick **ALL** that apply. If you cannot remember the exact date, you can fill in the month and the year.

	Type of treatment		Date of treatment (give first date if you had more than one)
a.	Surgery	☐ **Yes** ☐ **No**	Day (optional), month, year D D M M Y Y Y Y
b.	Chemotherapy	☐ **Yes** ☐ **No**	Day (optional), month, year D D M M Y Y Y Y
c.	Radiotherapy	☐ **Yes** ☐ **No**	Day (optional), month, year D D M M Y Y Y Y
d.	Other Please specify:	☐ **Yes** ☐ **No**	Day (optional), month, year D D M M Y Y Y Y
e.	Treatment not started yet	☐ **Yes**	

Sample

Copyright property of the International Benchmarking Partnership (ICBP) and Module 4 collaborators. Usage must be authorised

ICBP
International Cancer Benchmarking Partnership

11. Who is the consultant doctor who has taken responsibility for diagnosing and or/treating your cancer?

Name of consultant:
Hospital name:
Hospital department:

Please can you answer some more general questions about your health?
It will help us in interpreting your responses to this questionnaire to know about your general health and other health problems you may have had in the past.

12. Looking back to the 2 years before you were diagnosed with cancer, would you say your general health was (Please tick only **ONE** answer.): ✓

Sample

Very good	
Good	
Fair	
Poor	
Very poor	

13. Have you been treated before for any of the conditions below?
Please tick 'yes' or 'no' for each condition:

Heart disease	☐ Yes	☐ No
Stroke	☐ Yes	☐ No
Lung disease (excluding lung cancer)	☐ Yes	☐ No
Diabetes	☐ Yes	☐ No

Copyright property of the International Benchmarking Partnership (ICBP) and Module 4 collaborators. Usage must be authorised

ICBP
International Cancer Benchmarking Partnership

Finally, a little more information about you. The information you provide below will help us to analyse the results of the survey in more detail.

14. Which of these best describes your ethnic group? (please tick one box, as appropriate). If you are descended from more than one ethnic or racial group, please tick the group you consider you belong to, or tick 'any other ethnic group'.

	✓		✓		✓		✓
White		Chinese		Black - Caribbean		Black - African	
Black - other		Indian		Pakistani		Bangladeshi	
Any other ethnic group, please specify:							

Sample

15. What is the main language spoken in your home? Please tick

	✓
English	
Other, please specify:	

16. What is the highest level of education you have achieved?
Please tick only **ONE** answer.

	✓
Finished school at or before the age of fifteen	
Completed GCSEs, O-levels or equivalent	
Completed A Levels or equivalent	
Completed further education but not a degree	
Completed a Bachelor's degree / Masters degree / PhD	
Other, please specify:	

Copyright property of the International Benchmarking Partnership (ICBP) and Module 4 collaborators. Usage must be authorised

ICBP
International Cancer Benchmarking Partnership

17. Have you ever smoked cigarettes, including hand-rolled ones, pipes or cigars?

☐ Yes ☐ No

18. Are you a current smoker, smoking either cigarettes, including hand-rolled ones, pipes or cigars?

☐ Yes ☐ No

19. If you are a current smoker or have smoked in the past, how many cigarettes, including hand-rolled ones, pipes or cigars on average do you smoke/have you smoked per day?

Number per day:	

Sample

Copyright property of the International Benchmarking Partnership (ICBP) and Module 4 collaborators. Usage must be authorised

For peer review only - http://bmjopen.bmj.com/site/about/guidelines.xhtml

ICBP
International Cancer Benchmarking Partnership

20. Further comments

Please add anything else that you would like to tell us about your cancer diagnosis or treatment.

Sample

Thank you very much for taking the time to complete this questionnaire.

Copyright property of the International Benchmarking Partnership (ICBP) and Module 4 collaborators. Usage must be authorised

For peer review only - http://bmjopen.bmj.com/site/about/guidelines.xhtml

A2: Primary care physician (PCP) questionnaire

ICBP
International Cancer Benchmarking Partnership

International Cancer Benchmarking Partnership Module 4

Primary Care Audit
Lung Cancer

Thank you very much for agreeing to fill in this questionnaire. As part of an international study examining differences in cancer survival, we are sending the questionnaire to health care providers of a sample of consented patients with cancer.

Our aim is to gain a better understanding of the process by which people have their cancer diagnosed – the symptoms they experience, and the pathway they follow from onset of symptoms to treatment of their cancer. This will help in identifying ways in which cancers can be diagnosed and treated quickly and effectively. Thank you once again for your time.

Sample

Please can you refer to your patient's notes in completing the questionnaire as this will help in obtaining accurate data on time points.

If you would prefer to return this questionnaire without the patient details, please tear off along the dotted line

ID-number: Jurisdiction-ID + Patient-ID: ______

Copyright property of the International Benchmarking Partnership (ICBP) and Module 4 collaborators. Usage must be authorised

ICBP
International Cancer Benchmarking Partnership

Sample

Patient information

ID-number: Jurisdiction-ID + Patient-ID: ____________________

Full name: ____________________

Address: ____________________

____

Postcode: ____________________

Date of birth: | D | D | M | M | Y | Y | Y | Y |

Copyright property of the International Benchmarking Partnership (ICBP) and Module 4 collaborators. Usage must be authorised

ICBP
International Cancer Benchmarking Partnership

1. Duration of symptoms

Please estimate how long your patient had symptom(s), attributable to lung cancer, before attending your practice (or other health service).

We appreciate that identifying a 'date of first symptom' is not always straightforward – particularly when there are multiple and/or chronic symptoms. Nevertheless, we hope you can provide a 'best estimate'.

**Estimate of symptom duration** (please tick **one**):		**What were the symptoms?** Please describe:
Less than 1 week		
1 to 4 weeks		
5 to 7 weeks		
2-5 months		
6-12 months		
More than 12 months		
Not possible to estimate		
No symptoms (e.g. screen detected cancers)		

Sample

Copyright property of the International Benchmarking Partnership (ICBP) and Module 4 collaborators. Usage must be authorised

ICBP
International Cancer Benchmarking Partnership

2. Pathway of presentation

2.1 Through what route did the patient first present? Please tick **ONE**:

	✓	
Your patient first presented to primary care (either in-hours or out-of-hours)		Please can you provide your best approximation of the date of this **primary care** visit<br>D D M M Y Y Y Y
Your patient presented straight to A&E (with or without your involvement)		
Your patient first presented to primary care, but then at a later date presented to A&E as an emergency (with or without your involvement)		Please can you provide your best approximation of the date of this **primary care** visit<br>D D M M Y Y Y Y
Other – please describe:		

Sample

Copyright property of the International Benchmarking Partnership (ICBP) and Module 4 collaborators. Usage must be authorised

ICBP
International Cancer
Benchmarking Partnership

3. Date you ordered any tests/investigations in response to symptom(s).

We are interested in any kind of tests/investigations (e.g. imaging etc) that you may have ordered. Please only consider the tests/investigations that you ordered yourself. Please tick **all** that apply and put in the date that the test/investigation was ordered:

	✓	
Chest x-ray		D D M M Y Y Y Y
MRI scan		D D M M Y Y Y Y
CT scan		D D M M Y Y Y Y
PET scan		D D M M Y Y Y Y
Sputum test		D D M M Y Y Y Y
Lung biopsy		D D M M Y Y Y Y
Bronchoscopy		D D M M Y Y Y Y
Other (please specify):		D D M M Y Y Y Y

Sample

4. Date of referral to specialist medical services

At what date did you **first** refer the patient to hospital or another specialist transferring the responsibility for on-going investigation/treatment to other medical services?

D	D	M	M	Y	Y	Y	Y

Copyright property of the International Benchmarking Partnership (ICBP)
and Module 4 collaborators. Usage must be authorised

ICBP
International Cancer
Benchmarking Partnership

5. Nature of this referral

5.1 Do you know the date that the patient was seen for this referral?

☐ **Yes**, please provide the date: D D M M Y Y Y Y

☐ **No**

5.2 If you did make a referral to specialist services, which of the following best describes the nature/characteristics of this referral? Please tick **one**.

	✓
Emergency admission: a referral to A&E (or equivalent) for immediate admission	
An urgent referral for assessment of cancer symptoms/signs/test results (Note this will be within 2 weeks for England/Wales)	
A less urgent referral in which cancer is raised as a possibility (Note this will be greater than 2 weeks for England/Wales)	
A more general referral for investigation and assessment without cancer mentioned	
No referral was made	
Other – please describe	

Sample

5.3 Would you say this patient's diagnostic pathway was conducted predominantly in the public or private system? Please tick **one**.

	✓
Public healthcare system	
Private healthcare system	

Copyright property of the International Benchmarking Partnership (ICBP) and Module 4 collaborators. Usage must be authorised

ICBP
International Cancer Benchmarking Partnership

6. Date of lung cancer diagnosis

This can be decided in different ways. Please tick **all** that apply.
Please provide whichever of the following dates you have to hand:

	✓	
Date of histological confirmation [ideal]		D D M M Y Y Y Y
Date results of investigation (histological or other) confirming cancer received		D D M M Y Y Y Y
Date patient was told		D D M M Y Y Y Y
Date biopsy undertaken		D D M M Y Y Y Y
Date patient was first admitted to hospital because of the malignancy		D D M M Y Y Y Y
Other (please specify)		D D M M Y Y Y Y

Sample

Copyright property of the International Benchmarking Partnership (ICBP) and Module 4 collaborators. Usage must be authorised

ICBP
International Cancer Benchmarking Partnership

7. Additional information

Finally, we are interested to know what other conditions your patient has, and the severity/impact of these conditions

Have you and/or any of your partners treated this patient (or has the patient been to hospital) for any of the following conditions? Please tick **all** that apply:

Cardiovascular disease	☐ Yes ☐ No
Stroke	☐ Yes ☐ No
Lung disease (except lung cancer)	☐ Yes ☐ No
Diabetes	☐ Yes ☐ No

Sample

Are there any other comments you would like to make about this patient?

Name (and title): ______

Signature: ______

Date: ______

Thank you very much for taking the time to complete this questionnaire.

Copyright property of the International Benchmarking Partnership (ICBP)
and Module 4 collaborators. Usage must be authorised

A3: Cancer treatment specialists (CTS) questionnaire

ICBP
International Cancer Benchmarking Partnership

International Cancer Benchmarking Partnership Module 4

Specialist Care Audit
Lung Cancer

Thank you very much for agreeing to fill in this questionnaire – it should take about 10 minutes to complete. As part of an international study examining differences in cancer survival, we are sending the questionnaire to health care providers of a sample of patients with cancer.

Our aim is to gain a better understanding of the process by which people have their cancer diagnosed – the symptoms they experience, and the pathway they follow from onset of symptoms to treatment of their cancer. We hope you can help us with information on this patient's cancer journey once they were referred to specialist cancer services. This will help in identifying ways in which cancers can be diagnosed and treated quickly and effectively.

Sample

Thank you once again for your time

Please can you refer to your patient's notes in completing the questionnaire, as this will help in obtaining accurate data on time points.

If you would prefer to return this questionnaire without the patient details, please tear off along the dotted line.

Your patient ______________________________

is participating in the study.

Copyright property of the International Benchmarking Partnership (ICBP) and Module 4 collaborators. Usage must be authorised

ICBP
International Cancer Benchmarking Partnership

Sample

Patient information

ID-number: Jurisdiction-ID + Patient-ID:

Full name:

Address:

Postcode:

Date of birth: D D M M Y Y Y Y

Copyright property of the International Benchmarking Partnership (ICBP) and Module 4 collaborators. Usage must be authorised

ICBP
International Cancer Benchmarking Partnership

1. **Date patient first attended hospital/specialist services related to their cancer diagnosis.** We appreciate this date can at times be difficult to identify, particularly when there have been multiple visits in the lead up to a definitive diagnosis. Put another way, it's the date that the hospital/specialist service assumed responsibility for on-going investigation/treatment for your patient.

Day (optional), month, year

D	D	M	M	Y	Y	Y	Y

2. **How was the patient referred to the hospital/specialist services related to their cancer diagnosis?** Please tick.

Was it through a:

	✓		✓
GP referral		Referral from respiratory clinic	
Medical specialist/Consultant referral		Other referral – please specify:	

Sample

3. **Where did this first contact/appointment happen?** Please tick.
Which of the following best describes where this first contact/appointment took place?

	✓		✓
Emergency department ('A&E')		Medical outpatient department, please specify which department	
Oncology general outpatient department		Surgical outpatient department, please specify which department	
Other – please specify:			

Copyright property of the International Benchmarking Partnership (ICBP) and Module 4 collaborators. Usage must be authorised

ICBP
International Cancer Benchmarking Partnership

4. Date of diagnosis

This can be decided in different ways.
Please tick and complete as many of the following dates as possible.

	✓	
Date of histological confirmation (ideal)		Day (optional), month, year<br>D D M M Y Y Y Y
Date results of investigation confirming cancer received		Day (optional), month, year<br>D D M M Y Y Y Y
Date patient was told		Day (optional), month, year<br>D D M M Y Y Y Y
Date of biopsy		Day (optional), month, year<br>D D M M Y Y Y Y
Date patient was first admitted to hospital because of the malignancy		Day (optional), month, year<br>D D M M Y Y Y Y
Date of MDT confirmation of diagnosis		Day (optional), month, year<br>D D M M Y Y Y Y
Other (please specify):		Day (optional), month, year<br>D D M M Y Y Y Y

Sample

Copyright property of the International Benchmarking Partnership (ICBP)
and Module 4 collaborators. Usage must be authorised

ICBP
International Cancer Benchmarking Partnership

5. Date treatment for the cancer commenced

Based on your records, when would you say that any treatment specifically targeting the patient's cancer started?

Day (optional), month, year

D	D	M	M	Y	Y	Y	Y

6. Additional information

Please can you provide any further information on the patient's cancer:

**TNM**, please tick as appropriate:	
0	
I	
IIA	
IIB	
IIC	
IIIA	
IIIB	
IIIC	
IV	
Not able to stage	

Sample

Copyright property of the International Benchmarking Partnership (ICBP) and Module 4 collaborators. Usage must be authorised

ICBP
International Cancer Benchmarking Partnership

6.1 Histological subtype:

	✓
Squamous cell carcinoma	
Adenocarcinoma	
Large cell carcinoma	
Non-small carcinoma	
Small cell carcinoma	
Carcinoid	
Unclassified	
Other (please specify):	

Sample

Copyright property of the International Benchmarking Partnership (ICBP) and Module 4 collaborators. Usage must be authorised

ICBP
International Cancer Benchmarking Partnership

Further comments

Sample

Copyright property of the International Benchmarking Partnership (ICBP) and Module 4 collaborators. Usage must be authorised

ICBP
International Cancer Benchmarking Partnership

Name (and title):

Signature:

Date:

Sample

Are you a ... (please tick below): ✓

Surgeon	
Medical Oncologist	
Clinical Oncologist	
Clinical Nurse Specialist	
Other (please specify):	

Thank you very much for taking the time to complete this questionnaire.

Copyright property of the International Benchmarking Partnership (ICBP) and Module 4 collaborators. Usage must be authorised

Appendix B:

Rules for missing, incomplete, multiple response and out of range data

1. <u>Oversampling</u>
   To handle oversampling in Ontario, include only the first 315 consecutive lung cancer patients;

2. <u>Language/Participation in study/Presence of cancer</u>
   Exclude patients who checked “No, I don’t understand the language” or “I don’t want to participate in this study” or “I don’t have cancer”.

3. <u>Survey responders</u>
   a) Exclude Patient/PCP/Specialist survey from the analysis, if it was not written by Patient/PCP/Specialist (example: a medical oncologist completed a PCP survey);
   b) In the case of duplicates, include only the first survey (example: 2 specialists completed surveys for the same patient).

4. <u>Gender</u>
   Exclude patients with unknown Gender.

5. <u>Age</u>
   a) Exclude patients with unknown age;
   b) Exclude patients younger 40 years;
   c) Use registry data, if Age is reported by both patient and registry.

6. <u>No cancer or Previous cancer in the same organ</u>
   a) Exclude patients with no cancer based on registry data;
   b) Exclude patients with previous cancer in the same organ based on data from registry or free-text for Presentation in the patient survey.

7. <u>Date of consent</u>
   Exclude patients with date of consent which is unknown, before 01.01.2013 or in the future.

8. <u>Multiple responses to Dates</u>
   If multiple responses were given to the dates (of first symptom; first presentation to primary care; referral; diagnosis; treatment start), then use the earliest date.

9. <u>Order of Dates</u>
   The dates must be in the following order –

   First symptom; first presentation to Primary Care; referral; diagnosis; treatment start.

   If not, check for mistakes.

For peer review only - http://bmjopen.bmj.com/site/about/guidelines.xhtml

For peer review only

10. Date of first symptom

Date of first symptom is defined as date of first symptom from patient data.

11. Date of first presentation

Date of first presentation to Primary Care is defined as (in the order of declining priority):

a) date of first presentation to Primary Care from PCP data;
b) date of first presentation to Primary Care and A&E from PCP data;
c) date of first presentation to Primary Care from patient data.

12. Date of referral

Date of referral is defined as date of referral from PCP data.

13. Date of diagnosis

Definition

a) If Registry reports both date of histological confirmation and date of confirming investigation, then use date of histological confirmation.
b) Date of diagnosis (based on patient data, PCP data, specialist data, registry data) is defined as (in the order of declining priority):
   - date of diagnosis from registry;
   - date of histological confirmation (from specialist data, PCP data);
   - date of biopsy (from specialist data, PCP data);
   - date of confirming investigation (from specialist data, PCP data);
   - date of first hospital admission (from specialist data, PCP data);
   - date of MDT confirmation (from specialist data, PCP data);
   - date patient was told (from specialist data, PCP data);
   - other date of diagnosis (from specialist data, PCP data, patient data);

Choose a Date from a lower level of hierarchy, if the Date from a higher level is after the Date of consent or more than 9 months (=271 days) before the Date of consent.

Exclusion criteria

a) Unknown date of diagnosis;
b) Date of diagnosis is after the date of consent;
c) Date of diagnosis is more than 9 months before the Date of consent.

14. Date of treatment start

a) Date of treatment start from patient data is defined as the earliest of the treatment dates for Surgery, Chemo, Radio and Other;
b) Date of treatment start (based on registry data, specialist data, patient data) is defined as (in the order of declining priority):

For peer review only - http://bmjopen.bmj.com/site/about/guidelines.xhtml

- date of treatment start from registry data,
- date of treatment start from specialist data,
- date of treatment start from patient data,
- anticipated date of treatment from patient data.

15. Imputation of missing day in the date

Imputation rules for missing day (given month and year are known):

a) Set missing day to ‘16’;
b) Consider adjacent dates in a backwards order (from “Treatment” to “First symptom”). For each pair of such adjacent dates: If dates are not in a logical order (e.g. “Treatment” is before “Diagnosis”), but month and year are the same in both dates, and the day was imputed to ‘16’ in one of the dates:
   - Recode the day imputed earlier to ‘16’ to the day from the adjacent date.

16. Considering time

If patient gave multiple answers to the “How long did you have symptoms before contacting a doctor?” question, then use the option with the shortest time interval.

17. Delay arranging appointment

If patient gave multiple answers to the “How long did it take to get an appointment with PCP?” question, then use the option with the shortest time interval.

18. Duration of symptoms

If PCP gave multiple answers to the “Duration of symptoms” question, then use the option with the shortest time interval.

19. Definition of Presentation

A. *Define Presentation within a Data Source*

1. Review the free-text for Presentation (Patient, PCP sources) and re-code, if possible.
2. If PCP reports ‘Other’ as Presentation and at least one symptom (or “Duration of Symptoms”) or if Patient reports ‘Other’ as Presentation and at least one symptom (or date of first symptom or “Consider waiting time” or “Delay arranging appointment”), then re-code the Presentation in the corresponding data source to ‘Unknown’- option.
3. In the case of multiple Presentation responses (Patient, PCP sources) - use a single option (in the order of declining priority):
   a) ‘VisitPCP and AE’,
   b) ‘VisitPCP´, ‘AE´ (if both ‘VisitPCP´ and ‘AE´ are given, then re-code as ‘VisitPCP and AE’),
   c) ‘Unknown’,
   d) ‘Investigation for another problem’ ,
   e) ’Other”

B. *Define Presentation from Alternative Data*

For peer review only - http://bmjopen.bmj.com/site/about/guidelines.xhtml

If Presentation hasn't been reported in either of data sources, then define it 'Unknown',

if PCP reports at least one symptom (or "Duration of symptoms"); or if Patient reports

at least one symptom (or date of first symptom or "Consider waiting time" or "Delay arranging

appointment");

C. *Define Presentation from Data Source Hierarchy*

1. In all jurisdictions, except Sweden – use Presentation data from (in the order of declining priority):
   a) PCP data;
   b) Patient data;
2. In Sweden – use Presentation data from Patient data.

20. Patient interval

The Patient interval is defined as (in the order of declining priority):

a) "Date of first presentation to Primary Care" (rule 11) minus "Date of first symptom" (rule 10);
b) If the interval in (a) is unknown or negative: Calculate the interval as the low boundary of "Considering time" (rule 16) plus the low boundary of "Delay arranging appointment" (rule 17);
c) If the interval in (a) is unknown or negative and the interval in (b) is unknown: Calculate the interval as the low boundary of "Duration of symptoms interval" (rule 18).

21. Primary Care interval

The Primary Care interval is defined as "Date of referral" (rule 12) minus "Date of first presentation to Primary Care" (rule 11).

22. Diagnostic interval

a) The Diagnostic interval is defined as "Date of diagnosis" (rule 13) minus "Date of first presentation to Primary Care" (rule 11).

23. Treatment interval

The Treatment interval is defined as "Date of treatment start" (rule 14) minus "Date of diagnosis" (rule 13).

24. Total interval

a) The Total interval is defined as "Date of treatment start" (rule 14) minus "Date of first symptom" (rule 10).

25. Range of Time intervals

The time intervals (Patient, Primary Care, Diagnosis, Treatment, Total) must be in range 0-1 year.

If > 1 year: set the interval to 365 days

If negative: set the interval to 0.

For peer review only - http://bmjopen.bmj.com/site/about/guidelines.xhtml

26. Number of visits
If patient gave multiple answers to the "Number of visits" questions, then use the option with a fewer number of visits.

27. Specialist waiting time interval
If patient gave multiple answers to the ""How long did it take to get an appointment with specialist?" question, then use the option with the shortest time interval.

28. Type of treatment
If patient ticked both "Yes" and "No" as answers to the "Type of treatment (Surgery, Chemotherapy, Radiotherapy)" questions, then choose "Yes" answer.

29. Health state
If patient gave multiple answers to the "Health state" question, then use the option with a better health condition.

30. Comorbidity
a) If patient ticked both "Yes" and "No" as answers to the "Comorbidity (Heart disease, Stroke, Lung disease, Diabetes)" questions, then choose "Yes" answer;
b) If both patient and PCP report "Comorbidity", then use the PCP Data.

31. Ethnicity
a) If patient didn't report "Ethnicity", then use the information from (in the order of declining priority):
    - "Ethnicity_Other_Details";
    - "Other main language spoken at home";
    - "The main language spoken at home" (only for Victoria);
    - "The main language spoken at home is the chief one for this jurisdiction"="Yes" given "Main language spoken at home is other than the main one for this jurisdiction"="No";

b) Consider Ethnicity as unknown, if answers to the "Ethnicity" question are multiple and belong to
different categories ('white', 'Asian', 'black', 'other').

32. Education
If patient gave multiple answers to the "Education" question, then use the option with a higher level of education.

33. Smoking Current
a) If patient ticked both "Yes" and "No" as answers to the "Smoking Current" question, then use "Yes" answer;
b) If patient hasn't ticked neither "Yes" nor "No, then consider this case as Unknown.

For peer review only - http://bmjopen.bmj.com/site/about/guidelines.xhtml

34. Smoking Number

If patient reports "SmokingNumber" as text, then re-code using following rules:

a) Where there is a number smoked /day – accept number;
b) Where a range has been given – take the upper value;
c) Where patient has put 10+ or 20+ - capture this as 11 or 21;
d) Where number of cigarettes smoked in the past and currently being smoked are provided - average the numbers;
e) Non entries code as "." ;
f) Non-smokers (eg, "nil", "N/A") are coded as "0".

35. Smoked ever

a) If patient ticked both "Yes" and "No" as answers to the "Smoking ever" question, then use "Yes" answer;
b) If patient hasn't ticked neither "Yes" nor "No": consider it as "Yes", if patient is a current smoker ("Smoking_Current="Yes"") or has specified a number of cigarettes ("SmokingNumber">0). Otherwise consider this case as Unknown.
c) If patient has ticked "No": recode it to "Yes", if patient is a current smoker ("Smoking_Current="Yes").

36. Nature of referral

a) Review free-text for " Nature of referral" (PCP Data) and re-code, if possible;
b) In the case of multiple responses, use a single option as (in the order of declining priority):

- "Referral for immediate admission";
- "Urgent referral";
- "Less urgent referral";
- "General referral" ;
- "No referral";
- "Other".

37. Refer Public or Private

a) If PCP ticked both "Public" and "Private" as answers to the "Refer Public or Private" question, then use "Private" answer;
b) If PCP hasn't ticked neither "Public" nor "Private", then consider this case as Unknown.

38. Type of referral

If specialist gave multiple responses to the "How was the patient referred…" question, then use a single option (in the order of declining priority):

- "Respiratory clinic";
- "Specialist/consultant";
- "PCP";
- "Other".

For peer review only - http://bmjopen.bmj.com/site/about/guidelines.xhtml

39. First Attendance Place
If specialist gave multiple responses to the “First Attendance Place” question, then consider this case as Unknown.

40. Stage-TNM
a) If specialist gave multiple responses to the “Stage_TNM” question, then use the highest category;
b) If registry gave multiple responses to the “Stage_TNM”, then use a single option (in the order of declining priority):
   - stage at time of diagnosis
   - stage at surgery
   - stage at oncology
c) If “Stage_TNM” is reported by both the specialist and registry, then use the registry data.

For peer review only

Appendix C: Supplementary Tables

C 1: Classification of lung cancer -specific symptoms reported by patients into cancer specific and non- cancer specific

Cancer specific symptoms

1 persistent cough
2 chest infection that wouldn't get better
3 coughing up blood-stained phlegm (sputum)
4 breathlessness
5 chest or shoulder pain
6 a hoarse voice
7 difficulty swallowing
8 swelling of lymph nodes (glands) in my neck
9 ends of fingers becoming larger
10 breast and axilla swelling (in men)

Non-cancer specific symptoms

1 a dull ache or sharp pain when I coughed or took a deep breath
2 pain/discomfort under my ribs
3 face swelling
4 blood clots (thrombosis)
5 pins and needles or numbness in fingers
6 weight loss
7 felt sick/vomiting/nausea/loss of appetite
8 fatigue
9 muscle weakness
10 drowsiness, weakness, dizziness or confusion
11 high temperature (fever) of 38C (100.4F)
12 other

C2: Time intervals (days) depicted as median, 75th and 90th centiles for each of the ten jurisdictions. In Sweden, no data was available on primary care interval

For peer review only

Intervals	Centiles	Wales	Denmark	Sweden	England	N Ireland	Scotland	Manitoba	Ontario
Patient Interval	Number	181	233	172	213	179	169	133	205
	Median	21	14	21	17	18	21	25	22
	75th centile	61	53	61	65	60	60	67	61
	90th centile	216	180	214	205	240	267	180	187
Primary Care interval	Number	110	159	n/a	147	124	119	80	75
	Median	20	7		11	13	16	30	29
	75th centile	43	20		31	51	35	75	73
	90th centile	91	64		73	112	90	138	183
Diagnostic interval	Number	176	229	165	212	170	173	138	212
	Median	45	35	28	54	65	42	87	57
	75th centile	108	67	83	100	122	106	147	122
	90th centile	162	162	143	161	281	198	265	331
Treatment interval	Number	192	279	190	238	200	187	182	263
	Median	43	16	34	22	32	42	19	47
	75th centile	64	25	59	41	48	62	56	70
	90th centile	89	37	77	56	72	90	97	96
Total interval	Number	147	192	147	176	157	143	117	178
	Median	116	67	107	114	105	117	127	130
	75th centile	204	116	190	183	227	253	216	216
	90th centile	365	210	329	323	365	365	365	339

For peer review only

C3: Data sources used to define dates and percentage of imputed dates

Type of date	Data sources used to define a date* (%)				Cases with imput day in a date** (%
	Patient	PCP	CST	Registry	
First noticing symptoms	100	0	0	0	66
First presentation to health care	49	51	0	0	30
First referral to secondary care	0	100	0	0	1
Diagnosis	5	6	8	81	1
Start of curative or palliative treatment	55	0	32	13	11

* based on rules 10-14, supplementary file Appendix B
** based on rule 15, supplementary file Appendix B

For peer review only

For peer review only - http://bmjopen.bmj.com/site/about/guidelines.xhtml

C4. Percentages of negative intervals set to 0, large intervals (>365 days) set to 365 days, and intervals based not on dates

Type of interval	Negative intervals set to 0 days* (%)	Intervals >365 days set to 365 days* (%)	Intervals where variables other than dates were used (%)**
Patient	<1	5	29
Primary care	4	2	0
Diagnosis	6	5	0
Treatment	6	<1	0
Total	2	9	0

* based on rule 25, supplementary file Appendix B
* based on rule 20b,c, supplementary file Appendix B

For peer review only

For peer review only

For peer review only - http://bmjopen.bmj.com/site/about/guidelines.xhtml

The RECORD statement – checklist of items, extended from the STROBE statement, that should be reported in observational studies using routinely collected health data.

	Item No.	STROBE items	Location in manuscript where items are reported	RECORD items	Location in manuscript where items are reported
**Title and abstract**					
	1	(a) Indicate the study's design with a commonly used term in the title or the abstract (b) Provide in the abstract an informative and balanced summary of what was done and what was found	Title and abstract – p 1 and 3	RECORD 1.1: The type of data used should be specified in the title or abstract. When possible, the name of the databases used should be included.<br><br>RECORD 1.2: If applicable, the geographic region and timeframe within which the study took place should be reported in the title or abstract.<br><br>RECORD 1.3: If linkage between databases was conducted for the study, this should be clearly stated in the title or abstract.	Abstract – p 3<br><br>Abstract – p 3<br><br>N/A
**Introduction**					
Background rationale	2	Explain the scientific background and rationale for the investigation being reported	Introduction – p 6		
Objectives	3	State specific objectives, including any prespecified hypotheses	Introduction – p 6		
**Methods**					
Study Design	4	Present key elements of study design early in the paper	Methods – p 6		
Setting	5	Describe the setting, locations, and relevant dates, including periods of recruitment, exposure, follow-up, and data collection	Methods – p 6-8		
Participants	6	*(a) Cohort study* - Give the eligibility criteria, and the	Methods – p 6 – and as reference to	RECORD 6.1: The methods of study population selection (such as codes or	Provided as reference to

		sources and methods of selection of participants. Describe methods of follow-up<br>*Case-control study* - Give the eligibility criteria, and the sources and methods of case ascertainment and control selection. Give the rationale for the choice of cases and controls<br>*Cross-sectional study* - Give the eligibility criteria, and the sources and methods of selection of participants<br><br>*(b) Cohort study* - For matched studies, give matching criteria and number of exposed and unexposed<br>*Case-control study* - For matched studies, give matching criteria and the number of controls per case	previous paper.<br><br>N/A	algorithms used to identify subjects) should be listed in detail. If this is not possible, an explanation should be provided.<br><br>RECORD 6.2: Any validation studies of the codes or algorithms used to select the population should be referenced. If validation was conducted for this study and not published elsewhere, detailed methods and results should be provided.<br><br>RECORD 6.3: If the study involved linkage of databases, consider use of a flow diagram or other graphical display to demonstrate the data linkage process, including the number of individuals with linked data at each stage.	previous paper.<br><br>Provided as appendix and in reference to previous paper.<br><br>N/A
Variables	7	Clearly define all outcomes, exposures, predictors, potential confounders, and effect modifiers. Give diagnostic criteria, if applicable.	Methods – p 6-9 – and as reference to previous paper.	RECORD 7.1: A complete list of codes and algorithms used to classify exposures, outcomes, confounders, and effect modifiers should be provided. If these cannot be reported, an explanation should be provided.	N/A
Data sources/ measurement	8	For each variable of interest, give sources of data and details of methods of assessment (measurement).<br>Describe comparability of assessment methods if there is more than one group	Methods – p 6-9 – and as reference to previous paper.		
Bias	9	Describe any efforts to address potential sources of bias	Methods – p 7-8, discussion – p 24-25 – and as reference to		

For peer review only - http://bmjopen.bmj.com/site/about/guidelines.xhtml

			previous paper.		
Study size	10	Explain how the study size was arrived at	Methods – p 6 – and as reference to previous paper.		
Quantitative variables	11	Explain how quantitative variables were handled in the analyses. If applicable, describe which groupings were chosen, and why	Methods – p 6-8 – and as reference to previous paper.		
Statistical methods	12	(a) Describe all statistical methods, including those used to control for confounding<br>(b) Describe any methods used to examine subgroups and interactions<br>(c) Explain how missing data were addressed<br>(d) *Cohort study* - If applicable, explain how loss to follow-up was addressed<br>*Case-control study* - If applicable, explain how matching of cases and controls was addressed<br>*Cross-sectional study* - If applicable, describe analytical methods taking account of sampling strategy<br>(e) Describe any sensitivity analyses	Methods – p 8 – and as reference to previous paper.		
Data access and cleaning methods		..		RECORD 12.1: Authors should describe the extent to which the investigators had access to the database population used to create the study population.	N/A

For peer review only

				RECORD 12.2: Authors should provide information on the data cleaning methods used in the study.	Methods – p 7
Linkage		..		RECORD 12.3: State whether the study included person-level, institutional-level, or other data linkage across two or more databases. The methods of linkage and methods of linkage quality evaluation should be provided.	N/A
**Results**					
Participants	13	(a) Report the numbers of individuals at each stage of the study (*e.g.*, numbers potentially eligible, examined for eligibility, confirmed eligible, included in the study, completing follow-up, and analysed)<br>(b) Give reasons for non-participation at each stage.<br>(c) Consider use of a flow diagram	Results and as table – p 10-11. Flow diagram in previous paper, referenced.	RECORD 13.1: Describe in detail the selection of the persons included in the study (*i.e.,* study population selection) including filtering based on data quality, data availability and linkage. The selection of included persons can be described in the text and/or by means of the study flow diagram.	Results – p 10-15 - and as table. Flow diagram in previous paper, referenced.
Descriptive data	14	(a) Give characteristics of study participants (*e.g.*, demographic, clinical, social) and information on exposures and potential confounders<br>(b) Indicate the number of participants with missing data for each variable of interest<br>(c) *Cohort study* - summarise follow-up time (*e.g.*, average and total amount)	Results and as table – p 10-15.		
Outcome data	15	*Cohort study* - Report numbers of outcome events or summary measures over time<br>*Case-control study* - Report numbers in each exposure category, or summary measures	Results and as table – p 16-23.		

For peer review only

		of exposure<br>*Cross-sectional study* - Report numbers of outcome events or summary measures			
Main results	16	(a) Give unadjusted estimates and, if applicable, confounder-adjusted estimates and their precision (e.g., 95% confidence interval). Make clear which confounders were adjusted for and why they were included<br>(b) Report category boundaries when continuous variables were categorized<br>(c) If relevant, consider translating estimates of relative risk into absolute risk for a meaningful time period	Results and as table – p 21-23.		
Other analyses	17	Report other analyses done—e.g., analyses of subgroups and interactions, and sensitivity analyses	Results – p 23.		
**Discussion**					
Key results	18	Summarise key results with reference to study objectives	Discussion – p 24		
Limitations	19	Discuss limitations of the study, taking into account sources of potential bias or imprecision. Discuss both direction and magnitude of any potential bias	Discussion – p24-25	RECORD 19.1: Discuss the implications of using data that were not created or collected to answer the specific research question(s). Include discussion of misclassification bias, unmeasured confounding, missing data, and changing eligibility over time, as they pertain to the study being reported.	Discussion – p24-25
Interpretation	20	Give a cautious overall interpretation of results considering objectives, limitations, multiplicity of analyses, results from similar	Discussion – p25-28		

For peer review only

		studies, and other relevant evidence			
Generalisability	21	Discuss the generalisability (external validity) of the study results	Discussion, conclusion – p25-29.		
**Other Information**					
Funding	22	Give the source of funding and the role of the funders for the present study and, if applicable, for the original study on which the present article is based	Funding statement – p 31		
Accessibility of protocol, raw data, and programming code		..		RECORD 22.1: Authors should provide information on how to access any supplemental information such as the study protocol, raw data, or programming code.	Availability of data and material statement – p 31

*Reference: Benchimol EI, Smeeth L, Guttmann A, Harron K, Moher D, Petersen I, Sørensen HT, von Elm E, Langan SM, the RECORD Working Committee. The REporting of studies Conducted using Observational Routinely-collected health Data (RECORD) Statement. *PLoS Medicine* 2015; in press.

*Checklist is protected under Creative Commons Attribution (CC BY) license.

For peer review only

BMJ Open

Time intervals and routes to diagnosis for lung cancer in ten jurisdictions: cross-sectional study findings from the International Cancer Benchmarking Partnership (ICBP)

Journal:	*BMJ Open*
Manuscript ID	bmjopen-2018-025895.R2
Article Type:	Research
Date Submitted by the Author:	18-Jul-2019
Complete List of Authors:	Menon, Usha; University College London, Institute for Women's Health<br>Vedsted, Peter; Aarhus University, Research Unit for General Practice<br>Zalounina Falborg, Alina; Aarhus University, Research Unit for General Practice<br>Jensen, Henry; Aarhus University, Research Unit for General Practice<br>Harrison, Samantha; Cancer Research UK, Policy & information<br>Reguilon, Irene; Cancer Research UK, Policy & information<br>Barisic, Andriana; Cancer Care Ontario<br>Bergin, Rebecca; Cancer Council Victoria, Centre for Behavioural Research in Cancer; University of Melbourne, Department of General Practice<br>Brewster, David; Edinburgh University, Centre for Population Health Sciences; NHS Scotland National Services Division, Scottish Cancer Registry, Information Services Division<br>Butler, John; The Royal Marsden<br>Brustugun, Odd Terje; Olso University Hospital, Institute for Cancer Research<br>Bucher, Oliver; CancerCare Manitoba, Department of Epidemiology and Cancer Registry<br>Cairnduff, Victoria; Queen's University Belfast, Northern Ireland Cancer Registry<br>Gavin, Anna; Queen's University Belfast, Northern Ireland Cancer Registry<br>Grunfeld, Eva; Ontario Institute for Cancer Research, Health Services Research Program<br>Harland, Elizabeth; CancerCare Manitoba, Department of Epidemiology and Cancer Registry<br>Kalsi, Jatinderpal; University College London, Institute for Women's Health<br>Knudsen, Anne Kari; Olso University Hospital, European Palliative Care Research Centre (PRC); University of Oslo, Institute of Clinical Medicine<br>Lambe, Mats; Karolinska Institutet, Department of Medical Epidemiology; University Hospital, Regional Oncologic Center<br>Law, Rebecca-Jane; Bangor University, North Wales Centre for Primary Care Research<br>Lin, Yulan; University of Oslo, Institute of Clinical Medicine; Olso University Hospital, European Palliative Care Research Centre (PRC)<br>Malmberg, Martin; Skanes universitetssjukhus Lund<br>Turner, Donna; CancerCare Manitoba

	Neal, Richard; Bangor University, North Wales Centre for Primary Care Research; University of Leeds, Academic Unit of Primary Care<br>White, Victoria; Cancer Council Victoria, Centre for Behavioural Research in Cancer; Deakin University, School of Psychology<br>Weller, David; Edinburgh University, Centre for Population Health Sciences

**Primary Subject Heading**:	Epidemiology
Secondary Subject Heading:	Global health, Oncology
Keywords:	EPIDEMIOLOGY, International health services < HEALTH SERVICES ADMINISTRATION & MANAGEMENT, Organisation of health services < HEALTH SERVICES ADMINISTRATION & MANAGEMENT, Respiratory tract tumours < ONCOLOGY, lung cancer, routes to diagnosis, time intervals, international health systems, symptomatic presentation

SCHOLARONE™
Manuscripts

Time intervals and routes to diagnosis for lung cancer in ten jurisdictions: cross-sectional study findings from the International Cancer Benchmarking Partnership (ICBP)

1. Usha Menon*, MRC Clinical Trials Unit at UCL, Institute of Clinical Trials & Methodology, , University College London, London, United Kingdom

2. Peter Vedsted*, Research Unit for General Practice, Aarhus University, Aarhus, Denmark

3. Alina Zalounina Falborg, Research Unit for General Practice, Aarhus University, Aarhus, Denmark

4. Henry Jensen, Research Unit for General Practice, Aarhus University, Aarhus, Denmark

5. Samantha Harrison, Policy and Information, Cancer Research UK, London, United Kingdom

6. Irene Reguilon, Policy and Information, Cancer Research UK, London, United Kingdom

7. Andriana Barisic, Ontario Renal Network, Cancer Care Ontario, Toronto, Canada

8. Rebecca J Bergin, Centre for Behavioural Research in Cancer, Cancer Council Victoria, Victoria, Australia; Department of General Practice, University of Melbourne, Victoria, Australia

9. David H Brewster, Scottish Cancer Registry, Information Services Division, NHS National Services Scotland, Edinburgh, United Kingdom; Centre for Population Health Sciences, University of Edinburgh, Edinburgh, United Kingdom

10. John Butler, The Royal Marsden, Fulham Road, London, United Kingdom

11. Odd Terje Brustugun, Institute for Cancer Research, Department of Cancer Genetics, Oslo University Hospital, Oslo, Norway

12. Oliver Bucher, Department of Epidemiology and Cancer Registry, CancerCare Manitoba, Manitoba

13. Victoria Cairnduff, Northern Ireland Cancer Registry, Centre for Public Health, Queen's University Belfast, Mulhouse Building, Mulhouse Road, Belfast, United Kingdom

14. Anna T Gavin, Northern Ireland Cancer Registry, Queen's University Belfast, Belfast, United Kingdom

For peer review only

15. Eva Grunfeld, Health Services Research Program, Ontario Institute for Cancer Research, Toronto, Canada

16. Elizabeth Harland, Department of Epidemiology and Cancer Registry, CancerCare Manitoba, Winnipeg, Canada

17. Jatinderpal Kalsi, Women's Cancer, Institute for Women's Health, University College London, London, United Kingdom

18. Anne Kari Knudsen, European Palliative Care Research Centre (PRC), Department of Oncology, Oslo University Hospital and Institute of Clinical Medicine, University of Oslo, Oslo, Norway

19. Mats Lambe, Regional Cancer Center Uppsala and Department of Medical Epidemiology and Biostatics, Karolinska Institutet, Stockholm, Sweden

20. Rebecca-Jane Law, North Wales Centre for Primary Care Research, Bangor University, Wrexham, United Kingdom

21. Yulan Lin, European Palliative Care Research Centre (PRC), Department of Oncology, Oslo University Hospital and Institute of Clinical Medicine, University of Oslo, Oslo, Norway

22. Martin Malmberg, Department of Oncology, Lund University Hospital, Lund, Sweden

23. Donna Turner, Population Oncology, Cancer Care Manitoba, Winnipeg, Canada

24. Richard D Neal, North Wales Centre for Primary Care Research, Bangor University, Wrexham, United Kingdom; Academic Unit of Primary Care, University of Leeds, Leeds, United Kingdom

25. Victoria White, Centre for Behavioral Research in Cancer, Cancer Council Victoria, Victoria, Australia; School of Psychology Deakin University, Geelong, Victoria, 3217, Australia

26. David Weller*, Centre for Population Health Sciences, University of Edinburgh, Edinburgh, United Kingdom

On behalf of the ICBP Module 4 Working Group

* equal contribution

For peer review only

Corresponding author:
Professor Usha Menon
MRC CTU at UCL
Institute of Clinical Trials and Methodology
90 High Holborn, 2nd Floor, London WC1V 6LJ
Email: u.menon@ucl.ac.uk

Word count: 3074; Tables: 6 ; Figures: 2 ; Appendix A-D

For peer review only

Abstract

Objective

Differences in time intervals to diagnosis and treatment between jurisdictions may contribute to previously reported differences in stage at diagnosis and survival. The International Cancer Benchmarking Partnership Module 4 (ICBP M4) reports the first international comparison of routes to diagnosis and time intervals from symptom onset until treatment start for lung cancer patients.

Design

Newly diagnosed lung cancer patients, their primary care physicians (PCP) and cancer treatment specialists (CTS) were surveyed in Victoria (Australia), Manitoba and Ontario (Canada), Northern Ireland, England, Scotland and Wales (UK), Denmark, Norway and Sweden. Using Wales as the reference jurisdiction, the 50th, 75th and 90th percentiles for intervals were compared using quantile regression adjusted for age, gender and comorbidity.

Participants

Consecutive newly diagnosed lung cancer patients, aged >40 years, diagnosed between October 2012 and March 2015 were identified through cancer registries. Of 10,203 eligible symptomatic patients contacted, 2,631 (27.5%) responded and 2,143 (21.0%) were included in the analysis. Data was also available from 1,211 (56.6%) of their PCPs and 643 (37.0%) of their CTS.

Primary and secondary outcome measures:

Interval lengths (days, primary), routes to diagnosis, symptoms (secondary).

Results

With the exception of Denmark (-49 days), in all other jurisdictions the median adjusted total interval from symptom onset to treatment, for respondents diagnosed in 2012-15, was similar to that of Wales (116 days). Denmark had shorter median adjusted primary care interval (-11 days) than Wales (20 days); Sweden had shorter (-20) and Manitoba longer (+40) median adjusted diagnostic intervals compared to Wales (45 days). Denmark (-13), Manitoba (-11), England (-9) and Northern Ireland (-4) had shorter median adjusted treatment intervals than Wales (43 days). The differences were greater for the 10% of patients who waited the longest. Based on overall trends, jurisdictions could be grouped into those with trends of reduced,

For peer review only - http://bmjopen.bmj.com/site/about/guidelines.xhtml

longer and similar intervals to Wales.The proportion of patients diagnosed following presentation to the PCP ranged from 35-75%.

Conclusion

There are differences between jurisdictions in interval to treatment, which are magnified in lung cancer patients who wait the longest. The data could help jurisdictions develop more focused lung cancer policy and targeted clinical initiatives. Future analysis will explore if these differences in intervals impact on stage or survival.

Key words:

lung cancer, routes to diagnosis, time intervals, international health systems, symptomatic presentation

For peer review only

For peer review only - http://bmjopen.bmj.com/site/about/guidelines.xhtml

Strengths and limitations of this study

- This is the first study to use standardized survey methods and definitions to systematically examine key intervals from patients first noticing symptoms or bodily changes until the start of treatment for lung cancer across multiple jurisdictions
- Recall bias was minimised by the triangulation of different data sources and by patients completing the questionnaire within a limited time window (median 5 months) after the cancer diagnosis
- A key limitation, as with all questionnaire-based studies was selection and non-response bias which varied across jurisdictions
- Recruitment of patients up to 9 rather than 6 months after diagnosis might have magnified selection bias due to high mortality in lung cancer but a sensitivity analysis suggests that this did not impact on the results.
- The comparisons for Norway and Victoria, are limited by small sample size and inclusion of only surgical patients, respectively.

For peer review only

Introduction

Lung cancer is the most common cancer worldwide, with nearly 1.83 million cases diagnosed in 2012, and is the leading cause of cancer death globally, accounting for 19% of cancer deaths.[1] Survival is typically low, with 5-year survival in Europe,North America and Australia <20%.[2-3] A key factor is diagnosis at advanced stage. Reasons for this are multi-faceted and include delays due to the atypical nature of some presenting symptoms, poor sensitivity of chest X-rays and physicians not acting quickly enough.[4] Within European countries, differences of 12 and 5 percentage points in 1- and 5-year relative survival, respectively, have been reported for lung cancers diagnosed between 1999-2007.[5] This and other international comparisons raises the possibility of additional contributory factors such as variations in referral patterns, access to diagnostic tests and delays in treatment.[6]

One way of addressing this is to chart the patient journey from first noticing symptoms to treatment start. Many national studies using different methodologies have reported on time intervals to treatment of lung cancer and there are reviews that have looked at international timeframe comparisons [7-10] [11-24] However, as far as we are aware there is no study that has undertaken international comparisons of timeliness across multiple countries using the same methodology.

The International Cancer Benchmarking Partnership (ICBP) was established to explore differences in cancer outcomes and their causes in countries with comparable wealth and universal access to healthcare.[25] We report results from Module 4 (ICBP M4) on differences in time intervals and routes to diagnosis in symptomatic lung cancer patients from ten jurisdictions in Australia, Canada, Denmark, Norway, Sweden and the United Kingdom.

Methods

Methods have been previously detailed.[26] In brief, in each of the ten participating jurisdictions (Victoria (Australia), Manitoba, Ontario (Canada), Denmark, Norway, Sweden Northern Ireland, England, Scotland and Wales), consecutive patients aged ≥40 years, newly diagnosed with malignant lung or bronchus cancer (ICD-10:C34.0-C34.9; ICD-O-3 behaviour code /3) were identified by the cancer registry using validated methods (hospital episode, cancer registration, pathology). Exclusion criteria included previous lung or synchronous cancers. Patients with a previous non-lung primary cancer were eligible. Target recruitment was 200 symptomatic patients per jurisdiction.

Following a vital status check, cancer registries posted the patient questionnaire (Appendix A1) either 1) to the relevant primary care physician (PCP) who then forwarded the pre-

For peer review only - http://bmjopen.bmj.com/site/about/guidelines.xhtml

addressed envelope to the patient after confirmation that the person was aware of the diagnosis and not deemed too sick/anxious to participate in the survey. (Wales, England, Scotland) or 2) to the patient directly or via the research team (remaining seven jurisdictions). In an attempt to decrease attrition and recall bias, the protocol initially specified that all patient questionnaires should be completed within 6 months of diagnosis. As there were administrative delays in cancer notification, this was extended to 9 months.

On receipt of a completed patient questionnaire, in all jurisdictions except Sweden, the relevant PCP and cancer treatment specialist (CTS) were sent questionnaires (Appendix A.2 and A.3). Specialists provided information on diagnosis and start date of treatment. The latter was collected directly from registry records in Northern Ireland and clinical databases in Denmark. Manitoba did not provide specialist data. Date of diagnosis and stage was also collected where possible through cancer registries. Information on the types of treatment (surgery, chemotherapy, radiotherapy and other) were obtained from the patient survey.

Data handling

Data were recoded centrally to ensure that the same explicit rules were applied throughout. Patients in whom age, date of diagnosis or consent were missing were excluded from analyses. Rules were used to combine data from the different sources in a standardised way that ensured reproducibility and transparency (Appendix B). The rules employed a 'hierarchy' principle in terms of the order in which different data sources were used and included imputation rules based on the available data. The exact rule was guided by the measure in question – for example, patient interval was collected primarily from the patient questionnaire whereas primary care time-points from the PCP questionnaire. We applied rules for outliers and implausible measures (e.g. negative time intervals were recorded to zero-days and intervals longer than a year to 365 days).

Routes to diagnosis and symptoms prompting physician visit

These were derived from patient and PCP responses. Symptoms were coded by two PCP authors (DW and PV) into 'lung cancer specific' or 'other' (Appendix C1).

Time intervals

Time intervals were derived using the checklist for the Aarhus Statement.[27] The following time-points were used to calculate the corresponding time intervals (Figure 1):

- first noticing symptoms
- first presentation to health care
- first referral to secondary care

For peer review only - http://bmjopen.bmj.com/site/about/guidelines.xhtml

- diagnosis date
- start of curative or palliative treatment

FIGURE 1

All time-points were validated if there were obvious inconsistencies and negative intervals were set to 0 days. All intervals were truncated at 365 days. Missing data were imputed based on specific rules to ensure that the direction of a possible misclassification bias was known.

Demographics

Health status was measured using the self-reported general health item from short form 36 health survey (SF36).[28] Comorbidity was defined as one of four patient or PCP-reported diseases (heart or lung disease, stroke or diabetes) and categorised into: ‘none’, ‘medium’ (one or two) or ‘high’ (three or four). Level of educational was categorised as ‘low’ (vocational school or lower) and ‘high’ (university). Stage data (tumour, node, metastasis - TNM - classification) was grouped as I, II, III, IV or missing.[29]

Statistical analysis

Patient characteristics across jurisdictions were compared using Kruskal-Wallis test for continuous and ordinal data. For nominal data we used Pearson’s chi-squared test and Fisher’s exact test (if more than 20% of expected cell counts were less than 5 or at least one expected cell count was 0). The differences in intervals between the jurisdictions were estimated using quantile regression, as this method allows for a comparison across the whole distribution of length of the interval.[30] As we were interested in a measure of central tendency of length of the interval and in long and very long intervals, the focus of the study was on the 50th(median), 75th and 90th interval percentiles. Wales was chosen as the reference jurisdiction as it had the lowest lung cancer survival in ICBP Module 1 analysis.[10] Since the length of the interval in days is a continuous measure which has been rounded, we applied the quantile regression analysis on the smoothed quantiles; the method based on the smoothed quantiles is recommended for analyses of discrete (count) data [31]. In STATA this method is implemented in the ‘qcount’ procedure.[32] Parameters were calculated with 1000 jittered samples. For all interval analyses, the differences in intervals were calculated as marginal effects after quantile regression by setting the continuous covariate (age) to their mean values and the categorical covariates (sex and comorbidity) to their modes. Significance level was set to $\leq$0.05 with 95% confidence intervals (95%CI) calculated where appropriate. Statistical analyses were carried out using STATA v14.

For peer review only - http://bmjopen.bmj.com/site/about/guidelines.xhtml

Sensitivity and validity analyses

All analyses were repeated using only (1) those who fulfilled the 6-month cut-off criteria for interval from diagnosis to questionnaire completion and (2) patient data. The effect of excluding patients for whom at least one interval was missing was investigated.We also repeated the analysis after omitting time intervals which were negative or over 365 days. Agreement between the different data sources (registry, patient, PCP (except in Sweden) and CTS (except in Sweden and Manitoba) was measured by Lin's concordance correlation coefficient (CCC).[33]

Patient involvement

The research questions for this survey drew on an extensive literature relating to diagnosis and treatment delays leading to negative patient experiences. While patient experience was not a primary outcome measure for this study, patients were given the opportunity to comment on their experience through questionnaire free-text response options (under separate analysis). Patients were involved in the piloting of study instruments to ascertain if recruitment and questionnaire content and dissemination strategies were appropriate, described elsewhere.[26] Each jurisdiction has committed to communicating the findings and local implications of this study to organisations representing their study participants.

Results

Of 14,583 lung cancer patients, diagnosed between October 2012 and March 2015 who were alive when identified, 70% (10,203/14,583) were contacted (Table 1). Of 4380 not contacted, 3367 (77%) were from England, Wales and Scotland. Major reasons reported by the PCP for not forwarding the survey included patients being terminally ill, not aware of cancer diagnosis at the time of request, having cognitive or visual impairment, language / communication difficulties, no longer at the address, not wishing to take part in research and a handful not having the index cancer. In addition patents identified were not contacted in England as the target recruitment had been exceeded. For the non-UK jurisdictions, the main reasons for not contacting patients were the patient having died or no longer at the address.

2,631 (27.5% of contacted, 18% of eligible) completed the patient questionnaire at a median of 5 months (range: 0.1,9) after diagnosis (Table 2). The response rate of contacted patients varied from 11.1% (146/1318) in Norway to 61.8% (333/539) in Denmark. Responding patients were more likely to be aged 60-79 years with less advanced stage (Table 1) and alive one year post diagnosis (data not shown). Of the 2,631 responses, 2,143 (81.5%) were included in the analyses which equates to 14.7% (2,143/14,583) of eligible patients. The key reason respondents were excluded were local oversampling (43.9%; 214/488) for additional analyses

For peer review only - http://bmjopen.bmj.com/site/about/guidelines.xhtml

(Table 1). In Victoria, the registry was only able to contact patients who had undergone surgery while the sample size in Norway was limited (n=88) due to delays in securing appropriate approvals.

For peer review only

For peer review only - http://bmjopen.bmj.com/site/about/guidelines.xhtml

Table 1: Cohort for all ten jurisdictions and overall

Jurisdiction	Patients approached via PCP						Patient approached directly by registries/research teams														Total	
	Wales		England		Scotland		N Ireland		Denmark		Manitoba		Ontario		Sweden		Norway		Victoria			
	n	(%)	n	(%)	n	(%)	n	(%)	n	(%)	n	(%)	n	(%)	n	(%)	n	(%)	n	(%)	n	(%)
**Eligible patients [a, b]**	**1,811**	**(100)**	**2,517**	**(100)**	**1,366**	**(100)**	**620**	**(100)**	**539**	**(100)**	**980**	**(100)**	**4,080**	**(100)**	**493**	**(100)**	**1,318**	**(100)**	**859**	**(100)**	**14,583**	**(100)**
**Packs sent to PCP [c, d]**	**1,811**	**(99.7)**	**1,759**	**(69.9)**	**1,137**	**(83.2)**															**4,707**	**(82.7)**
pack not forwarded by PCP	547	(30.1)	255	(14.5)	201	(17.7)															1,003	(21.3)
unsure if pack forwarded by PCP	531	(29.2)	559	(31.8)	234	(20.6)															1,324	(28.1)
**Patients contacted by PCP [c, d]**	**733**	**(40.4)**	**945**	**(53.7)**	**702**	**(61.7)**															**2,380**	**(50.6)**
**Patients approached directly [c]**							**614**	**(99.0)**	**539**	**(100)**	**745**	**(76.0)**	**3,687**	**(90.4)**	**493**	**(100)**	**1,200**	**(91)**	**545**	**(63.4)**	**7,823**	**(88)**
patient died							6	(1.0)	0	(0.0)	103	(13.8)	249	(6.8)	0	(0.0)	0	(0.0)	0	(0.0)	358	(4.6)
no address							0	(0.0)	0	(0.0)	9	(1.2)	255	(6.9)	0	(0.0)	0	(0.0)	0	(0.0)	264	(3.4)
Other							0	(0.0)	0	(0.0)	6	(0.8)	215	(5.8)	0	(0.0)	0	(0.0)	0	(0.0)	221	(2.8)
**Patient responses (% of eligible patients)[c]**	**223**	**(12.3)**	**261**	**(10.4)**	**235**	**(17.2)**	**226**	**(36.5)**	**333**	**(61.8)**	**205**	**(20.9)**	**572**	**(14.0)**	**217**	**(44)**	**146**	**(11.1)**	**213**	**(24.8)**	**2,631**	**(18)**
**Patient responses (% of contacted)[e]**	**223**	**(30.4)**	**261**	**(27.6)**	**235**	**(33.5)**	**226**	**(37.2)**	**333**	**(61.8)**	**205**	**(32.7)**	**572**	**(19.3)**	**217**	**(44)**	**146**	**(12.2)**	**213**	**(39.1)**	**2,631**	**(27.5)**
extra sample for local purpose	0	(0.0)	0	(0.0)	0	(0.0)	0	(0.0)	0	(0.0)	0	(0.0)	214	(37.4)	0	(0.0)	0	(0.0)	0	(0.0)	214	(8.1)
other	0	(0.0)	0	(0.0)	35	(14.9)	25	(11.1)	38	(11.4)	0	(0.0)	43	(7.5)	0	(0.0)	0	(0.0)	3	(1.4)	144	(5.5)
**Patient surveys submitted for analyses [f]**	**223**	**(100)**	**261**	**(100)**	**200**	**(85.1)**	**201**	**(88.9)**	**295**	**(88.6)**	**205**	**(100)**	**315**	**(55.1)**	**217**	**(100)**	**146**	**(100)**	**210**	**(98.6)**	**2,273**	**(86.4)**
excluded for analyses – total	12	(5.4)	9	(3.4)	2	(1.0)	1	(0.5)	10	(3.4)	3	(1.5)	27	(8.6)	6	(2.8)	58	(39.7)	2	(1.0)	130	(5.7)
- previous cancer	0	(0.0)	5	(1.9)	0	(0.0)	0	(0.0)	0	(0.0)	0	(0.0)	3	(1.0)	0	(0.0)	0	(0.0)	0	(0.0)	8	(0.4)
- unknown date of consent or diagnosis	0	(0.0)	0	(0.0)	0	(0.0)	0	(0.0)	5	(1.7)	0	(0.0)	1	(0.3)	6	(2.8)	4	(2.7)	0	(0.0)	16	(0.7)
- consent too late/too early	12	(5.4)	4	(1.5)	2	(1.0)	1	(0.5)	5	(1.7)	3	(1.5)	22	(7.0)	0	(0.0)	33	(22.6)	2	(1.0)	84	(3.7)
- other	0	(0.0)	0	(0.0)	0	(0.0)	0	(0.0)	0	(0.0)	0	(0.0)	1	(0.3)	0	(0.0)	21	(14.4)	0	(0.0	22	(1.0)
**Patients included in analyses [i] (% of forwarded surveys)**	**211**	**(94.6)**	**252**	**(96.6)**	**198**	**(99.0)**	**200**	**(99.5)**	**285**	**(96.6)**	**202**	**(98.5)**	**288**	**(91.4)**	**211**	**(97.2)**	**88**	**(60.3)**	**208**	**(99.0)**	**2,143**	**(94.3)[h]**
**PCP surveys [j] (% of analysed patients)**	**133**	**(63.0)**	**196**	**(77.8)**	**149**	**(75.3)**	**181**	**(90.5)**	**218**	**(76.5)**	**109**	**(54.0)**	**93**	**(32.5)**	**n/a [h]**		**27**	**(30.7)**	**105**	**(50.5)**	**1,211**	**(56.6)[i]**
**Specialist surveys [k] (% of analysed patients)**	**98**	**(46.4)**	**153**	**(60.7)**	**106**	**(53.5)**	**n/a [g]**		**149 [g]**	**(52.3)**	**n/a [h]**		**62**	**(21.7)**	**n/a [h]**		**20**	**(22.7)**	**55**	**(26.4)**	**643**	**(37.0)[m]**

[a]Eligible as per protocol: individual aged 40 years or more, with cancer of the lung or bronchus (ICD-10 code: C34.0-C34.9; behaviour code ICD-O 3) but no synchronous primary cancer or prior history of lung cancer, alive at identification who completed consent to participate within nine months of diagnosis. [b]In some jurisdictions some 'eligible' patients had pre-opted out from being contacted and a small number where PCP information was not available. [c]Percentages of eligible patients. [d]Maximum of potentially contacted patients. i.e. sum of packs forwarded by PCP and packs unsure if forwarded by PCP. [e]Percentages of patients contacted by PCP (see note d) for Wales, England and Scotland or percentages of patients contacted directly by a registry; excl. non-accessible patients due to death or no patient addresses (all other jurisdictions). [f] Percentages of patient responses. [g] Data obtained from registries instead in N Ireland and Denmark. [h] Data not collected in this jurisdiction. [i] Denominator = total number of forwarded cases excl. patients not included in analytic sample in Ontario. [j] Denominator r = total number of analysed cases excl. patients from Sweden. [k] Denominator = total number of analysed cases excl. patients from Sweden. Manitoba & N Ireland

For peer review only - http://bmjopen.bmj.com/site/about/guidelines.xhtml

Baseline characteristics

Patient characteristics are detailed in Table 2. The cohort was predominantly White (95%), median age 70 years (IQR 64,75) with 82% reporting 'low' levels of education. Norway provided stage data classified as local, regional and distant which could not be converted to TNM stage data.

Ontario was the only jurisdiction with more female (65%) than male respondents. While self-reported health differed significantly, with Welsh patients (9%) reporting twice as high 'poor health' rates than English or Swedish (4%) and eight-fold that of Manitoba (2%), there was no difference in self-reported comorbidity rates. Even after the exclusion of Victoria, there was significant variation in early Stage (I/II) disease which ranged from 25% (Wales) to 59% (Ontario) and surgical resection rates which varied from 27% (Wales) to 58% (Ontario) (Table 2).

For peer review only

Table 2. The characteristics of eligible patients by jurisdictions and overall

	Wales (N=211)	England (N=252)	Scotland (N=198)	N Ireland (N=200)	Denmark (N=285)	Manitoba (N=202)	Ontario (N=288)	Sweden (N=211)	Norway (N=88)	Victoria (N=208)	Overall (N=2143)	p-value[6]
Date of diagnosis of first patient	03/04/2013	28/01/2013	26/04/2013	22/01/2013	16/05/2013	10/10/2012	08/10/2013	01/10/2013	16/01/2014	08/01/2013	10/10/2012	
Date of diagnosis of last patient	25/09/2014	14/08/2013	04/12/2013	02/12/2014	13/11/2013	27/03/2015	01/10/2014	30/05/2014	21/01/2015	28/12/2014	27/03/2015	
Interval from diagnosis date of first patient to last patient in months (recruitment period)	18	7	7	23	6	30	12	8	12	24	30	
Median (range) interval diagnosis to questionnaire completion in months	5 (0.6,9)	5 (3,9)	5 (1,9)	4 (0.1,9)	5 (2,8)	6 (4,9)	6 (4,9)	4 (3,8)	7 (5,9)	5 (0.2,8)	5 (0.1,9)	
**Age** years Median (IQR)	71 (65, 77)	71 (65, 77)	70 (64, 76)	69 (62, 75)	70 (63, 74)	70 (63, 77)	70 (64, 75)	70 (63, 75)	69 (64, 73)	68 (63, 73)	70 (64, 75)	0.010[1]
**Sex** n(%) Male	127(60)	134(53)	100(51)	105(53)	151(53)	100(50)	131(45)	111(53)	46(52)	112(54)	1117(52)	0.159[2]
**Health State** n(%) Good	135(64)	176(70)	131(66)	126(63)	190(67)	156(77)	220(76)	152(72)	50(57)	163(78)	1499(70)	<0.001*[1] <0.001**[2]
Fair	55(26)	59(23)	52(26)	51(26)	62(22)	41(20)	47(16)	49(23)	32(36)	33(16)	481(22)	
Poor	20(9)	11(4)	14(7)	16(8)	18(6)	4(2)	16(6)	9(4)	6(7)	10(5)	124(6)	
Missing	1(0.5)	6(2)	1(0,5)	7(4)	15(5)	1(0.5)	5(2)	1(0.5)	0(0)	2(1)	39(2)	
**Comorbidity**[3] n(%) No	92(44)	113(45)	98(49)	82(41)	111(39)	101(50)	136(47)	114(54)	35(40)	98(47)	980(46)	0.029*[1] 0.032**[2]
Medium	111(53)	129(51)	92(46)	103(52)	157(55)	93(46)	132(46)	87(41)	48(55)	100(48)	1052(49)	
High	7(3)	9(4)	8(4)	15(8)	11(4)	6(3)	18(6)	6(3)	5(6)	8(4)	93(4)	
Missing	1(0.5)	1(0.4)	0(0)	0(0)	6(2)	2(1)	2(0.7)	4(2)	0(0)	2(1)	18(0.8)	
**Education** n(%) Low	172(82)	217(86)	174(88)	166(83)	232(81)	170(84)	224(78)	159(75)	65(74)	181(87)	1760(82)	<0.001*[1] <0.001**[2]
High	11(5)	15(6)	11(6)	17(9)	15(5)	21(10)	55(19)	48(23)	14(16)	24(12)	231(11)	

For peer review only

	Wales (N=211)	England (N=252)	Scotland (N=198)	N Ireland (N=200)	Denmark (N=285)	Manitoba (N=202)	Ontario (N=288)	Sweden (N=211)	Norway (N=88)	Victoria (N=208)	Overall (N=2143)	p-value[6]
Missing	28(13)	20(8)	13(7)	17(9)	38(13)	11(5)	9(3)	4(2)	9(10)	3(1)	152(7)	
**Ethnicity** n(%) White	205(97)	249(99)	197(99)	196(98)	267(94)	173(86)	261(91)	n/a	87(99)	199(96)	1834(95)	<0.001*[2] <0.001**[2]
Asian	1(0.5)	0(0)	1(0.5)	0(0)	0(0)	12(6)	21(7)	n/a	1(1)	6(3)	41(2)	
Black	0(0)	1(0.4)	0(0)	0(0)	1(0.4)	0(0)	1(0.3)	n/a	0(0)	0(0)	3(0.2)	
Other	0(0)	0(0)	0(0)	0(0)	0(0)	15(7)	2(0.7)	n/a	0(0)	0(0)	17(0.9)	
Missing	5(2)	2(0.8)	0(0)	4(2)	17(6)	2(1)	3(1)	n/a	0(0)	3(1)	36(2)	
**Smoking** n(%) Never	13(6)	18(7)	12(6)	19(10)	15(5)	22(11)	31(11)	41(19)	11(13)	33(16)	215(10)	<0.001*[2] <0.001**[2]
Currently	19(9)	28811)	26(13)	41(21)	58(20)	24(12)	29(10)	29(14)	11(13)	9(4)	274(13)	
In the past	174(82)	204(81)	160(81)	137(69)	205(72)	156(77)	225(78)	139(66)	66(75)	165(79)	1631(76)	
Missing	5(2)	2(0.8)	0(0)	3(2)	7(2)	0(0)	3(1)	2(0.9)	0(0)	1(0.5)	23(1)	
**Tumour stage – TNM** n(%) I	26(12)	68(27)	49(25)	42(21)	74(26)	83(41)	133(46)	59(28)	3(3)	124(60)	661(31)	<0.001*[1] <0.001**[2]
II	27(13)	36(14)	32(16)	33(17)	26(9)	19(9)	38(13)	11(5)	5(6)	47(23)	274(13)	
III	64(30)	57(23)	56(28)	59(30)	84(29)	48(24)	54(19)	40(19)	5(6)	19(9)	486(23)	
IV	62(29)	80(32)	50(25)	62(31)	94(33)	48(24)	54(19)	94(45)	3(3)	13(6)	560(26)	
Missing	32(15)	11(4)	11(6)	4(2)	7(2)	4(2)	9(3)	7(3)	72(82)[4]	5(2)	162(8)	
**Tumour stage – TNM**[5] n(%) I/II	53(25)	104(41)	81(41)	75(38)	100(35)	102(51)	171(59)	70(33)	8(9)	*171(82)*	764(39)	<0.001*[2,5] <0.001**[2,5]
III/IV	126(60)	137(54)	106(54)	121(61)	178(62)	96(48)	108(38)	134(64)	8(9)	32(15)	1014(52)	
Missing	32(15)	11(4)	11(6)	4(2)	7(2)	4(2)	9(3)	7(3)	72(82)	5(2)	157(8)	
**Treatment Surgery** n(%) Yes	57(27)	107(42)	84(42)	65(33)	81(28)	113(56)	168(58)	65(31)	36(41)	199(96)	975(45)	<0.001*[2,5] <0.001**[2,5]
No	61(29)	56(22)	55(28)	94(47)	90(32)	44(22)	111(39)	79(37)	28(32)	5(2)	623(29)	
Missing	93(44)	89(35)	59(30)	41(21)	114(40)	45(22)	9(3)	67(32)	24(27)	4(2)	545(25)	
**Treatment Chemo** n(%) Yes	105(50)	125(50)	98(49)	93(47)	159(56)	93(46)	107(37)	133(63)	50(57)	63(30)	1026(48)	<0.001*[2,5] <0.001**[2,5]
No	41(19)	51(20)	46(23)	65(33)	45(16)	62(31)	172(60)	37(18)	18(20)	137(66)	674(32)	
Missing	65(31)	76(30)	54(27)	42(21)	81(28)	47(23)	9(3)	41(19)	20(23)	8(4)	443(21)	
**Treatment Radio** n(%) Yes	82(39)	68(27)	76(38)	72(36)	114(40)	81(40)	98(34)	70(33)	41(47)	29(14)	731(34)	<0.001*[2,5] <0.001**[2,5]
No	50(24)	72(29)	50(25)	77(39)	69(24)	71(35)	180(63)	69(33)	22(25)	155(75)	815(38)	
Missing	79(37)	112(44)	72(36)	51(26)	102(36)	50(25)	10(3)	72(34)	25(28)	24(12)	597(28)	
**Treatment Other** n(%) Yes	10(5)	14(6)	14(7)	11(6)	30(11)	18(9)	9(3)	0(0)	7(8)	16(8)	129(6)	<0.001*[2,5] <0.001**[2,5]

For peer review only - http://bmjopen.bmj.com/site/about/guidelines.xhtml

	Wales (N=211)	England (N=252)	Scotland (N=198)	N Ireland (N=200)	Denmark (N=285)	Manitoba (N=202)	Ontario (N=288)	Sweden (N=211)	Norway (N=88)	Victoria (N=208)	Overall (N=2143)	p-value[6]
No	48(23)	63(25)	45(23)	69(35)	255(89)	3(1)	261(91)	0(0)	2(2)	138(66)	884(41)	
Missing	153(72)	175(69)	139(70)	120(60)	0(0)	181(90)	18(6)	211(100)	79(90)	54(26)	1130(53)	

[1]Differences between jurisdictions were tested by the Kruskal-Wallis test, [2] Differences between jurisdictions were tested by the Pearson's Chi^2 test, [3]Comorbidity coded as none=no reported, medium=1-2 reported and high=3+ reported, [4] This included cases which could not be mapped as they were classified as per Cancer Registry of Norway into local (stage I), regional (stage II-III) and distant (stage IV) [5]Excluding Victoria, [6]Excluding Norway, *Missing category is excluded, **Missing category is included,
Abbreviations: IQR=inter-quartile range.

For peer review only

Routes to diagnosis

Results are detailed in Table 3. Over half (55%) were diagnosed following presentation to the PCP of whom 63% (range: 29% Norway - 82% Wales; data not shown) were urgently referred with a suspicion of cancer, based on the PCP questionnaire.

For peer review only

Table 3. Routes to diagnosis of lung cancer patients for each jurisdiction. All figures are n(%) unless otherwise stated.

	Wales (N=211)	England (N=252)	Scotland (N=198)	N Ireland (N=200)	Denmark (N=285)	Manitoba (N=202)	Ontario (N=288)	Sweden (N=211)	Norway (N=88)	Victoria (N=208)	Total (N=2143)
Symptoms prompting visit to PCP	109(52)	150(60)	128(65)	131(66)	170(60)	101(50)	91(32)	65(31)	35(40)	106(51)	1086(51)
Symptoms prompting emergency (A&E) department visit[1]	11(5)	18(7)	12(6)	22(11)	21(7)	25(12)	39(14)	18(9)	3(3)	6(3)	175(8)
Symptoms prompting visit to PCP and emergency (A&E) department[1]	10(5)	4(2)	7(4)	18(9)	8(3)	15(7)	12(4)	8(4)	5(6)	3(1)	90(4)
Incidental diagnosis in course of investigation/treatment for another problem[2]	68(32)	37(15)	36(18)	19(10)	55(19)	57(28)	116(40)	107(51)	32(36)	90(43)	617(29)
Unknown routes to diagnosis[3]	10(5)	17(7)	11(6)	5(3)	16(6)	4(2)	14(5)	11(5)	8(9)	0(0)	96(5)
Other[4]	3(1)	25(10)	3(2)	4(2)	14(5)	0(0)	16(6)	2(1)	5(6)	3(1)	75(3)
Missing	0(0)	1(0.4)	1(0.5)	1(0.5)	1(0.4)	0(0)	0(0)	0(0)	0(0)	0(0)	4(0.2)

[1] Emergency (A&E) route was ascribed when the patient reported pathway to cancer diagnosis involving going or being taken to the emergency department or the PCP reported that the patient presented to emergency department with or without their involvement [2] this could be by PCP, another doctor or via hospital; [3]includes cases where PCP or patient reported routes to diagnosis as 'Other' or 'Missing' but also reported symptoms or duration of symptoms, or date of first symptom, or waiting time for PCP appointment; [4]includes cases where PCP or patient reported routes to diagnosis as 'Other' and hasn't reported any symptoms or duration of symptoms, or date of first symptom, or waiting time for PCP appointment.

For peer review only

Symptoms prompting visit to physician

The median number of patient-reported symptoms were 2 (IQR 1,3). Across jurisdictions, the most common patient-reported symptoms were persistent cough (39%), breathlessness (37%) and fatigue (27%) although there was significant variation in proportion of patients presenting with individual symptoms (Table 4).

The PCPs reported a median of 1 (IQR 1,2) symptom at first presentation, with the most common being persistent cough (39%). Across jurisdictions, the reporting of other symptoms by the PCP was significantly lower compared to patients, especially fatigue (4%) and weight loss (8%). When the analysis was restricted to the cohort where both patient and PCP had completed the survey, this difference persisted. Unlike patients, there was minimal variation in PCP reporting of symptoms, with significant differences limited to 'no symptoms', 'other symptoms not previously listed' and weight loss. Overall 64% of symptoms were labelled as 'cancer specific' by the study PCPs (Table 4).

For peer review only - http://bmjopen.bmj.com/site/about/guidelines.xhtml

Table 4. Symptoms experienced by patients and presenting symptoms noted by PCP for eligible patients. All figures are n(%)

	Wales	England	Scotland	N Ireland	Denmark	Manitoba	Ontario	Sweden	Norway	Victoria	Overall	p[1]
**Symptoms (reported by patient)**	(N=211)	(N=252)	(N=198)	(N=200)	(N=285)	(N=202)	(N=288)	(N=211)	(N=88)	(N=208)	(N=2143)	
persistent cough	113(54)	123(49)	97(49)	83(42)	97(34)	71(35)	125(43)	84(40)	10(11)	39(19)	842(39)	<0.001
breathlessness	109(52)	126(50)	82(41)	73(37)	99(35)	63(31)	119(41)	77(36)	12(14)	24(12)	784(37)	<0.001
fatigue	75(36)	79(31)	64(32)	61(31)	38(13)	55(27)	92(32)	60(28)	17(19)	42(20)	583(27)	<0.001
weight loss	38(18)	39(15)	44(22)	37(19)	41(14)	29(14)	36(13)	34(16)	9(10)	20(10)	327(15)	0.147
felt sick /vomiting /nausea/loss of appetite	42(20)	33(13)	31(16)	24(12)	33(12)	22(11)	45(16)	28(13)	3(3)	20(10)	281(13)	0.132
coughing up blood-stained phlegm (sputum)	35(17)	32(13)	24(12)	31(16)	21(7)	16(8)	27(9)	25(12)	5(6)	23(11)	239(11)	0.014
chest or shoulder pain	23(11)	9(4)	18(9)	24(12)	10(4)	28(14)	43(15)	25(12)	4(5)	22(11)	206(10)	<0.001
other symptoms not listed above	51(24)	80(32)	59(30)	54(27)	63(22)	40(20)	30(10)	75(36)	26(30)	42(20)	520(24)	<0.001
no symptoms	29(14)	24(10)	32(16)	21(11)	63(22)	50(25)	67(23)	36(17)	33(38)	75(36)	430(20)	<0.001
missing	1(0.5)	16(6)	6(3)	17(9)	31(11)	9(4)	4(1)	4(2)	12(14)	1(0.5)	101(5)	<0.001
**Presenting symptom (reported by PCP)**	(N=85)	(N=133)	(N=115)	(N=0)	(N=151)	(N=86)	(N=61)	(N=0)	(N=17)	(N=81)	(N=729)	
persistent cough	33(39)	57(43)	45(39)	n/a	67(44)	24(28)	18(30)	n/a	7(41)	33(41)	284(39)	0.093
breathlessness	17(20)	27(20)	20(17)	n/a	27(18)	11(13)	11(18)	n/a	4(24)	13(16)	130(18)	0.803
fatigue	1(1)	5(4)	3(3)	n/a	9(6)	2(2)	2(3)	n/a	1(6)	3(4)	26(4)	0.539[2]
weight loss	5(6)	9(7)	15(13)	n/a	19(13)	3(3)	2(3)	n/a	0(0)	5(6)	58(8)	0.027
felt sick /vomiting /nausea/loss of appetite	1(1)	1(0.8)	2(2)	n/a	6(4)	2(2)	0(0)	n/a	0(0)	0(0)	12(2)	0.418[2]
coughing up blood-stained phlegm (sputum)	8(9)	10(8)	10(9)	n/a	7(5)	2(2)	3(5)	n/a	0(0)	7(9)	47(6)	0.305
chest or shoulder pain	8(9)	10(8)	15(13)	n/a	15(10)	1(1)	7(12)	n/a	1(6)	4(5)	61(8)	0.08
other symptoms not listed above	24(28)	44(33)	33(29)	n/a	64(42)	15(17)	7(12)	n/a	7(41)	20(25)	214(29)	<0.001

For peer review only - http://bmjopen.bmj.com/site/about/guidelines.xhtml

no symptoms	5(6)	6(5)	6(5)	n/a	2(1)	31(36)	12(20)	n/a	0(0)	10(12)	72(10)	<0.001
missing	7(8)	7(5)	11(10)	n/a	16(11)	12(14)	6(10)	n/a	2(12)	10(12)	71(10)	0.392
**Cancer-specificity of symptom presented**												
cancer-specific symptom	62(73)	97(73)	79(69)	n/a	94(62)	36(42)	38(62)	n/a	10(59)	48(59)	464(64)	<0.001
non-specific symptom	11(13)	23(17)	19(17)	n/a	39(26)	7(8)	5(8)	n/a	5(29)	13(16)	122(17)	
no symptoms /missing	12(14)	13(10)	17(15)	n/a	18(12)	43(50)	18(30)	n/a	2(12)	20(25)	143(19)	

[1] Differences between jurisdictions (excluding Victoria and Norway) were tested by the Pearson's Chi^2 test, if nothing else stated.
[2] Differences between jurisdictions (excluding Victoria and Norway) were tested by the Fisher's exact test.

For peer review only

Time intervals

The observed time intervals are shown in Appendix C2 and are summarised in Figure 2. Based on overall trends, jurisdictions could be grouped into those with reduced, longer and similar intervals to Wales (Table 5). It was not possible to interpret variations observed in Norway (small sample size) and Victoria (surgical cohort). In the remaining jurisdictions, there was no difference in the median adjusted patient interval compared to Wales. Denmark had shorter median adjusted primary care interval (-11 days); Sweden had shorter (-20) and Manitoba longer (+40) diagnostic intervals compared to Wales. Denmark (-13), Manitoba (-11), England (-9) and Northern Ireland (-4) had shorter treatment intervals. The median adjusted total interval was shorter only in Denmark (Table 5, Figure 2). The differences were greater for the 90th percentile. The total interval in patients who waited longest (90th percentile) was significantly shorter in two jurisdictions (Denmark -142, England -28 days) compared to Wales.

Table 5. Differences in adjusted intervals (days) between Wales and the other nine jurisdictions for lung cancer patients

Intervals	percentiles	Wales	Denmark	Sweden	England	N Ireland	Scotland	Manitoba	Ontario	Norway	Victoria
		Reference in days	Overall trend - shorter intervals			Similar with some intervals longer, some shorter		Overall trend - longer intervals		Difficult to interpret (see text for reasons)	
Ranking by 5-year survival rates for lung cancers diagnosed in 1999-2007 [5]		**10**	**6**	**1**	**9**	**7**	**8**	**3**	**2**	**5**	**4**
Patient Interval	Number of patients	181	233	172	213	179	169	133	205	55	141
	50th percentile (95% CI)	21	-6 (-13,0)	-1 (-9,8)	-3 (-10,5)	-3 (-13,6)	-3 (-10,4)	1 (-8,10)	1 (-11,14)	0 (-8,8)	**-9** (-16,-2)
	75th percentile (95% CI)	61	-13 (-38,13)	-2 (-24,21)	-2 (-42,37)	-8 (-55,39)	1 (-25,27)	3 (-23,30)	-7 (-54,39)	-9 (-60,42)	-4 (-46,38)
	90th percentile (95% CI)	216	**-34** (-55,-13)	-1 (-25,23)	-15 (-42,12)	24 (-21,70)	**43** (7,79)	**-35** (-59,-10)	**-34** (-66,-2)	**59** (21,96)	**-35** (-49,-21)
Primary Care interval	Number of patients	110	159	N/A	147	124	119	80	75	19	89
	50th percentile (95% CI)	20	**-11** (-18,-3)	N/A	-7 (-17,3)	-5 (-15,4)	-3 (-14,8)	7 (-8,21)	5 (-9,19)	**-11** (-18,-4)	-8 (-17,1)
	75th percentile (95% CI)	43	**-29** (-47,-12)		-17 (-42,8)	1 (-45,48)	-11 (-36,14)	19 (-47,85)	20 (-72,112)	-10 (-57,37)	-12 (-70,46)
	90th percentile (95% CI)	91	-30 (-66,7)		-39 (-85,6)	17 (-55,90)	-20 (-67,25)	13 (-38,65)	102 (-56,258)	-22 (-109,66)	-19 (-89,51)
Diagnostic interval	Number of patients	176	229	165	212	170	173	138	212	52	160
	50th percentile (95% CI)	45	-12 (-25,1)	**-20** (-35,-5)	9 (-3,21)	17 (-5,39)	-4 (-16,8)	**40** (14,66)	10 (-6,26)	4 (-16,24)	7 (-13,27)
	75th percentile (95% CI)	108	**-45** (-52,-39)	**-22** (-30,-15)	-7 (-20,7)	**12** (2,22)	-15 (-32,1)	**35** (22,48)	5 (-9,19)	-4 (-15,8)	-2 (-8,4)
	90th percentile (95% CI)	162	-27 (-153,99)	-34 (-206,138)	-14 (-100,72)	112 (-165,389)	31 (-81,143)	**112** (32,192)	106 (-122,335)	0 (-93,93)	**62** (10,114)
Treatment interval	Number of patients	192	279	190	238	200	187	182	263	87	199
	50th percentile (95% CI)	43	**-13** (-15,-11)	-2 (-8,3)	**-9** (-12,-5)	**-4** (-7,-2)	0 (-4,4)	**-11** (-17,-5)	3 (-4,10)	**-8** (-11,-6)	**-29** (-32,-27)
	75th percentile (95% CI)	64	**-32** (-36,-28)	-2 (-8,4)	**-18** (-23,-13)	**-13** (-18,-7)	1 (-7,9)	-5 (-16,6)	6 (-2,14)	**-13** (-19,-8)	**-33** (-41,-25)
	90th percentile (95% CI)	89	**-45** (-50,-40)	**-10** (-17,-4)	**-28** (-36,-20)	**-16** (-23,-9)	-6 (-14,1)	4 (-5,13)	4 (-4,13)	**-22** (-30,-14)	**-39** (-45,-32)
Total	Number of patients	147	192	147	176	153	143	117	178	52	113

interval	50th percentile (95% CI)	116	**-49** (-95,-3)	-8 (-64,47)	-7 (-52,38)	-16 (-41,10)	-2 (-70,66)	11 (-41,63)	9 (-78,97)	**-34** (-56,-12)	-32 (-64,2)
	75th percentile (95% CI)	204	-91 (-270,87)	-17 (-40,7)	-29 (-175,118)	5 (-191,201)	33 (-144,211)	13 (-77,103)	-7 (-331,317)	-39 (-107,29)	-23 (-61,14)
	90th percentile (95% CI)	365	**-142** (-150,-134)	-18 (-59,23)	**-28** (-37,-18)	0 (-4,5)	15 (-26,55)	15 (-26,55)	0 (-78,79)	**-84** (-119,-49)	0 (-3,3)

**Interval relative to Wales**	**Trends**	**Significant**
Reduction		
Increase		

The majority of patients were diagnosed between 2013-14. The differences were calculated for the 50th, 75th and 90th percentiles by setting age to its mean value and gender and comorbidity to their modes (i.e. male gender and medium comorbidity). It is not possible to interpret differences observed for Norway (due to the small sample size) and Victoria (the cohort was limited to those who had undergone surgery).

FIGURE 2

Sensitivity and validity analyses

The estimates of routes to diagnosis, time intervals, and regression analysis trends were not significantly altered by changing the cut-off to 6 months or using only patient data (results not shown).

Appendix C3 details, which sources were used based on the standardization rules, to define dates and also how often a day in the date was imputed. With regards to the dates of first presentation to healthcare (CCC=0.91), diagnosis (CCC$\geq$ 0.93) and treatment (CCC=0.94), there was adequate agreement between all data sources where the data on these dates was collected. Agreement between patient versus PCP for dates of first presentation to healthcare (CCC=0.91) and diagnosis (CCC=0.93) was also adequate as was agreement between patient versus CTS for dates of diagnosis (CCC=0.94) and treatment (CCC=0.94).

Omitting time intervals which were negative or over 365 days (Appendix C4) led to change in direction of difference which was non-significant in long intervals (75th or 90th percentile) between Wales and jurisdictions in four cases: Norway and Victoria (patient interval), N Ireland (diagnostic interval), England (total interval). All other results were similar to the main results.

Discussion

Main findings

This is the first international study we are aware of comparing lung cancer routes and time intervals. With the exception of Denmark, in all other jurisdictions, the median total interval

For peer review only - http://bmjopen.bmj.com/site/about/guidelines.xhtml

from symptom onset to treatment, for respondents diagnosed in 2012-15 was similar to that of Wales, the reference. However, there were jurisdiction specific differences in patient, diagnostic and treatment intervals, especially for the 10% of patients who waited the longest. Based on overall trends, jurisdictions could be grouped into those with trends of reduced, longer and similar intervals to Wales.

Across jurisdictions, all symptoms other than persistent cough were less frequently reported by the PCP when compared to patients. This was especially true for fatigue and weight loss. One in four patients reported incidental diagnosis and one in ten were diagnosed following a visit to the emergency (A&E) department.

Strengths and weaknesses

Our study helps address the shortcomings of current international comparisons across multiple national studies with significant variation in methodology including differences in definition of intervals. Strengths of our study include 1) use of the same methodology across countries 2) use of cancer registries to identify consecutive newly diagnosed patients; 3) use of standardised questionnaires; 4) inclusion of PCP and CTS questionnaires enriched by registry data; 5) minimal data interpretation by the local teams with all data cleaning performed in a standardised manner centrally; and 6) triangulation with comprehensive data rules to ensure validity, consistency and preserve statistical precision.[21] Recall bias was minimised by the triangulation of different data sources and by patients completing the questionnaire within a limited time window (median 5 months) after the cancer diagnosis.

A key limitation, as with all questionnaire-based studies was both selection and non-response bias which varied across jurisdictions and has implications for interpretation and generalisation of findings [34]. In comparing intervals, we adjusted for age, sex and comorbidity but were unable to adjust for ethnicity and education due to different classification systems. Recall bias was minimised by the triangulation of different data sources and by patients completing the questionnaire within a limited time window (median 5 months) after the cancer diagnosis. Recruitment of patients up to 9 rather than 6 months after diagnosis might have magnified the selection bias due to high mortality.[35] However, sensitivity analysis suggests that this did not impact on the results. Categorising presenting symptoms into indicative or not was done pragmatically as existing guidelines for lung cancer investigation vary across ICBP jurisdictions.[36] In Norway and Victoria, a small sample size and restriction of eligibility to only surgical patients, respectively, made comparison difficult. Nonetheless, significant differences in these two jurisdictions compared to Wales were largely limited to the treatment interval alone.

For peer review only - http://bmjopen.bmj.com/site/about/guidelines.xhtml

There was variation in stage distribution across jurisdictions. While this may be partly related to the varying response rate, true differences in lung cancer stage have been noted on analysis of registry data of patients diagnosed between 2004-2007.[6] The high lung cancer mortality and self-selection are likely to have contributed to an over-representation of early stage disease and tumours treated with surgical resection. This suggests that true variation may well be higher than that reported in this cohort of 'healthier early stage' patients.

Comparison to other studies

The most common patient-reported symptoms, in keeping with the literature, were persistent cough, breathlessness, fatigue and weight loss, with one in five reporting 'no symptoms'.[18] Only a minority (11%) of our respondents reported coughing blood or bloody sputum/spit, which is the only consistent predictor of lung cancer.[37] While haemoptysis was reported in a prospective survey (England 2011-12) by 22% of lung cancer patients identified through respiratory clinics, it was a presenting symptom in only 5% of cases.[11]

The median number of symptoms reported by patients was more than that reported by the PCP in all jurisdictions. This was especially so for fatigue and weight loss. A number of factors could have contributed to this - patients not listing all symptoms at presentation, patients having a different understanding/recall of their symptoms post diagnosis, PCPs only recording key symptoms such as cough. Further research on under reporting of systemic symptoms such as fatigue and weight loss is warranted.

As lung cancer mortality is higher in patients attending emergency (A&E) departments, the rates are often compared in an attempt to understand international survival differences.[38] The rate of respondents who attended A&E varied two-fold across jurisdictions from 9-10% in England, Scotland and Denmark to 18-20% in Northern Ireland, Ontario and Manitoba. While rates for Scotland (10%) were similar to that reported in a prospective Scottish audit (11.5%), as were rates for Denmark (7% vs 6.3% when PCP not involved), rates for England (9%) were lower than those reported in population based audits (25%) reflecting non-response bias.[14,15] In Victoria (4%) restriction of the cohort to surgical patients is likely to have accounted for the very low rates.

Our reported median patient, primary care and diagnostic intervals are in keeping with those previously reported from the participating jurisdictions (Table 6). Minor variations in interval estimates are likely due to differences in data source, sample size and cohort characteristics.[39] Longer intervals were reported from earlier cancer cohorts - median

For peer review only - http://bmjopen.bmj.com/site/about/guidelines.xhtml

primary care interval for England of 52 days in 1998-2000 (our median 11),[13] median total interval for Denmark of 108 days in 2004-5 (our median 67) and Norway of 118 in 2002-5 (our median 79).[17-19,24]

For peer review only

For peer review only - http://bmjopen.bmj.com/site/about/guidelines.xhtml

Table 6. Summary of literature on intervals in lung cancer patients diagnosed since 2000 in the ICBP Module 4 countries

Study No	Ref	Study Period	Jurisdiction	Design	Patients	No. of lung cancer patients	Interval[1] (days)				Total interval
							Patient	Primary care	Diagnostic	Treatment	
1	Walter FM et al (2015)	2011-2	England, UK	Prospective patient questionnaire survey - multi hospital cohort. Dates of diagnosis based on medical note review.	All attending urgent and routine respiratory clinics across the five hospitals in England aged over 40 years with symptoms suspicious of lung cancer.	153	Interval from first symptom to diagnosis Median 91 (IQR 49-184)				
2	Lyratzopoulos G et al (2015)	2009-10	England, UK	Prospective national audit of cancer diagnosis using primary practice patient records and continuous sampling during audit period	All patients aged ≥15 years who had first presented to a primary care practitioner and were subsequently diagnosed with one of 28 cancers	1128	Median 11 (IQR 0-32)	Median 3 (IQR 14-39)			
3	Neal R et al (2014)	2007–8	UK	Retrospective analysis of electonic health record data from General Practice Research Database - population cohort	All newly diagnosed with one of 15 cancers	2851			Median 112 (IQR 45-251)		
4	Barrett J et al (2008)	1998-2002	Exeter, England, UK	Retrospective case-control review of PCP records - population cohort	All with lung cancer aged ≥ 40 years, identified from the hospital cancer registry and computerised searches of all primary care practices	247		Median 52 (IQR 7–243)			
							Interval from first symptom to diagnosis - Median 121 (IQR 53,261)				
5	Baughan P et al (2009)	2005-6, 2007-8	Scotland, UK	Retrospective audit involving PCP review of medical records of all newly diagnosed cancer patients they had seen - population cohort	All newly diagnosed with lung cancer	981	Median 9.5 (IQR 31)		Median 11 (IQR 28)		
6	Guldbrandt LM et al (2015)	2010	Denmark	Retrospective PCP questionnaires survery of national registry-based population cohort	All consecutive newly diagnosed lung cancer patients	429-442 depending on interval		Median 7 (IQR, 0-30)	Median 29 (IQR, 12-69)		
7	Tørring ML et al (2013)	2004 - 5	Aarhus, Denmark	Prospective, population-based study using electronic health records and PCP survey of identified patients	All newly diagnosed with lung cancer after attending primary care	262			Median 112 (IQR 45-251)		
8	Hansen RP et al (2011)	2004-5	Aarhus, Denmark	Retrospective PCP survey	Cancer patients newly diagnosed during a 1-year period identified using administrative registry data	128-251 (depending on interval)	Median 28 (IQR 7-56)	Median 0 (IQR 0-9)		Median 51 (IQR 27-76)	Median 108 (IQR 82-167)
9	Bjerager M et al (2006)	2003	Aarhus, Denmark	Retrospective PCP survey using structured telephone interviews enriched with administrative registry data - population-based cohort	All lung cancer patients identified through histological and cytological tests from county-based registers	84		Median 32.5 (IQR 12–68)			
10	Rolke HB et al (2006)	2002-5	Norway (South)	Retrospective questionnaire-based patient survey - hospital cohort	All newly diagnosed with lung cancer	273 -376 (depending on interval)	Median 19 (2–77)				Median 118 (IQR 68–220)
11	Stokstad T et al (2017)	2011-13	Norway	Retrospective medical record audit -single hospital cohort	All cases that started diagnostic work-up and were diagnosed with lung cancer at St. Olavs Hospital, Trondheim	449				42 days (range: 2–296)	
12	Largey G et al (2016)	2013	Victoria, Australia	Retrospective medical record audit -three hospital cohort	Admitted with a new diagnosis of lung cancer over a three month period in three hospitals	78				Mean 30.4 (SD 45.3)	
13	Evans SM et al (2016)	2011-4	Victoria, Australia	Retrospective medical record audit - multi hospital cohort	All lung cancer patients newly diagnosed in six public and two private hospitals	1417			Median 15 (IQR, 5–36)	Median 30 (IQR, 6–84)	
14	Emery JD et al (2017)	2012 -4	Western rural Australia	Prospective cluster randomised trial of symptom awareness	Lung cancer patients newly diagnosed in the control arm of the trial	167	Interval from first symptom to diagnosis Median 34.5 (IQR 7 103.5)				

For peer review only

15	Burneister BH et al (2010)	2000-4	Queensland Australia	Retrospective analysis of radiation therapy waiting times	All lung cancer patients who received radiation therapy as initial treatment at a public hospital.	1535					Median 33[2]
16	Ellis PM and Vandermeer R (2011)	2010	Ontario, Canada	Retrospective patient survey using structured telephone interviews - single centre cohort. Appointment dates and diagnostic tests verified through family doctor or patient chart review.	All lung cancer patients referred to a regional cancer centre	52		Median 21	Median 27 (IQR 0-38)		Median 138 (IQR 79-175)
17	Lo DS et al (2007)	2005-7	Ontario, Canada	Retrospective medical record audit - multi hospital cohort	All with lung cancer seen on a newly implemented Time to Treat Program	144			Median interval from suspicion of lung cancer to diagnosis 37		

[1] Intervals as defined in Figure 1; [2] Limited to patients receiving radiation treatment

For peer review only

Across all jurisdictions, there was no significant difference in primary care intervals for the 10% of patients with longest interval. It is likely that these patients had vague or non-specific symptoms and signs. Referral guidelines for suspected lung cancer do not always favour patients with early symptoms and often prioritise those with more advanced disease.[40] Access to better diagnostic tools such as low-dose CT chest in the primary care setting may favour this group of patients.[41] It would be useful in future projects to explore whether such access may have contributed to the improved 1-year lung cancer survival rates reported from Australia and Canada.[6]

Diagnostic intervals were significantly longer for Manitoba compared to other jurisdictions and twice that reported in an ongoing local PCP audit (personal communication). While one might suspect overestimation due to differences in the source of date of first presentation, between our study (in almost half, it was derived from patients) and local audit, this is less likely as the concordance coefficient between PCP and patient derived data at Manitoba was 0.94.

Observed median treatment intervals were below 6 weeks for nearly all jurisdictions. This was the only interval where there were significant differences between jurisdictions with Denmark, England, Norway and Northern Ireland all having shorter adjusted treatment intervals across all percentiles, with larger differences for the 75th and 90th percentile. These improvements may reflect implementation of waiting time targets in Denmark (35-38 days from first consultation depending on treatment modality) and the UK (31 days from decision to treat).[42,43] The shorter treatment intervals in Norway are in keeping with long-standing provision of standardized cancer care pathways and effective coordination between primary care and treatment centers. While a systematic review did not find evidence to support an association between intervals and lung cancer outcomes, increasing mortality with longer diagnostic intervals was noted in a more recent, high-quality study.[16] In 2000, O'Rourke reported median intervals of 94 days (35-187) between the first hospital visit and starting treatment resulting in 21% of potentially curable patients becoming incurable.[44] Others have found metabolic evidence on PET/CT of pre-treatment disease progression in 21% and TNM upstaging in 18% of small-cell lung cancer patients after a relatively short median inter-scan interval of 43 days.[45] Long intervals can also result in deterioration in performance status. More recently, there is concern that the need for genotyping may result in further increase in time to treatment.

The shorter total interval in Denmark likely reflects the significant reductions in cancer waiting times following a collaborative effort to set-up and implement a national centralised quality

For peer review only - http://bmjopen.bmj.com/site/about/guidelines.xhtml

management system, the Danish Cancer Patient Pathways (CPPs). The latter includes PCP access to fast-track diagnostic work-up.[46]. The findings are in keeping with higher relative survival and lower mortality in Denmark among symptomatic cancer patients diagnosed through primary care after the implementation of CPPs and with the accelerated increase in 5-year survival among Danish lung cancer patients diagnosed in 2010-2014 when compared to patients from earlier time periods.[47] While there is some inherent lead-time bias, the findings highlight the importance and feasibility of a timely diagnosis of lung cancer.

Conclusions

The study provides for the first time, comparable data, collected through consistent methods in all jurisdictions, allowing for detailed comparisons of key diagnostic intervals in lung cancer and routes to diagnosis. While all jurisdictions except Denmark, had similar median adjusted total intervals, there were jurisdiction-specific significant differences in patient, diagnostic and treatment intervals, especially for the 10% of patients who waited the longest. The proportion of patients diagnosed following presentation to the PCP ranged from 35-75%. These data could help individual jurisdictions to better target their efforts to reduce time to treatment and ultimately improve patient experience and outcomes in lung cancer.'

Intervals and pathways are ultimately of interest as they relate to prognosis. A further analysis which includes all four cancers (lung, ovary, colon and breast) surveyed in ICBP4 module and explores the impact of these intervals on stage and 1-year survival is underway.

List of abbreviations

ICBP M4 – International Cancer Benchmarking Partnership Module 4

PCP – Primary Care Physician

CTS – Cancer Treatment Specialist

CPP – Danish Cancer Patient Pathways

Figure headings

Figure 1: Time intervals from onset of symptoms to start of treatment based on the Aarhus Statement

Figure 2: Differences in 50th, 75th and 90th centiles of the intervals (days) between Wales as the reference and the other nine jurisdictions. The data are adjusted for differences in age, gender and comorbidity. The bars in black show significant differences in intervals.

Acknowledgement

We are grateful to Catherine Foot, Martine Bomb, Brad Groves of Cancer Research UK for managing the programme. The ICBP Module 4 Working Group (below) for their support and

For peer review only - http://bmjopen.bmj.com/site/about/guidelines.xhtml

work keeping the study going. Christian Finley (Canada), David Baldwin (England) for reviewing the manuscript. Stefan Bergström, Jan Willem Coebergh, Jon Emery, Monique E van Leerdam, Marie-Louise Essink-Bot and Una MacLeod (the ICBP M4 Academic Reference Group) for providing independent peer review of the study protocol and analysis plan development. We thank all the patients, primary care physicians, cancer treatment specialists, and registry staff of all jurisdictions who took part in this study, and also the Danish Lung Cancer Registry for providing clinical information for the study.

ICBP Module 4 Working Group

Alina Zalounina Falborg, (Research Unit for General Practice, Department of Public Health, Aarhus University), Andriana Barisic (Department of Prevention and Cancer Control, Cancer Care Ontario) ,Anna Gavin (Northern Ireland Cancer Registry, Centre for Public Health, Queen's University Belfast), Anne Kari Knudsen (European Palliative Care Research Centre (PRC), Department of Oncology, Oslo University Hospital and Institute of Clinical Medicine), Breann Hawryluk (Department of Patient Navigation, Cancer Care Manitoba), Chantelle Anandan (Centre for Population Health Sciences, University of Edinburgh), Conan Donnelly (Centre for Public Health, Queen's University Belfast), David H Brewster, Scottish Cancer Registry, Information Services Division, NHS National Services Scotland , David Weller (Centre for Population Health Sciences, University of Edinburgh), Donna Turner (Population Oncology, Cancer Care Manitoba), Elizabeth Harland (Department of Epidemiology and Cancer Registry, CancerCare Manitoba), Eva Grunfeld (Knowledge Translation Research Network Health Services Research Program, Ontario Institute for Cancer Research; Professor and Vice Chair Research Department of Family and Community Medicine, University of Toronto), Evangelia Ourania Fourkala (Gynaecological Cancer Research Centre, Women's Cancer, Institute for Women's Health, University College London) , Henry Jensen (Research Unit for General Practice, Department of Public Health, Aarhus University), Irene Reguilon (International Cancer Benchmarking Partnership, Cancer Research UK), Jackie Boylan (Centre for Public Health, Queen's University Belfast), Jacqueline Kelly (Northern Ireland Cancer Registry, Centre for Public Health, Queen's University Belfast), Jatinderpal Kalsi (Gynaecological Cancer Research Centre, Women's Cancer, Institute for Women's Health, University College London), John Butler (The Royal Marsden) Kerry Moore, Centre for Public Health, Queen's University Belfast, Martin Malmberg (Department of Oncology, Lund University Hospital), Mats Lambe (Regional Cancer Center Uppsala and Department of Medical Epidemiology and Biostatics, Karolinska Institutet), Oliver Bucher (Department of Epidemiology and Cancer Registry, CancerCare Manitoba), Peter Vedsted, Research Unit for General Practice, Department of Public Health, Aarhus University, Rebecca-Jane Law (North Wales Centre for Primary Care Research, Bangor University), Rebecca Bergin (Centre for Behavioural Research in Cancer; Department of General Practice, University of Melbourne), Richard D Neal (North Wales Centre for Primary Care Research, Bangor University; Academic Unit of Primary Care, Leeds Institute of Health Sciences, University of Leeds), Samantha Harrison, Early Diagnosis and International Cancer Benchmarking Partnership, Policy and Information, Cancer Research UK, Sigrun Saur Almberg (Department of Cancer Research and Molecular Medicine, Faculty of Medicine, Norwegian University of Science and Technology (NTNU) , Therese Kearney (Northern Ireland Cancer Registry, Centre for Public Health, Queen's University Belfast), Victoria Cairnduff (Northern Ireland Cancer Registry, Centre for Public Health, Queen's University Belfast), Victoria Hammersley (Centre for Population Health Sciences, University of Edinburgh), Victoria White (Centre for Behavioural Research in Cancer, Cancer Council Victoria Road; School of Psychology Deakin University), Usha Menon (Gynaecological Cancer Research Centre, Women's Cancer, Institute for Women's Health, University College London), Yulan Lin (European Palliative Care Research Centre (PRC), Department of Oncology, Oslo University Hospital and Institute of Clinical Medicine)

For peer review only - http://bmjopen.bmj.com/site/about/guidelines.xhtml

ICBP Programme Board:

Aileen Keel (Scottish Government, Edinburgh, Scotland); Anna Boltong (Cancer Council Victoria, Carlton, Australia); Anna Gavin (Northern Ireland Cancer Registry, Queens University, Belfast, UK); David Currow (Cancer Institute New South Wales, Sydney, Australia); Gareth Davies (Wales Cancer Network, Cardiff, UK); Gunilla Gunnarsson (Swedish Association of Local Authorities and Regions, Stockholm, Sweden); Heather Bryant (Canadian Partnership Against Cancer, Toronto, Canada); Jane Hanson (Welsh Cancer National Specialist Advisory Group, Cardiff, UK); Kathryn Whitfield (Department of Health, Victoria Australia); Linda Rabeneck (Cancer Care Ontario, Toronto, Canada); Michael A Richards (Care Quality Commission, London, UK); Michael Sherar (Cancer Care Ontario, Toronto, Canada); Nicola Quin (Cancer Council Victoria, Carlton, Australia); Nicole Mittmann (Cancer Care Ontario, Toronto, Canada); Robert Thomas (Department of Health and Human Services, Victoria, Melbourne, Australia); Sara Hiom (Cancer Research UK); Sean Duffy (NHS England, London, UK); Chris Harrison (NHS England, London, UK); Søren Brostrøm (Danish Health and Medicines Authority, Copenhagen, Denmark); Stein Kaasa (University Hospital of Trondheim, Trondheim, Norway).

ICBP Academic Reference Group

Prof Jan Willem Coebergh, Professor of Cancer Surveillance, Department of Public Health, Erasmus Universiteit Rotterdam, Rotterdam, the Netherlands Jon Emery, Professor of Primary Care Cancer Research, University of Melbourne and Clinical Professor of General Practice, University of Western Australia, Australia Dr Stefan Bergström, Senior consultant oncologist, Department of Oncology, Gävle, Sweden Dr Monique E van Leerdam, Erasmus MC University Medical Centre, the Netherlands Prof Marie-Louise Essink-Bot, Academic Medical Centre, Amsterdam University, the Netherlands Prof Una MacLeod, Senior Lecturer in General Practice and Primary Care, Hull-York Medical School, United Kingdom

Author's contribution

UM, DW, PV, AZF, HJ planned the study design, data collection, carried out the analyses and wrote the draft manuscript. UM, PV, DW, HJ, AB, AKK, RJB, DHB, JK, VC, ATG, EG, EH, ML, RJW, YL, MM, DT, RDN, VW, IR and SH were responsible for local data collection (alongside the Working Group), management and interpretation, and have participated in writing and have approving the final manuscript version. JB, OB and OTB provided advice on the interpretation of results in their respective jurisdictions and comments or substantial edits on the manuscript, approving the final version.

For peer review only - http://bmjopen.bmj.com/site/about/guidelines.xhtml

Competing Interests

None

Data Availability statement

No additional data avaliable

Funding:

This work was supported by: CancerCare Manitoba; Cancer Care Ontario; Canadian Partnership Against Cancer (CPAC); Cancer Council Victoria; Cancer Research Wales; Cancer Research UK; Danish Cancer Society; Danish Health and Medicines Authority; European Palliative Care Research Centre (PRC), Norwegian University of Science and Technology (NTNU); Northern Ireland Guidelines Audit and Implementation Network (GAIN); Macmillan Cancer Support; National Cancer Action Team; NHS England; Northern Ireland Cancer Registry, funded by the Public Health Agency NI; Norwegian Directorate of Health; Research Centre for Cancer Diagnosis in Primary Care (CaP), Aarhus University, Denmark; Scottish Government; Swedish Association of Local Authorities and Regions; University College London and NIHR Biomedical Research Centre at University College London NHS Foundation Trust; University of Edinburgh; Victorian Department of Health and Human Services; Welsh Government. The funding bodies had no influence on the design of the study and collection, analysis, and interpretation of data, in writing the manuscript or whether to publish the results.

Availability of data and material

The data that support the findings of this study are available from the named authors from each ICBP jurisdiction, but restrictions apply to the availability of these data and so are not publicly available. Data are however available from the authors upon reasonable request and with permission of the ICBP Programme Board. Please contact the ICBP Programme Management team, based at Cancer Research UK, with any queries (icbp@cancer.org.uk).

Ethics approval and consent to participate

For each local data collection, there were specific procedures and approvals which included anonymised data transfer to University College London and Aarhus University. Approvals were received from the following institutions: Cancer Council Victoria Human Research Ethics Committee [HREC 1125]; Health Research Ethics Board, University of Manitoba [HS15227 (H2012:105)]; Research Resource Ethics Committee, CancerCare Manitoba [RRIC#28-2012];

For peer review only

University of Toronto Research Ethics Board [27881]; The Danish Data Protection Agency [2013-41-2030]; Swedish Ethics Review Board, Uppsala [2013/306]; Norway Regional committees for medical and health research ethics [2013/136/REK nord]; England, Wales and Scotland, NRES Committee East Midlands – Derby 2, local R&D for each health board [11/EM/0420]; Northern Ireland ORECNI Ethical approval, local governance for each health Trust [11/EM/0420].

For peer review only

References

1. Ferlay J, Steliarova-Foucher E, Lortet-Tieulent J, et al. Cancer incidence and mortality patterns in Europe: Estimates for 40 countries in 2012. Eur J Cancer 2013;49(6):1374-403.

2. Allemani C, Weir HK, Carreira H, et al. Global surveillance of cancer survival 1995-2009: analysis of individual data for 25 676 887 patients from 279 population-based registries in 67 countries (CONCORD-2). Lancet 2015;385(9972):977-1010.

3. Australian Institute of Health and Welfare 2012. Cancer survival and prevalence in Australia: period estimates from 1982 to 2010. Cancer Series no. 69. Cat. no. CAN 65. Canberra: AIHW https://www.aihw.gov.au/getmedia/da7bff73-5b78-4cf8-a719-5903a75fcc9a/13953.pdf.aspx?inline=true

4. Neal RD, Robbé IJ, Lewis M, Williamson I, Hanson J. The complexity and difficulty of diagnosing lung cancer: findings from a national primary-care study in Wales. Prim Health Care Res Dev 2015 Sep;16(5):436-49

5. Francisci S, Minicozzi P, Pierannunzio D, et al. Survival patterns in lung and pleural cancer in Europe 1999-2007: Results from the EUROCARE-5 study. Eur J Cancer 2015;51(15):2242-53.

6. Coleman MP, Forman D, Bryant H, et al. Cancer survival in Australia, Canada, Denmark, Norway, Sweden, and the UK, 1995-2007 (the International Cancer Benchmarking Partnership): an analysis of population-based cancer registry data. Lancet 2011;377(9760):127-38.

7. Olsson JK, Schultz EM, Gould MK. Timeliness of care in patients with lung cancer: a systematic review. Thorax. 2009;64(9):749-56

8. Malalasekera A, Nahm, S, Blinman PL, Kao SC, Dhillon H, VardyJ. How long is too long? A Scoping Review of Health System delays in lung cancer. European Respiratory Reviews. 2018; 29;27(149)

9. Jacobsen MM, Silverstein SC, Quinn M, Waterston LB, Thomas CA, Benneyan JC, et al. Timeliness of access to lung cancer diagnosis and treatment: A scoping literature review. Lung Cancer-J Iaslc. 2017;112:156-64.

10. Vinas F, Ben Hassen I, Jabot L, Monnet I, Chouaid C. Delays for diagnosis and treatment of lung cancers: a systematic review. Clin Respir J. 2016;10(3):267-

For peer review only - http://bmjopen.bmj.com/site/about/guidelines.xhtml

11. Walter FM, Rubin G, Bankhead C, et al. Symptoms and other factors associated with time to diagnosis and stage of lung cancer: a prospective cohort study. Br J Cancer 2015;112 Suppl 1:S6-13.

12. Neal RD, Din NU, Hamilton W, et al. Comparison of cancer diagnostic intervals before and after implementation of NICE guidelines: analysis of data from the UK General Practice Research Database. Br J Cancer 2014;110(3):584-92.

13. Barrett J, Hamilton W. Pathways to the diagnosis of lung cancer in the UK: a cohort study. BMC Fam Pract 2008;9:31.

14. Baughan P, O'Neill B, Fletcher E. Auditing the diagnosis of cancer in primary care: the experience in Scotland. Br J Cancer 2009;101 Suppl 2:S87-91.

15. Guldbrandt LM, Fenger-Gron M, Rasmussen TR, et al. The role of general practice in routes to diagnosis of lung cancer in Denmark: a population-based study of general practice involvement, diagnostic activity and diagnostic intervals. BMC Health Serv Res 2015;15:21.

16. Torring ML, Frydenberg M, Hansen RP, et al. Evidence of increasing mortality with longer diagnostic intervals for five common cancers: a cohort study in primary care. Eur J Cancer 2013;49(9):2187-98.

17. Hansen RP, Vedsted P, Sokolowski I, et al. Time intervals from first symptom to treatment of cancer: a cohort study of 2,212 newly diagnosed cancer patients. BMC Health Serv Res 2011;11:284.

18. Bjerager M, Palshof T, Dahl R, et al. Delay in diagnosis of lung cancer in general practice. Br J Gen Pract 2006;56(532):863-8.

19. Rolke HB, Bakke PS, Gallefoss F. Delays in the diagnostic pathways for primary pulmonary carcinoma in Southern Norway. Respir Med 2007;101(6):1251-7.

20. Largey G, Ristevski E, Chambers H, et al. Lung cancer interval times from point of referral to the acute health sector to the start of first treatment. Aust Health Rev 2016.

21. Evans SM, Earnest A, Bower W, et al. Timeliness of lung cancer care in Victoria: a retrospective cohort study. Med J Aust 2016;204(2):75 e1-9.

22. Ellis PM, Vandermeer R. Delays in the diagnosis of lung cancer. J Thorac Dis 2011;3(3):183-8.

For peer review only

23. Lo DS, Zeldin RA, Skrastins R, et al. Time to treat: a system redesign focusing on decreasing the time from suspicion of lung cancer to diagnosis. J Thorac Oncol 2007;2(11):1001-6.

24. Helsper CW, van Erp NF, Peeters PHM, de Wit NJ. Time to diagnosis and treatment for cancer patients in the Netherlands: Room for improvement? European Journal of Cancer 2017; (87) 113-121

25. Butler J, Foot C, Bomb M, et al. The International Cancer Benchmarking Partnership: an international collaboration to inform cancer policy in Australia, Canada, Denmark, Norway, Sweden and the United Kingdom. Health Policy 2013;112(1-2):148-55.

26. Weller D, Vedsted P, Anandan C, et al. An investigation of routes to cancer diagnosis in 10 international jurisdictions, as part of the International Cancer Benchmarking Partnership: survey development and implementation. BMJ Open 2016;6(7):e009641.

27. Weller D, Vedsted P, Rubin G, et al. The Aarhus statement: improving design and reporting of studies on early cancer diagnosis. Br J Cancer 2012;106(7):1262-7.

28. Jenkinson C, Coulter A, Wright L. Short form 36 (SF36) health survey questionnaire: normative data for adults of working age. Bmj. 1993 May 29;306(6890):1437-40

29. TNM classification of malignant tumours / edited by L.H. Sobin, M.K. Gospodarowicz, and Ch. Wittekind. — 7th ed. UICC International Union Against Cancer. ISBN 978-1-4443-3241-4

30. Koenker R, Bassett G. Regression quantiles. Econometrica 1978; 46:33-50

31. Machado JAF, Santos Silva JMC. Quantiles for Counts. J Amer Statistical Assoc 2005, 100(472): 1226-1237.

32. Miranda A. QCOUNT: Stata program to fit quantile regression models for count data. Boston: Boston College Department of Economics; 2006

33. Lin LI. A concordance correlation coefficient to evaluate reproducibility. Biometrics 1989;45(1):255-68.

34. Coxon D. The Aarhus statement on cancer diagnostic research: turning recommendations into new survey instruments. BMC Health Services Research 2018(18):677

35. Walters S, Maringe C, Coleman MP, et al. Lung cancer survival and stage at diagnosis in Australia, Canada, Denmark, Norway, Sweden and the UK: a population-based study, 2004-2007. Thorax 2013;68(6):551-64.

For peer review only

36. Nicholson BD, Mant D, Neal RD, et al. International variation in adherence to referral guidelines for suspected cancer: a secondary analysis of survey data. Br J Gen Pract 2016;66(643):e106-13.

37. Shim J(1), Brindle L, Simon M, George S. A systematic review of symptomatic diagnosis of lung cancer. Fam Pract. 2014 Apr;31(2):137-48

38. Hamilton W, Peters TJ, Round A, et al. What are the clinical features of lung cancer before the diagnosis is made? A population based case-control study. Thorax 2005;60(12):1059-65.

39. Beckett P, Tata LJ, Hubbard RB. Risk factors and survival outcome for non-elective referral in non-small cell lung cancer patients-analysis based on the National Lung Cancer Audit. Lung Cancer 2014;83(3):396-400.

40. Lim A, Mesher D, Gentry-Maharaj A, et al. Time to diagnosis of Type I or II invasive epithelial ovarian cancers: a multicentre observational study using patient questionnaire and primary care records. BJOG 2016;123(6):1012-20.

41. Neal RD, Allgar VL, Ali N, et al. Stage, survival and delays in lung, colorectal, prostate and ovarian cancer: comparison between diagnostic routes. Br J Gen Pract 2007;57(536):212-9.

42. Guldbrandt LM, Moller H, Jakobsen E, et al. General practice consultations, diagnostic investigations, and prescriptions in the year preceding a lung cancer diagnosis. Cancer Med 2017;6(1):79-88.

43. Danish National Board of Health. Pakkeforløb for lungekræft [Cancer Patient Pathway – Lung Cancer], 2016) National Board of Health, Copenhagen. Available at: https://www.sst.dk/da/sygdom-og-behandling/kraeft/pakkeforloeb/~/media/89192ECB2709401CAD8E4BBB0304691E.ashx [accessed 25 September 2017]

44. O'Rourke N, Edwards R. Lung cancer treatment waiting times and tumour growth. Clin Oncol (R Coll Radiol). 2000;12(3):141-4.

45. Wang J, Mahasittiwat P, Wong KK, et al. Natural growth and disease progression of non-small cell lung cancer evaluated with 18F-fluorodeoxyglucose PET/CT. Lung Cancer 2012;78(1):51-6.

46. Jensen H, Tørring ML,Vedsted P. Prognostic consequences of implementing cancer patient pathways in Denmark: a comparative cohort study of symptomatic cancer patients in primary care. BMC Cancer 2017;17:627.

For peer review only - http://bmjopen.bmj.com/site/about/guidelines.xhtml

47. Allemani C, Matsuda T, Di Carlo V, et al. Global surveillance of trends in cancer survival 2000–14 (CONCORD-3): analysis of individual records for 37 513 025 patients diagnosed with one of 18 cancers from 322 population-based registries in 71 countries. Lancet 2018 Mar 17;391(10125):1023-1075.

For peer review only

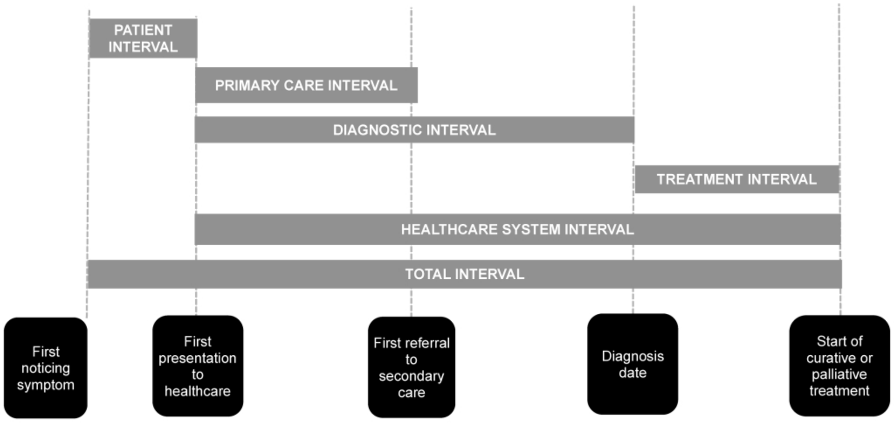

Time intervals from onset of symptoms to start of treatment based on the Aarhus Statement

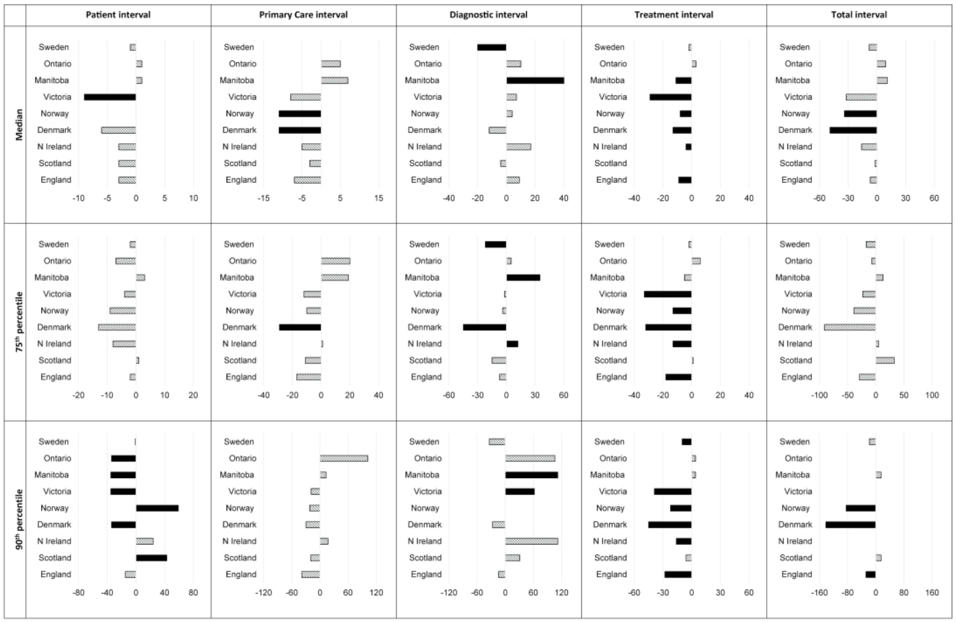

Differences in 50th, 75th and 90th centiles of the intervals (days) between Wales as the reference and the other nine jurisdictions. The data are adjusted for differences in age, gender and comorbidity. The bars in black show significant differences in intervals.

420x297mm (300 x 300 DPI)

Supplementary Web Appendix

Contents

Appendix A. Lung cancer questionnaires

A1: Patient questionnaire……………………………………………………………………………2

A2: Primary care physician (PCP) questionnaire………………………………………………...16

A3: Cancer treatment specialists (CTS) questionnaire ……………………………………………24

Appendix B . Rules for missing, incomplete, multiple response and out of range data…………………..…32

Appendix C . Supplementary Tables

C1: Classification of lung cancer -specific symptoms reported by patients into cancer specific and non- cancer specific…………………………………………………………………………………39

C2: Time intervals (days) depicted as median, 75th and 90th centiles for each of the ten jurisdictions. In Sweden, no data was available on primary care interval……………………………40

C3. Data sources used to define dates and percentage of imputed dates…….41

C4: Percentages of negative intervals set to 0, large intervals (>365 days) set to 365 days, and intervals based not on dates…………………………………………………………42

For peer review only - http://bmjopen.bmj.com/site/about/guidelines.xhtml

Appendix A: Lung cancer questionnaires

A1: Patient questionnaire

ICBP
International Cancer
Benchmarking Partnership

International Cancer Benchmarking Partnership Module 4

Patient questionnaire
Lung Cancer

Thank you very much for taking the time to fill in this questionnaire – it should take about 20 minutes to complete. We are sending the questionnaire to a large sample of people who we understand have had a diagnosis of lung cancer. If this has been sent to you in error and you do not have cancer, please do not continue and return the documents in the prepaid envelope.

Sample

Our aim is to gain a better understanding of the process by which people have their cancer diagnosed. We would also like to find out more about the symptoms they experience (if any), and the pathway they follow from start of symptoms to treatment of their cancer. This will help in identifying ways in which cancers can be diagnosed quickly and effectively. Thank you once again for your time.

This information is confidential and will not be passed to anyone involved in your treatment.

Name: ____________________

Date of Birth: ____________________

Address: ____________________

Copyright property of the International Benchmarking Partnership (ICBP)
and Module 4 collaborators. Usage must be authorised

ICBP
International Cancer Benchmarking Partnership

Consent form

Please read the consent form and sign your name and date **BELOW**.

If you require any clarification, please do not hesitate to ring the study team members. Their contact details are found on the information sheet.

Please be reassured that your responses are completely confidential and will not be passed to anyone involved in your treatment. For the purposes of the study it is important that you agree to consent to all the statements listed below.

- I confirm that I have read the attached information sheet and I understand why the research is being done.
- I am willing for the team to request information from my GP and hospital doctors which is relevant to the audit as described in the information sheet.
- I give permission for my details (name, address) to be given to the cancer registry (NHS Information Centre for Health and Social Care) for follow up.
- I agree for the information I have provided and any other relevant information from my medical records to be stored as described in the information sheet under the custodianship of University College London.
- I consent to sharing of coded data which contains no personal identifiers between researchers, some of whom are located outside the European Union.
- I consent for use of my data if I become mentally incapacitated during the course of the project.

I agree to all the statements listed and consent to participate in the study.

Name (Please print) ____________________

Signature: ____________________ Date: ____________________

If we have any questions, may we phone you for clarification? (Please tick) ☐ **Yes** ☐ **No**

If **Yes**, please provide your telephone number: ____________________

Sample

Copyright property of the International Benchmarking Partnership (ICBP) and Module 4 collaborators. Usage must be authorised

ICBP
International Cancer Benchmarking Partnership

1. **Please can you confirm the details of your GP/GP practice (name, practice address – as best as you can remember): We appreciate that you may have more than one GP involved in your care – in which case, we are interested in the GP you would say provides the majority of your care, particularly relating to the cancer you've had diagnosed.**

Name of doctor ________________

Name of practice ________________

Address ________________

____

____

Postcode ________________

Town ________________

Sample

Copyright property of the International Benchmarking Partnership (ICBP) and Module 4 collaborators. Usage must be authorised

ICBP
International Cancer Benchmarking Partnership

2. Which of the following best describes the events which led to your diagnosis of cancer? (please tick only **ONE** answer)

	✓
I had symptoms/I noticed a bodily change and went to see a doctor (e.g. GP)	
I had symptoms/I noticed a bodily change and went/was taken to Accident and Emergency (A&E)	
I had seen a doctor/GP with symptoms, but went/was taken to Accident and Emergency (A&E) when things worsened	
I was being investigated by my doctor(s) for another problem during which time the cancer was discovered	
Other (please describe):	

Sample

Copyright property of the International Benchmarking Partnership (ICBP) and Module 4 collaborators. Usage must be authorised

ICBP
International Cancer Benchmarking Partnership

3. The following health concerns or symptoms are commonly experienced with lung cancer.

Cough
Breathlessness
Being short of breath
Chest pain / Pain on breathing
Coughing up blood
Bloody sputum/blood in spit
Unexplained weight loss
Loss of appetite
Fatigue

Sample

Please write down ALL health concern(s) or symptom(s) you may have had before contacting a doctor or taking part in screening. It does not matter if they are not included in the list above:

Please write your health concern(s) or symptom(s) in the boxes below:
1)
2)
3)
4)
5)
6)

This is not applicable to me (e.g. I did not have any symptoms), please tick	

Copyright property of the International Benchmarking Partnership (ICBP) and Module 4 collaborators. Usage must be authorised

ICBP
International Cancer Benchmarking Partnership

4. Please write down your best estimate of the date you noticed the first of these health concern(s) or symptom(s). If you cannot remember the exact date, you can fill in the month and the year.

Day (optional), month, year

D	D	M	M	Y	Y	Y	Y

	✓
This is not applicable to me (e.g. I had no symptoms), please tick	

5. Approximately how long did you have health concern(s) or symptom(s) before contacting a doctor? (Please think of the first visit to the doctor, not re-visits after that). Please tick only **ONE** answer.

	✓
Less than 1 week	
1-2 weeks	
3-4 weeks	
5-7 weeks	
2-5 months	
6-12 months	
More than 12 months	

Sample

	✓
This is not applicable to me (e.g. I had no symptoms), please tick	

Copyright property of the International Benchmarking Partnership (ICBP) and Module 4 collaborators. Usage must be authorised

ICBP International Cancer Benchmarking Partnership

6a. Once you contacted a practice about your health concern(s) or symptom(s), how long did it take to get an appointment with a doctor? (Please think of the first visit to the doctor, to discuss your health concern(s) or symptom(s)).
Please tick only **ONE** answer.

	✓
Same day/next day	
Within 1 week	
1-2 weeks	
3-4 weeks	
Longer	
If there was no waiting time (e.g. you went/were taken to A&E), please tick this box	
This is not applicable to me (e.g. I had no symptoms), please tick	

Sample

6b. What was the date you first saw your doctor about your health concern(s) or symptom(s)? If you cannot remember the exact date, you can fill in the month and the year.

Day (optional), month, year

D	D	M	M	Y	Y	Y	Y

	✓
This is not applicable to me (e.g. I had no symptoms), please tick	

Copyright property of the International Benchmarking Partnership (ICBP) and Module 4 collaborators. Usage must be authorised

ICBP
International Cancer Benchmarking Partnership

7. How many times did you visit the following for the investigation of your symptoms before your cancer was diagnosed?

	Please write down the number of visits
GP	
Hospital	
Consultant/specialist outside of a hospital	

This is not applicable to me (e.g. I had no symptoms), please tick	

8a. After your doctor referred you to a specialist, how long did it take you to get an appointment? Please tick only **ONE** answer.

Sample

Less than 1 week	
1-2 weeks	
3-4 weeks	
5-7 weeks	
2-5 months	
6-12 months	
More than 12 months	

This is not applicable to me (e.g. my doctor did not refer me), please tick	

Copyright property of the International Benchmarking Partnership (ICBP) and Module 4 collaborators. Usage must be authorised

ICBP
International Cancer Benchmarking Partnership

8b. What was the date of your first appointment with a doctor, involved in investigating and/or treating your cancer, to whom you were referred? If you cannot remember the exact date, you can fill in the month and the year.

Day (optional), month, year

D	D	M	M	Y	Y	Y	Y

This is not applicable to me (e.g. my doctor did not refer me), please tick	✓

9. What was the date you were told you had cancer? If you cannot remember the exact date, you can fill in the month and the year.

Day (optional), month, year

D	D	M	M	Y	Y	Y	Y

Sample

Copyright property of the International Benchmarking Partnership (ICBP) and Module 4 collaborators. Usage must be authorised

ICBP
International Cancer Benchmarking Partnership

10. Have you had any of the following treatments for your cancer yet? If so, please can you estimate the date this treatment started? Please tick **ALL** that apply. If you cannot remember the exact date, you can fill in the month and the year.

	Type of treatment		Date of treatment (give first date if you had more than one)
a.	Surgery	☐ Yes<br>☐ No	Day (optional), month, year<br>D D M M Y Y Y Y
b.	Chemotherapy	☐ Yes<br>☐ No	Day (optional), month, year<br>D D M M Y Y Y Y
c.	Radiotherapy	☐ Yes<br>☐ No	Day (optional), month, year<br>D D M M Y Y Y Y
d.	Other<br>Please specify:	☐ Yes<br>☐ No	Day (optional), month, year<br>D D M M Y Y Y Y
e.	Treatment not started yet	☐ Yes	

Sample

Copyright property of the International Benchmarking Partnership (ICBP) and Module 4 collaborators. Usage must be authorised

ICBP
International Cancer Benchmarking Partnership

11. Who is the consultant doctor who has taken responsibility for diagnosing and or/treating your cancer?

Name of consultant:
Hospital name:
Hospital department:

Please can you answer some more general questions about your health?
It will help us in interpreting your responses to this questionnaire to know about your general health and other health problems you may have had in the past.

12. Looking back to the 2 years before you were diagnosed with cancer, would you say your general health was (Please tick only **ONE** answer.): ✓

Sample

Very good	
Good	
Fair	
Poor	
Very poor	

13. Have you been treated before for any of the conditions below?
Please tick 'yes' or 'no' for each condition:

Heart disease	☐ **Yes**	☐ **No**
Stroke	☐ **Yes**	☐ **No**
Lung disease (excluding lung cancer)	☐ **Yes**	☐ **No**
Diabetes	☐ **Yes**	☐ **No**

Copyright property of the International Benchmarking Partnership (ICBP)
and Module 4 collaborators. Usage must be authorised

Finally, a little more information about you. The information you provide below will help us to analyse the results of the survey in more detail.

14. Which of these best describes your ethnic group? (please tick one box, as appropriate). If you are descended from more than one ethnic or racial group, please tick the group you consider you belong to, or tick 'any other ethnic group'.

	✓		✓		✓		✓
White		Chinese		Black - Caribbean		Black - African	
Black - other		Indian		Pakistani		Bangladeshi	
Any other ethnic group, please specify:							

Sample

15. What is the main language spoken in your home? Please tick

	✓
English	
Other, please specify:	

16. What is the highest level of education you have achieved?
Please tick only **ONE** answer.

	✓
Finished school at or before the age of fifteen	
Completed GCSEs, O-levels or equivalent	
Completed A Levels or equivalent	
Completed further education but not a degree	
Completed a Bachelor's degree / Masters degree / PhD	
Other, please specify:	

Copyright property of the International Benchmarking Partnership (ICBP) and Module 4 collaborators. Usage must be authorised

ICBP
International Cancer Benchmarking Partnership

17. **Have you ever smoked cigarettes, including hand-rolled ones, pipes or cigars?**

☐ Yes ☐ No

18. **Are you a current smoker, smoking either cigarettes, including hand-rolled ones, pipes or cigars?**

☐ Yes ☐ No

19. **If you are a current smoker or have smoked in the past, how many cigarettes, including hand-rolled ones, pipes or cigars on average do you smoke/have you smoked per day?**

Number per day:	

Sample

Copyright property of the International Benchmarking Partnership (ICBP) and Module 4 collaborators. Usage must be authorised

ICBP
International Cancer Benchmarking Partnership

20. Further comments

Please add anything else that you would like to tell us about your cancer diagnosis or treatment.

Sample

Thank you very much for taking the time to complete this questionnaire.

Copyright property of the International Benchmarking Partnership (ICBP) and Module 4 collaborators. Usage must be authorised

A2: Primary care physician (PCP) questionnaire

ICBP
International Cancer
Benchmarking Partnership

International Cancer Benchmarking Partnership Module 4

Primary Care Audit
Lung Cancer

Thank you very much for agreeing to fill in this questionnaire. As part of an international study examining differences in cancer survival, we are sending the questionnaire to health care providers of a sample of consented patients with cancer.

Our aim is to gain a better understanding of the process by which people have their cancer diagnosed – the symptoms they experience, and the pathway they follow from onset of symptoms to treatment of their cancer. This will help in identifying ways in which cancers can be diagnosed and treated quickly and effectively. Thank you once again for your time.

Sample

Please can you refer to your patient's notes in completing the questionnaire as this will help in obtaining accurate data on time points.

If you would prefer to return this questionnaire without the patient details, please tear off along the dotted line

ID-number: Jurisdiction-ID + Patient-ID: ______________________

Copyright property of the International Benchmarking Partnership (ICBP) and Module 4 collaborators. Usage must be authorised

ICBP
International Cancer Benchmarking Partnership

Sample

Patient information

ID-number: Jurisdiction-ID + Patient-ID:

Full name:

Address:

Postcode:

Date of birth: D D M M Y Y Y Y

Copyright property of the International Benchmarking Partnership (ICBP) and Module 4 collaborators. Usage must be authorised

ICBP
International Cancer
Benchmarking Partnership

1. Duration of symptoms

Please estimate how long your patient had symptom(s), attributable to lung cancer, before attending your practice (or other health service).

We appreciate that identifying a 'date of first symptom' is not always straightforward – particularly when there are multiple and/or chronic symptoms. Nevertheless, we hope you can provide a 'best estimate'.

**Estimate of symptom duration** (please tick **one**):	✓	**What were the symptoms?** Please describe:
Less than 1 week		
1 to 4 weeks		
5 to 7 weeks		
2-5 months		
6-12 months		
More than 12 months		
Not possible to estimate		
No symptoms (e.g. screen detected cancers)		

Sample

Copyright property of the International Benchmarking Partnership (ICBP) and Module 4 collaborators. Usage must be authorised

ICBP
International Cancer Benchmarking Partnership

2. Pathway of presentation

2.1 Through what route did the patient first present? Please tick **ONE**:

	✓	
Your patient first presented to primary care (either in-hours or out-of-hours)		Please can you provide your best approximation of the date of this **primary care** visit<br>D D M M Y Y Y Y
Your patient presented straight to A&E (with or without your involvement)		
Your patient first presented to primary care, but then at a later date presented to A&E as an emergency (with or without your involvement)		Please can you provide your best approximation of the date of this **primary care** visit<br>D D M M Y Y Y Y
Other – please describe:		

Sample

Copyright property of the International Benchmarking Partnership (ICBP)
and Module 4 collaborators. Usage must be authorised

ICBP International Cancer Benchmarking Partnership

3. Date you ordered any tests/investigations in response to symptom(s).

We are interested in any kind of tests/investigations (e.g. imaging etc) that you may have ordered. Please only consider the tests/investigations that you ordered yourself. Please tick **all** that apply and put in the date that the test/investigation was ordered:

	✓	
Chest x-ray		D D M M Y Y Y Y
MRI scan		D D M M Y Y Y Y
CT scan		D D M M Y Y Y Y
PET scan		D D M M Y Y Y Y
Sputum test		D D M M Y Y Y Y
Lung biopsy		D D M M Y Y Y Y
Bronchoscopy		D D M M Y Y Y Y
Other (please specify):		D D M M Y Y Y Y

Sample

4. Date of referral to specialist medical services

At what date did you **first** refer the patient to hospital or another specialist transferring the responsibility for on-going investigation/treatment to other medical services?

D D M M Y Y Y Y

Copyright property of the International Benchmarking Partnership (ICBP) and Module 4 collaborators. Usage must be authorised

ICBP
International Cancer
Benchmarking Partnership

5. Nature of this referral

5.1 Do you know the date that the patient was seen for this referral?

☐ **Yes**, please provide the date: D D M M Y Y Y Y

☐ **No**

5.2 If you did make a referral to specialist services, which of the following best describes the nature/characteristics of this referral? Please tick **one**.

	✓
Emergency admission: a referral to A&E (or equivalent) for immediate admission	
An urgent referral for assessment of cancer symptoms/signs/test results (Note this will be within 2 weeks for England/Wales)	
A less urgent referral in which cancer is raised as a possibility (Note this will be greater than 2 weeks for England/Wales)	
A more general referral for investigation and assessment without cancer mentioned	
No referral was made	
Other – please describe	

Sample

5.3 Would you say this patient's diagnostic pathway was conducted predominantly in the public or private system? Please tick **one**.

	✓
Public healthcare system	
Private healthcare system	

Copyright property of the International Benchmarking Partnership (ICBP) and Module 4 collaborators. Usage must be authorised

ICBP
International Cancer
Benchmarking Partnership

6. Date of lung cancer diagnosis

This can be decided in different ways. Please tick **all** that apply.
Please provide whichever of the following dates you have to hand:

	✓	
Date of histological confirmation [ideal]		D D M M Y Y Y Y
Date results of investigation (histological or other) confirming cancer received		D D M M Y Y Y Y
Date patient was told		D D M M Y Y Y Y
Date biopsy undertaken		D D M M Y Y Y Y
Date patient was first admitted to hospital because of the malignancy		D D M M Y Y Y Y
Other (please specify)		D D M M Y Y Y Y

Sample

Copyright property of the International Benchmarking Partnership (ICBP)
and Module 4 collaborators. Usage must be authorised

ICBP
International Cancer Benchmarking Partnership

7. Additional information

Finally, we are interested to know what other conditions your patient has, and the severity/impact of these conditions

Have you and/or any of your partners treated this patient (or has the patient been to hospital) for any of the following conditions? Please tick **all** that apply:

Cardiovascular disease	☐ Yes ☐ No
Stroke	☐ Yes ☐ No
Lung disease (except lung cancer)	☐ Yes ☐ No
Diabetes	☐ Yes ☐ No

Sample

Are there any other comments you would like to make about this patient?

Name (and title):

Signature:

Date:

Thank you very much for taking the time to complete this questionnaire.

Copyright property of the International Benchmarking Partnership (ICBP)
and Module 4 collaborators. Usage must be authorised

A3: Cancer treatment specialists (CTS) questionnaire

ICBP
International Cancer Benchmarking Partnership

International Cancer Benchmarking Partnership Module 4

Specialist Care Audit
Lung Cancer

Thank you very much for agreeing to fill in this questionnaire – it should take about 10 minutes to complete. As part of an international study examining differences in cancer survival, we are sending the questionnaire to health care providers of a sample of patients with cancer.

Our aim is to gain a better understanding of the process by which people have their cancer diagnosed – the symptoms they experience, and the pathway they follow from onset of symptoms to treatment of their cancer. We hope you can help us with information on this patient's cancer journey once they were referred to specialist cancer services. This will help in identifying ways in which cancers can be diagnosed and treated quickly and effectively.

Sample

Thank you once again for your time

Please can you refer to your patient's notes in completing the questionnaire, as this will help in obtaining accurate data on time points.

If you would prefer to return this questionnaire without the patient details, please tear off along the dotted line.

Your patient ______________________________

is participating in the study.

Copyright property of the International Benchmarking Partnership (ICBP) and Module 4 collaborators. Usage must be authorised

ICBP
International Cancer Benchmarking Partnership

Sample

Patient information

ID-number: Jurisdiction-ID + Patient-ID:

Full name:

Address:

Postcode:

Date of birth: D D M M Y Y Y Y

Copyright property of the International Benchmarking Partnership (ICBP) and Module 4 collaborators. Usage must be authorised

ICBP International Cancer Benchmarking Partnership

1. **Date patient first attended hospital/specialist services related to their cancer diagnosis.** We appreciate this date can at times be difficult to identify, particularly when there have been multiple visits in the lead up to a definitive diagnosis. Put another way, it's the date that the hospital/specialist service assumed responsibility for on-going investigation/treatment for your patient.

Day (optional), month, year

D	D	M	M	Y	Y	Y	Y

2. **How was the patient referred to the hospital/specialist services related to their cancer diagnosis?** Please tick.

Was it through a:

	✓		✓
GP referral		Referral from respiratory clinic	
Medical specialist/Consultant referral		Other referral – please specify:	

Sample

3. **Where did this first contact/appointment happen?** Please tick.
Which of the following best describes where this first contact/appointment took place?

	✓		✓
Emergency department ('A&E')		Medical outpatient department, please specify which department	
Oncology general outpatient department		Surgical outpatient department, please specify which department	
Other – please specify:			

Copyright property of the International Benchmarking Partnership (ICBP) and Module 4 collaborators. Usage must be authorised

ICBP
International Cancer Benchmarking Partnership

4. Date of diagnosis

This can be decided in different ways.
Please tick and complete as many of the following dates as possible.

	✓	
Date of histological confirmation (ideal)		Day (optional), month, year D D M M Y Y Y Y
Date results of investigation confirming cancer received		Day (optional), month, year D D M M Y Y Y Y
Date patient was told		Day (optional), month, year D D M M Y Y Y Y
Date of biopsy		Day (optional), month, year D D M M Y Y Y Y
Date patient was first admitted to hospital because of the malignancy		Day (optional), month, year D D M M Y Y Y Y
Date of MDT confirmation of diagnosis		Day (optional), month, year D D M M Y Y Y Y
Other (please specify):		Day (optional), month, year D D M M Y Y Y Y

Sample

Copyright property of the International Benchmarking Partnership (ICBP)
and Module 4 collaborators. Usage must be authorised

ICBP
International Cancer Benchmarking Partnership

5. Date treatment for the cancer commenced

Based on your records, when would you say that any treatment specifically targeting the patient's cancer started?

Day (optional), month, year

D	D	M	M	Y	Y	Y	Y

6. Additional information

Please can you provide any further information on the patient's cancer:

**TNM**, please tick as appropriate:	
0	
I	
IIA	
IIB	
IIC	
IIIA	
IIIB	
IIIC	
IV	
Not able to stage	

Sample

Copyright property of the International Benchmarking Partnership (ICBP) and Module 4 collaborators. Usage must be authorised

ICBP
International Cancer Benchmarking Partnership

6.1 Histological subtype:

	✓
Squamous cell carcinoma	
Adenocarcinoma	
Large cell carcinoma	
Non-small carcinoma	
Small cell carcinoma	
Carcinoid	
Unclassified	
Other (please specify):	

Sample

Copyright property of the International Benchmarking Partnership (ICBP)
and Module 4 collaborators. Usage must be authorised

Further comments

Sample

Copyright property of the International Benchmarking Partnership (ICBP)
and Module 4 collaborators. Usage must be authorised

ICBP
International Cancer Benchmarking Partnership

Name (and title):

Signature:

Date:

Sample

Are you a ... (please tick below): ✓

Surgeon	
Medical Oncologist	
Clinical Oncologist	
Clinical Nurse Specialist	
Other (please specify):	

Thank you very much for taking the time to complete this questionnaire.

Copyright property of the International Benchmarking Partnership (ICBP) and Module 4 collaborators. Usage must be authorised

Appendix B:

Rules for missing, incomplete, multiple response and out of range data

1. Oversampling
   To handle oversampling in Ontario, include only the first 315 consecutive lung cancer patients;

2. Language/Participation in study/Presence of cancer
   Exclude patients who checked "No, I don't understand the language" or "I don't want to participate in this study" or "I don't have cancer".

3. Survey responders
   a) Exclude Patient/PCP/Specialist survey from the analysis, if it was not written by Patient/PCP/Specialist (example: a medical oncologist completed a PCP survey);
   b) In the case of duplicates, include only the first survey (example: 2 specialists completed surveys for the same patient).

4. Gender
   Exclude patients with unknown Gender.

5. Age
   a) Exclude patients with unknown age;
   b) Exclude patients younger 40 years;
   c) Use registry data, if Age is reported by both patient and registry.

6. No cancer or Previous cancer in the same organ
   a) Exclude patients with no cancer based on registry data;
   b) Exclude patients with previous cancer in the same organ based on data from registry or free-text for Presentation in the patient survey.

7. Date of consent
   Exclude patients with date of consent which is unknown, before 01.01.2013 or in the future.

8. Multiple responses to Dates
   If multiple responses were given to the dates (of first symptom; first presentation to primary care; referral; diagnosis; treatment start), then use the earliest date.

9. Order of Dates
   The dates must be in the following order –

   First symptom; first presentation to Primary Care; referral; diagnosis; treatment start.

   If not, check for mistakes.

For peer review only - http://bmjopen.bmj.com/site/about/guidelines.xhtml

10. Date of first symptom
Date of first symptom is defined as date of first symptom from patient data.

11. Date of first presentation
Date of first presentation to Primary Care is defined as (in the order of declining priority):
a) date of first presentation to Primary Care from PCP data;
b) date of first presentation to Primary Care and A&E from PCP data;
c) date of first presentation to Primary Care from patient data.

12. Date of referral
Date of referral is defined as date of referral from PCP data.

13. Date of diagnosis
Definition
a) If Registry reports both date of histological confirmation and date of confirming investigation, then use date of histological confirmation.
b) Date of diagnosis (based on patient data, PCP data, specialist data, registry data) is defined as (in the order of declining priority):
- date of diagnosis from registry;
- date of histological confirmation (from specialist data, PCP data);
- date of biopsy (from specialist data, PCP data);
- date of confirming investigation (from specialist data, PCP data);
- date of first hospital admission (from specialist data, PCP data);
- date of MDT confirmation (from specialist data, PCP data);
- date patient was told (from specialist data, PCP data);
- other date of diagnosis (from specialist data, PCP data, patient data);

Choose a Date from a lower level of hierarchy, if the Date from a higher level is after the Date of consent or more than 9 months (=271 days) before the Date of consent.

Exclusion criteria

a) Unknown date of diagnosis;
b) Date of diagnosis is after the date of consent;
c) Date of diagnosis is more than 9 months before the Date of consent.

14. Date of treatment start
a) Date of treatment start from patient data is defined as the earliest of the treatment dates for Surgery, Chemo, Radio and Other (e.g. palliative care, participation in a clinical trial, targeted agents like erlotinib and procedures like plueral tap)

For peer review only - http://bmjopen.bmj.com/site/about/guidelines.xhtml

b) Date of treatment start (based on registry data, specialist data, patient data) is defined as (in the order of declining priority):
- date of treatment start from registry data,
- date of treatment start from specialist data,
- date of treatment start from patient data,
- anticipated date of treatment from patient data.

15. Imputation of missing day in the date

Imputation rules for missing day (given month and year are known):

a) Set missing day to '16';
b) Consider adjacent dates in a backwards order (from "Treatment" to "First symptom"). For each pair of such adjacent dates: If dates are not in a logical order (e.g. "Treatment" is before "Diagnosis"), but month and year are the same in both dates, and the day was imputed to '16' in one of the dates:
- Recode the day imputed earlier to '16' to the day from the adjacent date.

16. Considering time

If patient gave multiple answers to the "How long did you have symptoms before contacting a doctor?" question, then use the option with the shortest time interval.

17. Delay arranging appointment

If patient gave multiple answers to the "How long did it take to get an appointment with PCP?" question, then use the option with the shortest time interval.

18. Duration of symptoms

If PCP gave multiple answers to the "Duration of symptoms" question, then use the option with the shortest time interval.

19. Definition of Presentation

A. *Define Presentation within a Data Source*

1. Review the free-text for Presentation (Patient, PCP sources) and re-code, if possible.
2. If PCP reports 'Other' as Presentation and at least one symptom (or "Duration of Symptoms") or if Patient reports 'Other' as Presentation and at least one symptom (or date of first symptom or "Consider waiting time" or "Delay arranging appointment"), then re-code the Presentation in the corresponding data source to 'Unknown'- option.
3. In the case of multiple Presentation responses (Patient, PCP sources) - use a single option (in the order of declining priority):
   a) 'VisitPCP and AE',
   b) 'VisitPCP´, 'AE´ (if both 'VisitPCP´ and 'AE´ are given, then re-code as 'VisitPCP and AE'),
   c) 'Unknown',
   d) 'Investigation for another problem' ,
   e) 'Other"

For peer review only - http://bmjopen.bmj.com/site/about/guidelines.xhtml

B. *Define Presentation from Alternative Data*

If Presentation hasn't been reported in either of data sources, then define it 'Unknown',

if PCP reports at least one symptom (or "Duration of symptoms"); or if Patient reports

at least one symptom (or date of first symptom or "Consider waiting time" or "Delay arranging

appointment");

C. *Define Presentation from Data Source Hierarchy*

1. In all jurisdictions, except Sweden – use Presentation data from (in the order of declining priority):
   a) PCP data;
   b) Patient data;
2. In Sweden – use Presentation data from Patient data.

20. Patient interval

The Patient interval is defined as (in the order of declining priority):

a) "Date of first presentation to Primary Care" (rule 11) minus "Date of first symptom" (rule 10);
b) If the interval in (a) is unknown or negative: Calculate the interval as the low boundary of "Considering time" (rule 16) plus the low boundary of "Delay arranging appointment" (rule 17);
c) If the interval in (a) is unknown or negative and the interval in (b) is unknown: Calculate the interval as the low boundary of "Duration of symptoms interval" (rule 18).

21. Primary Care interval

The Primary Care interval is defined as "Date of referral" (rule 12) minus "Date of first presentation to Primary Care" (rule 11).

22. Diagnostic interval

a) The Diagnostic interval is defined as "Date of diagnosis" (rule 13) minus "Date of first presentation to Primary Care" (rule 11).

23. Treatment interval

The Treatment interval is defined as "Date of treatment start" (rule 14) minus "Date of diagnosis" (rule 13).

24. Total interval

a) The Total interval is defined as "Date of treatment start" (rule 14) minus "Date of first symptom" (rule 10).

25. Range of Time intervals

The time intervals (Patient, Primary Care, Diagnosis, Treatment, Total) must be in range 0-1 year.

If > 1 year: set the interval to 365 days

For peer review only - http://bmjopen.bmj.com/site/about/guidelines.xhtml

If negative: set the interval to 0.
26. Number of visits<br>If patient gave multiple answers to the "Number of visits" questions, then use the option with a fewer number of visits.
27. Specialist waiting time interval<br>If patient gave multiple answers to the ""How long did it take to get an appointment with specialist?" question, then use the option with the shortest time interval.
28. Type of treatment<br>If patient ticked both "Yes" and "No" as answers to the "Type of treatment (Surgery, Chemotherapy, Radiotherapy)" questions, then choose "Yes" answer.
29. Health state<br>If patient gave multiple answers to the "Health state" question, then use the option with a better health condition.
30. Comorbidity<br>a) If patient ticked both "Yes" and "No" as answers to the "Comorbidity (Heart disease, Stroke, Lung disease, Diabetes)" questions, then choose "Yes" answer;<br>b) If both patient and PCP report "Comorbidity", then use the PCP Data.
31. Ethnicity<br>a) If patient didn't report "Ethnicity", then use the information from (in the order of declining priority):<br>- "Ethnicity_Other_Details";<br>- "Other main language spoken at home";<br>- "The main language spoken at home" (only for Victoria);<br>- "The main language spoken at home is the chief one for this jurisdiction"="Yes" given "Main language spoken at home is other than the main one for this jurisdiction"="No";<br>b) Consider Ethnicity as unknown, if answers to the "Ethnicity" question are multiple and belong to<br>different categories ('white', 'Asian', 'black', 'other').
32. Education<br>If patient gave multiple answers to the "Education" question, then use the option with a higher level of education.
33. Smoking Current<br>a) If patient ticked both "Yes" and "No" as answers to the "Smoking Current" question, then use "Yes" answer;<br>b) If patient hasn't ticked neither "Yes" nor "No, then consider this case as Unknown.

For peer review only - http://bmjopen.bmj.com/site/about/guidelines.xhtml

For peer review only

34. Smoking Number

If patient reports "SmokingNumber" as text, then re-code using following rules:

a) Where there is a number smoked /day – accept number;

b) Where a range has been given – take the upper value;

c) Where patient has put 10+ or 20+ - capture this as 11 or 21;

d) Where number of cigarettes smoked in the past and currently being smoked are provided - average the numbers;

e) Non entries code as "." ;

f) Non-smokers (eg, "nil", "N/A") are coded as "0".

35. Smoked ever

a) If patient ticked both "Yes" and "No" as answers to the "Smoking ever" question, then use "Yes" answer;

b) If patient hasn't ticked neither "Yes" nor "No": consider it as "Yes", if patient is a current smoker ("Smoking_Current="Yes"") or has specified a number of cigarettes ("SmokingNumber">0). Otherwise consider this case as Unknown.

c) If patient has ticked "No": recode it to "Yes", if patient is a current smoker ("Smoking_Current="Yes").

36. Nature of referral

a) Review free-text for " Nature of referral" (PCP Data) and re-code, if possible;

b) In the case of multiple responses, use a single option as (in the order of declining priority):

- "Referral for immediate admission";
- "Urgent referral";
- "Less urgent referral";
- "General referral" ;
- "No referral";
- "Other".

37. Refer Public or Private

a) If PCP ticked both "Public" and "Private" as answers to the "Refer Public or Private" question, then use "Private" answer;

b) If PCP hasn't ticked neither "Public" nor "Private", then consider this case as Unknown.

38. Type of referral

If specialist gave multiple responses to the "How was the patient referred…" question, then use a single option (in the order of declining priority):

- "Respiratory clinic";
- "Specialist/consultant";
- "PCP";
- "Other".

For peer review only - http://bmjopen.bmj.com/site/about/guidelines.xhtml

39. First Attendance Place

If specialist gave multiple responses to the "First Attendance Place" question, then consider this case as Unknown.

40. Stage-TNM

a) If specialist gave multiple responses to the "Stage_TNM" question, then use the highest category;
b) If registry gave multiple responses to the "Stage_TNM", then use a single option (in the order of declining priority):
   - stage at time of diagnosis
   - stage at surgery
   - stage at oncology
c) If "Stage_TNM" is reported by both the specialist and registry, then use the registry data.

For peer review only

For peer review only - http://bmjopen.bmj.com/site/about/guidelines.xhtml

Appendix C: Supplementary Tables

C 1: Classification of lung cancer -specific symptoms reported by patients into cancer specific and non- cancer specific

Cancer specific symptoms
1 persistent cough
2 chest infection that wouldn't get better
3 coughing up blood-stained phlegm (sputum)
4 breathlessness
5 chest or shoulder pain
6 a hoarse voice
7 difficulty swallowing
8 swelling of lymph nodes (glands) in my neck
9 ends of fingers becoming larger
10 breast and axilla swelling (in men)

Non-cancer specific symptoms
1 a dull ache or sharp pain when I coughed or took a deep breath
2 pain/discomfort under my ribs
3 face swelling
4 blood clots (thrombosis)
5 pins and needles or numbness in fingers
6 weight loss
7 felt sick/vomiting/nausea/loss of appetite
8 fatigue
9 muscle weakness
10 drowsiness, weakness, dizziness or confusion
11 high temperature (fever) of 38C (100.4F)
12 other

C2: Time intervals (days) depicted as median, 75th and 90th centiles for each of the ten jurisdictions. In Sweden, no data was available on primary care interval

For peer review only

For peer review only - http://bmjopen.bmj.com/site/about/guidelines.xhtml

Intervals	Centiles	Wales	Denmark	Sweden	England	N Ireland	Scotland	Manitoba	Ontario
Patient Interval	Number	181	233	172	213	179	169	133	205
	Median	21	14	21	17	18	21	25	22
	75th centile	61	53	61	65	60	60	67	61
	90th centile	216	180	214	205	240	267	180	187
Primary Care interval	Number	110	159	n/a	147	124	119	80	75
	Median	20	7		11	13	16	30	29
	75th centile	43	20		31	51	35	75	73
	90th centile	91	64		73	112	90	138	183
Diagnostic interval	Number	176	229	165	212	170	173	138	212
	Median	45	35	28	54	65	42	87	57
	75th centile	108	67	83	100	122	106	147	122
	90th centile	162	162	143	161	281	198	265	331
Treatment interval	Number	192	279	190	238	200	187	182	263
	Median	43	16	34	22	32	42	19	47
	75th centile	64	25	59	41	48	62	56	70
	90th centile	89	37	77	56	72	90	97	96
Total interval	Number	147	192	147	176	157	143	117	178
	Median	116	67	107	114	105	117	127	130
	75th centile	204	116	190	183	227	253	216	216
	90th centile	365	210	329	323	365	365	365	339

For peer review only

C3: Data sources used to define dates and percentage of imputed dates

Type of date	Data sources used to define a date* (%)				Cases with imputed day in a date** (%)
	Patient	PCP	CST	Registry	
First noticing symptoms	100	0	0	0	66
First presentation to health care	49	51	0	0	30
First referral to secondary care	0	100	0	0	1
Diagnosis	5	6	8	81	1
Start of curative or palliative treatment	55	0	32	13	11

* based on rules 10-14, supplementary file Appendix B
** based on rule 15, supplementary file Appendix B
*** Registry/CST medical records on date of treatment were not available for 55% patients, therefore an alternative data source (patient survey) was used instead

For peer review only

C4. Percentages of negative intervals set to 0, large intervals (>365 days) set to 365 days, and intervals based not on dates

**Type of interval**	**Negative intervals set to 0 days* (%)**	**Intervals >365 days set to 365 days* (%)**	**Intervals where variables other than dates were used (%)****
Patient	<1	5	29
Primary care	4	2	0
Diagnosis	6	5	0
Treatment	6	<1	0
Total	2	9	0

* based on rule 25, supplementary file Appendix B
* based on rule 20b,c, supplementary file Appendix B

For peer review only

For peer review only

For peer review only - http://bmjopen.bmj.com/site/about/guidelines.xhtml

The RECORD statement – checklist of items, extended from the STROBE statement, that should be reported in observational studies using routinely collected health data.

	Item No.	STROBE items	Location in manuscript where items are reported	RECORD items	Location in manuscript where items are reported
**Title and abstract**					
	1	(a) Indicate the study's design with a commonly used term in the title or the abstract (b) Provide in the abstract an informative and balanced summary of what was done and what was found	Title and abstract – p 1 and 3	RECORD 1.1: The type of data used should be specified in the title or abstract. When possible, the name of the databases used should be included.<br><br>RECORD 1.2: If applicable, the geographic region and timeframe within which the study took place should be reported in the title or abstract.<br><br>RECORD 1.3: If linkage between databases was conducted for the study, this should be clearly stated in the title or abstract.	Abstract – p 3<br><br>Abstract – p 3<br><br>N/A
**Introduction**					
Background rationale	2	Explain the scientific background and rationale for the investigation being reported	Introduction – p 6		
Objectives	3	State specific objectives, including any prespecified hypotheses	Introduction – p 6		
**Methods**					
Study Design	4	Present key elements of study design early in the paper	Methods – p 6		
Setting	5	Describe the setting, locations, and relevant dates, including periods of recruitment, exposure, follow-up, and data collection	Methods – p 6-8		
Participants	6	*(a) Cohort study* - Give the eligibility criteria, and the	Methods – p 6 – and as reference to	RECORD 6.1: The methods of study population selection (such as codes or	Provided as reference to

For peer review only - http://bmjopen.bmj.com/site/about/guidelines.xhtml

		sources and methods of selection of participants. Describe methods of follow-up<br>*Case-control study* - Give the eligibility criteria, and the sources and methods of case ascertainment and control selection. Give the rationale for the choice of cases and controls<br>*Cross-sectional study* - Give the eligibility criteria, and the sources and methods of selection of participants<br><br>*(b) Cohort study* - For matched studies, give matching criteria and number of exposed and unexposed<br>*Case-control study* - For matched studies, give matching criteria and the number of controls per case	previous paper.<br><br>N/A	algorithms used to identify subjects) should be listed in detail. If this is not possible, an explanation should be provided.<br><br>RECORD 6.2: Any validation studies of the codes or algorithms used to select the population should be referenced. If validation was conducted for this study and not published elsewhere, detailed methods and results should be provided.<br><br>RECORD 6.3: If the study involved linkage of databases, consider use of a flow diagram or other graphical display to demonstrate the data linkage process, including the number of individuals with linked data at each stage.	previous paper.<br><br>Provided as appendix and in reference to previous paper.<br><br>N/A
Variables	7	Clearly define all outcomes, exposures, predictors, potential confounders, and effect modifiers. Give diagnostic criteria, if applicable.	Methods – p 6-9 – and as reference to previous paper.	RECORD 7.1: A complete list of codes and algorithms used to classify exposures, outcomes, confounders, and effect modifiers should be provided. If these cannot be reported, an explanation should be provided.	N/A
Data sources/ measurement	8	For each variable of interest, give sources of data and details of methods of assessment (measurement).<br>Describe comparability of assessment methods if there is more than one group	Methods – p 6-9 – and as reference to previous paper.		
Bias	9	Describe any efforts to address potential sources of bias	Methods – p 7-8, discussion – p 24-25 – and as reference to		

For peer review only

			previous paper.		
Study size	10	Explain how the study size was arrived at	Methods – p 6 – and as reference to previous paper.		
Quantitative variables	11	Explain how quantitative variables were handled in the analyses. If applicable, describe which groupings were chosen, and why	Methods – p 6-8 – and as reference to previous paper.		
Statistical methods	12	(a) Describe all statistical methods, including those used to control for confounding<br>(b) Describe any methods used to examine subgroups and interactions<br>(c) Explain how missing data were addressed<br>(d) *Cohort study* - If applicable, explain how loss to follow-up was addressed<br>*Case-control study* - If applicable, explain how matching of cases and controls was addressed<br>*Cross-sectional study* - If applicable, describe analytical methods taking account of sampling strategy<br>(e) Describe any sensitivity analyses	Methods – p 8 – and as reference to previous paper.		
Data access and cleaning methods		..		RECORD 12.1: Authors should describe the extent to which the investigators had access to the database population used to create the study population.	N/A

For peer review only

				RECORD 12.2: Authors should provide information on the data cleaning methods used in the study.	Methods – p 7
Linkage		..		RECORD 12.3: State whether the study included person-level, institutional-level, or other data linkage across two or more databases. The methods of linkage and methods of linkage quality evaluation should be provided.	N/A
**Results**					
Participants	13	(a) Report the numbers of individuals at each stage of the study (*e.g.*, numbers potentially eligible, examined for eligibility, confirmed eligible, included in the study, completing follow-up, and analysed)<br>(b) Give reasons for non-participation at each stage.<br>(c) Consider use of a flow diagram	Results and as table – p 10-11. Flow diagram in previous paper, referenced.	RECORD 13.1: Describe in detail the selection of the persons included in the study (*i.e.,* study population selection) including filtering based on data quality, data availability and linkage. The selection of included persons can be described in the text and/or by means of the study flow diagram.	Results – p 10-15 - and as table. Flow diagram in previous paper, referenced.
Descriptive data	14	(a) Give characteristics of study participants (*e.g.*, demographic, clinical, social) and information on exposures and potential confounders<br>(b) Indicate the number of participants with missing data for each variable of interest<br>(c) *Cohort study* - summarise follow-up time (*e.g.*, average and total amount)	Results and as table – p 10-15.		
Outcome data	15	*Cohort study* - Report numbers of outcome events or summary measures over time<br>*Case-control study* - Report numbers in each exposure category, or summary measures	Results and as table – p 16-23.		

For peer review only

		of exposure<br>*Cross-sectional study* - Report numbers of outcome events or summary measures			
Main results	16	(a) Give unadjusted estimates and, if applicable, confounder-adjusted estimates and their precision (e.g., 95% confidence interval). Make clear which confounders were adjusted for and why they were included<br>(b) Report category boundaries when continuous variables were categorized<br>(c) If relevant, consider translating estimates of relative risk into absolute risk for a meaningful time period	Results and as table – p 21-23.		
Other analyses	17	Report other analyses done—e.g., analyses of subgroups and interactions, and sensitivity analyses	Results – p 23.		
**Discussion**					
Key results	18	Summarise key results with reference to study objectives	Discussion – p 24		
Limitations	19	Discuss limitations of the study, taking into account sources of potential bias or imprecision. Discuss both direction and magnitude of any potential bias	Discussion – p24-25	RECORD 19.1: Discuss the implications of using data that were not created or collected to answer the specific research question(s). Include discussion of misclassification bias, unmeasured confounding, missing data, and changing eligibility over time, as they pertain to the study being reported.	Discussion – p24-25
Interpretation	20	Give a cautious overall interpretation of results considering objectives, limitations, multiplicity of analyses, results from similar	Discussion – p25-28		

For peer review only

		studies, and other relevant evidence			
Generalisability	21	Discuss the generalisability (external validity) of the study results	Discussion, conclusion – p25-29.		
**Other Information**					
Funding	22	Give the source of funding and the role of the funders for the present study and, if applicable, for the original study on which the present article is based	Funding statement – p 31		
Accessibility of protocol, raw data, and programming code		..		RECORD 22.1: Authors should provide information on how to access any supplemental information such as the study protocol, raw data, or programming code.	Availability of data and material statement – p 31

*Reference: Benchimol EI, Smeeth L, Guttmann A, Harron K, Moher D, Petersen I, Sørensen HT, von Elm E, Langan SM, the RECORD Working Committee. The REporting of studies Conducted using Observational Routinely-collected health Data (RECORD) Statement. *PLoS Medicine* 2015; in press.

*Checklist is protected under Creative Commons Attribution (CC BY) license.

For peer review only
